# Supplementary material for: In silico simulation of a clinical trial with anti-CTLA-4 and anti-PD-L1 immunotherapies in metastatic breast cancer using a systems pharmacology model
Source: R Soc Open Sci. 2019 May 22;6(5):190366. doi: 10.1098/rsos.190366 (PMC6549962; doi:10.1098/rsos.190366)
Supplement: Supplementary Material [file rsos190366supp1.docx]

Supplementary Material

**In silico simulation of a clinical trial in metastatic breast cancer with combination of PD-L1 and CTLA-4 immunotherapies using a systems pharmacology model**

**Hanwen Wang^1*^, Oleg Milberg^1^, Imke H. Bartelink^2,3,4^, Paolo Vicini^5^, Bing Wang^6^, Rajesh Narwal^7^, Lorin Roskos^7^, Cesar A. Santa-Maria^8^, Aleksander S. Popel^1,8^**

*** Correspondence:** Hanwen Wang: hwang163@jhu.edu

S0 – Permeability between the blood and the peripheral/tumor tissues

S1 (Table)– Permeability-surface area product in clinical studies of multiple tumor types

S1 (Figure) – Waterfall plot

S2 (Figure) – Personalized prediction with only changing tumor growth rate

S3 (Figure) – Personalized prediction with only changing antigen intensity

S4 (Figure) – Personalized prediction with only changing PD-L1 expression

S5 (Figure) – Optimization results

S2 (Table) – Kinetic parameters of antibody PK

S3 (Table) – Definition of species in the model

S4 (Table) – Model Reaction

S5 (Table) – Model Reaction Rates

S6 (Table) – Model Reaction and Rate Descriptions

S7 (Table) – Model Parameters

S8 (Table) – Model Algebraic Equations

S9 (Table) – Model Discontinuous Equation Sets

**S0. Permeability between the blood and the peripheral/tumor tissues**

The permeability of the antibody between the peripheral tissues and capillaries can be estimated by its Stokes-Einstein radius, which is calculated via the equation by *Venturoli* *et al*: $a_{e}=0.483{*\left( MW \right)}^{0.386}$ [1]. For example, durvalumab, which has a molecular weight of 146.3 kDa, has a Stokes-Einstein radius of 47.6 Å. The calculated radius is used to determine the permeability-surface area product as 1.5e-4 mL/(s*100g) [2]. With a surface area of 70 cm^2^/g, the permeability of durvalumab between the blood and the peripheral compartment is calculated to be 2e-8 cm/s. For antibody transport in this study, the permeability between the blood and the peripheral compartment is optimized within a realistic range. The permeability of antibody between the blood and the tumor compartments is estimated to be 3e-7 cm/s according to multiple literature evidence [3, 4], and the surface area is estimated to be 28.4 cm^2^/cm^3^ [4]. The published permeability-surface area product in multiple tumor types are listed in Table S2.

The clearance rate, volume fraction of plasma and interstitial space in peripheral tissues available to each antibody are optimized using published two-compartment PK model [5, 6]. The transport equation between central and peripheral compartment used in the published model is shown in equation (1,2), and that used in our model is shown in equation (3,4).

$\hat{V}_{B}*\frac{d\left[ A \right]_{B}}{dt}=Q*\left( \left[ A \right]_{P}-\left[ A \right]_{B} \right)-\hat{Cl}*\left[ A \right]_{B}$ (1)

$\hat{V}_{P}*\frac{d\left[ A \right]_{P}}{dt}=Q*\left( \left[ A \right]_{B}-\left[ A \right]_{P} \right)$ (2)

Where $\hat{V}$ is effective compartment volume, [*A*] is antibody concentration, *Q* is inter-compartment clearance rate, and $\hat{Cl}$ is clearance rate. Subscript B and P represent blood and peripheral compartment, respectively. Three dimensionless parameters are deduced as below,

$$\alpha=\frac{\hat{V}_{P}}{\hat{V}_{B}}; \beta=\frac{{\hat{Cl}*\hat{V}}_{P}}{Q}; \gamma=\frac{\hat{V}_{P}}{Q}$$

$K_{B}{*V}_{B}*\frac{d\left[ A \right]_{B}}{dt}=k_{Ab, BP}*SA_{BP}*\left( \left[ A \right]_{P}-\left[ A \right]_{B} \right)-Cl*K_{B}*\left[ A \right]_{B}$ (3)

$K_{P}{*V}_{P}*\frac{d\left[ A \right]_{P}}{dt}=k_{Ab, BP}*SA_{BP}*\left( \left[ A \right]_{B}-\left[ A \right]_{P} \right)$ (4)

Where $K$ is volume fraction of interstitial space, [*A*] is antibody concentration, *V* is volume, *k* is permeability, *SA* is surface area, and *Cl* is clearance rate. Subscript B and P represent blood and peripheral compartment, respectively. Three dimensionless parameters are deduced as below,

$$\alpha=\frac{K_{P}*V_{P}}{K_{B}*V_{B}}; \beta=\frac{Cl*V_{P}}{k*SA}; \gamma=\frac{K_{P}*V_{P}}{k*SA}$$

In order to have a perfect fit of the plasma concentration, the three dimensionless parameters must be equal to each other, which is used to estimate surface area to volume ratio in peripheral tissues.

**Table S1. Permeability-surface area product in clinical studies of multiple tumor types**

| Tumor Type | Methods | Baseline tumor mean permeability surface area product (mL/min/100 g) | Calculated surface area to volume ratio using estimated permeability with the above method [2] (cm^2^/cm^3^) | Reference |
| --- | --- | --- | --- | --- |
| Metastatic Uveal Melanoma at liver | CT Perfusion (ultravist 370 mg/mL) | 57 | 94.4 | [7] |
| Neuroendocrine liver metastases | CT Perfusion | 55.39 | 91.8 | [8] |
| Prostate Cancer | CT Perfusion | 26.34 | 43.6 | [9] |
| Metastatic carcinoid melanoma at liver/abdominal lymph node | CT Perfusion (ioversol, 320 mg of iodine/100 mL) | 20.7 | 34.3 | [10] |
| Advanced non–small cell lung cancer | CT Perfusion | 14.57 | 24.1 | [11] |
| Lung tumors larger than 25 mm in longest axial diameter | CT Perfusion (320 mg of iodine/100 mL) | 24.9 | 41.3 | [12] |
| Brain gliomas and metastases | CT Perfusion | 7.1 | 11.8 | [13] |

**Figure S1. Waterfall Plot of Combination Therapy with Different a) Tumor Size, b) PD-L1 Expression, and c) Antigen Intensity.**

**Figure S2. Prediction of Tumor Response with only Changing Tumor Growth Rate.**

**Figure S3. Prediction of Tumor Response with only Changing Antigen Intensity.**

**Figure S4. Prediction of Tumor Response with only Changing PD-L1 Expression on Tumor Cells.**

**Figure S5. Optimization Result. Serum concentration of a) Q4W 1 mg/kg tremelimumab b) Q4W 10 mg/kg durvalumab.**

**Table S2. Kinetic Parameters of Antibody Transport (Start)**

| Variable | Value | Unit | Source | Description |
| --- | --- | --- | --- | --- |
| V_Blood_ | 6 | L | Estimated | Total Blood volume |
| Cl_CTLA4 | 0.395915055274963 | L/day | Optimized  [5] | Clearance rate of tremelimumab |
| Cl_PDL1 | 0.407977128028870 | L/day | Optimized  [6] | Clearance rate of durvalumab |
| K_B_CTLA4 | 0.652704873681068 | cm^3^/cm^3^ | Optimized  [5] | Volume fraction of plasma in blood available to tremelimumab |
| K_B_PDL1 | 0.584437051415443 | cm^3^/cm^3^ | Optimized  [6] | Volume fraction of plasma in blood available to durvalumab |
| K_P_CTLA4 | 0.058274932943285 | cm^3^/cm^3^ | Optimized  [5] | Volume fraction of interstitial space in periphery available to tremelimumab |
| K_P_PDL1 | 0.056224055394530 | cm^3^/cm^3^ | Optimized  [6] | Volume fraction of interstitial space in periphery available to durvalumab |
| V_Peripheral_ | 61.321 | L | [14] | Total peripheral volume |
| V_Tumor_ | 10 | mL | Estimated | Total tumor volume used for antibody PK |
| V_tdln_ | 1 | mL | Estimated  [15] | Total volume of each TDLN |
| K_LN | 0.20 | cm^3^/cm^3^ | Estimated | Volume fraction of interstitial space in TDLN available to any antibody |
| K_T | 0.522024 | cm^3^/cm^3^ | [16] | Volume fraction of interstitial space in tumor available to any antibody |
| SA_BT | 28.4 | cm^2^/cm^3^ | [4] | Surface area of microvessels in tumor |
| SA_BP | 8.48 | cm^2^/cm^3^ | Optimized  [5, 6] | Surface area of microvessels in normal tissues |
| k_Lt | 0.0015 | 1/min | [17] | Lymphatic flow rate |
| k_Ab_BT | 3e-7 | cm/s | [4, 16] | Microvascular permeability of antibody in tumor |
| k_CTLA4_BP | 1.0e-8 | cm/s | Optimized  [2, 5] | Microvascular permeability of tremelimumab between central and peripheral compartment |
| k_PDL1_BP | 1.05905278e-8 | cm/s | Optimized  [2, 5] | Microvascular permeability of durvalumab between central and peripheral compartment |
| k_Ab_BLN | 3.83e-7 | cm/s | [18] | Microvascular permeability of antibody in TDLNs |

**Table S2. Kinetic Parameters of Antibody Transport (End)**

**Table S3 – Definition of species in the model (Start)**

| Variable Number | Compartment | Variable Name | Unit | Variable Definition |
| --- | --- | --- | --- | --- |
| 1 | Blood-Lymph | CTLA4_mabB | mole/L | Anti-CTLA-4 antibody in the blood |
| 2 | Blood-Lymph | CTLA4_mabB_ugml | µg/ml | Serum concentration of Anti-CTLA-4 |
| 3 | Blood-Lymph | PD1_mabB | mole/L | Anti-PD-1 antibody in the blood |
| 4 | Blood-Lymph | PDL1_mabB | mole/L | Anti-PD-L1 antibody in the blood |
| 5 | Blood-Lymph | PDL1_mabB_ugml | µg/ml | Serum concentration of Anti-PD-L1 |
| 6 | Blood-Lymph | PD1_mabB_ugml | µg/ml | Serum concentration of Anti-PD-1 |
| 7 | Blood-Lymph | Effector_TB | cell | Effector T cells that have been generated from all the lymph nodes considered that have migrated into the blood |
| 8 | Blood-Lymph | Effector_T_TOTAL | cell | The total number of Effector T cells present that have exited the lymph nodes |
| 9 | Blood-Lymph | Effector_T_TB | cell | Effector T cells that have been generated from all the lymph nodes considered that are in the process of migrating into the blood |
| 10 | Lymph_Node | Naive_T | cell | Naïve T cells that undergo the first phase of priming in the Lymph Nodes |
| 11 | Lymph_Node | Primed_Naive_T | cell | Primed Naive T cells that undergo the second phase of priming in the Lymph Nodes |
| 12 | Lymph_Node | PNT_Int_CD28 | mole | CD28 expressed by interacting Primed Naive T cells engaged in the second priming phase |
| 13 | Lymph_Node | mAPC | cell | Total number of mature antigen presenting cells (mAPCs) in the lymph node compartment |
| 14 | Lymph_Node | Effector_T | cell | Effector T cells that are generate in the lymph node compartment (considering one lymph node) |
| 15 | Lymph_Node | CTLA4_mab | mole/L | Anti-CTLA-4 antibody in the lymph node |
| 16 | Lymph_Node | Naive_T0 | cell | Naive T cells that can undergo priming in the lymph node |
| 17 | Lymph_Node | Prolif_Naive_T | cell | Proliferating Naive T cells that have undergone the second phase of priming in the lymph node |
| 18 | Lymph_Node | Anergic_Naive_T | cell | Primed Naive T cells that do not undergo proliferation following the second phase of priming and are deemed to be anergic T cells in the lymph node |
| 19 | Lymph_Node | PD1_mab | mole/L | Anti-PD-1 antibody in the lymph node |
| 20 | Lymph_Node | PDL1_mab | mole/L | Anti-PD-L1 antibody in the lymph node |
| 21 | Lymph_Node | Naive_T1 | cell | Naive T cells engaged in the first priming phase in the lymph node |
| 22 | Lymph_Node | Primed_Naive_T1 | cell | Primed Naive T cells engaged in the second priming phase in the lymph node |
| 23 | Lymph_Node | mAPC_Total_Calc_Pr | cell | mAPCs in the lymph node that are not engaged in priming |
| 24 | Lymph_Node | Total_T_CD8-CD4 | cell | Total number of all T cells in the lymph node |
| 25 | Lymph_Node | PNT1_Int | cell | Primed Naive T cells engaged in the second priming phase in the lymph node that are accounting for the expression of immune checkpoints |
| 26 | Lymph_Node | PNT_CTLA4 | mole | CTLA-4 expressed by interacting Primed Naive T cells engaged in the second priming phase |
| 27 | Lymph_Node | mAPC_Int_P1 | cell | mAPCs interacting with Naive T cells in the first phase of priming |
| 28 | Lymph_Node | mAPC_Int_P2 | cell | mAPCs interacting with Primed Naive T cells in the second phase of priming |
| 29 | Lymph_Node | mAPC_Int_P2_CD80 | mole | CD80 receptors expressed by mAPCs that are involved in the second priming phase interactions |
| 30 | Lymph_Node | mAPC_Int_P2_CD86 | mole | CD86 receptors expressed by mAPCs that are involved in the second priming phase interactions |
| 31 | Lymph_Node | POS_Sig_PNT_CD80 | mole | Receptor-receptor interactions at the immunological synapse between CD28 expressed on Primed Naive T cells and CD80 expressed on mAPCs during the second phase of priming |
| 32 | Lymph_Node | POS_Sig_PNT_CD86 | mole | Receptor-receptor interactions at the immunological synapse between CD28 expressed on Primed Naive T cells and CD86 expressed on mAPCs during the second phase of priming |
| 33 | Lymph_Node | NEG_Sig_PNT_CD80 | mole | Receptor-receptor interactions at the immunological synapse between CTLA-4 expressed on Primed Naive T cells and CD80 expressed on mAPCs during the second phase of priming |
| 34 | Lymph_Node | NEG_Sig_PNT_CD86 | mole | Receptor-receptor interactions at the immunological synapse between CTLA-4 expressed on Primed Naive T cells and CD80 expressed on mAPCs during the second phase of priming |
| 35 | Lymph_Node | CTLA4_mAb_CTLA4 | mole | Heterogeneous receptor-antibody interactions at the immunological synapse between CTLA-4 expressed on Primed Naive T cells during the second priming phase and Anti-CTLA-4 mAb delivered to the lymph node by way of IV injection into the blood |
| 36 | Lymph_Node | mAPC_Int_P2_PDL1 | mole | PD-L1 receptors expressed by mAPCs that are involved in the second priming phase interactions |
| 37 | Lymph_Node | mAPC_Int_P2_PDL2 | mole | PD-L2 receptors expressed by mAPCs that are involved in the second priming phase interactions |
| 38 | Lymph_Node | mAPC_Int_P2_PD1 | mole | PD-1 receptors expressed by mAPCs that are involved in the second priming phase interactions |
| 39 | Lymph_Node | PNT_Int_CD80 | mole | CD80 expressed by interacting Primed Naive T cells engaged in the second priming phase |
| 40 | Lymph_Node | PNT_Int_PD1 | mole | PD-1 expressed by interacting Primed Naive T cells engaged in the second priming phase |
| 41 | Lymph_Node | PNT_Int_PDL1 | mole | PD-L1 expressed by interacting Primed Naive T cells engaged in the second priming phase |
| 42 | Lymph_Node | PNT_CD80-PDL1 | mole | Receptor-receptor interactions at the immunological synapse between CD80 expressed on Primed Naive T cells and PD-L1 expressed on mAPCs during the second phase of priming |
| 43 | Lymph_Node | PNT_PD1-PDL2 | mole | Receptor-receptor interactions at the immunological synapse between PD-1 expressed on Primed Naive T cells and PD-L2 expressed on mAPCs during the second phase of priming |
| 44 | Lymph_Node | PNT_PD1-PDL1 | mole | Receptor-receptor interactions at the immunological synapse between PD-1 expressed on Primed Naive T cells and PD-L1 expressed on mAPCs during the second phase of priming |
| 45 | Lymph_Node | PNT_PDL1-CD80 | mole | Receptor-receptor interactions at the immunological synapse between PD-L1 expressed on Primed Naive T cells and CD80 expressed on mAPCs during the second phase of priming |
| 46 | Lymph_Node | PNT_PDL1-PD1 | mole | Receptor-receptor interactions at the immunological synapse between PD-L1 expressed on Primed Naive T cells and PD-1 expressed on mAPCs during the second phase of priming |
| 47 | Lymph_Node | mAPC_nInt | cell | mAPCs in the lymph node that are not engaged in priming |
| 48 | Lymph_Node | PDL1mAb-PNT_PDL1 | mole | Heterogenous receptor-antibody interactions at the immunological synapse between PD-L1 expressed on Primed Naive T cells during the second priming phase and Anti-PD-L1 mAb delivered to the lymph node by way of IV injection into the blood |
| 49 | Lymph_Node | PDL1mAb-mAPC_PDL1 | mole | Heterogeneous receptor-antibody interactions at the immunological synapse between PD-L1 expressed on mAPCs during the second priming phase and Anti-PD-L1 mAb delivered to the lymph node by way of IV injection into the blood |
| 50 | Lymph_Node | PD1mAb_mAPC_PD1 | mole | Heterogeneous receptor-antibody interactions at the immunological synapse between PD-1 expressed on mAPCs during the second priming phase and Anti-PD-1 mAb delivered to the lymph node by way of IV injection into the blood |
| 51 | Lymph_Node | PD1mAb_PNT_PD1 | mole | Heterogeneous receptor-antibody interactions at the immunological synapse between PD-1 expressed on Primed Naive T cells during the second priming phase and Anti-PD-1 mAb delivered to the lymph node by way of IV injection into the blood |
| 52 | Lymph_Node | mAPC_PD1 | mole | Total PD-1 expression by all mAPCs in the lymph node |
| 53 | Lymph_Node | mAPC_PDL2 | mole | Total PD-L2 expression by all mAPCs in the lymph node |
| 54 | Lymph_Node | mAPC_PDL1 | mole | Total PD-L1 expression by all mAPCs in the lymph node |
| 55 | Lymph_Node | mAPC_CD86 | mole | Total CD86 expression by all mAPCs in the lymph node |
| 56 | Lymph_Node | mAPC_CD80 | mole | Total CD80 expression by all mAPCs in the lymph node |
| 57 | Lymph_Node | TregLN_Int | cell | T Regulatory cells in the lymph node that have initiated engagement with mAPCs, and Naïve and Primed Naïve T cells |
| 58 | Lymph_Node | TregLN-NT | cell | T Regulatory cells in the lymph node that have engaged in interacting with Naïve T cells |
| 59 | Lymph_Node | TregLN-PNT | cell | T Regulatory cells in the lymph node that have engaged in interacting with Primed Naïve T cells |
| 60 | Lymph_Node | TregLN_mAPC | cell | T Regulatory cells in the lymph node that have engaged in interacting with mAPCs |
| 61 | Lymph_Node | TregLN | cell | Total number of T Regulatory cells that able to engage with other cell types in the lymph node |
| 62 | Lymph_Node | Tr-mAPC_CTLA4 | mole | CTLA-4 receptors expressed by T Regulatory cells in the lymph node |
| 63 | Lymph_Node | Tr-PNT_PD1 | mole | PD-1 receptors expressed by T Regulatory cells in the lymph node |
| 64 | Lymph_Node | Tr-PNT_PDL1 | mole | PD-L1 receptors expressed by T Regulatory cells in the lymph node |
| 65 | Lymph_Node | PNT-Tr_PDL1 | mole | PD-L1 receptors expressed by Primed Naïve T cells in the lymph node during engagement with T Regulatory cells |
| 66 | Lymph_Node | PNT-Tr_PD1 | mole | PD-1 receptors expressed by Primed Naïve T cells in the lymph node during engagement with T Regulatory cells |
| 67 | Lymph_Node | PNT-Tr_CD80 | mole | CD80 receptors expressed by Primed Naïve T cells in the lymph node during engagement with T Regulatory cells |
| 68 | Lymph_Node | TrALN_CT_CD80 | mole | Receptor-receptor interactions at the immunological synapse between CTLA-4 expressed on T Regulatory cells and CD80 expressed on mAPCs in the lymph node |
| 69 | Lymph_Node | TrALN_CT_CD86 | mole | Receptor-receptor interactions at the immunological synapse between CTLA-4 expressed on T Regulatory cells and CD86 expressed on mAPCs in the lymph node |
| 70 | Lymph_Node | TrPNT_PD1-L1 | mole | Receptor-receptor interactions at the immunological synapse between PD-1 expressed on T Regulatory cells and PD-L1 expressed on Primed Naïve T cells in the lymph node |
| 71 | Lymph_Node | TrPNT_PDL1-PD1 | mole | Receptor-receptor interactions at the immunological synapse between PD-L1 expressed on T Regulatory cells and PD-1 expressed on Primed Naïve T cells in the lymph node |
| 72 | Lymph_Node | TrPNT_PDL1-CD80 | mole | Receptor-receptor interactions at the immunological synapse between PD-L1 expressed on T Regulatory cells and CD80 expressed on Primed Naïve T cells in the lymph node |
| 73 | Lymph_Node | TrALN_CT_aCT | mole | Heterogeneous receptor-antibody interactions at the immunological synapse between CTLA-4 expressed on T Regulatory cells and Anti-CTLA-4 mAb delivered to the lymph node by way of IV injection into the blood |
| 74 | Lymph_Node | TrPNT_PD1_aPD1 | mole | Heterogeneous receptor-antibody interactions at the immunological synapse between PD-1 expressed on T Regulatory cells and Anti-PD-1 mAb delivered to the lymph node by way of IV injection into the blood |
| 75 | Lymph_Node | PNTTr_PDL1_aPDL1 | mole | Heterogeneous receptor-antibody interactions at the immunological synapse between PD-L1 expressed on Primed Naïve T cells and Anti-PD-L1 mAb delivered to the lymph node by way of IV injection into the blood |
| 76 | Lymph_Node | TrPNT_PDL1_aPDL1 | mole | Heterogeneous receptor-antibody interactions at the immunological synapse between PD-L1 expressed on T Regulatory cells and Anti-PD-L1 mAb delivered to the lymph node by way of IV injection into the blood |
| 77 | Lymph_Node | PNTTr_PD1_aPD1 | mole | Heterogeneous receptor-antibody interactions at the immunological synapse between PD-1 expressed on Primed Naïve T cells and Anti-PD-1 mAb delivered to the lymph node by way of IV injection into the blood |
| 78 | Lymph_Node | Tr-mAPC_CD80 | mole | CD80 receptors expressed by mACPs that are interacting with T Regulatory cells in the lymph node |
| 79 | Lymph_Node | Tr-mAPC_CD86 | mole | CD86 receptors expressed by mACPs that are interacting with T Regulatory cells in the lymph node |
| 80 | Lymph_Node | TregLN_mAPC1 | cell | T Regulatory cells in the lymph node that have engaged in interacting with mAPCs in the lymph node that are accounting for the expression of immune checkpoints |
| 81 | Lymph_Node | TregLN-PNT1 | cell | T Regulatory cells in the lymph node that have engaged in interacting with Primed Naïve T cells in the lymph node that are accounting for the expression of immune checkpoints |
| 82 | Lymph_Node | C_DebrisLN | cell | Cancer debris that has been transported to the lymph node |
| 83 | Lymph_Node | APCLN | cell | Resident antigen presenting cells in the lymph node that have not yet phagocytosed tumor debris |
| 84 | Lymph_Node | TrLN_CTLA4 | mole | CTLA-4 expressed on the surface of non-interacting T Regulatory cells |
| 85 | Lymph_Node | TrLN_CT_aCT | mole | Receptor-antibody interactions between CTLA-4 expressed on T Regulatory cells and Anti-CTLA-4 mAb delivered to the lymph node by way of IV injection into the blood |
| 86 | Lymph_Node | PNT1_Int1 | cell | Naive T cells undergoing the first priming phase in the lymph node that are accounting for the expression of immune checkpoints |
| 87 | Lymph_Node | NT_Int_CD28 | mole | CD28 expressed to the immunological synapse of Naive T cells that are undergoing the first phase of priming |
| 88 | Lymph_Node | mAPC_Int_P1_CD86 | mole | CD86 receptors expressed by mAPCs that are involved in the first priming phase interactions with Naïve T cells |
| 89 | Lymph_Node | mAPC_Int_P1_CD80 | mole | CD80 receptors expressed by mAPCs that are involved in the first priming phase interactions with Naïve T cells |
| 90 | Lymph_Node | POS_Sig_NT_CD86 | mole | Receptor-receptor interactions at the immunological synapse between CD28 expressed on Naive T cells and CD86 expressed on mAPCs during the first phase of priming |
| 91 | Lymph_Node | POS_Sig_NT_CD80 | mole | Receptor-receptor interactions at the immunological synapse between CD28 expressed on Naive T cells and CD86 expressed on mAPCs during the first phase of priming |
| 92 | Lymph_Node | TregLN_Secrete | cell | T Regulatory cells in the lymph node that are accounting for the constitutive secretion of CTLA-4 |
| 93 | Lymph_Node | TrLN_CTLA4S | mole | CTLA-4 that is constitutively secreted by T Regulatory cells in the lymph node |
| 94 | Lymph_Node | mAPC_nInt_CD80 | mole | CD80 receptors expressed by non-interacting mAPCs in the lymph nodes |
| 95 | Lymph_Node | mAPC_nInt_CD86 | mole | CD86 receptors expressed by non-interacting mAPCs in the lymph nodes |
| 96 | Lymph_Node | CTLA4S_CD80 | mole | Receptor-antibody interactions between CD80 expressed on non-interacting mACPs in the lymph node and CTLA-4 secreted by TT Regulatory cells |
| 97 | Lymph_Node | CTLA4S_CD86 | mole | Receptor-antibody interactions between CD86 expressed on non-interacting mACPs in the lymph node and CTLA-4 secreted by TT Regulatory cells |
| 98 | Lymph_Node | TrLN_CTLA4S_aCTLA4 | mole | Receptor-antibody interactions between CTLA-4 expressed on non-interacting mACPs in the lymph node and Anti-CTLA-4 mAb delivered to the lymph node by way of IV injection into the blood |
| 99 | Peripheral | CTLA4_mabP | mole/L | Anti-CTLA-4 antibody in the peripheral compartment |
| 100 | Peripheral | PD1_mabP | mole/L | Anti-PD-1 antibody in the peripheral compartment |
| 101 | Peripheral | PDL1_mabP | mole/L | Anti-PD-L1 antibody in the peripheral compartment |
| 102 | Peripheral | Effector_TP | cell | Effector T cells that have migrated into the peripheral tissues from the blood/plasma |
| 103 | Peripheral | Effector_Tf | cell | Free effector T cells in the vascular space |
| 104 | Peripheral | Effector_Tb | cell | Captured effector T cells in the vascular space |
| 105 | Peripheral | Effector_Ta | cell | Arrested effector T cells in the vascular space |
| 106 | Tumor | PD1_mabT | mole/L | Anti-PD-1 antibody in the tumor compartment |
| 107 | Tumor | PDL1_mabT | mole/L | Anti-PD-L1 antibody in the tumor compartment |
| 108 | Tumor | CTLA4_mabT | mole/L | Anti-CTLA-4 antibody in the tumor compartment |
| 109 | Tumor | Effector_TT | cell | Effector T cells in the tumor |
| 110 | Tumor | Effector_Tf | cell | Free effector T cells in the vascular space |
| 111 | Tumor | Effector_Tb | cell | Captured effector T cells in the vascular space |
| 112 | Tumor | Effector_Ta | cell | Arrested effector T cells in the vascular space |
| 113 | Tumor | Cancer | cell | Cancer cells in the tumor |
| 114 | Tumor | Effector_TT_C_Eng | cell | Effector T cells that can engage with cancer cells in the tumor |
| 115 | Tumor | TC1 | cell | Cancer cells that are engaged with Effector T cells in the tumor |
| 116 | Tumor | Exhausted_TT | cell | Effector T cells that have entered a deeply exhausted state |
| 117 | Tumor | C_DebrisT | cell | Cancer debris in the tumor that has resulted from cancer death |
| 118 | Tumor | TC2 | cell | Effector T cells that are engaged with cancer cells in the tumor |
| 119 | Tumor | Monocytes | cell | Monocytes that migrate into the tumor and differentiate into APCs |
| 120 | Tumor | APC_T | cell | Antigen Presenting Cells (APCs) in the tumor that can phagocytose tumor debris (tumor antigens) and become mature APCs |
| 121 | Tumor | mAPC_T | cell | Mature Antigen Presenting Cells (mAPCs) in the tumor that resulted from APCs that have phagocytosed tumor debris |
| 122 | Tumor | Cancer1 | cell | Cancer cells that can engage with Effector T cells in the tumor |
| 123 | Tumor | T{PD1}{CD80}-{PDL1}C | cell | Effector T cells in the tumor that interact with the T3 subgroup of cancer cells expressing PD-L1 only, and other unknown factors |
| 124 | Tumor | T{PD1}-{PDL2}C | cell | Effector T cells in the tumor that interact with the T4 subgroup of cancer cells expressing PD-L2 only, and other unknown factors |
| 125 | Tumor | T{PDL1}-{PD1}C | cell | Effector T cells in the tumor that interact with the T2 subgroup of cancer cells expressing PD-1 only, and other unknown factors |
| 126 | Tumor | T{PDL1}-{CD80}C | cell | Effector T cells in the tumor that interact with the T5 subgroup of cancer cells expressing CD80 only, and other unknown factors |
| 127 | Tumor | T{PD1}{L1}{80}-{PD1}{L1}C | cell | Effector T cells in the tumor that interact with the T6 subgroup of cancer cells expressing PD-1 and PD-L1 only, and other unknown factors |
| 128 | Tumor | T{PD1}{80}-{PDL1}{PDL2}C | cell | Effector T cells in the tumor that interact with the T8 subgroup of cancer cells expressing PD-L1 and PD-L2 only, and other unknown factors |
| 129 | Tumor | T{PD1}{L1}-{PD1}{L2}C | cell | Effector T cells in the tumor that interact with the T7 subgroup of cancer cells expressing PD-1 and PD-L2 only, and other unknown factors |
| 130 | Tumor | T{PD1}{L1}{80}-{PD1}{L1}{L2}C | cell | Effector T cells in the tumor that interact with the T10 subgroup of cancer cells expressing PD-1, PD-L1 and PD-L2 only, and other unknown factors |
| 131 | Tumor | T{PDL1}-{CD80}{PD1}C | cell | Effector T cells in the tumor that interact with the T9 subgroup of cancer cells expressing CD80 and PD-1 only, and other unknown factors |
| 132 | Tumor | T{PD1}{L1}-{PDL1}{80}C | cell | Effector T cells in the tumor that interact with the T11 subgroup of cancer cells expressing PD-L1 and CD80 only, and other unknown factors |
| 133 | Tumor | T{PD1}{L1}-{PDL2}{80}C | cell | Effector T cells in the tumor that interact with the T12 subgroup of cancer cells expressing PD-L2 and CD80 only, and other unknown factors |
| 134 | Tumor | T{PD1}{L1}-{PDL1}{80}{L2}C | cell | Effector T cells in the tumor that interact with the T13 subgroup of cancer cells expressing PD-L1, CD80 and PD-L2 only, and other unknown factors |
| 135 | Tumor | T{PD1}{L1}{80}-{PD1}{80}{L1}C | cell | Effector T cells in the tumor that interact with the T14 subgroup of cancer cells expressing PD-1, CD80 and PD-L1 only, and other unknown factors |
| 136 | Tumor | T{PD1}{L1}-{PD1}{80}{PDL2}C | cell | Effector T cells in the tumor that interact with the T15 subgroup of cancer cells expressing PD-1, CD80 and PD-L2 only, and other unknown factors |
| 137 | Tumor | T{PD1}{L1}{80}-{PD1}{80}{L1}{L2}C | cell | Effector T cells in the tumor that interact with the T16 subgroup of cancer cells expressing PD-1, CD80, PD-L1 and PD-L2 only, and other unknown factors |
| 138 | Tumor | T2=PDL1 | mole | PD-L1 expressed by Effector T cells that interact with T2 subgroup of cancer cells |
| 139 | Tumor | T3a=PD1 | mole | PD-1 expressed by Effector T cells that interact with T3 subgroup of cancer cells |
| 140 | Tumor | T4=PD1 | mole | PD-1 expressed by Effector T cells that interact with T4 subgroup of cancer cells |
| 141 | Tumor | T5=PDL1 | mole | PD-L1 expressed by Effector T cells that interact with T5 subgroup of cancer cells |
| 142 | Tumor | T3b=CD80 | mole | CD80 expressed by Effector T cells that interact with T3 subgroup of cancer cells |
| 143 | Tumor | T7a=PDL1 | mole | PD-L1 expressed by Effector T cells that interact with T7 subgroup of cancer cells |
| 144 | Tumor | T10b=PD1 | mole | PD-1 expressed by Effector T cells that interact with T10 subgroup of cancer cells |
| 145 | Tumor | T7b=PD1 | mole | PD-1 expressed by Effector T cells that interact with T7 subgroup of cancer cells |
| 146 | Tumor | T6a=PDL1 | mole | PD-L1 expressed by Effector T cells that interact with T6 subgroup of cancer cells |
| 147 | Tumor | T6c=CD80 | mole | CD80 expressed by Effector T cells that interact with T6 subgroup of cancer cells |
| 148 | Tumor | T6b=PD1 | mole | PD-1 expressed by Effector T cells that interact with T6 subgroup of cancer cells |
| 149 | Tumor | T11a=PDL1 | mole | PD-L1 expressed by Effector T cells that interact with T11 subgroup of cancer cells |
| 150 | Tumor | T11b=PD1 | mole | PD-1 expressed by Effector T cells that interact with T11 subgroup of cancer cells |
| 151 | Tumor | T10c=CD80 | mole | CD80 expressed by Effector T cells that interact with T10 subgroup of cancer cells |
| 152 | Tumor | T12b=PD1 | mole | PD-1 expressed by Effector T cells that interact with T12 subgroup of cancer cells |
| 153 | Tumor | T13a=PDL1 | mole | PD-L1 expressed by Effector T cells that interact with T13 subgroup of cancer cells |
| 154 | Tumor | T12a=PDL1 | mole | PD-L1 expressed by Effector T cells that interact with T12 subgroup of cancer cells |
| 155 | Tumor | T14c=CD80 | mole | CD80 expressed by Effector T cells that interact with T14 subgroup of cancer cells |
| 156 | Tumor | T15b=PD1 | mole | PD-1 expressed by Effector T cells that interact with T15 subgroup of cancer cells |
| 157 | Tumor | T15a=PDL1 | mole | PD-L1 expressed by Effector T cells that interact with T15 subgroup of cancer cells |
| 158 | Tumor | T13b=PD1 | mole | PD-1 expressed by Effector T cells that interact with T13 subgroup of cancer cells |
| 159 | Tumor | T14b=PD1 | mole | PD-1 expressed by Effector T cells that interact with T14 subgroup of cancer cells |
| 160 | Tumor | T14a=PDL1 | mole | PD-L1 expressed by Effector T cells that interact with T14 subgroup of cancer cells |
| 161 | Tumor | T16b=PD1 | mole | PD-1 expressed by Effector T cells that interact with T16 subgroup of cancer cells |
| 162 | Tumor | T16c=CD80 | mole | CD80 expressed by Effector T cells that interact with T16 subgroup of cancer cells |
| 163 | Tumor | T16a=PDL1 | mole | PD-L1 expressed by Effector T cells that interact with T16 subgroup of cancer cells |
| 164 | Tumor | T8a=PD1 | mole | PD-1 expressed by Effector T cells that interact with T8 subgroup of cancer cells |
| 165 | Tumor | T8b=CD80 | mole | CD80 expressed by Effector T cells that interact with T8 subgroup of cancer cells |
| 166 | Tumor | T9=PDL1 | mole | PD-L1 expressed by Effector T cells that interact with T9 subgroup of cancer cells |
| 167 | Tumor | T10a=PDL1 | mole | PD-L1 expressed by Effector T cells that interact with T10 subgroup of cancer cells |
| 168 | Tumor | C10a=PD1 | mole | PD-1 expressed by the T10 subtype of cancer cells that interact with Effector T cells in the tumor |
| 169 | Tumor | C9b=CD80 | mole | CD80 expressed by the T9 subtype of cancer cells that interact with Effector T cells in the tumor |
| 170 | Tumor | C9a=PD1 | mole | PD-1 expressed by the T9 subtype of cancer cells that interact with Effector T cells in the tumor |
| 171 | Tumor | C8b=PDL2 | mole | PD-L2 expressed by the T8 subtype of cancer cells that interact with Effector T cells in the tumor |
| 172 | Tumor | C16a=PD1 | mole | PD-1 expressed by the T16 subtype of cancer cells that interact with Effector T cells in the tumor |
| 173 | Tumor | C16c=PDL2 | mole | PD-L2 expressed by the T16 subtype of cancer cells that interact with Effector T cells in the tumor |
| 174 | Tumor | C16b=PDL1 | mole | PD-L1 expressed by the T16 subtype of cancer cells that interact with Effector T cells in the tumor |
| 175 | Tumor | C14a=PD1 | mole | PD-1 expressed by the T14 subtype of cancer cells that interact with Effector T cells in the tumor |
| 176 | Tumor | C14b=PDL1 | mole | PD-L1 expressed by the T14 subtype of cancer cells that interact with Effector T cells in the tumor |
| 177 | Tumor | C13b=PDL2 | mole | PD-L2 expressed by the T13 subtype of cancer cells that interact with Effector T cells in the tumor |
| 178 | Tumor | C15a=PD1 | mole | PD-1 expressed by the T15 subtype of cancer cells that interact with Effector T cells in the tumor |
| 179 | Tumor | C15b=PDL2 | mole | PD-L2 expressed by the T15 subtype of cancer cells that interact with Effector T cells in the tumor |
| 180 | Tumor | C14c=CD80 | mole | CD80 expressed by the T14 subtype of cancer cells that interact with Effector T cells in the tumor |
| 181 | Tumor | C12a=PDL2 | mole | PD-L2 expressed by the T12 subtype of cancer cells that interact with Effector T cells in the tumor |
| 182 | Tumor | C13a=PDL1 | mole | PD-L1 expressed by the T13 subtype of cancer cells that interact with Effector T cells in the tumor |
| 183 | Tumor | C12b=CD80 | mole | CD80 expressed by the T12 subtype of cancer cells that interact with Effector T cells in the tumor |
| 184 | Tumor | C10c=PDL2 | mole | PD-L2 expressed by the T10 subtype of cancer cells that interact with Effector T cells in the tumor |
| 185 | Tumor | C11b=CD80 | mole | CD80 expressed by the T11 subtype of cancer cells that interact with Effector T cells in the tumor |
| 186 | Tumor | C11a=PDL1 | mole | PD-L1 expressed by the T11 subtype of cancer cells that interact with Effector T cells in the tumor |
| 187 | Tumor | C6b=PDL1 | mole | PD-L1 expressed by the T6 subtype of cancer cells that interact with Effector T cells in the tumor |
| 188 | Tumor | C7a=PD1 | mole | PD-1 expressed by the T7 subtype of cancer cells that interact with Effector T cells in the tumor |
| 189 | Tumor | C6a=PD1 | mole | PD-1 expressed by the T6 subtype of cancer cells that interact with Effector T cells in the tumor |
| 190 | Tumor | C8a=PDL1 | mole | PD-L1 expressed by the T8 subtype of cancer cells that interact with Effector T cells in the tumor |
| 191 | Tumor | C10b=PDL1 | mole | PD-L1 expressed by the T10 subtype of cancer cells that interact with Effector T cells in the tumor |
| 192 | Tumor | C7b=PDL2 | mole | PD-L2 expressed by the T7 subtype of cancer cells that interact with Effector T cells in the tumor |
| 193 | Tumor | C5=CD80 | mole | CD80 expressed by the T5 subtype of cancer cells that interact with Effector T cells in the tumor |
| 194 | Tumor | C4=PDL2 | mole | PD-L2 expressed by the T4 subtype of cancer cells that interact with Effector T cells in the tumor |
| 195 | Tumor | C3=PDL1 | mole | PD-L1 expressed by the T3 subtype of cancer cells that interact with Effector T cells in the tumor |
| 196 | Tumor | C2=PD1 | mole | PD-1 expressed by the T2 subtype of cancer cells that interact with Effector T cells in the tumor |
| 197 | Tumor | C{PD1} | cell | T2 subgroup of cancer cells expressing PD-1 only, and other unknown factors |
| 198 | Tumor | C{PDL1} | cell | T3 subgroup of cancer cells expressing PD-L1 only, and other unknown factors |
| 199 | Tumor | C{PDL2} | cell | T4 subgroup of cancer cells expressing PD-L2 only, and other unknown factors |
| 200 | Tumor | C{CD80} | cell | T5 subgroup of cancer cells expressing CD80 only, and other unknown factors |
| 201 | Tumor | C{PD1}{PDL1} | cell | T6 subgroup of cancer cells expressing PD-1 and PD-L1 only, and other unknown factors |
| 202 | Tumor | C{PD1}{PDL2} | cell | T7 subgroup of cancer cells expressing PD-1 and PD-L2 only, and other unknown factors |
| 203 | Tumor | C{PDL1}{PDL2} | cell | T8 subgroup of cancer cells expressing PD-L1 and PD-L2 only, and other unknown factors |
| 204 | Tumor | C{PD1}{CD80} | cell | T9 subgroup of cancer cells expressing PD-1 and CD80 only, and other unknown factors |
| 205 | Tumor | C{PD1}{PDL1}{PDL2} | cell | T10 subgroup of cancer cells expressing PD-1, PD-L1 and PD-L2 only, and other unknown factors |
| 206 | Tumor | C{PDL1}{CD80} | cell | T11 subgroup of cancer cells expressing PD-L1 and CD80 only, and other unknown factors |
| 207 | Tumor | C{PDL2}{CD80} | cell | T12 subgroup of cancer cells expressing PD-L2 and CD80 only, and other unknown factors |
| 208 | Tumor | C{PDL1}{PDL2}{CD80} | cell | T13 subgroup of cancer cells expressing PD-L1, PDL-L2 and CD80 only, and other unknown factors |
| 209 | Tumor | C{PD1}{PDL1}{CD80} | cell | T14 subgroup of cancer cells expressing PD-1, PD-L1 and CD80 only, and other unknown factors |
| 210 | Tumor | C{PD1}{PDL2}{CD80} | cell | T15 subgroup of cancer cells expressing PD-1, PD-L2 and CD80 only, and other unknown factors |
| 211 | Tumor | C{PD1}{PDL1}{PDL2}{CD80} | cell | T16 subgroup of cancer cells expressing PD-1, PD-L1, PD-L2 and CD80 only, and other unknown factors |
| 212 | Tumor | C13c=CD80 | mole | CD80 expressed by the T13 subtype of cancer cells that interact with Effector T cells in the tumor |
| 213 | Tumor | C15c=CD80 | mole | CD80 expressed by the T15 subtype of cancer cells that interact with Effector T cells in the tumor |
| 214 | Tumor | C16d=CD80 | mole | CD80 expressed by the T16 subtype of cancer cells that interact with Effector T cells in the tumor |
| 215 | Tumor | T2=PDL1:PD1=C2 | mole | Receptor-receptor interactions at the immunological synapse between PD-1 expressed by the T2 subtype of cancer cells and PD-L1 expressed on Effector T cells |
| 216 | Tumor | T3a=PD1:PDL1=C3 | mole | Receptor-receptor interactions at the immunological synapse between PD-L1 expressed by the T3 subtype of cancer cells and PD-1 expressed on Effector T cells |
| 217 | Tumor | T3b=CD80:PDL1=C3 | mole | Receptor-receptor interactions at the immunological synapse between PD-L1 expressed by the T3 subtype of cancer cells and CD80 expressed on Effector T cells |
| 218 | Tumor | T4=PD1:PDL2=C4 | mole | Receptor-receptor interactions at the immunological synapse between PD-L2 expressed by the T4 subtype of cancer cells and PD-1 expressed on Effector T cells |
| 219 | Tumor | T5=PDL1:CD80=C5 | mole | Receptor-receptor interactions at the immunological synapse between CD80 expressed by the T5 subtype of cancer cells and PD-L1 expressed on Effector T cells |
| 220 | Tumor | T6a=PDL1:PD1=C6a | mole | Receptor-receptor interactions at the immunological synapse between PD-1 expressed by the T6 subtype of cancer cells and PD-L1 expressed on Effector T cells |
| 221 | Tumor | T6c=CD80:PDL1=C6b | mole | Receptor-receptor interactions at the immunological synapse between PD-L1 expressed by the T6 subtype of cancer cells and CD80 expressed on Effector T cells |
| 222 | Tumor | T6b=PD1:PDL1=C6b | mole | Receptor-receptor interactions at the immunological synapse between PD-L1 expressed by the T6 subtype of cancer cells and PD-1 expressed on Effector T cells |
| 223 | Tumor | T7a=PDL1:PD1=C7a | mole | Receptor-receptor interactions at the immunological synapse between PD-1 expressed by the T7 subtype of cancer cells and PD-L1 expressed on Effector T cells |
| 224 | Tumor | T7b=PD1:PDL2=C7b | mole | Receptor-receptor interactions at the immunological synapse between PD-L2 expressed by the T7 subtype of cancer cells and PD-1 expressed on Effector T cells |
| 225 | Tumor | T8a=PD1:PDL2=C8b | mole | Receptor-receptor interactions at the immunological synapse between PD-L2 expressed by the T8 subtype of cancer cells and PD-1 expressed on Effector T cells |
| 226 | Tumor | T8a=PD1:PDL1=C8a | mole | Receptor-receptor interactions at the immunological synapse between PD-L1 expressed by the T8 subtype of cancer cells and PD-1 expressed on Effector T cells |
| 227 | Tumor | T8b=CD80:PDL1=C8a | mole | Receptor-receptor interactions at the immunological synapse between PD-L1 expressed by the T8 subtype of cancer cells and CD80 expressed on Effector T cells |
| 228 | Tumor | T9=PDL1-PD1=C9a | mole | Receptor-receptor interactions at the immunological synapse between PD-1 expressed by the T9 subtype of cancer cells and PD-L1 expressed on Effector T cells |
| 229 | Tumor | T9=PDL1-CD80=C9b | mole | Receptor-receptor interactions at the immunological synapse between CD80 expressed by the T9 subtype of cancer cells and PD-L1 expressed on Effector T cells |
| 230 | Tumor | T10a=PDL1:PD1=C10a | mole | Receptor-receptor interactions at the immunological synapse between PD-1 expressed by the T10 subtype of cancer cells and PD-L1 expressed on Effector T cells |
| 231 | Tumor | T10b=PD1:PDL1=C10b | mole | Receptor-receptor interactions at the immunological synapse between PD-L1 expressed by the T10 subtype of cancer cells and PD-1 expressed on Effector T cells |
| 232 | Tumor | T10b=PD1:PDL2=C10c | mole | Receptor-receptor interactions at the immunological synapse between PD-L2 expressed by the T10 subtype of cancer cells and PD-1 expressed on Effector T cells |
| 233 | Tumor | T10c=CD80:PDL1=C10b | mole | Receptor-receptor interactions at the immunological synapse between PD-L1 expressed by the T10 subtype of cancer cells and CD80 expressed on Effector T cells |
| 234 | Tumor | T11b=PD1:PDL1=C11a | mole | Receptor-receptor interactions at the immunological synapse between PD-L1 expressed by the T11 subtype of cancer cells and PD-1 expressed on Effector T cells |
| 235 | Tumor | T11a=PDL1:CD80=C11b | mole | Receptor-receptor interactions at the immunological synapse between CD80 expressed by the T11 subtype of cancer cells and PD-L1 expressed on Effector T cells |
| 236 | Tumor | T12b=PD1:PDL2=C12a | mole | Receptor-receptor interactions at the immunological synapse between PD-L2 expressed by the T12 subtype of cancer cells and PD-1 expressed on Effector T cells |
| 237 | Tumor | T12a=PDL1:CD80=C12b | mole | Receptor-receptor interactions at the immunological synapse between CD80 expressed by the T12 subtype of cancer cells and PD-L1 expressed on Effector T cells |
| 238 | Tumor | T13b=PD1:PDL1=C13a | mole | Receptor-receptor interactions at the immunological synapse between PD-L1 expressed by the T13 subtype of cancer cells and PD-1 expressed on Effector T cells |
| 239 | Tumor | T13a=PDL1:CD80=C13c | mole | Receptor-receptor interactions at the immunological synapse between CD80 expressed by the T13 subtype of cancer cells and PD-L1 expressed on Effector T cells |
| 240 | Tumor | T13b=PD1:PDL2=C13b | mole | Receptor-receptor interactions at the immunological synapse between PD-L2 expressed by the T13 subtype of cancer cells and PD-1 expressed on Effector T cells |
| 241 | Tumor | T14a=PDL1:PD1=C14a | mole | Receptor-receptor interactions at the immunological synapse between PD-1 expressed by the T14 subtype of cancer cells and PD-L1 expressed on Effector T cells |
| 242 | Tumor | T14a=PDL1:CD80=C14c | mole | Receptor-receptor interactions at the immunological synapse between CD80 expressed by the T14 subtype of cancer cells and PD-L1 expressed on Effector T cells |
| 243 | Tumor | T14b=PD1:PDL1=C14b | mole | Receptor-receptor interactions at the immunological synapse between PD-L1 expressed by the T14 subtype of cancer cells and PD-1 expressed on Effector T cells |
| 244 | Tumor | T14c=CD80:PDL1=C14b | mole | Receptor-receptor interactions at the immunological synapse between CD80 expressed by the T14 subtype of cancer cells and PD-L1 expressed on Effector T cells |
| 245 | Tumor | T15a=PDL1:CD80=C15c | mole | Receptor-receptor interactions at the immunological synapse between CD80 expressed by the T15 subtype of cancer cells and PD-L1 expressed on Effector T cells |
| 246 | Tumor | T15a=PDL1:PD1=C15a | mole | Receptor-receptor interactions at the immunological synapse between PD-1 expressed by the T15 subtype of cancer cells and PD-L1 expressed on Effector T cells |
| 247 | Tumor | T15b=PD1:PDL2=C15b | mole | Receptor-receptor interactions at the immunological synapse between PD-L2 expressed by the T15 subtype of cancer cells and PD-1 expressed on Effector T cells |
| 248 | Tumor | T16b=PD1:PDL2=C16c | mole | Receptor-receptor interactions at the immunological synapse between PD-L2 expressed by the T15 subtype of cancer cells and PD-1 expressed on Effector T cells |
| 249 | Tumor | T16b=PD1:PDL1=C16b | mole | Receptor-receptor interactions at the immunological synapse between PD-L1 expressed by the T15 subtype of cancer cells and PD-1 expressed on Effector T cells |
| 250 | Tumor | T16c=CD80:PDL1=C16b | mole | Receptor-receptor interactions at the immunological synapse between PD-L1 expressed by the T15 subtype of cancer cells and CD80 expressed on Effector T cells |
| 251 | Tumor | T16a=PDL1:PD1=C16a | mole | Receptor-receptor interactions at the immunological synapse between PD-1 expressed by the T15 subtype of cancer cells and PD-L1 expressed on Effector T cells |
| 252 | Tumor | T16a=PDL1:CD80=C16d | mole | Receptor-receptor interactions at the immunological synapse between CD80 expressed by the T15 subtype of cancer cells and PD-L1 expressed on Effector T cells |
| 253 | Tumor | T2=PDL1:aPDL1 | mole | Heterogeneous receptor-antibody interactions at the immunological synapse between PD-L1 expressed by Effector T cells that interact with the T2 subtype of cancer cells and Anti-PD-L1 mAb delivered to the tumor by way of IV injection into the blood |
| 254 | Tumor | T3a=PD1:aPD1 | mole | Heterogeneous receptor-antibody interactions at the immunological synapse between PD-1 expressed by Effector T cells that interact with the T3 subtype of cancer cells and Anti-PD-L1 mAb delivered to the tumor by way of IV injection into the blood |
| 255 | Tumor | C3=PDL1:aPDL1 | mole | Heterogeneous receptor-antibody interactions at the immunological synapse between PD-L1 expressed by the T3 subtype of cancer cells and Anti-PD-L1 mAb delivered to the tumor by way of IV injection into the blood |
| 256 | Tumor | T5=PDL1:aPDL1 | mole | Heterogeneous receptor-antibody interactions at the immunological synapse between PD-L1 expressed by Effector T cells that interact with the T5 subtype of cancer cells and Anti-PD-L1 mAb delivered to the tumor by way of IV injection into the blood |
| 257 | Tumor | T6a=PDL1:aPDL1 | mole | Heterogeneous receptor-antibody interactions at the immunological synapse between PD-L1 expressed by Effector T cells that interact with the T6 subtype of cancer cells and Anti-PD-L1 mAb delivered to the tumor by way of IV injection into the blood |
| 258 | Tumor | C6b=PDL1:aPDL1 | mole | Heterogeneous receptor-antibody interactions at the immunological synapse between PD-L1 expressed by the T6 subtype of cancer cells and Anti-PD-L1 mAb delivered to the tumor by way of IV injection into the blood |
| 259 | Tumor | T10a=PDL1:aPDL1 | mole | Heterogeneous receptor-antibody interactions at the immunological synapse between PD-L1 expressed by Effector T cells that interact with the T10 subtype of cancer cells and Anti-PD-L1 mAb delivered to the tumor by way of IV injection into the blood |
| 260 | Tumor | C10b=PDL1:aPDL1 | mole | Heterogeneous receptor-antibody interactions at the immunological synapse between PD-L1 expressed by the T10 subtype of cancer cells and Anti-PD-L1 mAb delivered to the tumor by way of IV injection into the blood |
| 261 | Tumor | C11a=PDL1:aPDL1 | mole | Heterogeneous receptor-antibody interactions at the immunological synapse between PD-L1 expressed by the T11 subtype of cancer cells and Anti-PD-L1 mAb delivered to the tumor by way of IV injection into the blood |
| 262 | Tumor | T11a=PDL1:aPDL1 | mole | Heterogeneous receptor-antibody interactions at the immunological synapse between PD-L1 expressed by Effector T cells that interact with the T11 subtype of cancer cells and Anti-PD-L1 mAb delivered to the tumor by way of IV injection into the blood |
| 263 | Tumor | T12a=PDL1:aPDL1 | mole | Heterogeneous receptor-antibody interactions at the immunological synapse between PD-L1 expressed by Effector T cells that interact with the T12 subtype of cancer cells and Anti-PD-L1 mAb delivered to the tumor by way of IV injection into the blood |
| 264 | Tumor | T7a=PDL1:aPDL1 | mole | Heterogeneous receptor-antibody interactions at the immunological synapse between PD-L1 expressed by Effector T cells that interact with the T7 subtype of cancer cells and Anti-PD-L1 mAb delivered to the tumor by way of IV injection into the blood |
| 265 | Tumor | C8a=PDL1:aPDL1 | mole | Heterogeneous receptor-antibody interactions at the immunological synapse between PD-L1 expressed by the T8 subtype of cancer cells and Anti-PD-L1 mAb delivered to the tumor by way of IV injection into the blood |
| 266 | Tumor | T9=PDL1:aPDL1 | mole | Heterogeneous receptor-antibody interactions at the immunological synapse between PD-L1 expressed by Effector T cells that interact with the T9 subtype of cancer cells and Anti-PD-L1 mAb delivered to the tumor by way of IV injection into the blood |
| 267 | Tumor | C13a=PDL1:aPDL1 | mole | Heterogeneous receptor-antibody interactions at the immunological synapse between PD-L1 expressed by the T13 subtype of cancer cells and Anti-PD-L1 mAb delivered to the tumor by way of IV injection into the blood |
| 268 | Tumor | T13a=PDL1:aPDL1 | mole | Heterogeneous receptor-antibody interactions at the immunological synapse between PD-L1 expressed by Effector T cells that interact with the T13 subtype of cancer cells and Anti-PD-L1 mAb delivered to the tumor by way of IV injection into the blood |
| 269 | Tumor | T14a=PDL1:aPDL1 | mole | Heterogeneous receptor-antibody interactions at the immunological synapse between PD-L1 expressed by Effector T cells that interact with the T14 subtype of cancer cells and Anti-PD-L1 mAb delivered to the tumor by way of IV injection into the blood |
| 270 | Tumor | C14b=PDL1:aPDL1 | mole | Heterogeneous receptor-antibody interactions at the immunological synapse between PD-L1 expressed by the T14 subtype of cancer cells and Anti-PD-L1 mAb delivered to the tumor by way of IV injection into the blood |
| 271 | Tumor | T15a=PDL1:aPDL1 | mole | Heterogeneous receptor-antibody interactions at the immunological synapse between PD-L1 expressed by Effector T cells that interact with the T15 subtype of cancer cells and Anti-PD-L1 mAb delivered to the tumor by way of IV injection into the blood |
| 272 | Tumor | T16a=PDL1:aPDL1 | mole | Heterogeneous receptor-antibody interactions at the immunological synapse between PD-L1 expressed by Effector T cells that interact with the T16 subtype of cancer cells and Anti-PD-L1 mAb delivered to the tumor by way of IV injection into the blood |
| 273 | Tumor | C16b=PDL1:aPDL1 | mole | Heterogeneous receptor-antibody interactions at the immunological synapse between PD-L1 expressed by the T16 subtype of cancer cells and Anti-PD-L1 mAb delivered to the tumor by way of IV injection into the blood |
| 274 | Tumor | C2=PD1:aPD1 | mole | Heterogeneous receptor-antibody interactions at the immunological synapse between PD-1 expressed by the T2 subtype of cancer cells and Anti-PD-1 mAb delivered to the tumor by way of IV injection into the blood |
| 275 | Tumor | T4=PD1:aPD1 | mole | Heterogeneous receptor-antibody interactions at the immunological synapse between PD-1 expressed by Effector T cells that interact with the T4 subtype of cancer cells and Anti-PD-L1 mAb delivered to the tumor by way of IV injection into the blood |
| 276 | Tumor | C6a=PD1:aPD1 | mole | Heterogeneous receptor-antibody interactions at the immunological synapse between PD-1 expressed by the T6 subtype of cancer cells and Anti-PD-1 mAb delivered to the tumor by way of IV injection into the blood |
| 277 | Tumor | T6b=PD1:aPD1 | mole | Heterogeneous receptor-antibody interactions at the immunological synapse between PD-1 expressed by Effector T cells that interact with the T6 subtype of cancer cells and Anti-PD-L1 mAb delivered to the tumor by way of IV injection into the blood |
| 278 | Tumor | C7a=PD1:aPD1 | mole | Heterogeneous receptor-antibody interactions at the immunological synapse between PD-1 expressed by the T7 subtype of cancer cells and Anti-PD-1 mAb delivered to the tumor by way of IV injection into the blood |
| 279 | Tumor | T7b=PD1:aPD1 | mole | Heterogeneous receptor-antibody interactions at the immunological synapse between PD-1 expressed by Effector T cells that interact with the T7 subtype of cancer cells and Anti-PD-L1 mAb delivered to the tumor by way of IV injection into the blood |
| 280 | Tumor | T8a=PD1:aPD1 | mole | Heterogeneous receptor-antibody interactions at the immunological synapse between PD-1 expressed by Effector T cells that interact with the T8 subtype of cancer cells and Anti-PD-L1 mAb delivered to the tumor by way of IV injection into the blood |
| 281 | Tumor | C9a=PD1:aPD1 | mole | Heterogeneous receptor-antibody interactions at the immunological synapse between PD-1 expressed by the T9 subtype of cancer cells and Anti-PD-1 mAb delivered to the tumor by way of IV injection into the blood |
| 282 | Tumor | C10a=PD1:aPD1 | mole | Heterogeneous receptor-antibody interactions at the immunological synapse between PD-1 expressed by the T10 subtype of cancer cells and Anti-PD-1 mAb delivered to the tumor by way of IV injection into the blood |
| 283 | Tumor | T10b=PD1:aPD1 | mole | Heterogeneous receptor-antibody interactions at the immunological synapse between PD-1 expressed by Effector T cells that interact with the T10 subtype of cancer cells and Anti-PD-L1 mAb delivered to the tumor by way of IV injection into the blood |
| 284 | Tumor | T11b=PD1:aPD1 | mole | Heterogeneous receptor-antibody interactions at the immunological synapse between PD-1 expressed by Effector T cells that interact with the T11 subtype of cancer cells and Anti-PD-L1 mAb delivered to the tumor by way of IV injection into the blood |
| 285 | Tumor | T13b=PD1:aPD1 | mole | Heterogeneous receptor-antibody interactions at the immunological synapse between PD-1 expressed by Effector T cells that interact with the T13 subtype of cancer cells and Anti-PD-L1 mAb delivered to the tumor by way of IV injection into the blood |
| 286 | Tumor | C14a=PD1:aPD1 | mole | Heterogeneous receptor-antibody interactions at the immunological synapse between PD-1 expressed by the T14 subtype of cancer cells and Anti-PD-1 mAb delivered to the tumor by way of IV injection into the blood |
| 287 | Tumor | T14b=PD1:aPD1 | mole | Heterogeneous receptor-antibody interactions at the immunological synapse between PD-1 expressed by Effector T cells that interact with the T14 subtype of cancer cells and Anti-PD-L1 mAb delivered to the tumor by way of IV injection into the blood |
| 288 | Tumor | C15a=PD1:aPD1 | mole | Heterogeneous receptor-antibody interactions at the immunological synapse between PD-1 expressed by the T15 subtype of cancer cells and Anti-PD-1 mAb delivered to the tumor by way of IV injection into the blood |
| 289 | Tumor | T15b=PD1:aPD1 | mole | Heterogeneous receptor-antibody interactions at the immunological synapse between PD-1 expressed by Effector T cells that interact with the T15 subtype of cancer cells and Anti-PD-L1 mAb delivered to the tumor by way of IV injection into the blood |
| 290 | Tumor | C16a=PD1:aPD1 | mole | Heterogeneous receptor-antibody interactions at the immunological synapse between PD-1 expressed by the T16 subtype of cancer cells and Anti-PD-1 mAb delivered to the tumor by way of IV injection into the blood |
| 291 | Tumor | T16b=PD1:aPD1 | mole | Heterogeneous receptor-antibody interactions at the immunological synapse between PD-1 expressed by Effector T cells that interact with the T16 subtype of cancer cells and Anti-PD-L1 mAb delivered to the tumor by way of IV injection into the blood |
| 292 | Tumor | T12b=PD1:aPD1 | mole | Heterogeneous receptor-antibody interactions at the immunological synapse between PD-1 expressed by Effector T cells that interact with the T12 subtype of cancer cells and Anti-PD-L1 mAb delivered to the tumor by way of IV injection into the blood |
| 293 | Tumor | TregT | cell | Total number of T Regulatory cells that able to engage with Effector T cells and mAPCs in the tumor |
| 294 | Tumor | TregT_Teff | cell | T Regulatory cells in the tumor that have engaged in interacting with Effector T cells |
| 295 | Tumor | TregT_mAPCT | cell | T Regulatory cells in the tumor that have engaged in interacting with mAPCs |
| 296 | Tumor | MDSC_T | cell | Total number of MDSCs that able to engage with Effector T cells in the tumor |
| 297 | Tumor | MDSCsT_Teff | cell | MDSCs in the tumor that have engaged in interacting with Effector T cells |
| 298 | Tumor | MDSCsT_EngTeff | cell | MDSCs in the tumor that have engaged in interacting with Effector T cells that are accounting for the expression of PD-1 and PD-L1 |
| 299 | Tumor | TregT_EngAPC | cell | T Regulatory cells in the tumor that have engaged in interacting with mAPCs that are accounting for the expression of CTLA-4 |
| 300 | Tumor | mAPCT_EngTregT | cell | mAPCs in the tumor that have engaged in interacting with T Regulatory cells that are accounting for the expression of CD80 and CD86 |
| 301 | Tumor | Teff_EngTregT | cell | Effector T cells in the tumor that have engaged in interacting with T Regulatory cells that are accounting for the expression of CD80, PD-1 and PD-L1 |
| 302 | Tumor | PD1_TeffT | mole | PD-1 expressed by Effector T cells that interact with T Regulatory cells in the tumor |
| 303 | Tumor | PDL1_TeffT | mole | PD-L1 expressed by Effector T cells that interact with T Regulatory cells in the tumor |
| 304 | Tumor | CD80_TeffT | mole | CD80 expressed by Effector T cells that interact with T Regulatory cells in the tumor |
| 305 | Tumor | CD80_mAPCT | mole | CD80 expressed by mAPCs that interact with T Regulatory cells in the tumor |
| 306 | Tumor | PDL1_MDSCsT | mole | PD-L1 expressed by MDSCs that interact with Effector T cells in the tumor |
| 307 | Tumor | PD1_MDSCsT | mole | PD-1 expressed by MDSCs that interact with Effector T cells in the tumor |
| 308 | Tumor | CD86_mAPCT | mole | CD86 expressed by mAPCs that interact with T Regulatory cells in the tumor |
| 309 | Tumor | PD1_TregT | mole | PD-1 expressed by T Regulatory cells that interact with Effector T cells in the tumor |
| 310 | Tumor | PDL1_TregT | mole | PD-L1 expressed by T Regulatory cells that interact with Effector T cells in the tumor |
| 311 | Tumor | CTLA4_TregT | mole | CTLA-4 expressed by T Regulatory cells that interact with mAPCs in the tumor |
| 312 | Tumor | CTLA4:CD80_TrAT | mole | Receptor-receptor interactions at the immunological synapse between CD80 expressed by mAPCs and CTLA-4 expressed on T Regulatory cells in the tumor |
| 313 | Tumor | CTLA4:CD86_TrAT | mole | Receptor-receptor interactions at the immunological synapse between CD86 expressed by mAPCs and CTLA-4 expressed on T Regulatory cells in the tumor |
| 314 | Tumor | CTLA4_CTLA4-Trt | mole | Heterogeneous receptor-antibody interactions at the immunological synapse between CTLA-4 expressed by T Regulatory cells that interact with the mAPCs and Anti-PD-L1 mAb delivered to the tumor by way of IV injection into the blood |
| 315 | Tumor | PDL1:PD1_TrTeff | mole | Receptor-receptor interactions at the immunological synapse between PD-1 expressed by the Effector T cells and PD-L1 expressed on T Regulatory cells in the tumor |
| 316 | Tumor | PDL1:CD80_TrTeff | mole | Receptor-receptor interactions at the immunological synapse between CD80 expressed by the Effector T cells and PD-L1 expressed on T Regulatory cells in the tumor |
| 317 | Tumor | PD1:PDL1_TrTeff | mole | Receptor-receptor interactions at the immunological synapse between PD-L1 expressed by the Effector T cells and PD-1 expressed on T Regulatory cells in the tumor |
| 318 | Tumor | PDL1:CD80_MDSCT | mole | Receptor-receptor interactions at the immunological synapse between CD80 expressed by the Effector T cells and PD-L1 expressed on MDSCs in the tumor |
| 319 | Tumor | PDL1:PD1_MDSCT | mole | Receptor-receptor interactions at the immunological synapse between PD-1 expressed by the Effector T cells and PD-L1 expressed on MDSCs cells in the tumor |
| 320 | Tumor | PD1:PDL1_MDSCT | mole | Receptor-receptor interactions at the immunological synapse between PD-L1 expressed by the Effector T cells and PD-1 expressed on MDSCs in the tumor |
| 321 | Tumor | PD1:aPD1_Teff | mole | Heterogeneous receptor-antibody interactions at the immunological synapse between PD-1 expressed by Effector T cells that interact with the T Regulatory cells and Anti-PD-1 mAb delivered to the tumor by way of IV injection into the blood |
| 322 | Tumor | PDL1:aPDL1_Teff | mole | Heterogeneous receptor-antibody interactions at the immunological synapse between PD-L1 expressed by Effector T cells that interact with the T Regulatory cells and Anti-PD-L1 mAb delivered to the tumor by way of IV injection into the blood |
| 323 | Tumor | PDL1:aPDL1_Treg | mole | Heterogeneous receptor-antibody interactions at the immunological synapse between PD-L1 expressed by T Regulatory cells that interact with Effector T cells and Anti-PD-L1 mAb delivered to the tumor by way of IV injection into the blood |
| 324 | Tumor | PD1:aPD1_Treg | mole | Heterogeneous receptor-antibody interactions at the immunological synapse between PD-1 expressed by T Regulatory cells that interact with Effector T cells and Anti-PD-1 mAb delivered to the tumor by way of IV injection into the blood |
| 325 | Tumor | PD1:aPD1_MDSCs | mole | Heterogeneous receptor-antibody interactions at the immunological synapse between PD-1 expressed by MDSCs that interact with Effector T cells and Anti-PD-1 mAb delivered to the tumor by way of IV injection into the blood |
| 326 | Tumor | PDL1:aPDL1_MDSCs | mole | Heterogeneous receptor-antibody interactions at the immunological synapse between PD-L1 expressed by MDSCs that interact with Effector T cells and Anti-PD-L1 mAb delivered to the tumor by way of IV injection into the blood |
| 327 | Tumor | TregT_EngTeff | cell | T Regulatory cells in the tumor that have engaged in interacting with Effector T cells that are accounting for the expression of PD-1 and PD-L1 |
| 328 | Tumor | Teff_EngMDSC | cell | Effector T cells in the tumor that have engaged in interacting with MDSCs that are accounting for the expression of CD80, PD-1 and PD-L1 |
| 329 | Tumor | CD80_TeffT1 | mole | CD80 expressed by Effector T cells that interact with MDSCs in the tumor |
| 330 | Tumor | PDL1_TeffT1 | mole | PD-L1 expressed by Effector T cells that interact with MDSCs in the tumor |
| 331 | Tumor | PD1_TeffT1 | mole | PD-1 expressed by Effector T cells that interact with MDSCs in the tumor |
| 332 | Tumor | PD1:aPD1_Teff1 | mole | Heterogeneous receptor-antibody interactions at the immunological synapse between PD-1 expressed by Effector T cells that interact with the MDSCs and Anti-PD-1 mAb delivered to the tumor by way of IV injection into the blood |
| 333 | Tumor | PDL1:aPDL1_Teff1 | mole | Heterogeneous receptor-antibody interactions at the immunological synapse between PD-L1 expressed by Effector T cells that interact with the MDSCs and Anti-PD-L1 mAb delivered to the tumor by way of IV injection into the blood |
| 334 | Tumor | TregT1 | cell | Non-interacting T Regulatory cells in the tumor that account for the constitutive expression of CTLA-4 to their surface |
| 335 | Tumor | CTLA4_TregTS | mole | CTLA-4 constitutively expressed to the surface of T Regulatory cells in the tumor |
| 336 | Tumor | CTLA4_aCTLA4-TrTS | mole | Receptor-antibody interactions between CTLA-4 expressed by T Regulatory cells in the tumor and Anti-CTLA-4 mAb delivered to the tumor by way of IV injection into the blood |
| 337 | Tumor | Effector_TT_TregT | cell | Total number of Effector T cells that able to engage with T Regulatory cells in the tumor |
| 338 | Tumor | Effector_TT_MDSCs | cell | Total number of Effector T cells that able to engage with MDSCs in the tumor |
| 339 | Tumor | mAPC_T_TregT | cell | Total number of mAPCs that able to engage with T Regulatory cells in the tumor |
| 340 | Tumor | Effector_TT_Count | cell | The total number of Effector and Resident Effector Memory cells |
| 341 | Tumor | Effector_TT_per_Treg | cell/cell | The number of Effector T cells per T Regulatory cells in the tumor |
| 342 | Tumor | Effector_TT_per_mm3 | cell/mm^3^ | The volumetric density of Effector T cells per cubic millimeter in the tumor |
| 343 | Tumor | Ratio_T/cm3_per_max | cell/cm^3^ | The volumetric density of Effector T cells per maximum density physically possible |

**Table S3 – Definition of species in the model (End)**

**Table S4 – Model Reactions (Start)**

| Reaction Number | Reaction |
| --- | --- |
| 1 | [Blood-Lymph].CTLA4_mabB -> null |
| 2 | [Blood-Lymph].CTLA4_mabB -> null |
| 3 | [Blood-Lymph].CTLA4_mabB <-> Peripheral.CTLA4_mabP |
| 4 | [Blood-Lymph].CTLA4_mabB <-> Tumor.CTLA4_mabT |
| 5 | [Blood-Lymph].Effector_T_TB -> [Blood-Lymph].Effector_T_TB + [Blood-Lymph].Effector_TB |
| 6 | [Blood-Lymph].Effector_TB <-> Peripheral.Effector_Tf |
| 7 | [Blood-Lymph].Effector_TB <-> Tumor.Effector_Tf |
| 8 | [Blood-Lymph].PD1_mabB -> null |
| 9 | [Blood-Lymph].PD1_mabB -> null |
| 10 | [Blood-Lymph].PD1_mabB <-> Peripheral.PD1_mabP |
| 11 | [Blood-Lymph].PD1_mabB <-> Tumor.PD1_mabT |
| 12 | [Blood-Lymph].PDL1_mabB -> null |
| 13 | [Blood-Lymph].PDL1_mabB -> null |
| 14 | [Blood-Lymph].PDL1_mabB <-> Peripheral.PDL1_mabP |
| 15 | [Blood-Lymph].PDL1_mabB <-> Tumor.PDL1_mabT |
| 16 | Lymph_Node.[PNT-Tr_CD80] -> null |
| 17 | Lymph_Node.[PNT-Tr_CD80] + Lymph_Node.[Tr-PNT_PDL1] <-> Lymph_Node.[TrPNT_PDL1-CD80] |
| 18 | Lymph_Node.[PNT-Tr_PD1] -> null |
| 19 | Lymph_Node.[PNT-Tr_PD1] + Lymph_Node.[Tr-PNT_PDL1] <-> Lymph_Node.[TrPNT_PDL1-PD1] |
| 20 | Lymph_Node.[PNT-Tr_PD1] + Lymph_Node.PD1_mab <-> Lymph_Node.PNTTr_PD1_aPD1 |
| 21 | Lymph_Node.[PNT-Tr_PDL1] -> null |
| 22 | Lymph_Node.[PNT-Tr_PDL1] + Lymph_Node.PDL1_mab <-> Lymph_Node.PNTTr_PDL1_aPDL1 |
| 23 | Lymph_Node.[Tr-mAPC_CD80] -> null |
| 24 | Lymph_Node.[Tr-mAPC_CD86] -> null |
| 25 | Lymph_Node.[Tr-mAPC_CTLA4] -> null |
| 26 | Lymph_Node.[Tr-mAPC_CTLA4] + Lymph_Node.[Tr-mAPC_CD80] <-> Lymph_Node.TrALN_CT_CD80 |
| 27 | Lymph_Node.[Tr-mAPC_CTLA4] + Lymph_Node.[Tr-mAPC_CD86] <-> Lymph_Node.TrALN_CT_CD86 |
| 28 | Lymph_Node.[Tr-mAPC_CTLA4] + Lymph_Node.CTLA4_mab <-> Lymph_Node.TrALN_CT_aCT |
| 29 | Lymph_Node.[Tr-PNT_PD1] -> null |
| 30 | Lymph_Node.[Tr-PNT_PD1] + Lymph_Node.[PNT-Tr_PDL1] <-> Lymph_Node.[TrPNT_PD1-L1] |
| 31 | Lymph_Node.[Tr-PNT_PD1] + Lymph_Node.PD1_mab <-> Lymph_Node.TrPNT_PD1_aPD1 |
| 32 | Lymph_Node.[Tr-PNT_PDL1] -> null |
| 33 | Lymph_Node.[Tr-PNT_PDL1] + Lymph_Node.PDL1_mab <-> Lymph_Node.TrPNT_PDL1_aPDL1 |
| 34 | Lymph_Node.[TregLN-NT] -> null |
| 35 | Lymph_Node.[TregLN-NT] + Lymph_Node.Naive_T -> null |
| 36 | Lymph_Node.[TregLN-PNT] -> null |
| 37 | Lymph_Node.[TregLN-PNT] + Lymph_Node.Primed_Naive_T -> null |
| 38 | Lymph_Node.[TregLN-PNT1] -> Lymph_Node.[PNT-Tr_CD80] + Lymph_Node.[TregLN-PNT1] |
| 39 | Lymph_Node.[TregLN-PNT1] -> Lymph_Node.[PNT-Tr_PD1] + Lymph_Node.[TregLN-PNT1] |
| 40 | Lymph_Node.[TregLN-PNT1] -> Lymph_Node.[PNT-Tr_PDL1] + Lymph_Node.[TregLN-PNT1] |
| 41 | Lymph_Node.[TregLN-PNT1] -> Lymph_Node.[Tr-PNT_PD1] + Lymph_Node.[TregLN-PNT1] |
| 42 | Lymph_Node.[TregLN-PNT1] -> Lymph_Node.[Tr-PNT_PDL1] + Lymph_Node.[TregLN-PNT1] |
| 43 | Lymph_Node.APCLN + Lymph_Node.C_DebrisLN -> Lymph_Node.mAPC |
| 44 | Lymph_Node.C_DebrisLN -> null |
| 45 | Lymph_Node.CTLA4_mab -> null |
| 46 | Lymph_Node.CTLA4_mab + Lymph_Node.PNT_CTLA4 <-> Lymph_Node.CTLA4_mAb_CTLA4 |
| 47 | Lymph_Node.CTLA4_mAb_CTLA4 -> null |
| 48 | Lymph_Node.Effector_T -> null |
| 49 | Lymph_Node.mAPC -> Lymph_Node.mAPC_CD80 + Lymph_Node.mAPC |
| 50 | Lymph_Node.mAPC -> Lymph_Node.mAPC_CD86 + Lymph_Node.mAPC |
| 51 | Lymph_Node.mAPC -> Lymph_Node.mAPC_PD1 + Lymph_Node.mAPC |
| 52 | Lymph_Node.mAPC -> Lymph_Node.mAPC_PDL1 + Lymph_Node.mAPC |
| 53 | Lymph_Node.mAPC -> Lymph_Node.mAPC_PDL2 + Lymph_Node.mAPC |
| 54 | Lymph_Node.mAPC -> null |
| 55 | Lymph_Node.mAPC -> null |
| 56 | Lymph_Node.mAPC_CD80 -> null |
| 57 | Lymph_Node.mAPC_CD86 -> null |
| 58 | Lymph_Node.mAPC_Int_P1 -> Lymph_Node.mAPC_Int_P1 + Lymph_Node.mAPC_Int_P1_CD80 |
| 59 | Lymph_Node.mAPC_Int_P1 -> Lymph_Node.mAPC_Int_P1 + Lymph_Node.mAPC_Int_P1_CD86 |
| 60 | Lymph_Node.mAPC_Int_P1_CD80 -> null |
| 61 | Lymph_Node.mAPC_Int_P1_CD86 -> null |
| 62 | Lymph_Node.mAPC_Int_P2 -> Lymph_Node.mAPC_Int_P2_CD80 + Lymph_Node.mAPC_Int_P2 |
| 63 | Lymph_Node.mAPC_Int_P2 -> Lymph_Node.mAPC_Int_P2_CD86 + Lymph_Node.mAPC_Int_P2 |
| 64 | Lymph_Node.mAPC_Int_P2 -> Lymph_Node.mAPC_Int_P2_PD1 + Lymph_Node.mAPC_Int_P2 |
| 65 | Lymph_Node.mAPC_Int_P2 -> Lymph_Node.mAPC_Int_P2_PDL1 + Lymph_Node.mAPC_Int_P2 |
| 66 | Lymph_Node.mAPC_Int_P2 -> Lymph_Node.mAPC_Int_P2_PDL2 + Lymph_Node.mAPC_Int_P2 |
| 67 | Lymph_Node.mAPC_Int_P2_CD80 -> null |
| 68 | Lymph_Node.mAPC_Int_P2_CD80 + Lymph_Node.PNT_CTLA4 <-> Lymph_Node.NEG_Sig_PNT_CD80 |
| 69 | Lymph_Node.mAPC_Int_P2_CD80 + Lymph_Node.PNT_Int_PDL1 <-> Lymph_Node.[PNT_PDL1-CD80] |
| 70 | Lymph_Node.mAPC_Int_P2_CD86 -> null |
| 71 | Lymph_Node.mAPC_Int_P2_CD86 + Lymph_Node.PNT_CTLA4 <-> Lymph_Node.NEG_Sig_PNT_CD86 |
| 72 | Lymph_Node.mAPC_Int_P2_CD86 + Lymph_Node.PNT_Int_CD28 <-> Lymph_Node.POS_Sig_PNT_CD86 |
| 73 | Lymph_Node.mAPC_Int_P2_PD1 -> null |
| 74 | Lymph_Node.mAPC_Int_P2_PD1 + Lymph_Node.PD1_mab <-> Lymph_Node.PD1mAb_mAPC_PD1 |
| 75 | Lymph_Node.mAPC_Int_P2_PDL1 -> null |
| 76 | Lymph_Node.mAPC_Int_P2_PDL2 -> null |
| 77 | Lymph_Node.mAPC_Int_P2_PDL2 + Lymph_Node.PNT_Int_PD1 <-> Lymph_Node.[PNT_PD1-PDL2] |
| 78 | Lymph_Node.mAPC_nInt -> Lymph_Node.mAPC_nInt + Lymph_Node.mAPC_nInt_CD80 |
| 79 | Lymph_Node.mAPC_nInt -> Lymph_Node.mAPC_nInt + Lymph_Node.mAPC_nInt_CD86 |
| 80 | Lymph_Node.mAPC_nInt_CD80 -> null |
| 81 | Lymph_Node.mAPC_nInt_CD80 + Lymph_Node.TrLN_CTLA4S <-> Lymph_Node.CTLA4S_CD80 |
| 82 | Lymph_Node.mAPC_nInt_CD86 -> null |
| 83 | Lymph_Node.mAPC_nInt_CD86 + Lymph_Node.TrLN_CTLA4S <-> Lymph_Node.CTLA4S_CD86 |
| 84 | Lymph_Node.mAPC_PD1 -> null |
| 85 | Lymph_Node.mAPC_PDL1 -> null |
| 86 | Lymph_Node.mAPC_PDL2 -> null |
| 87 | Lymph_Node.Naive_T -> Lymph_Node.Naive_T1 |
| 88 | Lymph_Node.Naive_T -> null |
| 89 | Lymph_Node.Naive_T1 -> Lymph_Node.Naive_T |
| 90 | Lymph_Node.Naive_T1 -> Lymph_Node.Primed_Naive_T |
| 91 | Lymph_Node.NEG_Sig_PNT_CD80 -> null |
| 92 | Lymph_Node.NEG_Sig_PNT_CD86 -> null |
| 93 | Lymph_Node.NT_Int_CD28 -> null |
| 94 | Lymph_Node.NT_Int_CD28 + Lymph_Node.mAPC_Int_P1_CD80 <-> Lymph_Node.POS_Sig_NT_CD80 |
| 95 | Lymph_Node.NT_Int_CD28 + Lymph_Node.mAPC_Int_P1_CD86 <-> Lymph_Node.POS_Sig_NT_CD86 |
| 96 | Lymph_Node.NT1_Int1 -> Lymph_Node.NT1_Int1 + Lymph_Node.NT_Int_CD28 |
| 97 | Lymph_Node.PD1_mab -> null |
| 98 | Lymph_Node.PDL1_mab -> null |
| 99 | Lymph_Node.PDL1_mab + Lymph_Node.mAPC_Int_P2_PDL1 <-> Lymph_Node.[PDL1mAb-mAPC_PDL1] |
| 100 | Lymph_Node.PDL1_mab + Lymph_Node.PNT_Int_PDL1 <-> Lymph_Node.[PDL1mAb-PNT_PDL1] |
| 101 | Lymph_Node.PNT_CTLA4 -> null |
| 102 | Lymph_Node.PNT_Int_CD28 -> null |
| 103 | Lymph_Node.PNT_Int_CD28 + Lymph_Node.mAPC_Int_P2_CD80 <-> Lymph_Node.POS_Sig_PNT_CD80 |
| 104 | Lymph_Node.PNT_Int_CD80 -> null |
| 105 | Lymph_Node.PNT_Int_CD80 + Lymph_Node.mAPC_Int_P2_PDL1 <-> Lymph_Node.[PNT_CD80-PDL1] |
| 106 | Lymph_Node.PNT_Int_PD1 -> null |
| 107 | Lymph_Node.PNT_Int_PD1 + Lymph_Node.mAPC_Int_P2_PDL1 <-> Lymph_Node.[PNT_PD1-PDL1] |
| 108 | Lymph_Node.PNT_Int_PD1 + Lymph_Node.PD1_mab <-> Lymph_Node.PD1mAb_PNT_PD1 |
| 109 | Lymph_Node.PNT_Int_PDL1 -> null |
| 110 | Lymph_Node.PNT_Int_PDL1 + Lymph_Node.mAPC_Int_P2_PD1 <-> Lymph_Node.[PNT_PDL1-PD1] |
| 111 | Lymph_Node.PNT1_Int -> Lymph_Node.PNT_CTLA4 + Lymph_Node.PNT1_Int |
| 112 | Lymph_Node.PNT1_Int -> Lymph_Node.PNT_Int_CD28 + Lymph_Node.PNT1_Int |
| 113 | Lymph_Node.PNT1_Int -> Lymph_Node.PNT_Int_CD80 + Lymph_Node.PNT1_Int |
| 114 | Lymph_Node.PNT1_Int -> Lymph_Node.PNT1_Int + Lymph_Node.PNT_Int_PD1 |
| 115 | Lymph_Node.PNT1_Int -> Lymph_Node.PNT1_Int + Lymph_Node.PNT_Int_PDL1 |
| 116 | Lymph_Node.Primed_Naive_T -> Lymph_Node.Primed_Naive_T1 |
| 117 | Lymph_Node.Primed_Naive_T1 -> Lymph_Node.Anergic_Naive_T |
| 118 | Lymph_Node.Primed_Naive_T1 -> Lymph_Node.Primed_Naive_T |
| 119 | Lymph_Node.Primed_Naive_T1 -> Lymph_Node.Prolif_Naive_T |
| 120 | Lymph_Node.Prolif_Naive_T -> Lymph_Node.Effector_T |
| 121 | Lymph_Node.Prolif_Naive_T -> null |
| 122 | Lymph_Node.TrALN_CT_aCT -> null |
| 123 | Lymph_Node.TrALN_CT_CD80 -> null |
| 124 | Lymph_Node.TrALN_CT_CD86 -> null |
| 125 | Lymph_Node.TregLN -> Lymph_Node.TregLN + Lymph_Node.TrLN_CTLA4 |
| 126 | Lymph_Node.TregLN -> Lymph_Node.TregLN_Int + Lymph_Node.TregLN |
| 127 | Lymph_Node.TregLN_Int + Lymph_Node.mAPC -> Lymph_Node.TregLN_mAPC + Lymph_Node.mAPC |
| 128 | Lymph_Node.TregLN_Int + Lymph_Node.Naive_T -> Lymph_Node.[TregLN-NT] + Lymph_Node.Naive_T |
| 129 | Lymph_Node.TregLN_Int + Lymph_Node.Primed_Naive_T -> Lymph_Node.[TregLN-PNT] + Lymph_Node.Primed_Naive_T |
| 130 | Lymph_Node.TregLN_mAPC -> null |
| 131 | Lymph_Node.TregLN_mAPC + Lymph_Node.mAPC -> null |
| 132 | Lymph_Node.TregLN_mAPC1 -> Lymph_Node.[Tr-mAPC_CD80] + Lymph_Node.TregLN_mAPC1 |
| 133 | Lymph_Node.TregLN_mAPC1 -> Lymph_Node.[Tr-mAPC_CD86] + Lymph_Node.TregLN_mAPC1 |
| 134 | Lymph_Node.TregLN_mAPC1 -> Lymph_Node.[Tr-mAPC_CTLA4] + Lymph_Node.TregLN_mAPC1 |
| 135 | Lymph_Node.TregLN_Secrete -> Lymph_Node.TrLN_CTLA4S + Lymph_Node.TregLN_Secrete |
| 136 | Lymph_Node.TrLN_CTLA4 -> null |
| 137 | Lymph_Node.TrLN_CTLA4 + Lymph_Node.CTLA4_mab <-> Lymph_Node.TrLN_CT_aCT |
| 138 | Lymph_Node.TrLN_CTLA4S + Lymph_Node.CTLA4_mab <-> Lymph_Node.TrLN_CTLA4S_aCTLA4 |
| 139 | null -> [Blood-Lymph].CTLA4_mabB |
| 140 | null -> [Blood-Lymph].CTLA4_mabB |
| 141 | null -> [Blood-Lymph].PD1_mabB |
| 142 | null -> [Blood-Lymph].PD1_mabB |
| 143 | null -> [Blood-Lymph].PDL1_mabB |
| 144 | null -> [Blood-Lymph].PDL1_mabB |
| 145 | null -> Lymph_Node.CTLA4_mab |
| 146 | null -> Lymph_Node.CTLA4_mab |
| 147 | null -> Lymph_Node.Naive_T |
| 148 | null -> Lymph_Node.PD1_mab |
| 149 | null -> Lymph_Node.PD1_mab |
| 150 | null -> Lymph_Node.PDL1_mab |
| 151 | null -> Lymph_Node.PDL1_mab |
| 152 | null -> Lymph_Node.Prolif_Naive_T |
| 153 | null -> Tumor.APC_T |
| 154 | null -> Tumor.Cancer |
| 155 | null -> Tumor.Effector_TT |
| 156 | Peripheral.Effector_Ta -> null |
| 157 | Peripheral.Effector_Ta -> Peripheral.Effector_TP |
| 158 | Peripheral.Effector_Tb -> null |
| 159 | Peripheral.Effector_Tb -> Peripheral.Effector_Ta |
| 160 | Peripheral.Effector_Tf <-> Peripheral.Effector_Tb |
| 161 | Peripheral.Effector_TP -> [Blood-Lymph].Effector_TB |
| 162 | Tumor.[C{CD80}] -> Tumor.[C{CD80}] + Tumor.[C5=CD80] |
| 163 | Tumor.[C{PD1}] -> Tumor.[C2=PD1] + Tumor.[C{PD1}] |
| 164 | Tumor.[C{PD1}{CD80}] -> Tumor.[C{PD1}{CD80}] + Tumor.[C9a=PD1] |
| 165 | Tumor.[C{PD1}{CD80}] -> Tumor.[C{PD1}{CD80}] + Tumor.[C9b=CD80] |
| 166 | Tumor.[C{PD1}{PDL1}] -> Tumor.[C6a=PD1] + Tumor.[C{PD1}{PDL1}] |
| 167 | Tumor.[C{PD1}{PDL1}] -> Tumor.[C6b=PDL1] + Tumor.[C{PD1}{PDL1}] |
| 168 | Tumor.[C{PD1}{PDL1}{CD80}] -> Tumor.[C{PD1}{PDL1}{CD80}] + Tumor.[C14a=PD1] |
| 169 | Tumor.[C{PD1}{PDL1}{CD80}] -> Tumor.[C{PD1}{PDL1}{CD80}] + Tumor.[C14b=PDL1] |
| 170 | Tumor.[C{PD1}{PDL1}{CD80}] -> Tumor.[C{PD1}{PDL1}{CD80}] + Tumor.[C14c=CD80] |
| 171 | Tumor.[C{PD1}{PDL1}{PDL2}] -> Tumor.[C{PD1}{PDL1}{PDL2}] + Tumor.[C10a=PD1] |
| 172 | Tumor.[C{PD1}{PDL1}{PDL2}] -> Tumor.[C{PD1}{PDL1}{PDL2}] + Tumor.[C10b=PDL1] |
| 173 | Tumor.[C{PD1}{PDL1}{PDL2}] -> Tumor.[C{PD1}{PDL1}{PDL2}] + Tumor.[C10c=PDL2] |
| 174 | Tumor.[C{PD1}{PDL1}{PDL2}{CD80}] -> Tumor.[C{PD1}{PDL1}{PDL2}{CD80}] + Tumor.[C16a=PD1] |
| 175 | Tumor.[C{PD1}{PDL1}{PDL2}{CD80}] -> Tumor.[C{PD1}{PDL1}{PDL2}{CD80}] + Tumor.[C16b=PDL1] |
| 176 | Tumor.[C{PD1}{PDL1}{PDL2}{CD80}] -> Tumor.[C{PD1}{PDL1}{PDL2}{CD80}] + Tumor.[C16c=PDL2] |
| 177 | Tumor.[C{PD1}{PDL1}{PDL2}{CD80}] -> Tumor.[C{PD1}{PDL1}{PDL2}{CD80}] + Tumor.[C16d=CD80] |
| 178 | Tumor.[C{PD1}{PDL2}] -> Tumor.[C{PD1}{PDL2}] + Tumor.[C7a=PD1] |
| 179 | Tumor.[C{PD1}{PDL2}] -> Tumor.[C{PD1}{PDL2}] + Tumor.[C7b=PDL2] |
| 180 | Tumor.[C{PD1}{PDL2}{CD80}] -> Tumor.[C{PD1}{PDL2}{CD80}] + Tumor.[C15a=PD1] |
| 181 | Tumor.[C{PD1}{PDL2}{CD80}] -> Tumor.[C{PD1}{PDL2}{CD80}] + Tumor.[C15b=PDL2] |
| 182 | Tumor.[C{PD1}{PDL2}{CD80}] -> Tumor.[C{PD1}{PDL2}{CD80}] + Tumor.[C15c=CD80] |
| 183 | Tumor.[C{PDL1}] -> Tumor.[C{PDL1}] + Tumor.[C3=PDL1] |
| 184 | Tumor.[C{PDL1}{CD80}] -> Tumor.[C{PDL1}{CD80}] + Tumor.[C11a=PDL1] |
| 185 | Tumor.[C{PDL1}{CD80}] -> Tumor.[C{PDL1}{CD80}] + Tumor.[C11b=CD80] |
| 186 | Tumor.[C{PDL1}{PDL2}] -> Tumor.[C{PDL1}{PDL2}] + Tumor.[C8a=PDL1] |
| 187 | Tumor.[C{PDL1}{PDL2}] -> Tumor.[C{PDL1}{PDL2}] + Tumor.[C8b=PDL2] |
| 188 | Tumor.[C{PDL1}{PDL2}{CD80}] -> Tumor.[C{PDL1}{PDL2}{CD80}] + Tumor.[C13a=PDL1] |
| 189 | Tumor.[C{PDL1}{PDL2}{CD80}] -> Tumor.[C{PDL1}{PDL2}{CD80}] + Tumor.[C13b=PDL2] |
| 190 | Tumor.[C{PDL1}{PDL2}{CD80}] -> Tumor.[C{PDL1}{PDL2}{CD80}] + Tumor.[C13c=CD80] |
| 191 | Tumor.[C{PDL2}] -> Tumor.[C{PDL2}] + Tumor.[C4=PDL2] |
| 192 | Tumor.[C{PDL2}{CD80}] -> Tumor.[C{PDL2}{CD80}] + Tumor.[C12a=PDL2] |
| 193 | Tumor.[C{PDL2}{CD80}] -> Tumor.[C{PDL2}{CD80}] + Tumor.[C12b=CD80] |
| 194 | Tumor.[C10a=PD1] -> null |
| 195 | Tumor.[C10a=PD1] + Tumor.PD1_mabT <-> Tumor.[C10a=PD1:aPD1] |
| 196 | Tumor.[C10b=PDL1] -> null |
| 197 | Tumor.[C10b=PDL1] + Tumor.PDL1_mabT <-> Tumor.[C10b=PDL1:aPDL1] |
| 198 | Tumor.[C10c=PDL2] -> null |
| 199 | Tumor.[C11a=PDL1] -> null |
| 200 | Tumor.[C11a=PDL1] + Tumor.PDL1_mabT <-> Tumor.[C11a=PDL1:aPDL1] |
| 201 | Tumor.[C11b=CD80] -> null |
| 202 | Tumor.[C12a=PDL2] -> null |
| 203 | Tumor.[C12b=CD80] -> null |
| 204 | Tumor.[C13a=PDL1] -> null |
| 205 | Tumor.[C13a=PDL1] + Tumor.PDL1_mabT <-> Tumor.[C13a=PDL1:aPDL1] |
| 206 | Tumor.[C13b=PDL2] -> null |
| 207 | Tumor.[C13c=CD80] -> null |
| 208 | Tumor.[C14a=PD1] -> null |
| 209 | Tumor.[C14a=PD1] + Tumor.[T14a=PDL1] <-> Tumor.[T14a=PDL1:PD1=C14a] |
| 210 | Tumor.[C14b=PDL1] -> null |
| 211 | Tumor.[C14b=PDL1] + Tumor.[T14c=CD80] <-> Tumor.[T14c=CD80:PDL1=C14b] |
| 212 | Tumor.[C14b=PDL1] + Tumor.PDL1_mabT <-> Tumor.[C14b=PDL1:aPDL1] |
| 213 | Tumor.[C14c=CD80] -> null |
| 214 | Tumor.[C14c=CD80] + Tumor.[T14a=PDL1] <-> Tumor.[T14a=PDL1:CD80=C14c] |
| 215 | Tumor.[C15a=PD1] -> null |
| 216 | Tumor.[C15a=PD1] + Tumor.PD1_mabT <-> Tumor.[C15a=PD1:aPD1] |
| 217 | Tumor.[C15b=PDL2] -> null |
| 218 | Tumor.[C15b=PDL2] + Tumor.[T15b=PD1] <-> Tumor.[T15b=PD1:PDL2=C15b] |
| 219 | Tumor.[C15c=CD80] -> null |
| 220 | Tumor.[C15c=CD80] + Tumor.[T15a=PDL1] <-> Tumor.[T15a=PDL1:CD80=C15c] |
| 221 | Tumor.[C16a=PD1] -> null |
| 222 | Tumor.[C16a=PD1] + Tumor.[T16a=PDL1] <-> Tumor.[T16a=PDL1:PD1=C16a] |
| 223 | Tumor.[C16a=PD1] + Tumor.PD1_mabT <-> Tumor.[C16a=PD1:aPD1] |
| 224 | Tumor.[C16b=PDL1] -> null |
| 225 | Tumor.[C16b=PDL1] + Tumor.PDL1_mabT <-> Tumor.[C16b=PDL1:aPDL1] |
| 226 | Tumor.[C16c=PDL2] -> null |
| 227 | Tumor.[C16c=PDL2] + Tumor.[T16b=PD1] <-> Tumor.[T16b=PD1:PDL2=C16c] |
| 228 | Tumor.[C16d=CD80] -> null |
| 229 | Tumor.[C2=PD1] -> null |
| 230 | Tumor.[C3=PDL1] -> null |
| 231 | Tumor.[C4=PDL2] -> null |
| 232 | Tumor.[C5=CD80] -> null |
| 233 | Tumor.[C6a=PD1] -> null |
| 234 | Tumor.[C6a=PD1] + Tumor.PD1_mabT <-> Tumor.[C6a=PD1:aPD1] |
| 235 | Tumor.[C6b=PDL1] -> null |
| 236 | Tumor.[C6b=PDL1] + Tumor.[T6b=PD1] <-> Tumor.[T6b=PD1:PDL1=C6b] |
| 237 | Tumor.[C6b=PDL1] + Tumor.[T6c=CD80] <-> Tumor.[T6c=CD80:PDL1=C6b] |
| 238 | Tumor.[C6b=PDL1] + Tumor.PDL1_mabT <-> Tumor.[C6b=PDL1:aPDL1] |
| 239 | Tumor.[C7a=PD1] -> null |
| 240 | Tumor.[C7b=PDL2] -> null |
| 241 | Tumor.[C8a=PDL1] -> null |
| 242 | Tumor.[C8a=PDL1] + Tumor.[T8b=CD80] <-> Tumor.[T8b=CD80:PDL1=C8a] |
| 243 | Tumor.[C8a=PDL1] + Tumor.PDL1_mabT <-> Tumor.[C8a=PDL1:aPDL1] |
| 244 | Tumor.[C8b=PDL2] -> null |
| 245 | Tumor.[C9a=PD1] -> null |
| 246 | Tumor.[C9a=PD1] + Tumor.[T9=PDL1] <-> Tumor.[T9=PDL1-PD1=C9a] |
| 247 | Tumor.[C9b=CD80] -> null |
| 248 | Tumor.[CTLA4_CTLA4-Trt] -> null |
| 249 | Tumor.[CTLA4:CD80_TrAT] -> null |
| 250 | Tumor.[CTLA4:CD86_TrAT] -> null |
| 251 | Tumor.[T{PD1}-{PDL2}C] -> Tumor.[T{PD1}-{PDL2}C] + Tumor.[T4=PD1] |
| 252 | Tumor.[T{PD1}{80}-{PDL1}{PDL2}C] -> Tumor.[T{PD1}{80}-{PDL1}{PDL2}C] + Tumor.[T8a=PD1] |
| 253 | Tumor.[T{PD1}{80}-{PDL1}{PDL2}C] -> Tumor.[T{PD1}{80}-{PDL1}{PDL2}C] + Tumor.[T8b=CD80] |
| 254 | Tumor.[T{PD1}{CD80}-{PDL1}C] -> Tumor.[T{PD1}{CD80}-{PDL1}C] + Tumor.[T3a=PD1] |
| 255 | Tumor.[T{PD1}{CD80}-{PDL1}C] -> Tumor.[T{PD1}{CD80}-{PDL1}C] + Tumor.[T3b=CD80] |
| 256 | Tumor.[T{PD1}{L1}-{PD1}{80}{PDL2}C] -> Tumor.[T{PD1}{L1}-{PD1}{80}{PDL2}C] + Tumor.[T15a=PDL1] |
| 257 | Tumor.[T{PD1}{L1}-{PD1}{80}{PDL2}C] -> Tumor.[T{PD1}{L1}-{PD1}{80}{PDL2}C] + Tumor.[T15b=PD1] |
| 258 | Tumor.[T{PD1}{L1}-{PD1}{L2}C] -> Tumor.[T{PD1}{L1}-{PD1}{L2}C] + Tumor.[T7a=PDL1] |
| 259 | Tumor.[T{PD1}{L1}-{PD1}{L2}C] -> Tumor.[T{PD1}{L1}-{PD1}{L2}C] + Tumor.[T7b=PD1] |
| 260 | Tumor.[T{PD1}{L1}-{PDL1}{80}{L2}C] -> Tumor.[T{PD1}{L1}-{PDL1}{80}{L2}C] + Tumor.[T13a=PDL1] |
| 261 | Tumor.[T{PD1}{L1}-{PDL1}{80}{L2}C] -> Tumor.[T{PD1}{L1}-{PDL1}{80}{L2}C] + Tumor.[T13b=PD1] |
| 262 | Tumor.[T{PD1}{L1}-{PDL1}{80}C] -> Tumor.[T{PD1}{L1}-{PDL1}{80}C] + Tumor.[T11a=PDL1] |
| 263 | Tumor.[T{PD1}{L1}-{PDL1}{80}C] -> Tumor.[T{PD1}{L1}-{PDL1}{80}C] + Tumor.[T11b=PD1] |
| 264 | Tumor.[T{PD1}{L1}-{PDL2}{80}C] -> Tumor.[T{PD1}{L1}-{PDL2}{80}C] + Tumor.[T12a=PDL1] |
| 265 | Tumor.[T{PD1}{L1}-{PDL2}{80}C] -> Tumor.[T{PD1}{L1}-{PDL2}{80}C] + Tumor.[T12b=PD1] |
| 266 | Tumor.[T{PD1}{L1}{80}-{PD1}{80}{L1}{L2}C] -> Tumor.[T{PD1}{L1}{80}-{PD1}{80}{L1}{L2}C] + Tumor.[T16a=PDL1] |
| 267 | Tumor.[T{PD1}{L1}{80}-{PD1}{80}{L1}{L2}C] -> Tumor.[T{PD1}{L1}{80}-{PD1}{80}{L1}{L2}C] + Tumor.[T16b=PD1] |
| 268 | Tumor.[T{PD1}{L1}{80}-{PD1}{80}{L1}{L2}C] -> Tumor.[T{PD1}{L1}{80}-{PD1}{80}{L1}{L2}C] + Tumor.[T16c=CD80] |
| 269 | Tumor.[T{PD1}{L1}{80}-{PD1}{80}{L1}C] -> Tumor.[T{PD1}{L1}{80}-{PD1}{80}{L1}C] + Tumor.[T14a=PDL1] |
| 270 | Tumor.[T{PD1}{L1}{80}-{PD1}{80}{L1}C] -> Tumor.[T{PD1}{L1}{80}-{PD1}{80}{L1}C] + Tumor.[T14b=PD1] |
| 271 | Tumor.[T{PD1}{L1}{80}-{PD1}{80}{L1}C] -> Tumor.[T{PD1}{L1}{80}-{PD1}{80}{L1}C] + Tumor.[T14c=CD80] |
| 272 | Tumor.[T{PD1}{L1}{80}-{PD1}{L1}{L2}C] -> Tumor.[T{PD1}{L1}{80}-{PD1}{L1}{L2}C] + Tumor.[T10a=PDL1] |
| 273 | Tumor.[T{PD1}{L1}{80}-{PD1}{L1}{L2}C] -> Tumor.[T{PD1}{L1}{80}-{PD1}{L1}{L2}C] + Tumor.[T10b=PD1] |
| 274 | Tumor.[T{PD1}{L1}{80}-{PD1}{L1}{L2}C] -> Tumor.[T{PD1}{L1}{80}-{PD1}{L1}{L2}C] + Tumor.[T10c=CD80] |
| 275 | Tumor.[T{PD1}{L1}{80}-{PD1}{L1}C] -> Tumor.[T{PD1}{L1}{80}-{PD1}{L1}C] + Tumor.[T6a=PDL1] |
| 276 | Tumor.[T{PD1}{L1}{80}-{PD1}{L1}C] -> Tumor.[T{PD1}{L1}{80}-{PD1}{L1}C] + Tumor.[T6b=PD1] |
| 277 | Tumor.[T{PD1}{L1}{80}-{PD1}{L1}C] -> Tumor.[T{PD1}{L1}{80}-{PD1}{L1}C] + Tumor.[T6c=CD80] |
| 278 | Tumor.[T{PDL1}-{CD80}{PD1}C] -> Tumor.[T{PDL1}-{CD80}{PD1}C] + Tumor.[T9=PDL1] |
| 279 | Tumor.[T{PDL1}-{CD80}C] -> Tumor.[T{PDL1}-{CD80}C] + Tumor.[T5=PDL1] |
| 280 | Tumor.[T{PDL1}-{PD1}C] -> Tumor.[T{PDL1}-{PD1}C] + Tumor.[T2=PDL1] |
| 281 | Tumor.[T10a=PDL1] -> null |
| 282 | Tumor.[T10a=PDL1] + Tumor.[C10a=PD1] <-> Tumor.[T10a=PDL1:PD1=C10a] |
| 283 | Tumor.[T10a=PDL1] + Tumor.PDL1_mabT <-> Tumor.[T10a=PDL1:aPDL1] |
| 284 | Tumor.[T10b=PD1] -> null |
| 285 | Tumor.[T10b=PD1] + Tumor.[C10b=PDL1] <-> Tumor.[T10b=PD1:PDL1=C10b] |
| 286 | Tumor.[T10b=PD1] + Tumor.[C10c=PDL2] <-> Tumor.[T10b=PD1:PDL2=C10c] |
| 287 | Tumor.[T10c=CD80] -> null |
| 288 | Tumor.[T10c=CD80] + Tumor.[C10b=PDL1] <-> Tumor.[T10c=CD80:PDL1=C10b] |
| 289 | Tumor.[T11a=PDL1] -> null |
| 290 | Tumor.[T11a=PDL1] + Tumor.[C11b=CD80] <-> Tumor.[T11a=PDL1:CD80=C11b] |
| 291 | Tumor.[T11a=PDL1] + Tumor.PDL1_mabT <-> Tumor.[T11a=PDL1:aPDL1] |
| 292 | Tumor.[T11b=PD1] -> null |
| 293 | Tumor.[T11b=PD1] + Tumor.[C11a=PDL1] <-> Tumor.[T11b=PD1:PDL1=C11a] |
| 294 | Tumor.[T12a=PDL1] -> null |
| 295 | Tumor.[T12a=PDL1] + Tumor.[C12b=CD80] <-> Tumor.[T12a=PDL1:CD80=C12b] |
| 296 | Tumor.[T12a=PDL1] + Tumor.PDL1_mabT <-> Tumor.[T12a=PDL1:aPDL1] |
| 297 | Tumor.[T12b=PD1] -> null |
| 298 | Tumor.[T12b=PD1] + Tumor.[C12a=PDL2] <-> Tumor.[T12b=PD1:PDL2=C12a] |
| 299 | Tumor.[T13a=PDL1] -> null |
| 300 | Tumor.[T13a=PDL1] + Tumor.[C13c=CD80] <-> Tumor.[T13a=PDL1:CD80=C13c] |
| 301 | Tumor.[T13b=PD1] -> null |
| 302 | Tumor.[T13b=PD1] + Tumor.[C13a=PDL1] <-> Tumor.[T13b=PD1:PDL1=C13a] |
| 303 | Tumor.[T13b=PD1] + Tumor.[C13b=PDL2] <-> Tumor.[T13b=PD1:PDL2=C13b] |
| 304 | Tumor.[T13b=PD1] + Tumor.PD1_mabT <-> Tumor.[T13b=PD1:aPD1] |
| 305 | Tumor.[T14a=PDL1] -> null |
| 306 | Tumor.[T14a=PDL1] + Tumor.PDL1_mabT <-> Tumor.[T14a=PDL1:aPDL1] |
| 307 | Tumor.[T14b=PD1] -> null |
| 308 | Tumor.[T14b=PD1] + Tumor.[C14b=PDL1] <-> Tumor.[T14b=PD1:PDL1=C14b] |
| 309 | Tumor.[T14c=CD80] -> null |
| 310 | Tumor.[T15a=PDL1] -> null |
| 311 | Tumor.[T15a=PDL1] + Tumor.[C15a=PD1] <-> Tumor.[T15a=PDL1:PD1=C15a] |
| 312 | Tumor.[T15a=PDL1] + Tumor.PDL1_mabT <-> Tumor.[T15a=PDL1:aPDL1] |
| 313 | Tumor.[T15b=PD1] -> null |
| 314 | Tumor.[T15b=PD1] + Tumor.PD1_mabT <-> Tumor.[T15b=PD1:aPD1] |
| 315 | Tumor.[T16a=PDL1] -> null |
| 316 | Tumor.[T16a=PDL1] + Tumor.[C16d=CD80] <-> Tumor.[T16a=PDL1:CD80=C16d] |
| 317 | Tumor.[T16a=PDL1] + Tumor.PDL1_mabT <-> Tumor.[T16a=PDL1:aPDL1] |
| 318 | Tumor.[T16b=PD1] -> null |
| 319 | Tumor.[T16b=PD1] + Tumor.[C16b=PDL1] <-> Tumor.[T16b=PD1:PDL1=C16b] |
| 320 | Tumor.[T16b=PD1] + Tumor.PD1_mabT <-> Tumor.[T16b=PD1:aPD1] |
| 321 | Tumor.[T16c=CD80] -> null |
| 322 | Tumor.[T16c=CD80] + Tumor.[C16b=PDL1] <-> Tumor.[T16c=CD80:PDL1=C16b] |
| 323 | Tumor.[T2=PDL1] -> null |
| 324 | Tumor.[T2=PDL1] + Tumor.[C2=PD1] <-> Tumor.[T2=PDL1:PD1=C2] |
| 325 | Tumor.[T3a=PD1] -> null |
| 326 | Tumor.[T3a=PD1] + Tumor.[C3=PDL1] <-> Tumor.[T3a=PD1:PDL1=C3] |
| 327 | Tumor.[T3a=PD1] + Tumor.PD1_mabT <-> Tumor.[T3a=PD1:aPD1] |
| 328 | Tumor.[T3b=CD80] -> null |
| 329 | Tumor.[T3b=CD80] + Tumor.[C3=PDL1] <-> Tumor.[T3b=CD80:PDL1=C3] |
| 330 | Tumor.[T4=PD1] -> null |
| 331 | Tumor.[T4=PD1] + Tumor.[C4=PDL2] <-> Tumor.[T4=PD1:PDL2=C4] |
| 332 | Tumor.[T4=PD1] + Tumor.PD1_mabT <-> Tumor.[T4=PD1:aPD1] |
| 333 | Tumor.[T5=PDL1] -> null |
| 334 | Tumor.[T5=PDL1] + Tumor.[C5=CD80] <-> Tumor.[T5=PDL1:CD80=C5] |
| 335 | Tumor.[T5=PDL1] + Tumor.PDL1_mabT <-> Tumor.[T5=PDL1:aPDL1] |
| 336 | Tumor.[T6a=PDL1] -> null |
| 337 | Tumor.[T6a=PDL1] + Tumor.[C6a=PD1] <-> Tumor.[T6a=PDL1:PD1=C6a] |
| 338 | Tumor.[T6a=PDL1] + Tumor.PDL1_mabT <-> Tumor.[T6a=PDL1:aPDL1] |
| 339 | Tumor.[T6b=PD1] -> null |
| 340 | Tumor.[T6b=PD1] + Tumor.PD1_mabT <-> Tumor.[T6b=PD1:aPD1] |
| 341 | Tumor.[T6c=CD80] -> null |
| 342 | Tumor.[T7a=PDL1] -> null |
| 343 | Tumor.[T7a=PDL1] + Tumor.[C7a=PD1] <-> Tumor.[T7a=PDL1:PD1=C7a] |
| 344 | Tumor.[T7b=PD1] -> null |
| 345 | Tumor.[T7b=PD1] + Tumor.[C7b=PDL2] <-> Tumor.[T7b=PD1:PDL2=C7b] |
| 346 | Tumor.[T8a=PD1] -> null |
| 347 | Tumor.[T8a=PD1] + Tumor.[C8a=PDL1] <-> Tumor.[T8a=PD1:PDL1=C8a] |
| 348 | Tumor.[T8a=PD1] + Tumor.[C8b=PDL2] <-> Tumor.[T8a=PD1:PDL2=C8b] |
| 349 | Tumor.[T8b=CD80] -> null |
| 350 | Tumor.[T9=PDL1] -> null |
| 351 | Tumor.[T9=PDL1] + Tumor.[C9b=CD80] <-> Tumor.[T9=PDL1-CD80=C9b] |
| 352 | Tumor.[T9=PDL1] + Tumor.PDL1_mabT <-> Tumor.[T9=PDL1:aPDL1] |
| 353 | Tumor.APC_T -> null |
| 354 | Tumor.C_DebrisT -> Lymph_Node.C_DebrisLN |
| 355 | Tumor.C_DebrisT -> null |
| 356 | Tumor.C_DebrisT -> null |
| 357 | Tumor.C_DebrisT -> null |
| 358 | Tumor.C_DebrisT + Tumor.APC_T -> Tumor.mAPC_T |
| 359 | Tumor.Cancer -> 19 Tumor.C_DebrisT |
| 360 | Tumor.CD80_mAPCT -> null |
| 361 | Tumor.CD80_TeffT -> null |
| 362 | Tumor.CD80_TeffT + Tumor.PDL1_TregT <-> Tumor.[PDL1:CD80_TrTeff] |
| 363 | Tumor.CD80_TeffT1 -> null |
| 364 | Tumor.CD86_mAPCT -> null |
| 365 | Tumor.CTLA4_mabT -> null |
| 366 | Tumor.CTLA4_mabT + Tumor.CTLA4_TregTS <-> Tumor.[CTLA4_aCTLA4-TrTS] |
| 367 | Tumor.CTLA4_TregT -> null |
| 368 | Tumor.CTLA4_TregT + Tumor.CD80_mAPCT <-> Tumor.[CTLA4:CD80_TrAT] |
| 369 | Tumor.CTLA4_TregT + Tumor.CD86_mAPCT <-> Tumor.[CTLA4:CD86_TrAT] |
| 370 | Tumor.CTLA4_TregT + Tumor.CTLA4_mabT <-> Tumor.[CTLA4_CTLA4-Trt] |
| 371 | Tumor.CTLA4_TregTS -> null |
| 372 | Tumor.Effector_Ta -> null |
| 373 | Tumor.Effector_Ta -> Tumor.Effector_TT |
| 374 | Tumor.Effector_Tb -> null |
| 375 | Tumor.Effector_Tb -> Tumor.Effector_Ta |
| 376 | Tumor.Effector_Tf <-> Tumor.Effector_Tb |
| 377 | Tumor.Effector_TT -> null |
| 378 | Tumor.Effector_TT_C_Eng + Tumor.Cancer1 -> Tumor.Effector_TT_C_Eng + Tumor.TC1 + Tumor.Cancer1 |
| 379 | Tumor.Exhausted_TT -> null |
| 380 | Tumor.mAPC_T -> Lymph_Node.mAPC |
| 381 | Tumor.mAPC_T -> null |
| 382 | Tumor.mAPC_T -> null |
| 383 | Tumor.mAPCT_EngTregT -> Tumor.CD80_mAPCT + Tumor.mAPCT_EngTregT |
| 384 | Tumor.mAPCT_EngTregT -> Tumor.CD86_mAPCT + Tumor.mAPCT_EngTregT |
| 385 | Tumor.MDSC_T + Tumor.Effector_TT_MDSCs -> Tumor.MDSCsT_Teff + Tumor.Effector_TT_MDSCs + Tumor.MDSC_T |
| 386 | Tumor.MDSCsT_EngTeff -> Tumor.PD1_MDSCsT + Tumor.MDSCsT_EngTeff |
| 387 | Tumor.MDSCsT_EngTeff -> Tumor.PDL1_MDSCsT + Tumor.MDSCsT_EngTeff |
| 388 | Tumor.MDSCsT_Teff -> null |
| 389 | Tumor.MDSCsT_Teff + Tumor.Effector_TT -> null |
| 390 | Tumor.PD1_mabT -> null |
| 391 | Tumor.PD1_mabT + Tumor.[C14a=PD1] <-> Tumor.[C14a=PD1:aPD1] |
| 392 | Tumor.PD1_mabT + Tumor.[C2=PD1] <-> Tumor.[C2=PD1:aPD1] |
| 393 | Tumor.PD1_mabT + Tumor.[C7a=PD1] <-> Tumor.[C7a=PD1:aPD1] |
| 394 | Tumor.PD1_mabT + Tumor.[C9a=PD1] <-> Tumor.[C9a=PD1:aPD1] |
| 395 | Tumor.PD1_mabT + Tumor.[T10b=PD1] <-> Tumor.[T10b=PD1:aPD1] |
| 396 | Tumor.PD1_mabT + Tumor.[T11b=PD1] <-> Tumor.[T11b=PD1:aPD1] |
| 397 | Tumor.PD1_mabT + Tumor.[T12b=PD1] <-> Tumor.[T12b=PD1:aPD1] |
| 398 | Tumor.PD1_mabT + Tumor.[T14b=PD1] <-> Tumor.[T14b=PD1:aPD1] |
| 399 | Tumor.PD1_mabT + Tumor.[T7b=PD1] <-> Tumor.[T7b=PD1:aPD1] |
| 400 | Tumor.PD1_mabT + Tumor.[T8a=PD1] <-> Tumor.[T8a=PD1:aPD1] |
| 401 | Tumor.PD1_mabT + Tumor.PD1_TeffT <-> Tumor.[PD1:aPD1_Teff] |
| 402 | Tumor.PD1_MDSCsT -> null |
| 403 | Tumor.PD1_MDSCsT + Tumor.PD1_mabT <-> Tumor.[PD1:aPD1_MDSCs] |
| 404 | Tumor.PD1_MDSCsT + Tumor.PDL1_TeffT1 <-> Tumor.[PD1:PDL1_MDSCT] |
| 405 | Tumor.PD1_TeffT -> null |
| 406 | Tumor.PD1_TeffT + Tumor.PDL1_TregT <-> Tumor.[PDL1:PD1_TrTeff] |
| 407 | Tumor.PD1_TeffT1 -> null |
| 408 | Tumor.PD1_TeffT1 + Tumor.PD1_mabT <-> Tumor.[PD1:aPD1_Teff1] |
| 409 | Tumor.PD1_TregT -> null |
| 410 | Tumor.PD1_TregT + Tumor.PD1_mabT <-> Tumor.[PD1:aPD1_Treg] |
| 411 | Tumor.PDL1_mabT -> null |
| 412 | Tumor.PDL1_mabT + Tumor.[C3=PDL1] <-> Tumor.[C3=PDL1:aPDL1] |
| 413 | Tumor.PDL1_mabT + Tumor.[T13a=PDL1] <-> Tumor.[T13a=PDL1:aPDL1] |
| 414 | Tumor.PDL1_mabT + Tumor.[T2=PDL1] <-> Tumor.[T2=PDL1:aPDL1] |
| 415 | Tumor.PDL1_mabT + Tumor.[T7a=PDL1] <-> Tumor.[T7a=PDL1:aPDL1] |
| 416 | Tumor.PDL1_mabT + Tumor.PDL1_TeffT <-> Tumor.[PDL1:aPDL1_Teff] |
| 417 | Tumor.PDL1_mabT + Tumor.PDL1_TeffT1 <-> Tumor.[PDL1:aPDL1_Teff1] |
| 418 | Tumor.PDL1_MDSCsT -> null |
| 419 | Tumor.PDL1_MDSCsT + Tumor.CD80_TeffT1 <-> Tumor.[PDL1:CD80_MDSCT] |
| 420 | Tumor.PDL1_MDSCsT + Tumor.PD1_TeffT1 <-> Tumor.[PDL1:PD1_MDSCT] |
| 421 | Tumor.PDL1_MDSCsT + Tumor.PDL1_mabT <-> Tumor.[PDL1:aPDL1_MDSCs] |
| 422 | Tumor.PDL1_TeffT -> null |
| 423 | Tumor.PDL1_TeffT + Tumor.PD1_TregT <-> Tumor.[PD1:PDL1_TrTeff] |
| 424 | Tumor.PDL1_TeffT1 -> null |
| 425 | Tumor.PDL1_TregT -> null |
| 426 | Tumor.PDL1_TregT + Tumor.PDL1_mabT <-> Tumor.[PDL1:aPDL1_Treg] |
| 427 | Tumor.TC1 -> null |
| 428 | Tumor.TC1 + Tumor.Cancer -> 19 Tumor.C_DebrisT |
| 429 | Tumor.TC1 + Tumor.Effector_TT -> Tumor.Exhausted_TT |
| 430 | Tumor.Teff_EngMDSC -> Tumor.Teff_EngMDSC + Tumor.CD80_TeffT1 |
| 431 | Tumor.Teff_EngMDSC -> Tumor.Teff_EngMDSC + Tumor.PD1_TeffT1 |
| 432 | Tumor.Teff_EngMDSC -> Tumor.Teff_EngMDSC + Tumor.PDL1_TeffT1 |
| 433 | Tumor.Teff_EngTregT -> Tumor.CD80_TeffT + Tumor.Teff_EngTregT |
| 434 | Tumor.Teff_EngTregT -> Tumor.PD1_TeffT + Tumor.Teff_EngTregT |
| 435 | Tumor.Teff_EngTregT -> Tumor.PDL1_TeffT + Tumor.Teff_EngTregT |
| 436 | Tumor.TregT + Tumor.Effector_TT_TregT -> Tumor.TregT_Teff + Tumor.Effector_TT_TregT + Tumor.TregT |
| 437 | Tumor.TregT + Tumor.mAPC_T_TregT -> Tumor.TregT_mAPCT + Tumor.mAPC_T_TregT + Tumor.TregT |
| 438 | Tumor.TregT_EngAPC -> Tumor.CTLA4_TregT + Tumor.TregT_EngAPC |
| 439 | Tumor.TregT_EngTeff -> Tumor.PD1_TregT + Tumor.TregT_EngTeff |
| 440 | Tumor.TregT_EngTeff -> Tumor.PDL1_TregT + Tumor.TregT_EngTeff |
| 441 | Tumor.TregT_mAPCT -> null |
| 442 | Tumor.TregT_mAPCT + Tumor.mAPC_T -> null |
| 443 | Tumor.TregT_Teff -> null |
| 444 | Tumor.TregT_Teff + Tumor.Effector_TT -> null |
| 445 | Tumor.TregT1 -> Tumor.TregT1 + Tumor.CTLA4_TregTS |

**Table S4 – Model Reactions (End)**

**Table S5 – Model Reaction Rates (Start)**

| Reaction Number | Reaction Rate |
| --- | --- |
| 1 | Cl_CTLA4*[Blood-Lymph].CTLA4_mabB |
| 2 | k_Ab_BLN*SA_BP/K_B_CTLA4*[Blood-Lymph].CTLA4_mabB*Vtdln*Num_TDLN_Considered |
| 3 | (k_CTLA4_BP*SA_BP/K_B_CTLA4)*[Blood-Lymph].CTLA4_mabB*Peripheral-k_CTLA4_BP*SA_BP/K_P_CTLA4*Peripheral.CTLA4_mabP*Peripheral |
| 4 | (k_Ab_BT*SA_BT/K_B_CTLA4)*[Blood-Lymph].CTLA4_mabB*Tumor-k_Ab_BT*SA_BT/K_T*Tumor.CTLA4_mabT*Tumor |
| 5 | EffT_Migrate*[Blood-Lymph].Effector_T_TB |
| 6 | ((Q_P/Vv_P)*[Blood-Lymph].Effector_TB-((Q_P-k_Lp)/Vv_P)*Peripheral.Effector_Tf) |
| 7 | (Q_T/Vv_T)*[Blood-Lymph].Effector_TB-((Q_T-k_Lt*Tumor)/Vv_T)*Tumor.Effector_Tf |
| 8 | Cl_PD1*[Blood-Lymph].PD1_mabB |
| 9 | k_Ab_BLN*SA_BP/K_B_PD1*[Blood-Lymph].PD1_mabB*Vtdln*Num_TDLN_Considered |
| 10 | (k_PD1_BP*SA_BP/K_B_PD1)*[Blood-Lymph].PD1_mabB*Peripheral-k_PD1_BP*SA_BP/K_P_PD1*Peripheral.PD1_mabP*Peripheral |
| 11 | (k_Ab_BT*SA_BT/K_B_PD1)*[Blood-Lymph].PD1_mabB*Tumor-k_Ab_BT*SA_BT/K_T*Tumor.PD1_mabT*Tumor |
| 12 | Cl_PDL1*[Blood-Lymph].PDL1_mabB |
| 13 | k_Ab_BLN*SA_BP/K_B_PDL1*[Blood-Lymph].PDL1_mabB*Vtdln*Num_TDLN_Considered |
| 14 | (k_PDL1_BP*SA_BP/K_B_PDL1)*[Blood-Lymph].PDL1_mabB*Peripheral-k_PDL1_BP*SA_BP/K_P_PDL1*Peripheral.PDL1_mabP*Peripheral |
| 15 | (k_Ab_BT*SA_BT/K_B_PDL1)*[Blood-Lymph].PDL1_mabB*Tumor-k_Ab_BT*SA_BT/K_T*Tumor.PDL1_mabT*Tumor |
| 16 | [Exp_CD28/80/86/PD1/L1/L2]*(Lymph_Node.[PNT-Tr_CD80]+[TrPNT_PDL1-CD80]) |
| 17 | [kon_PDL1_CD80]*Lymph_Node.[PNT-Tr_CD80]*Lymph_Node.[Tr-PNT_PDL1]/[Vol_Cell-Rec_Tr-PNT] - [koff_PDL1_CD80]*Lymph_Node.[TrPNT_PDL1-CD80] |
| 18 | [Exp_CD28/80/86/PD1/L1/L2]*(Lymph_Node.[PNT-Tr_PD1]+PNTTr_PD1_aPD1+[TrPNT_PDL1-PD1]) |
| 19 | [kon_PD1_PDL1]*Lymph_Node.[PNT-Tr_PD1]*Lymph_Node.[Tr-PNT_PDL1]/[Vol_Cell-Rec_Tr-PNT] - [koff_PD1_PDL1]*Lymph_Node.[TrPNT_PDL1-PD1] |
| 20 | [kon_PD1-PD1mAb]*Lymph_Node.[PNT-Tr_PD1]*Lymph_Node.PD1_mab - [koff_PD1-PD1mAb]*Lymph_Node.PNTTr_PD1_aPD1 |
| 21 | [Exp_CD28/80/86/PD1/L1/L2]*(Lymph_Node.[PNT-Tr_PDL1]+PNTTr_PDL1_aPDL1+[TrPNT_PD1-L1]) |
| 22 | [kon_PDL1-PDL1mAb]*Lymph_Node.[PNT-Tr_PDL1]*Lymph_Node.PDL1_mab - [koff_PDL1-PDL1mAb]*Lymph_Node.PNTTr_PDL1_aPDL1 |
| 23 | [Exp_CD28/80/86/PD1/L1/L2]*(Lymph_Node.[Tr-mAPC_CD80]+TrALN_CT_CD80) |
| 24 | [Exp_CD28/80/86/PD1/L1/L2]*(Lymph_Node.[Tr-mAPC_CD86]+TrALN_CT_CD86) |
| 25 | [Exp_CTLA4]*(Lymph_Node.[Tr-mAPC_CTLA4]+[TrALN_CT_aCT]+[TrALN_CT_CD80]+[TrALN_CT_CD86]) |
| 26 | [kon_CTLA4_CD80]*Lymph_Node.[Tr-mAPC_CD80]*Lymph_Node.[Tr-mAPC_CTLA4]/[Vol_Cell-Rec_Tr-mAPC] - [koff_CTLA4_CD80]*Lymph_Node.TrALN_CT_CD80 |
| 27 | [kon_CTLA4_CD86]*Lymph_Node.[Tr-mAPC_CD86]*Lymph_Node.[Tr-mAPC_CTLA4]/[Vol_Cell-Rec_Tr-mAPC]-[koff_CTLA4_CD86]*Lymph_Node.TrALN_CT_CD86 |
| 28 | kon_CTLA4mAb_CTLA4*Lymph_Node.[Tr-mAPC_CTLA4]*Lymph_Node.CTLA4_mab - koff_CTLA4mAb_CTLA4*Lymph_Node.TrALN_CT_aCT |
| 29 | [Exp_CD28/80/86/PD1/L1/L2]*(Lymph_Node.[Tr-PNT_PD1]+TrPNT_PD1_aPD1+[TrPNT_PD1-L1]) |
| 30 | [kon_PD1_PDL1]*Lymph_Node.[Tr-PNT_PD1]*Lymph_Node.[PNT-Tr_PDL1]/[Vol_Cell-Rec_Tr-PNT] - [koff_PD1_PDL1]*Lymph_Node.[TrPNT_PD1-L1] |
| 31 | [kon_PD1-PD1mAb]*Lymph_Node.[Tr-PNT_PD1]*Lymph_Node.PD1_mab - [koff_PD1-PD1mAb]*Lymph_Node.TrPNT_PD1_aPD1 |
| 32 | [Exp_CD28/80/86/PD1/L1/L2]*(Lymph_Node.[Tr-PNT_PDL1]+TrPNT_PDL1_aPDL1+[TrPNT_PDL1-PD1]+[TrPNT_PDL1-CD80]) |
| 33 | [kon_PDL1-PDL1mAb]*Lymph_Node.[Tr-PNT_PDL1]*Lymph_Node.PDL1_mab - [koff_PDL1-PDL1mAb]*Lymph_Node.TrPNT_PDL1_aPDL1 |
| 34 | [Treg:T_IntTime]*Lymph_Node.[TregLN-NT] |
| 35 | [Treg:T_IntTime]*Lymph_Node.[TregLN-NT] |
| 36 | [Treg:T_IntTime]*Lymph_Node.[TregLN-PNT]*(1-[Sig_TrPNT=PD1/L1/CD80]) |
| 37 | [Treg:T_IntTime]*Lymph_Node.[TregLN-PNT]*[Sig_TrPNT=PD1/L1/CD80] |
| 38 | [Exp_CD28/80/86/PD1/L1/L2]*Lymph_Node.[TregLN-PNT1]*(([%CD80_receptor_level_PNT]*[CD80_receptors-per-Tcell])/[Avagadro's_Num])*(1/Tr_per_T_cell) |
| 39 | [Exp_CD28/80/86/PD1/L1/L2]*Lymph_Node.[TregLN-PNT1]*(([%PD1_receptor_level_PNT]*[PD1_receptors-per-Tcell])/[Avagadro's_Num])*(1/Tr_per_T_cell) |
| 40 | [Exp_CD28/80/86/PD1/L1/L2]*Lymph_Node.[TregLN-PNT1]*(([%PDL1_receptor_level_PNT]*[PDL1_receptors-per-Tcell])/[Avagadro's_Num])*(1/Tr_per_T_cell) |
| 41 | [Exp_CD28/80/86/PD1/L1/L2]*Lymph_Node.[TregLN-PNT1]*([PD1_receptors-per-Tcell]/[Avagadro's_Num])*(1/T_per_Tr_cell) |
| 42 | [Exp_CD28/80/86/PD1/L1/L2]*Lymph_Node.[TregLN-PNT1]*([PDL1_receptors-per-Tcell]/[Avagadro's_Num])*(1/T_per_Tr_cell) |
| 43 | Phago_Debris*Lymph_Node.APCLN*Lymph_Node.C_DebrisLN |
| 44 | Debris_Decay*Lymph_Node.C_DebrisLN |
| 45 | (k_Lt*Tumor/Vtdln/K_LN/Num_TDLN_Considered+k_Ab_BLN*SA_BP/K_LN)*Vtdln*Lymph_Node.CTLA4_mab |
| 46 | kon_CTLA4mAb_CTLA4*Lymph_Node.CTLA4_mab*Lymph_Node.PNT_CTLA4-koff_CTLA4mAb_CTLA4*Lymph_Node.CTLA4_mAb_CTLA4 |
| 47 | Endo_CTLA4*Lymph_Node.CTLA4_mAb_CTLA4 |
| 48 | EffT_Migrate*Lymph_Node.Effector_T |
| 49 | Exp_All_mAPCLN*(Lymph_Node.mAPC*([CD80_receptors-per-mAPC]/[Avagadro's_Num])) |
| 50 | Exp_All_mAPCLN*(Lymph_Node.mAPC*([CD86_receptors-per-mAPC]/[Avagadro's_Num])) |
| 51 | Exp_All_mAPCLN*Lymph_Node.mAPC*([PD1_receptors-per-mAPC]/[Avagadro's_Num]) |
| 52 | Exp_All_mAPCLN*Lymph_Node.mAPC*([PDL1_receptors-per-mAPC]/[Avagadro's_Num]) |
| 53 | Exp_All_mAPCLN*Lymph_Node.mAPC*([PDL2_receptors-per-mAPC]/[Avagadro's_Num]) |
| 54 | kf_APC_turnover*Lymph_Node.mAPC |
| 55 | kf_TregLNS_Inact*Lymph_Node.mAPC*CTLA4Sig_Secrete |
| 56 | Exp_All_mAPCLN*(Lymph_Node.mAPC_CD80) |
| 57 | Exp_All_mAPCLN*(Lymph_Node.mAPC_CD86) |
| 58 | [Exp_CD28/80/86/PD1/L1/L2]*((Lymph_Node.mAPC_CD80)*(Lymph_Node.mAPC_Int_P1/(Lymph_Node.mAPC+ 1E-100*mole)))*(1/T_cells_per_mAPC) |
| 59 | [Exp_CD28/80/86/PD1/L1/L2]*(Lymph_Node.mAPC_CD86*(Lymph_Node.mAPC_Int_P1/(Lymph_Node.mAPC+ 1E-100*mole)))*(1/T_cells_per_mAPC) |
| 60 | [Exp_CD28/80/86/PD1/L1/L2]*(Lymph_Node.mAPC_Int_P1_CD80 + POS_Sig_NT_CD80) |
| 61 | [Exp_CD28/80/86/PD1/L1/L2]*(Lymph_Node.mAPC_Int_P1_CD86 + POS_Sig_NT_CD86) |
| 62 | [Exp_CD28/80/86/PD1/L1/L2]*((Lymph_Node.mAPC_CD80)*(Lymph_Node.mAPC_Int_P2/(Lymph_Node.mAPC+1E-100*mole)))*(1/T_cells_per_mAPC) |
| 63 | [Exp_CD28/80/86/PD1/L1/L2]*(Lymph_Node.mAPC_CD86*(Lymph_Node.mAPC_Int_P2/(Lymph_Node.mAPC+1E-100*mole)))*(1/T_cells_per_mAPC) |
| 64 | [Exp_CD28/80/86/PD1/L1/L2]*Lymph_Node.mAPC_PD1*(Lymph_Node.mAPC_Int_P2/(Lymph_Node.mAPC+1E-100*mole))*(1/T_cells_per_mAPC) |
| 65 | [Exp_CD28/80/86/PD1/L1/L2]*Lymph_Node.mAPC_PDL1*(Lymph_Node.mAPC_Int_P2/(Lymph_Node.mAPC+1E-100*mole))*(1/T_cells_per_mAPC) |
| 66 | [Exp_CD28/80/86/PD1/L1/L2]*Lymph_Node.mAPC_PDL2*(Lymph_Node.mAPC_Int_P2/(Lymph_Node.mAPC+1E-100*mole))*(1/T_cells_per_mAPC) |
| 67 | [Exp_CD28/80/86/PD1/L1/L2]*(Lymph_Node.mAPC_Int_P2_CD80+NEG_Sig_PNT_CD80+POS_Sig_PNT_CD80+[PNT_PDL1-CD80]) |
| 68 | [kon_CTLA4_CD80]*Lymph_Node.mAPC_Int_P2_CD80*Lymph_Node.PNT_CTLA4/([Volume_PNT-Receptor_Int])-[koff_CTLA4_CD80]*Lymph_Node.NEG_Sig_PNT_CD80 |
| 69 | [kon_PDL1_CD80]*Lymph_Node.mAPC_Int_P2_CD80*Lymph_Node.PNT_Int_PDL1/[Volume_PNT-Receptor_Int]-[koff_PDL1_CD80]*Lymph_Node.[PNT_PDL1-CD80] |
| 70 | [Exp_CD28/80/86/PD1/L1/L2]*(Lymph_Node.mAPC_Int_P2_CD86+NEG_Sig_PNT_CD86+POS_Sig_PNT_CD86) |
| 71 | [kon_CTLA4_CD86]*Lymph_Node.mAPC_Int_P2_CD86*Lymph_Node.PNT_CTLA4/([Volume_PNT-Receptor_Int])-[koff_CTLA4_CD86]*Lymph_Node.NEG_Sig_PNT_CD86 |
| 72 | [kon_CD28_CD86]*Lymph_Node.mAPC_Int_P2_CD86*Lymph_Node.PNT_Int_CD28/([Volume_PNT-Receptor_Int])-[koff_CD28_CD86]*Lymph_Node.POS_Sig_PNT_CD86 |
| 73 | [Exp_CD28/80/86/PD1/L1/L2]*(Lymph_Node.mAPC_Int_P2_PD1+PD1mAb_mAPC_PD1+[PNT_PDL1-PD1]) |
| 74 | [kon_PD1-PD1mAb]*Lymph_Node.mAPC_Int_P2_PD1*Lymph_Node.PD1_mab-[koff_PD1-PD1mAb]*[PD1mAb_mAPC_PD1] |
| 75 | [Exp_CD28/80/86/PD1/L1/L2]*(Lymph_Node.mAPC_Int_P2_PDL1+[PDL1mAb-mAPC_PDL1]+[PNT_PD1-PDL1]+[PNT_CD80-PDL1]) |
| 76 | [Exp_CD28/80/86/PD1/L1/L2]*(Lymph_Node.mAPC_Int_P2_PDL2+[PNT_PD1-PDL2]) |
| 77 | [kon_PD1_PDL2]*Lymph_Node.mAPC_Int_P2_PDL2*Lymph_Node.PNT_Int_PD1/[Volume_PNT-Receptor_Int]-[koff_PD1_PDL2]*Lymph_Node.[PNT_PD1-PDL2] |
| 78 | Exp_All_mAPCLN*((Lymph_Node.mAPC_CD80)*(Lymph_Node.mAPC_nInt/(Lymph_Node.mAPC+ 1E-100*mole))) |
| 79 | Exp_All_mAPCLN*((Lymph_Node.mAPC_CD86)*(Lymph_Node.mAPC_nInt/(Lymph_Node.mAPC+ 1E-100*mole))) |
| 80 | Exp_All_mAPCLN*(Lymph_Node.mAPC_nInt_CD80+CTLA4S_CD80) |
| 81 | [kon_CTLA4_CD80]*Lymph_Node.mAPC_nInt_CD80*Lymph_Node.TrLN_CTLA4S/Vtdln -[koff_CTLA4_CD80]*Lymph_Node.CTLA4S_CD80 |
| 82 | Exp_All_mAPCLN*(Lymph_Node.mAPC_nInt_CD86+CTLA4S_CD86) |
| 83 | [kon_CTLA4_CD86]*Lymph_Node.mAPC_nInt_CD86*Lymph_Node.TrLN_CTLA4S/Vtdln - [koff_CTLA4_CD86]*Lymph_Node.CTLA4S_CD86 |
| 84 | Exp_All_mAPCLN*Lymph_Node.mAPC_PD1 |
| 85 | Exp_All_mAPCLN*Lymph_Node.mAPC_PDL1 |
| 86 | Exp_All_mAPCLN*Lymph_Node.mAPC_PDL2 |
| 87 | PrimeNT_rate*Lymph_Node.Naive_T*[Prob_NT-mAPC_Interact]*[Sig_NT=CD28] |
| 88 | EffT_InOutLN*Lymph_Node.Naive_T |
| 89 | PrimeNT1_rate*Lymph_Node.Naive_T1*(1-Antigen_Intensity)*((mAPC_Int_P1)/(mAPC_Int_P1+Naive_T1*mAPC50_per_T_cell+0.001*mole)) |
| 90 | PrimeNT1_rate*Lymph_Node.Naive_T1*Antigen_Intensity*((mAPC_Int_P1)/(mAPC_Int_P1+Naive_T1*mAPC50_per_T_cell+0.001*mole)) |
| 91 | Endo_CTLA4*Lymph_Node.NEG_Sig_PNT_CD80 |
| 92 | Endo_CTLA4*Lymph_Node.NEG_Sig_PNT_CD86 |
| 93 | [Exp_CD28/80/86/PD1/L1/L2]*(Lymph_Node.NT_Int_CD28+POS_Sig_NT_CD80+POS_Sig_NT_CD86) |
| 94 | [kon_CD28_CD80]*Lymph_Node.NT_Int_CD28*Lymph_Node.mAPC_Int_P1_CD80/[Volume_NT-Receptor_Int] - [koff_CD28_CD80]*Lymph_Node.POS_Sig_NT_CD80 |
| 95 | [kon_CD28_CD86]*Lymph_Node.NT_Int_CD28*Lymph_Node.mAPC_Int_P1_CD86/[Volume_NT-Receptor_Int] - [koff_CD28_CD86]*Lymph_Node.POS_Sig_NT_CD86 |
| 96 | [Exp_CD28/80/86/PD1/L1/L2]*(([CD28_receptors-per-Tcell]/[Avagadro's_Num]))*(mAPC_Int_P1/(mAPC_per_T_cell)) |
| 97 | (k_Lt*Tumor/Vtdln/K_LN/Num_TDLN_Considered+k_Ab_BLN*SA_BP/K_LN)*Vtdln*Lymph_Node.PD1_mab |
| 98 | (k_Lt*Tumor/Vtdln/K_LN/Num_TDLN_Considered+k_Ab_BLN*SA_BP/K_LN)*Vtdln*Lymph_Node.PDL1_mab |
| 99 | [kon_PDL1-PDL1mAb]*Lymph_Node.PDL1_mab*Lymph_Node.mAPC_Int_P2_PDL1 - [koff_PDL1-PDL1mAb]*Lymph_Node.[PDL1mAb-mAPC_PDL1] |
| 100 | [kon_PDL1-PDL1mAb]*Lymph_Node.PDL1_mab*Lymph_Node.PNT_Int_PDL1 - [koff_PDL1-PDL1mAb]*Lymph_Node.[PDL1mAb-PNT_PDL1] |
| 101 | [Exp_CTLA4]*(Lymph_Node.PNT_CTLA4+NEG_Sig_PNT_CD80+[NEG_Sig_PNT_CD86]+[CTLA4_mAb_CTLA4]) |
| 102 | [Exp_CD28/80/86/PD1/L1/L2]*(Lymph_Node.PNT_Int_CD28+POS_Sig_PNT_CD80+POS_Sig_PNT_CD86) |
| 103 | [kon_CD28_CD80]*Lymph_Node.PNT_Int_CD28*Lymph_Node.mAPC_Int_P2_CD80/([Volume_PNT-Receptor_Int])-[koff_CD28_CD80]*Lymph_Node.POS_Sig_PNT_CD80 |
| 104 | [Exp_CD28/80/86/PD1/L1/L2]*(Lymph_Node.PNT_Int_CD80+[PNT_CD80-PDL1]) |
| 105 | [kon_PDL1_CD80]*Lymph_Node.PNT_Int_CD80*Lymph_Node.mAPC_Int_P2_PDL1/[Volume_PNT-Receptor_Int]-[koff_PDL1_CD80]*Lymph_Node.[PNT_CD80-PDL1] |
| 106 | [Exp_CD28/80/86/PD1/L1/L2]*(Lymph_Node.PNT_Int_PD1+PD1mAb_PNT_PD1+[PNT_PD1-PDL2]+[PNT_PD1-PDL1]) |
| 107 | [kon_PD1_PDL1]*Lymph_Node.PNT_Int_PD1*Lymph_Node.mAPC_Int_P2_PDL1/[Volume_PNT-Receptor_Int]-[koff_PD1_PDL1]*Lymph_Node.[PNT_PD1-PDL1] |
| 108 | [kon_PD1-PD1mAb]*Lymph_Node.PNT_Int_PD1*Lymph_Node.PD1_mab-[koff_PD1-PD1mAb]*[PD1mAb_PNT_PD1] |
| 109 | [Exp_CD28/80/86/PD1/L1/L2]*(Lymph_Node.PNT_Int_PDL1+[PDL1mAb-PNT_PDL1]+[PNT_PDL1-CD80]+[PNT_PDL1-PD1]) |
| 110 | [kon_PD1_PDL1]*Lymph_Node.PNT_Int_PDL1*Lymph_Node.mAPC_Int_P2_PD1/[Volume_PNT-Receptor_Int]-[koff_PD1_PDL1]*Lymph_Node.[PNT_PDL1-PD1] |
| 111 | [Exp_CTLA4]*(([CTLA4_receptors-Int-PNT]/[Avagadro's_Num]))*(mAPC_Int_P2/(mAPC_per_T_cell)) |
| 112 | [Exp_CD28/80/86/PD1/L1/L2]*(([CD28_receptors-per-Tcell]/[Avagadro's_Num]))*(mAPC_Int_P2/(mAPC_per_T_cell)) |
| 113 | [Exp_CD28/80/86/PD1/L1/L2]*((([%CD80_receptor_level_PNT]*[CD80_receptors-per-Tcell])/[Avagadro's_Num]))*(mAPC_Int_P2/(mAPC_per_T_cell)) |
| 114 | [Exp_CD28/80/86/PD1/L1/L2]*((([%PD1_receptor_level_PNT]*[PD1_receptors-per-Tcell])/[Avagadro's_Num]))*(mAPC_Int_P2/(mAPC_per_T_cell)) |
| 115 | [Exp_CD28/80/86/PD1/L1/L2]*((([%PDL1_receptor_level_PNT]*[PDL1_receptors-per-Tcell])/[Avagadro's_Num]))*(mAPC_Int_P2/(mAPC_per_T_cell)) |
| 116 | kf_Phase2P*Lymph_Node.Primed_Naive_T*[Prob_PNT-mAPC_Interact] |
| 117 | kf_no_prolif*Lymph_Node.Primed_Naive_T1*[Sig_PNT=CTLA4/PD1/L1/CD80] |
| 118 | PrimeTLN2_rate*Lymph_Node.Primed_Naive_T1*(1-Antigen_Intensity)*((mAPC_Int_P2)/(mAPC_Int_P2+Primed_Naive_T1*mAPC50_per_T_cell+0.001*mole)) |
| 119 | PrimeTLN2_rate*Lymph_Node.Primed_Naive_T1*(1-[Sig_PNT=CTLA4/PD1/L1/CD80])*Antigen_Intensity*((mAPC_Int_P2)/(mAPC_Int_P2 +Primed_Naive_T1* mAPC50_per_T_cell +0.001*mole)) |
| 120 | kf_Prolif_end*Lymph_Node.Prolif_Naive_T |
| 121 | Lymph_Node.Prolif_Naive_T*[Prolif_Thresh] |
| 122 | Endo_CTLA4*Lymph_Node.TrALN_CT_aCT |
| 123 | Endo_CTLA4*Lymph_Node.TrALN_CT_CD80 |
| 124 | Endo_CTLA4*Lymph_Node.TrALN_CT_CD86 |
| 125 | [Exp_CTLA4]*Lymph_Node.TregLN*([CTLA4_receptors-Tr]/[Avagadro's_Num]) |
| 126 | PrimeNT_rate*Lymph_Node.TregLN |
| 127 | TregLN_Engage*Lymph_Node.TregLN_Int*mAPC |
| 128 | TregLN_Engage*Lymph_Node.TregLN_Int*Lymph_Node.Naive_T |
| 129 | TregLN_Engage*Lymph_Node.TregLN_Int*Lymph_Node.Primed_Naive_T |
| 130 | [Treg:mAPC_IntTime]*Lymph_Node.TregLN_mAPC*(1-[CTLA4Sig_TrLN-mAPC]) |
| 131 | [Treg:mAPC_IntTime]*Lymph_Node.TregLN_mAPC*[CTLA4Sig_TrLN-mAPC] |
| 132 | [Exp_CD28/80/86/PD1/L1/L2]*Lymph_Node.TregLN_mAPC1*([CD80_receptors-per-mAPC]/[Avagadro's_Num])*(1/Tr_cells_per_mAPC) |
| 133 | [Exp_CD28/80/86/PD1/L1/L2]*Lymph_Node.TregLN_mAPC1*([CD86_receptors-per-mAPC]/[Avagadro's_Num])*(1/Tr_cells_per_mAPC) |
| 134 | [Exp_CTLA4]*Lymph_Node.TregLN_mAPC1*([CTLA4_receptors-Tr]/[Avagadro's_Num])*(1/mAPC_per_Tr_cell) |
| 135 | [CTLA4S_Molec_per_Sec_TregLN]*(Lymph_Node.TregLN_Secrete/[Avagadro's_Num])*( [CTLA4Sig_Secrete_max]-[TrLN_CTLA4S])/(1E-100*mole+[CTLA4Sig_Secrete_max]) |
| 136 | [Exp_CTLA4]*(Lymph_Node.TrLN_CTLA4+[TrLN_CT_aCT]) |
| 137 | kon_CTLA4mAb_CTLA4*Lymph_Node.TrLN_CTLA4*Lymph_Node.CTLA4_mab - koff_CTLA4mAb_CTLA4*Lymph_Node.TrLN_CT_aCT |
| 138 | kon_CTLA4mAb_CTLA4*Lymph_Node.TrLN_CTLA4S*Lymph_Node.CTLA4_mab-koff_CTLA4mAb_CTLA4*Lymph_Node.TrLN_CTLA4S_aCTLA4 |
| 139 | (k__DoseAdmin_AntiCTLA4*[BodyWeight (kg)]*CTLA4mAb)/(Tremelimumab_MW)/[Blood-Lymph] |
| 140 | (k_Lt*Tumor/Vtdln/K_LN+k_Ab_BLN*SA_BP/K_LN*Num_TDLN_Considered)*Vtdln*Lymph_Node.CTLA4_mab |
| 141 | (k_Lt*Tumor/Vtdln/K_LN+k_Ab_BLN*SA_BP/K_LN*Num_TDLN_Considered)*Vtdln*Lymph_Node.PD1_mab |
| 142 | (k__DoseAdmin_AntiPD1*[BodyWeight (kg)]*PD1mAb)/(Nivolumab_MW)/[Blood-Lymph] |
| 143 | (k_Lt*Tumor/Vtdln/K_LN+k_Ab_BLN*SA_BP/K_LN*Num_TDLN_Considered)*Vtdln*Lymph_Node.PDL1_mab |
| 144 | (k__DoseAdmin_AntiPDL1*[BodyWeight (kg)]*PDL1mAb)/(Durvalumab_MW)/[Blood-Lymph] |
| 145 | k_Ab_BLN*SA_BP/K_B_CTLA4*[Blood-Lymph].CTLA4_mabB*Vtdln |
| 146 | k_Lt*Tumor/K_T*Tumor.CTLA4_mabT/Num_TDLN_Considered |
| 147 | EffT_InOutLN*Lymph_Node.Naive_T0 |
| 148 | k_Ab_BLN*SA_BP/K_B_PD1*[Blood-Lymph].PD1_mabB*Vtdln |
| 149 | k_Lt*Tumor/K_T*Tumor.PD1_mabT/Num_TDLN_Considered |
| 150 | k_Ab_BLN*SA_BP/K_B_PDL1*[Blood-Lymph].PDL1_mabB*Vtdln |
| 151 | k_Lt*Tumor/K_T*Tumor.PDL1_mabT/Num_TDLN_Considered |
| 152 | Lymph_Node.Prolif_Naive_T*[Prolif_Fract] |
| 153 | kf_Monocytes_intoT*Monocytes |
| 154 | Rate_Tumor_Growth*Tumor.Cancer*(1-Cancer/Cancer_max) |
| 155 | Phi*Tumor.Effector_TT |
| 156 | EffT_Turnover*Peripheral.Effector_Ta |
| 157 | J_P*Peripheral.Effector_Ta |
| 158 | EffT_Turnover*Peripheral.Effector_Tb |
| 159 | AR_P*Peripheral.Effector_Tb |
| 160 | kf_P*(B_P-Peripheral.Effector_Tb/Vv_P-Peripheral.Effector_Ta/Vv_P)*Peripheral.Effector_Tf-kr_P*Peripheral.Effector_Tb |
| 161 | k_Lp/Vi_P*Peripheral.Effector_TP |
| 162 | [Exp_CD28/80/86/PD1/L1/L2]*Tumor.[C{CD80}]*([CD80_receptors_per_C_Cl]/[Avagadro's_Num])*([T_per_Cancer_Cell_Int]/[T_per_Cancer_Cell_max]) |
| 163 | [Exp_CD28/80/86/PD1/L1/L2]*Tumor.[C{PD1}]*([PD1_receptors_per_C_Cl]/[Avagadro's_Num])*([T_per_Cancer_Cell_Int]/[T_per_Cancer_Cell_max]) |
| 164 | [Exp_CD28/80/86/PD1/L1/L2]*Tumor.[C{PD1}{CD80}]*([PD1_receptors_per_C_Cl]/[Avagadro's_Num])*([T_per_Cancer_Cell_Int]/[T_per_Cancer_Cell_max]) |
| 165 | [Exp_CD28/80/86/PD1/L1/L2]*Tumor.[C{PD1}{CD80}]*([CD80_receptors_per_C_Cl]/[Avagadro's_Num])*([T_per_Cancer_Cell_Int]/[T_per_Cancer_Cell_max]) |
| 166 | [Exp_CD28/80/86/PD1/L1/L2]*Tumor.[C{PD1}{PDL1}]*([PD1_receptors_per_C_Cl]/[Avagadro's_Num])*([T_per_Cancer_Cell_Int]/[T_per_Cancer_Cell_max]) |
| 167 | [Exp_CD28/80/86/PD1/L1/L2]*Tumor.[C{PD1}{PDL1}]*([PDL1_receptors_per_C_Cl]/[Avagadro's_Num])*([T_per_Cancer_Cell_Int]/[T_per_Cancer_Cell_max]) |
| 168 | [Exp_CD28/80/86/PD1/L1/L2]*Tumor.[C{PD1}{PDL1}{CD80}]*([PD1_receptors_per_C_Cl]/[Avagadro's_Num])*([T_per_Cancer_Cell_Int]/[T_per_Cancer_Cell_max]) |
| 169 | [Exp_CD28/80/86/PD1/L1/L2]*Tumor.[C{PD1}{PDL1}{CD80}]*([PDL1_receptors_per_C_Cl]/[Avagadro's_Num])*([T_per_Cancer_Cell_Int]/[T_per_Cancer_Cell_max]) |
| 170 | [Exp_CD28/80/86/PD1/L1/L2]*Tumor.[C{PD1}{PDL1}{CD80}]*([CD80_receptors_per_C_Cl]/[Avagadro's_Num])*([T_per_Cancer_Cell_Int]/[T_per_Cancer_Cell_max]) |
| 171 | [Exp_CD28/80/86/PD1/L1/L2]*Tumor.[C{PD1}{PDL1}{PDL2}]*([PD1_receptors_per_C_Cl]/[Avagadro's_Num])*([T_per_Cancer_Cell_Int]/[T_per_Cancer_Cell_max]) |
| 172 | [Exp_CD28/80/86/PD1/L1/L2]*Tumor.[C{PD1}{PDL1}{PDL2}]*([PDL1_receptors_per_C_Cl]/[Avagadro's_Num])*([T_per_Cancer_Cell_Int]/[T_per_Cancer_Cell_max]) |
| 173 | [Exp_CD28/80/86/PD1/L1/L2]*Tumor.[C{PD1}{PDL1}{PDL2}]*([PDL2_receptors_per_C_Cl]/[Avagadro's_Num])*([T_per_Cancer_Cell_Int]/[T_per_Cancer_Cell_max]) |
| 174 | [Exp_CD28/80/86/PD1/L1/L2]*Tumor.[C{PD1}{PDL1}{PDL2}{CD80}]*([PD1_receptors_per_C_Cl]/[Avagadro's_Num])*([T_per_Cancer_Cell_Int]/[T_per_Cancer_Cell_max]) |
| 175 | [Exp_CD28/80/86/PD1/L1/L2]*Tumor.[C{PD1}{PDL1}{PDL2}{CD80}]*([PDL1_receptors_per_C_Cl]/[Avagadro's_Num])*([T_per_Cancer_Cell_Int]/[T_per_Cancer_Cell_max]) |
| 176 | [Exp_CD28/80/86/PD1/L1/L2]*Tumor.[C{PD1}{PDL1}{PDL2}{CD80}]*([PDL2_receptors_per_C_Cl]/[Avagadro's_Num])*([T_per_Cancer_Cell_Int]/[T_per_Cancer_Cell_max]) |
| 177 | [Exp_CD28/80/86/PD1/L1/L2]*Tumor.[C{PD1}{PDL1}{PDL2}{CD80}]*([CD80_receptors_per_C_Cl]/[Avagadro's_Num])*([T_per_Cancer_Cell_Int]/[T_per_Cancer_Cell_max]) |
| 178 | [Exp_CD28/80/86/PD1/L1/L2]*Tumor.[C{PD1}{PDL2}]*([PD1_receptors_per_C_Cl]/[Avagadro's_Num])*([T_per_Cancer_Cell_Int]/[T_per_Cancer_Cell_max]) |
| 179 | [Exp_CD28/80/86/PD1/L1/L2]*Tumor.[C{PD1}{PDL2}]*([PDL2_receptors_per_C_Cl]/[Avagadro's_Num])*([T_per_Cancer_Cell_Int]/[T_per_Cancer_Cell_max]) |
| 180 | [Exp_CD28/80/86/PD1/L1/L2]*Tumor.[C{PD1}{PDL2}{CD80}]*([PD1_receptors_per_C_Cl]/[Avagadro's_Num])*([T_per_Cancer_Cell_Int]/[T_per_Cancer_Cell_max]) |
| 181 | [Exp_CD28/80/86/PD1/L1/L2]*Tumor.[C{PD1}{PDL2}{CD80}]*([PDL2_receptors_per_C_Cl]/[Avagadro's_Num])*([T_per_Cancer_Cell_Int]/[T_per_Cancer_Cell_max]) |
| 182 | [Exp_CD28/80/86/PD1/L1/L2]*Tumor.[C{PD1}{PDL2}{CD80}]*([CD80_receptors_per_C_Cl]/[Avagadro's_Num])*([T_per_Cancer_Cell_Int]/[T_per_Cancer_Cell_max]) |
| 183 | [Exp_CD28/80/86/PD1/L1/L2]*Tumor.[C{PDL1}]*([PDL1_receptors_per_C_Cl]/[Avagadro's_Num])*([T_per_Cancer_Cell_Int]/[T_per_Cancer_Cell_max]) |
| 184 | [Exp_CD28/80/86/PD1/L1/L2]*Tumor.[C{PDL1}{CD80}]*([PDL1_receptors_per_C_Cl]/[Avagadro's_Num])*([T_per_Cancer_Cell_Int]/[T_per_Cancer_Cell_max]) |
| 185 | [Exp_CD28/80/86/PD1/L1/L2]*Tumor.[C{PDL1}{CD80}]*([CD80_receptors_per_C_Cl]/[Avagadro's_Num])*([T_per_Cancer_Cell_Int]/[T_per_Cancer_Cell_max]) |
| 186 | [Exp_CD28/80/86/PD1/L1/L2]*Tumor.[C{PDL1}{PDL2}]*([PDL1_receptors_per_C_Cl]/[Avagadro's_Num])*([T_per_Cancer_Cell_Int]/[T_per_Cancer_Cell_max]) |
| 187 | [Exp_CD28/80/86/PD1/L1/L2]*Tumor.[C{PDL1}{PDL2}]*([PDL2_receptors_per_C_Cl]/[Avagadro's_Num])*([T_per_Cancer_Cell_Int]/[T_per_Cancer_Cell_max]) |
| 188 | [Exp_CD28/80/86/PD1/L1/L2]*Tumor.[C{PDL1}{PDL2}{CD80}]*([PDL1_receptors_per_C_Cl]/[Avagadro's_Num])*([T_per_Cancer_Cell_Int]/[T_per_Cancer_Cell_max]) |
| 189 | [Exp_CD28/80/86/PD1/L1/L2]*Tumor.[C{PDL1}{PDL2}{CD80}]*([PDL2_receptors_per_C_Cl]/[Avagadro's_Num])*([T_per_Cancer_Cell_Int]/[T_per_Cancer_Cell_max]) |
| 190 | [Exp_CD28/80/86/PD1/L1/L2]*Tumor.[C{PDL1}{PDL2}{CD80}]*([CD80_receptors_per_C_Cl]/[Avagadro's_Num])*([T_per_Cancer_Cell_Int]/[T_per_Cancer_Cell_max]) |
| 191 | [Exp_CD28/80/86/PD1/L1/L2]*Tumor.[C{PDL2}]*([PDL2_receptors_per_C_Cl]/[Avagadro's_Num])*([T_per_Cancer_Cell_Int]/[T_per_Cancer_Cell_max]) |
| 192 | [Exp_CD28/80/86/PD1/L1/L2]*Tumor.[C{PDL2}{CD80}]*([PDL2_receptors_per_C_Cl]/[Avagadro's_Num])*([T_per_Cancer_Cell_Int]/[T_per_Cancer_Cell_max]) |
| 193 | [Exp_CD28/80/86/PD1/L1/L2]*Tumor.[C{PDL2}{CD80}]*([CD80_receptors_per_C_Cl]/[Avagadro's_Num])*([T_per_Cancer_Cell_Int]/[T_per_Cancer_Cell_max]) |
| 194 | [Exp_CD28/80/86/PD1/L1/L2]*(Tumor.[C10a=PD1]+[C10a=PD1:aPD1]+[T10a=PDL1:PD1=C10a]) |
| 195 | [kon_PD1-PD1mAb]*Tumor.[C10a=PD1]*Tumor.PD1_mabT-[koff_PD1-PD1mAb]*Tumor.[C10a=PD1:aPD1] |
| 196 | [Exp_CD28/80/86/PD1/L1/L2]*(Tumor.[C10b=PDL1]+[C10b=PDL1:aPDL1]+ [T10b=PD1:PDL1=C10b]+ [T10c=CD80:PDL1=C10b]) |
| 197 | [kon_PDL1-PDL1mAb]*Tumor.[C10b=PDL1]*Tumor.PDL1_mabT-[koff_PDL1-PDL1mAb]*Tumor.[C10b=PDL1:aPDL1] |
| 198 | [Exp_CD28/80/86/PD1/L1/L2]*(Tumor.[C10c=PDL2] + [T10b=PD1:PDL2=C10c]) |
| 199 | [Exp_CD28/80/86/PD1/L1/L2]*(Tumor.[C11a=PDL1]+[C11a=PDL1:aPDL1]+ [T11b=PD1:PDL1=C11a]) |
| 200 | [kon_PDL1-PDL1mAb]*Tumor.[C11a=PDL1]*Tumor.PDL1_mabT-[koff_PDL1-PDL1mAb]*Tumor.[C11a=PDL1:aPDL1] |
| 201 | [Exp_CD28/80/86/PD1/L1/L2]*(Tumor.[C11b=CD80]+ [T11a=PDL1:CD80=C11b]) |
| 202 | [Exp_CD28/80/86/PD1/L1/L2]*(Tumor.[C12a=PDL2]+ [T12b=PD1:PDL2=C12a]) |
| 203 | [Exp_CD28/80/86/PD1/L1/L2]*(Tumor.[C12b=CD80]+ [T12a=PDL1:CD80=C12b]) |
| 204 | [Exp_CD28/80/86/PD1/L1/L2]*(Tumor.[C13a=PDL1]+[C13a=PDL1:aPDL1]+ [T13b=PD1:PDL1=C13a]) |
| 205 | [kon_PDL1-PDL1mAb]*Tumor.[C13a=PDL1]*Tumor.PDL1_mabT-[koff_PDL1-PDL1mAb]*Tumor.[C13a=PDL1:aPDL1] |
| 206 | [Exp_CD28/80/86/PD1/L1/L2]*(Tumor.[C13b=PDL2]+ [T13b=PD1:PDL2=C13b]) |
| 207 | [Exp_CD28/80/86/PD1/L1/L2]*(Tumor.[C13c=CD80]+ [T13a=PDL1:CD80=C13c]) |
| 208 | [Exp_CD28/80/86/PD1/L1/L2]*(Tumor.[C14a=PD1]+[C14a=PD1:aPD1]+ [T14a=PDL1:PD1=C14a]) |
| 209 | [kon_PD1_PDL1]*Tumor.[C14a=PD1]*Tumor.[T14a=PDL1]/[V_T:C_14] -[koff_PD1_PDL1]*Tumor.[T14a=PDL1:PD1=C14a] |
| 210 | [Exp_CD28/80/86/PD1/L1/L2]*(Tumor.[C14b=PDL1]+[C14b=PDL1:aPDL1]+ [T14b=PD1:PDL1=C14b]+ [T14c=CD80:PDL1=C14b]) |
| 211 | [kon_PDL1_CD80]*Tumor.[C14b=PDL1]*Tumor.[T14c=CD80]/[V_T:C_14] - [koff_PDL1_CD80]*Tumor.[T14c=CD80:PDL1=C14b] |
| 212 | [kon_PDL1-PDL1mAb]*Tumor.[C14b=PDL1]*Tumor.PDL1_mabT-[koff_PDL1-PDL1mAb]*Tumor.[C14b=PDL1:aPDL1] |
| 213 | [Exp_CD28/80/86/PD1/L1/L2]*(Tumor.[C14c=CD80]+ [T14a=PDL1:CD80=C14c]) |
| 214 | [kon_PDL1_CD80]*Tumor.[C14c=CD80]*Tumor.[T14a=PDL1]/[V_T:C_14] - [koff_PDL1_CD80]*Tumor.[T14a=PDL1:CD80=C14c] |
| 215 | [Exp_CD28/80/86/PD1/L1/L2]*(Tumor.[C15a=PD1]+[C15a=PD1:aPD1]+ [T15a=PDL1:PD1=C15a]) |
| 216 | [kon_PD1-PD1mAb]*Tumor.[C15a=PD1]*Tumor.PD1_mabT-[koff_PD1-PD1mAb]*Tumor.[C15a=PD1:aPD1] |
| 217 | [Exp_CD28/80/86/PD1/L1/L2]*(Tumor.[C15b=PDL2] + [T15b=PD1:PDL2=C15b]) |
| 218 | [kon_PD1_PDL2]*Tumor.[C15b=PDL2]*Tumor.[T15b=PD1]/[V_T:C_15] - [koff_PD1_PDL2]*Tumor.[T15b=PD1:PDL2=C15b] |
| 219 | [Exp_CD28/80/86/PD1/L1/L2]*(Tumor.[C15c=CD80]+ [T15a=PDL1:CD80=C15c]) |
| 220 | [kon_PDL1_CD80]*Tumor.[C15c=CD80]*Tumor.[T15a=PDL1]/[V_T:C_15] - [koff_PDL1_CD80]*Tumor.[T15a=PDL1:CD80=C15c] |
| 221 | [Exp_CD28/80/86/PD1/L1/L2]*(Tumor.[C16a=PD1]+[C16a=PD1:aPD1]+ [T16a=PDL1:PD1=C16a]) |
| 222 | [kon_PD1_PDL1]*Tumor.[C16a=PD1]*Tumor.[T16a=PDL1]/[V_T:C_16] - [koff_PD1_PDL1]*Tumor.[T16a=PDL1:PD1=C16a] |
| 223 | [kon_PD1-PD1mAb]*Tumor.[C16a=PD1]*Tumor.PD1_mabT-[koff_PD1-PD1mAb]*Tumor.[C16a=PD1:aPD1] |
| 224 | [Exp_CD28/80/86/PD1/L1/L2]*(Tumor.[C16b=PDL1]+[C16b=PDL1:aPDL1]+ [T16c=CD80:PDL1=C16b]+ [T16b=PD1:PDL1=C16b]) |
| 225 | [kon_PDL1-PDL1mAb]*Tumor.[C16b=PDL1]*Tumor.PDL1_mabT-[koff_PDL1-PDL1mAb]*Tumor.[C16b=PDL1:aPDL1] |
| 226 | [Exp_CD28/80/86/PD1/L1/L2]*(Tumor.[C16c=PDL2]+ [T16b=PD1:PDL2=C16c]) |
| 227 | [kon_PD1_PDL2]*Tumor.[C16c=PDL2]*Tumor.[T16b=PD1]/[V_T:C_16] - [koff_PD1_PDL2]*Tumor.[T16b=PD1:PDL2=C16c] |
| 228 | [Exp_CD28/80/86/PD1/L1/L2]*(Tumor.[C16d=CD80]+ [T16a=PDL1:CD80=C16d]) |
| 229 | [Exp_CD28/80/86/PD1/L1/L2]*(Tumor.[C2=PD1]+[C2=PD1:aPD1]+[T2=PDL1:PD1=C2]) |
| 230 | [Exp_CD28/80/86/PD1/L1/L2]*(Tumor.[C3=PDL1]+[C3=PDL1:aPDL1]+[T3a=PD1:PDL1=C3]+[T3b=CD80:PDL1=C3]) |
| 231 | [Exp_CD28/80/86/PD1/L1/L2]*(Tumor.[C4=PDL2]+[T4=PD1:PDL2=C4]) |
| 232 | [Exp_CD28/80/86/PD1/L1/L2]*(Tumor.[C5=CD80]+[T5=PDL1:CD80=C5]) |
| 233 | [Exp_CD28/80/86/PD1/L1/L2]*(Tumor.[C6a=PD1]+[C6a=PD1:aPD1]+[T6a=PDL1:PD1=C6a]) |
| 234 | [kon_PD1-PD1mAb]*Tumor.[C6a=PD1]*Tumor.PD1_mabT-[koff_PD1-PD1mAb]*Tumor.[C6a=PD1:aPD1] |
| 235 | [Exp_CD28/80/86/PD1/L1/L2]*(Tumor.[C6b=PDL1]+[C6b=PDL1:aPDL1]+[T6b=PD1:PDL1=C6b]+[T6c=CD80:PDL1=C6b]) |
| 236 | [kon_PD1_PDL1]*Tumor.[C6b=PDL1]*Tumor.[T6b=PD1]/[V_T:C_6] - [koff_PD1_PDL1]*Tumor.[T6b=PD1:PDL1=C6b] |
| 237 | [kon_PDL1_CD80]*Tumor.[C6b=PDL1]*Tumor.[T6c=CD80]/[V_T:C_6] - [koff_PDL1_CD80]*Tumor.[T6c=CD80:PDL1=C6b] |
| 238 | [kon_PDL1-PDL1mAb]*Tumor.[C6b=PDL1]*Tumor.PDL1_mabT-[koff_PDL1-PDL1mAb]*Tumor.[C6b=PDL1:aPDL1] |
| 239 | [Exp_CD28/80/86/PD1/L1/L2]*(Tumor.[C7a=PD1]+[C7a=PD1:aPD1]+[T7a=PDL1:PD1=C7a]) |
| 240 | [Exp_CD28/80/86/PD1/L1/L2]*(Tumor.[C7b=PDL2]+[T7b=PD1:PDL2=C7b]) |
| 241 | [Exp_CD28/80/86/PD1/L1/L2]*(Tumor.[C8a=PDL1]+[C8a=PDL1:aPDL1]+[T8a=PD1:PDL1=C8a]+[T8b=CD80:PDL1=C8a]) |
| 242 | [kon_PDL1_CD80]*Tumor.[C8a=PDL1]*Tumor.[T8b=CD80]/[V_T:C_8] -[koff_PDL1_CD80]*Tumor.[T8b=CD80:PDL1=C8a] |
| 243 | [kon_PDL1-PDL1mAb]*Tumor.[C8a=PDL1]*Tumor.PDL1_mabT-[koff_PDL1-PDL1mAb]*Tumor.[C8a=PDL1:aPDL1] |
| 244 | [Exp_CD28/80/86/PD1/L1/L2]*(Tumor.[C8b=PDL2]+[T8a=PD1:PDL2=C8b]) |
| 245 | [Exp_CD28/80/86/PD1/L1/L2]*(Tumor.[C9a=PD1]+[C9a=PD1:aPD1]+[T9=PDL1-PD1=C9a]) |
| 246 | [kon_PD1_PDL1]*Tumor.[C9a=PD1]*Tumor.[T9=PDL1]/[V_T:C_9] - [koff_PD1_PDL1]*Tumor.[T9=PDL1-PD1=C9a] |
| 247 | [Exp_CD28/80/86/PD1/L1/L2]*(Tumor.[C9b=CD80]+[T9=PDL1-CD80=C9b]) |
| 248 | Endo_CTLA4*Tumor.[CTLA4_CTLA4-Trt] |
| 249 | Endo_CTLA4*Tumor.[CTLA4:CD80_TrAT] |
| 250 | Endo_CTLA4*Tumor.[CTLA4:CD86_TrAT] |
| 251 | [Exp_CD28/80/86/PD1/L1/L2]*Tumor.[T{PD1}-{PDL2}C]*([PD1_receptors-per-Tcell]/[Avagadro's_Num])*([Cancer_per_T_Cell_Int]/[Cancer_per_T_Cell_max]) |
| 252 | [Exp_CD28/80/86/PD1/L1/L2]*Tumor.[T{PD1}{80}-{PDL1}{PDL2}C]*([PD1_receptors-per-Tcell]/[Avagadro's_Num])*([Cancer_per_T_Cell_Int]/[Cancer_per_T_Cell_max]) |
| 253 | [Exp_CD28/80/86/PD1/L1/L2]*Tumor.[T{PD1}{80}-{PDL1}{PDL2}C]*([CD80_receptors-per-Tcell]/[Avagadro's_Num])*([Cancer_per_T_Cell_Int]/[Cancer_per_T_Cell_max]) |
| 254 | [Exp_CD28/80/86/PD1/L1/L2]*Tumor.[T{PD1}{CD80}-{PDL1}C]*([PD1_receptors-per-Tcell]/[Avagadro's_Num])*([Cancer_per_T_Cell_Int]/[Cancer_per_T_Cell_max]) |
| 255 | [Exp_CD28/80/86/PD1/L1/L2]*Tumor.[T{PD1}{CD80}-{PDL1}C]*([CD80_receptors-per-Tcell]/[Avagadro's_Num])*([Cancer_per_T_Cell_Int]/[Cancer_per_T_Cell_max]) |
| 256 | [Exp_CD28/80/86/PD1/L1/L2]*Tumor.[T{PD1}{L1}-{PD1}{80}{PDL2}C]*([PDL1_receptors-per-Tcell]/[Avagadro's_Num])*([Cancer_per_T_Cell_Int]/[Cancer_per_T_Cell_max]) |
| 257 | [Exp_CD28/80/86/PD1/L1/L2]*Tumor.[T{PD1}{L1}-{PD1}{80}{PDL2}C]*([PD1_receptors-per-Tcell]/[Avagadro's_Num])*([Cancer_per_T_Cell_Int]/[Cancer_per_T_Cell_max]) |
| 258 | [Exp_CD28/80/86/PD1/L1/L2]*Tumor.[T{PD1}{L1}-{PD1}{L2}C]*([PDL1_receptors-per-Tcell]/[Avagadro's_Num])*([Cancer_per_T_Cell_Int]/[Cancer_per_T_Cell_max]) |
| 259 | [Exp_CD28/80/86/PD1/L1/L2]*Tumor.[T{PD1}{L1}-{PD1}{L2}C]*([PD1_receptors-per-Tcell]/[Avagadro's_Num])*([Cancer_per_T_Cell_Int]/[Cancer_per_T_Cell_max]) |
| 260 | [Exp_CD28/80/86/PD1/L1/L2]*Tumor.[T{PD1}{L1}-{PDL1}{80}{L2}C]*([PDL1_receptors-per-Tcell]/[Avagadro's_Num])*([Cancer_per_T_Cell_Int]/[Cancer_per_T_Cell_max]) |
| 261 | [Exp_CD28/80/86/PD1/L1/L2]*Tumor.[T{PD1}{L1}-{PDL1}{80}{L2}C]*([PD1_receptors-per-Tcell]/[Avagadro's_Num])*([Cancer_per_T_Cell_Int]/[Cancer_per_T_Cell_max]) |
| 262 | [Exp_CD28/80/86/PD1/L1/L2]*Tumor.[T{PD1}{L1}-{PDL1}{80}C]*([PDL1_receptors-per-Tcell]/[Avagadro's_Num])*([Cancer_per_T_Cell_Int]/[Cancer_per_T_Cell_max]) |
| 263 | [Exp_CD28/80/86/PD1/L1/L2]*Tumor.[T{PD1}{L1}-{PDL1}{80}C]*([PD1_receptors-per-Tcell]/[Avagadro's_Num])*([Cancer_per_T_Cell_Int]/[Cancer_per_T_Cell_max]) |
| 264 | [Exp_CD28/80/86/PD1/L1/L2]*Tumor.[T{PD1}{L1}-{PDL2}{80}C]*([PDL1_receptors-per-Tcell]/[Avagadro's_Num])*([Cancer_per_T_Cell_Int]/[Cancer_per_T_Cell_max]) |
| 265 | [Exp_CD28/80/86/PD1/L1/L2]*Tumor.[T{PD1}{L1}-{PDL2}{80}C]*([PD1_receptors-per-Tcell]/[Avagadro's_Num])*([Cancer_per_T_Cell_Int]/[Cancer_per_T_Cell_max]) |
| 266 | [Exp_CD28/80/86/PD1/L1/L2]*Tumor.[T{PD1}{L1}{80}-{PD1}{80}{L1}{L2}C]*([PDL1_receptors-per-Tcell]/[Avagadro's_Num])*([Cancer_per_T_Cell_Int]/[Cancer_per_T_Cell_max]) |
| 267 | [Exp_CD28/80/86/PD1/L1/L2]*Tumor.[T{PD1}{L1}{80}-{PD1}{80}{L1}{L2}C]*([PD1_receptors-per-Tcell]/[Avagadro's_Num])*([Cancer_per_T_Cell_Int]/[Cancer_per_T_Cell_max]) |
| 268 | [Exp_CD28/80/86/PD1/L1/L2]*Tumor.[T{PD1}{L1}{80}-{PD1}{80}{L1}{L2}C]*([CD80_receptors-per-Tcell]/[Avagadro's_Num])*([Cancer_per_T_Cell_Int]/[Cancer_per_T_Cell_max]) |
| 269 | [Exp_CD28/80/86/PD1/L1/L2]*Tumor.[T{PD1}{L1}{80}-{PD1}{80}{L1}C]*([PDL1_receptors-per-Tcell]/[Avagadro's_Num])*([Cancer_per_T_Cell_Int]/[Cancer_per_T_Cell_max]) |
| 270 | [Exp_CD28/80/86/PD1/L1/L2]*Tumor.[T{PD1}{L1}{80}-{PD1}{80}{L1}C]*([PD1_receptors-per-Tcell]/[Avagadro's_Num])*([Cancer_per_T_Cell_Int]/[Cancer_per_T_Cell_max]) |
| 271 | [Exp_CD28/80/86/PD1/L1/L2]*Tumor.[T{PD1}{L1}{80}-{PD1}{80}{L1}C]*([CD80_receptors-per-Tcell]/[Avagadro's_Num])*([Cancer_per_T_Cell_Int]/[Cancer_per_T_Cell_max]) |
| 272 | [Exp_CD28/80/86/PD1/L1/L2]*Tumor.[T{PD1}{L1}{80}-{PD1}{L1}{L2}C]*([PDL1_receptors-per-Tcell]/[Avagadro's_Num])*([Cancer_per_T_Cell_Int]/[Cancer_per_T_Cell_max]) |
| 273 | [Exp_CD28/80/86/PD1/L1/L2]*Tumor.[T{PD1}{L1}{80}-{PD1}{L1}{L2}C]*([PD1_receptors-per-Tcell]/[Avagadro's_Num])*([Cancer_per_T_Cell_Int]/[Cancer_per_T_Cell_max]) |
| 274 | [Exp_CD28/80/86/PD1/L1/L2]*Tumor.[T{PD1}{L1}{80}-{PD1}{L1}{L2}C]*([CD80_receptors-per-Tcell]/[Avagadro's_Num])*([Cancer_per_T_Cell_Int]/[Cancer_per_T_Cell_max]) |
| 275 | [Exp_CD28/80/86/PD1/L1/L2]*Tumor.[T{PD1}{L1}{80}-{PD1}{L1}C]*([PDL1_receptors-per-Tcell]/[Avagadro's_Num])*([Cancer_per_T_Cell_Int]/[Cancer_per_T_Cell_max]) |
| 276 | [Exp_CD28/80/86/PD1/L1/L2]*Tumor.[T{PD1}{L1}{80}-{PD1}{L1}C]*([PD1_receptors-per-Tcell]/[Avagadro's_Num])*([Cancer_per_T_Cell_Int]/[Cancer_per_T_Cell_max]) |
| 277 | [Exp_CD28/80/86/PD1/L1/L2]*Tumor.[T{PD1}{L1}{80}-{PD1}{L1}C]*([CD80_receptors-per-Tcell]/[Avagadro's_Num])*([Cancer_per_T_Cell_Int]/[Cancer_per_T_Cell_max]) |
| 278 | [Exp_CD28/80/86/PD1/L1/L2]*Tumor.[T{PDL1}-{CD80}{PD1}C]*([PDL1_receptors-per-Tcell]/[Avagadro's_Num])*([Cancer_per_T_Cell_Int]/[Cancer_per_T_Cell_max]) |
| 279 | [Exp_CD28/80/86/PD1/L1/L2]*Tumor.[T{PDL1}-{CD80}C]*([PDL1_receptors-per-Tcell]/[Avagadro's_Num])*([Cancer_per_T_Cell_Int]/[Cancer_per_T_Cell_max]) |
| 280 | [Exp_CD28/80/86/PD1/L1/L2]*Tumor.[T{PDL1}-{PD1}C]*([PDL1_receptors-per-Tcell]/[Avagadro's_Num])*([Cancer_per_T_Cell_Int]/[Cancer_per_T_Cell_max]) |
| 281 | [Exp_CD28/80/86/PD1/L1/L2]*(Tumor.[T10a=PDL1]+[T10a=PDL1:aPDL1]+[T10a=PDL1:PD1=C10a]) |
| 282 | [kon_PD1_PDL1]*Tumor.[T10a=PDL1]*Tumor.[C10a=PD1]/[V_T:C_10] - [koff_PD1_PDL1]*Tumor.[T10a=PDL1:PD1=C10a] |
| 283 | [kon_PDL1-PDL1mAb]*Tumor.[T10a=PDL1]*Tumor.PDL1_mabT-[koff_PDL1-PDL1mAb]*Tumor.[T10a=PDL1:aPDL1] |
| 284 | [Exp_CD28/80/86/PD1/L1/L2]*(Tumor.[T10b=PD1]+[T10b=PD1:aPD1]+ [T10b=PD1:PDL1=C10b]+ [T10b=PD1:PDL2=C10c]) |
| 285 | [kon_PD1_PDL1]*Tumor.[T10b=PD1]*Tumor.[C10b=PDL1]/[V_T:C_10] - [koff_PD1_PDL1]*Tumor.[T10b=PD1:PDL1=C10b] |
| 286 | [kon_PD1_PDL2]*Tumor.[T10b=PD1]*Tumor.[C10c=PDL2]/[V_T:C_10] - [koff_PD1_PDL2]*Tumor.[T10b=PD1:PDL2=C10c] |
| 287 | [Exp_CD28/80/86/PD1/L1/L2]*(Tumor.[T10c=CD80]+ [T10c=CD80:PDL1=C10b]) |
| 288 | [kon_PDL1_CD80]*Tumor.[T10c=CD80]*Tumor.[C10b=PDL1]/[V_T:C_10] - [koff_PDL1_CD80]*Tumor.[T10c=CD80:PDL1=C10b] |
| 289 | [Exp_CD28/80/86/PD1/L1/L2]*(Tumor.[T11a=PDL1]+[T11a=PDL1:aPDL1]+ [T11a=PDL1:CD80=C11b]) |
| 290 | [kon_PDL1_CD80]*Tumor.[T11a=PDL1]*Tumor.[C11b=CD80]/[V_T:C_11] - [koff_PDL1_CD80]*Tumor.[T11a=PDL1:CD80=C11b] |
| 291 | [kon_PDL1-PDL1mAb]*Tumor.[T11a=PDL1]*Tumor.PDL1_mabT-[koff_PDL1-PDL1mAb]*Tumor.[T11a=PDL1:aPDL1] |
| 292 | [Exp_CD28/80/86/PD1/L1/L2]*(Tumor.[T11b=PD1]+[T11b=PD1:aPD1]+ [T11b=PD1:PDL1=C11a]) |
| 293 | [kon_PD1_PDL1]*Tumor.[T11b=PD1]*Tumor.[C11a=PDL1]/[V_T:C_11] - [koff_PD1_PDL1]*Tumor.[T11b=PD1:PDL1=C11a] |
| 294 | [Exp_CD28/80/86/PD1/L1/L2]*(Tumor.[T12a=PDL1]+[T12a=PDL1:aPDL1]+ [T12a=PDL1:CD80=C12b]) |
| 295 | [kon_PDL1_CD80]*Tumor.[T12a=PDL1]*Tumor.[C12b=CD80]/[V_T:C_12] - [koff_PDL1_CD80]*Tumor.[T12a=PDL1:CD80=C12b] |
| 296 | [kon_PDL1-PDL1mAb]*Tumor.[T12a=PDL1]*Tumor.PDL1_mabT-[koff_PDL1-PDL1mAb]*Tumor.[T12a=PDL1:aPDL1] |
| 297 | [Exp_CD28/80/86/PD1/L1/L2]*(Tumor.[T12b=PD1]+[T12b=PD1:aPD1]+ [T12b=PD1:PDL2=C12a]) |
| 298 | [kon_PD1_PDL2]*Tumor.[T12b=PD1]*Tumor.[C12a=PDL2]/[V_T:C_12] - [koff_PD1_PDL2]*Tumor.[T12b=PD1:PDL2=C12a] |
| 299 | [Exp_CD28/80/86/PD1/L1/L2]*(Tumor.[T13a=PDL1]+[T13a=PDL1:aPDL1]+ [T13a=PDL1:CD80=C13c]) |
| 300 | [kon_PDL1_CD80]*Tumor.[T13a=PDL1]*Tumor.[C13c=CD80]/[V_T:C_13] - [koff_PDL1_CD80]*Tumor.[T13a=PDL1:CD80=C13c] |
| 301 | [Exp_CD28/80/86/PD1/L1/L2]*(Tumor.[T13b=PD1]+[T13b=PD1:aPD1]+ [T13b=PD1:PDL2=C13b]+ [T13b=PD1:PDL1=C13a]) |
| 302 | [kon_PD1_PDL1]*Tumor.[T13b=PD1]*Tumor.[C13a=PDL1]/[V_T:C_13] - [koff_PD1_PDL1]*Tumor.[T13b=PD1:PDL1=C13a] |
| 303 | [kon_PD1_PDL2]*Tumor.[T13b=PD1]*Tumor.[C13b=PDL2]/[V_T:C_13] - [koff_PD1_PDL2]*Tumor.[T13b=PD1:PDL2=C13b] |
| 304 | [kon_PD1-PD1mAb]*Tumor.[T13b=PD1]*Tumor.PD1_mabT-[koff_PD1-PD1mAb]*Tumor.[T13b=PD1:aPD1] |
| 305 | [Exp_CD28/80/86/PD1/L1/L2]*(Tumor.[T14a=PDL1]+[T14a=PDL1:aPDL1]+ [T14a=PDL1:PD1=C14a]+ [T14a=PDL1:CD80=C14c]) |
| 306 | [kon_PDL1-PDL1mAb]*Tumor.[T14a=PDL1]*Tumor.PDL1_mabT-[koff_PDL1-PDL1mAb]*Tumor.[T14a=PDL1:aPDL1] |
| 307 | [Exp_CD28/80/86/PD1/L1/L2]*(Tumor.[T14b=PD1]+[T14b=PD1:aPD1]+ [T14b=PD1:PDL1=C14b]) |
| 308 | [kon_PD1_PDL1]*Tumor.[T14b=PD1]*Tumor.[C14b=PDL1]/[V_T:C_14] - [koff_PD1_PDL1]*Tumor.[T14b=PD1:PDL1=C14b] |
| 309 | [Exp_CD28/80/86/PD1/L1/L2]*(Tumor.[T14c=CD80]+ [T14c=CD80:PDL1=C14b]) |
| 310 | [Exp_CD28/80/86/PD1/L1/L2]*(Tumor.[T15a=PDL1]+[T15a=PDL1:aPDL1]+ [T15a=PDL1:CD80=C15c]+ [T15a=PDL1:PD1=C15a]) |
| 311 | [kon_PD1_PDL1]*Tumor.[T15a=PDL1]*Tumor.[C15a=PD1]/[V_T:C_15] - [koff_PD1_PDL1]*Tumor.[T15a=PDL1:PD1=C15a] |
| 312 | [kon_PDL1-PDL1mAb]*Tumor.[T15a=PDL1]*Tumor.PDL1_mabT-[koff_PDL1-PDL1mAb]*Tumor.[T15a=PDL1:aPDL1] |
| 313 | [Exp_CD28/80/86/PD1/L1/L2]*(Tumor.[T15b=PD1]+[T15b=PD1:aPD1]+ [T15b=PD1:PDL2=C15b]) |
| 314 | [kon_PD1-PD1mAb]*Tumor.[T15b=PD1]*Tumor.PD1_mabT-[koff_PD1-PD1mAb]*Tumor.[T15b=PD1:aPD1] |
| 315 | [Exp_CD28/80/86/PD1/L1/L2]*(Tumor.[T16a=PDL1]+[T16a=PDL1:aPDL1]+ [T16a=PDL1:PD1=C16a]+ [T16a=PDL1:CD80=C16d]) |
| 316 | [kon_PDL1_CD80]*Tumor.[T16a=PDL1]*Tumor.[C16d=CD80]/[V_T:C_16] - [koff_PDL1_CD80]*Tumor.[T16a=PDL1:CD80=C16d] |
| 317 | [kon_PDL1-PDL1mAb]*Tumor.[T16a=PDL1]*Tumor.PDL1_mabT-[koff_PDL1-PDL1mAb]*Tumor.[T16a=PDL1:aPDL1] |
| 318 | [Exp_CD28/80/86/PD1/L1/L2]*(Tumor.[T16b=PD1]+[T16b=PD1:aPD1]+ [T16b=PD1:PDL2=C16c]+ [T16b=PD1:PDL1=C16b]) |
| 319 | [kon_PD1_PDL1]*Tumor.[T16b=PD1]*Tumor.[C16b=PDL1]/[V_T:C_16] - [koff_PD1_PDL1]*Tumor.[T16b=PD1:PDL1=C16b] |
| 320 | [kon_PD1-PD1mAb]*Tumor.[T16b=PD1]*Tumor.PD1_mabT-[koff_PD1-PD1mAb]*Tumor.[T16b=PD1:aPD1] |
| 321 | [Exp_CD28/80/86/PD1/L1/L2]*(Tumor.[T16c=CD80]+ [T16c=CD80:PDL1=C16b]) |
| 322 | [kon_PDL1_CD80]*Tumor.[T16c=CD80]*Tumor.[C16b=PDL1]/[V_T:C_16] - [koff_PDL1_CD80]*Tumor.[T16c=CD80:PDL1=C16b] |
| 323 | [Exp_CD28/80/86/PD1/L1/L2]*(Tumor.[T2=PDL1]+[T2=PDL1:aPDL1]+[T2=PDL1:PD1=C2]) |
| 324 | [kon_PD1_PDL1]*Tumor.[T2=PDL1]*Tumor.[C2=PD1]/[V_T:C_2] - [koff_PD1_PDL1]*Tumor.[T2=PDL1:PD1=C2] |
| 325 | [Exp_CD28/80/86/PD1/L1/L2]*(Tumor.[T3a=PD1]+[T3a=PD1:aPD1]+[T3a=PD1:PDL1=C3]) |
| 326 | [kon_PD1_PDL1]*Tumor.[T3a=PD1]*Tumor.[C3=PDL1]/[V_T:C_3] - [koff_PD1_PDL1]*Tumor.[T3a=PD1:PDL1=C3] |
| 327 | [kon_PD1-PD1mAb]*Tumor.[T3a=PD1]*Tumor.PD1_mabT-[koff_PD1-PD1mAb]*Tumor.[T3a=PD1:aPD1] |
| 328 | [Exp_CD28/80/86/PD1/L1/L2]*(Tumor.[T3b=CD80]+[T3b=CD80:PDL1=C3]) |
| 329 | [kon_PDL1_CD80]*Tumor.[T3b=CD80]*Tumor.[C3=PDL1]/[V_T:C_3] - [koff_PDL1_CD80]*Tumor.[T3b=CD80:PDL1=C3] |
| 330 | [Exp_CD28/80/86/PD1/L1/L2]*(Tumor.[T4=PD1]+[T4=PD1:aPD1]+[T4=PD1:PDL2=C4]) |
| 331 | [kon_PD1_PDL2]*Tumor.[T4=PD1]*Tumor.[C4=PDL2]/[V_T:C_4] - [koff_PD1_PDL2]*Tumor.[T4=PD1:PDL2=C4] |
| 332 | [kon_PD1-PD1mAb]*Tumor.[T4=PD1]*Tumor.PD1_mabT-[koff_PD1-PD1mAb]*Tumor.[T4=PD1:aPD1] |
| 333 | [Exp_CD28/80/86/PD1/L1/L2]*(Tumor.[T5=PDL1]+[T5=PDL1:aPDL1]+[T5=PDL1:CD80=C5]) |
| 334 | [kon_PDL1_CD80]*Tumor.[T5=PDL1]*Tumor.[C5=CD80]/[V_T:C_5] - [koff_PDL1_CD80]*Tumor.[T5=PDL1:CD80=C5] |
| 335 | [kon_PDL1-PDL1mAb]*Tumor.[T5=PDL1]*Tumor.PDL1_mabT-[koff_PDL1-PDL1mAb]*Tumor.[T5=PDL1:aPDL1] |
| 336 | [Exp_CD28/80/86/PD1/L1/L2]*(Tumor.[T6a=PDL1]+[T6a=PDL1:aPDL1]+[T6a=PDL1:PD1=C6a]) |
| 337 | [kon_PD1_PDL1]*Tumor.[T6a=PDL1]*Tumor.[C6a=PD1]/[V_T:C_6] - [koff_PD1_PDL1]*Tumor.[T6a=PDL1:PD1=C6a] |
| 338 | [kon_PDL1-PDL1mAb]*Tumor.[T6a=PDL1]*Tumor.PDL1_mabT-[koff_PDL1-PDL1mAb]*Tumor.[T6a=PDL1:aPDL1] |
| 339 | [Exp_CD28/80/86/PD1/L1/L2]*(Tumor.[T6b=PD1]+[T6b=PD1:aPD1]+[T6b=PD1:PDL1=C6b]) |
| 340 | [kon_PD1-PD1mAb]*Tumor.[T6b=PD1]*Tumor.PD1_mabT-[koff_PD1-PD1mAb]*Tumor.[T6b=PD1:aPD1] |
| 341 | [Exp_CD28/80/86/PD1/L1/L2]*(Tumor.[T6c=CD80]+[T6c=CD80:PDL1=C6b]) |
| 342 | [Exp_CD28/80/86/PD1/L1/L2]*(Tumor.[T7a=PDL1]+[T7a=PDL1:aPDL1]+[T7a=PDL1:PD1=C7a]) |
| 343 | [kon_PD1_PDL1]*Tumor.[T7a=PDL1]*Tumor.[C7a=PD1]/[V_T:C_7] - [koff_PD1_PDL1]*Tumor.[T7a=PDL1:PD1=C7a] |
| 344 | [Exp_CD28/80/86/PD1/L1/L2]*(Tumor.[T7b=PD1]+[T7b=PD1:aPD1]+[T7b=PD1:PDL2=C7b]) |
| 345 | [kon_PD1_PDL2]*Tumor.[T7b=PD1]*Tumor.[C7b=PDL2]/[V_T:C_7] - [koff_PD1_PDL2]*Tumor.[T7b=PD1:PDL2=C7b] |
| 346 | [Exp_CD28/80/86/PD1/L1/L2]*(Tumor.[T8a=PD1]+[T8a=PD1:aPD1]+[T8a=PD1:PDL1=C8a]+[T8a=PD1:PDL2=C8b]) |
| 347 | [kon_PD1_PDL1]*Tumor.[T8a=PD1]*Tumor.[C8a=PDL1]/[V_T:C_8] - [koff_PD1_PDL1]*Tumor.[T8a=PD1:PDL1=C8a] |
| 348 | [kon_PD1_PDL2]*Tumor.[T8a=PD1]*Tumor.[C8b=PDL2]/[V_T:C_8] - [koff_PD1_PDL2]*Tumor.[T8a=PD1:PDL2=C8b] |
| 349 | [Exp_CD28/80/86/PD1/L1/L2]*(Tumor.[T8b=CD80]+[T8b=CD80:PDL1=C8a]) |
| 350 | [Exp_CD28/80/86/PD1/L1/L2]*(Tumor.[T9=PDL1]+[T9=PDL1:aPDL1]+[T9=PDL1-PD1=C9a]+[T9=PDL1-CD80=C9b]) |
| 351 | [kon_PDL1_CD80]*Tumor.[T9=PDL1]*Tumor.[C9b=CD80]/[V_T:C_9] - [koff_PDL1_CD80]*Tumor.[T9=PDL1-CD80=C9b] |
| 352 | [kon_PDL1-PDL1mAb]*Tumor.[T9=PDL1]*Tumor.PDL1_mabT-[koff_PDL1-PDL1mAb]*Tumor.[T9=PDL1:aPDL1] |
| 353 | kf_RestingMacrophage*Tumor.APC_T |
| 354 | Debris_Transport*Tumor.C_DebrisT*(1/[Num_TDLN_Considered])*(1-[mAPC_Debis_T_Inact]) |
| 355 | Debris_Decay*Tumor.C_DebrisT |
| 356 | Debris_Transport*Tumor.C_DebrisT*[mAPC_Debis_T_Inact] |
| 357 | Debris_Transport*Tumor.C_DebrisT*(1-1/[Num_TDLN_Considered]) |
| 358 | Phago_Debris*Tumor.APC_T*Tumor.C_DebrisT |
| 359 | kf_CanDecay*Tumor.Cancer |
| 360 | [Exp_CD28/80/86/PD1/L1/L2]*(Tumor.CD80_mAPCT+[CTLA4:CD80_TrAT]) |
| 361 | [Exp_CD28/80/86/PD1/L1/L2]*(Tumor.CD80_TeffT+[PDL1:CD80_TrTeff]) |
| 362 | [kon_PDL1_CD80]*Tumor.CD80_TeffT*Tumor.PDL1_TregT/[Vol_Cell-Rec_Tr-TeffT] - [koff_PDL1_CD80]*Tumor.[PDL1:CD80_TrTeff] |
| 363 | [Exp_CD28/80/86/PD1/L1/L2]*(Tumor.CD80_TeffT1+[PDL1:CD80_MDSCT]) |
| 364 | [Exp_CD28/80/86/PD1/L1/L2]*(Tumor.CD86_mAPCT+[CTLA4:CD86_TrAT]) |
| 365 | k_Lt*Tumor/K_T*Tumor.CTLA4_mabT |
| 366 | kon_CTLA4mAb_CTLA4*Tumor.CTLA4_mabT*Tumor.CTLA4_TregTS-koff_CTLA4mAb_CTLA4*Tumor.[CTLA4_aCTLA4-TrTS] |
| 367 | [Exp_CTLA4]*(Tumor.CTLA4_TregT+[CTLA4:CD80_TrAT]+[CTLA4:CD86_TrAT]+[CTLA4_CTLA4-Trt]) |
| 368 | [kon_CTLA4_CD80]*Tumor.CTLA4_TregT*Tumor.CD80_mAPCT/[Vol_Cell-Rec_Tr-APCT] - [koff_CTLA4_CD80]*Tumor.[CTLA4:CD80_TrAT] |
| 369 | [kon_CTLA4_CD86]*Tumor.CTLA4_TregT*Tumor.CD86_mAPCT/[Vol_Cell-Rec_Tr-APCT] - [koff_CTLA4_CD86]*Tumor.[CTLA4:CD86_TrAT] |
| 370 | kon_CTLA4mAb_CTLA4*Tumor.CTLA4_TregT*Tumor.CTLA4_mabT-koff_CTLA4mAb_CTLA4*Tumor.[CTLA4_CTLA4-Trt] |
| 371 | [Exp_CTLA4]*(Tumor.CTLA4_TregTS+[CTLA4_aCTLA4-TrTS]) |
| 372 | EffT_Turnover*Tumor.Effector_Ta |
| 373 | J_T*Tumor.Effector_Ta |
| 374 | EffT_Turnover*Tumor.Effector_Tb |
| 375 | AR_T*Tumor.Effector_Tb |
| 376 | kf_T*(B_T-Tumor.Effector_Tb/Vv_T-Tumor.Effector_Ta/Vv_T)*Tumor.Effector_Tf-kr_T*Tumor.Effector_Tb |
| 377 | EffT_Turnover*Tumor.Effector_TT |
| 378 | CancerTEng*Tumor.Cancer1*Tumor.Effector_TT_C_Eng/Total_Cell_T |
| 379 | EffT_Turnover*Tumor.Exhausted_TT |
| 380 | mAPC_Migrate*Tumor.mAPC_T*(1/[Num_TDLN_Considered])*(1-[mAPC_Debis_T_Inact]) |
| 381 | mAPC_Migrate*Tumor.mAPC_T*(1-1/[Num_TDLN_Considered]) |
| 382 | mAPC_Migrate*Tumor.mAPC_T*[mAPC_Debis_T_Inact] |
| 383 | [Exp_CD28/80/86/PD1/L1/L2]*Tumor.mAPCT_EngTregT*([CD80_receptors-per-mAPC]/[Avagadro's_Num])*(1/Tr_cells_per_mAPC) |
| 384 | [Exp_CD28/80/86/PD1/L1/L2]*Tumor.mAPCT_EngTregT*([CD86_receptors-per-mAPC]/[Avagadro's_Num])*(1/Tr_cells_per_mAPC) |
| 385 | TregTMDSCEng*Tumor.Effector_TT_MDSCs*Tumor.MDSC_T/Total_Cell_T |
| 386 | [Exp_CD28/80/86/PD1/L1/L2]*Tumor.MDSCsT_EngTeff*([PD1_receptors-per-Tcell]/[Avagadro's_Num])*(1/T_per_Tr_cell) |
| 387 | [Exp_CD28/80/86/PD1/L1/L2]*Tumor.MDSCsT_EngTeff*([PDL1_receptors-per-Tcell]/[Avagadro's_Num])*(1/T_per_Tr_cell) |
| 388 | [Treg:T_IntTime]*Tumor.MDSCsT_Teff*(1-[Sig_MDSCTeff=Total]) |
| 389 | [Treg:T_IntTime]*Tumor.MDSCsT_Teff*[Sig_MDSCTeff=Total] |
| 390 | k_Lt*Tumor/K_T*Tumor.PD1_mabT |
| 391 | [kon_PD1-PD1mAb]*Tumor.PD1_mabT*Tumor.[C14a=PD1]-[koff_PD1-PD1mAb]*Tumor.[C14a=PD1:aPD1] |
| 392 | [kon_PD1-PD1mAb]*Tumor.PD1_mabT*Tumor.[C2=PD1]-[koff_PD1-PD1mAb]*Tumor.[C2=PD1:aPD1] |
| 393 | [kon_PD1-PD1mAb]*Tumor.PD1_mabT*Tumor.[C7a=PD1]-[koff_PD1-PD1mAb]*Tumor.[C7a=PD1:aPD1] |
| 394 | [kon_PD1-PD1mAb]*Tumor.PD1_mabT*Tumor.[C9a=PD1]-[koff_PD1-PD1mAb]*Tumor.[C9a=PD1:aPD1] |
| 395 | [kon_PD1-PD1mAb]*Tumor.PD1_mabT*Tumor.[T10b=PD1]-[koff_PD1-PD1mAb]*Tumor.[T10b=PD1:aPD1] |
| 396 | [kon_PD1-PD1mAb]*Tumor.PD1_mabT*Tumor.[T11b=PD1]-[koff_PD1-PD1mAb]*Tumor.[T11b=PD1:aPD1] |
| 397 | [kon_PD1-PD1mAb]*Tumor.PD1_mabT*Tumor.[T12b=PD1]-[koff_PD1-PD1mAb]*Tumor.[T12b=PD1:aPD1] |
| 398 | [kon_PD1-PD1mAb]*Tumor.PD1_mabT*Tumor.[T14b=PD1]-[koff_PD1-PD1mAb]*Tumor.[T14b=PD1:aPD1] |
| 399 | [kon_PD1-PD1mAb]*Tumor.PD1_mabT*Tumor.[T7b=PD1]-[koff_PD1-PD1mAb]*Tumor.[T7b=PD1:aPD1] |
| 400 | [kon_PD1-PD1mAb]*Tumor.PD1_mabT*Tumor.[T8a=PD1]-[koff_PD1-PD1mAb]*Tumor.[T8a=PD1:aPD1] |
| 401 | [kon_PD1-PD1mAb]*Tumor.PD1_mabT*Tumor.PD1_TeffT-[koff_PD1-PD1mAb]*Tumor.[PD1:aPD1_Teff] |
| 402 | [Exp_CD28/80/86/PD1/L1/L2]*(Tumor.PD1_MDSCsT+[PD1:PDL1_MDSCT]+[PD1:aPD1_MDSCs]) |
| 403 | [kon_PD1-PD1mAb]*Tumor.PD1_MDSCsT*Tumor.PD1_mabT-[koff_PD1-PD1mAb]*Tumor.[PD1:aPD1_MDSCs] |
| 404 | [kon_PD1_PDL1]*Tumor.PD1_MDSCsT*Tumor.PDL1_TeffT1/[Vol_Cell-Rec_MDSC-TeffT] - [koff_PD1_PDL1]*Tumor.[PD1:PDL1_MDSCT] |
| 405 | [Exp_CD28/80/86/PD1/L1/L2]*(Tumor.PD1_TeffT+[PDL1:PD1_TrTeff]+[PD1:aPD1_Teff]) |
| 406 | [kon_PD1_PDL1]*Tumor.PD1_TeffT*Tumor.PDL1_TregT/[Vol_Cell-Rec_Tr-TeffT] - [koff_PD1_PDL1]*Tumor.[PDL1:PD1_TrTeff] |
| 407 | [Exp_CD28/80/86/PD1/L1/L2]*(Tumor.PD1_TeffT1+[PDL1:PD1_MDSCT]+[PD1:aPD1_Teff1]) |
| 408 | [kon_PD1-PD1mAb]*Tumor.PD1_TeffT1*Tumor.PD1_mabT-[koff_PD1-PD1mAb]*Tumor.[PD1:aPD1_Teff1] |
| 409 | [Exp_CD28/80/86/PD1/L1/L2]*(Tumor.PD1_TregT+[PD1:PDL1_TrTeff]+[PD1:aPD1_Treg]) |
| 410 | [kon_PD1-PD1mAb]*Tumor.PD1_TregT*Tumor.PD1_mabT-[koff_PD1-PD1mAb]*Tumor.[PD1:aPD1_Treg] |
| 411 | k_Lt*Tumor/K_T*Tumor.PDL1_mabT |
| 412 | [kon_PDL1-PDL1mAb]*Tumor.PDL1_mabT*Tumor.[C3=PDL1]-[koff_PDL1-PDL1mAb]*Tumor.[C3=PDL1:aPDL1] |
| 413 | [kon_PDL1-PDL1mAb]*Tumor.PDL1_mabT*Tumor.[T13a=PDL1]-[koff_PDL1-PDL1mAb]*Tumor.[T13a=PDL1:aPDL1] |
| 414 | [kon_PDL1-PDL1mAb]*Tumor.PDL1_mabT*Tumor.[T2=PDL1]-[koff_PDL1-PDL1mAb]*Tumor.[T2=PDL1:aPDL1] |
| 415 | [kon_PDL1-PDL1mAb]*Tumor.PDL1_mabT*Tumor.[T7a=PDL1]-[koff_PDL1-PDL1mAb]*Tumor.[T7a=PDL1:aPDL1] |
| 416 | [kon_PDL1-PDL1mAb]*Tumor.PDL1_mabT*Tumor.PDL1_TeffT-[koff_PDL1-PDL1mAb]*Tumor.[PDL1:aPDL1_Teff] |
| 417 | [kon_PDL1-PDL1mAb]*Tumor.PDL1_mabT*Tumor.PDL1_TeffT1-[koff_PDL1-PDL1mAb]*Tumor.[PDL1:aPDL1_Teff1] |
| 418 | [Exp_CD28/80/86/PD1/L1/L2]*(Tumor.PDL1_MDSCsT+[PDL1:CD80_MDSCT]+[PDL1:PD1_MDSCT]+[PDL1:aPDL1_MDSCs]) |
| 419 | [kon_PDL1_CD80]*Tumor.PDL1_MDSCsT*Tumor.CD80_TeffT1/[Vol_Cell-Rec_MDSC-TeffT] - [koff_PDL1_CD80]*Tumor.[PDL1:CD80_MDSCT] |
| 420 | [kon_PD1_PDL1]*Tumor.PDL1_MDSCsT*Tumor.PD1_TeffT1/[Vol_Cell-Rec_MDSC-TeffT] - [koff_PD1_PDL1]*Tumor.[PDL1:PD1_MDSCT] |
| 421 | [kon_PDL1-PDL1mAb]*Tumor.PDL1_MDSCsT*Tumor.PDL1_mabT-[koff_PDL1-PDL1mAb]*Tumor.[PDL1:aPDL1_MDSCs] |
| 422 | [Exp_CD28/80/86/PD1/L1/L2]*(Tumor.PDL1_TeffT+[PD1:PDL1_TrTeff]+[PDL1:aPDL1_Teff]) |
| 423 | [kon_PD1_PDL1]*Tumor.PDL1_TeffT*Tumor.PD1_TregT/[Vol_Cell-Rec_Tr-TeffT] - [koff_PD1_PDL1]*Tumor.[PD1:PDL1_TrTeff] |
| 424 | [Exp_CD28/80/86/PD1/L1/L2]*(Tumor.PDL1_TeffT1+[PD1:PDL1_MDSCT]+[PDL1:aPDL1_Teff1]) |
| 425 | [Exp_CD28/80/86/PD1/L1/L2]*(Tumor.PDL1_TregT+[PDL1:CD80_TrTeff]+[PDL1:PD1_TrTeff]+[PDL1:aPDL1_Treg]) |
| 426 | [kon_PDL1-PDL1mAb]*Tumor.PDL1_TregT*Tumor.PDL1_mabT-[koff_PDL1-PDL1mAb]*Tumor.[PDL1:aPDL1_Treg] |
| 427 | CancerTInt*Tumor.TC1*[Total_TC_Sig]*[%Sig_Inhibit_Cancer]*(1-[%Deep_Exhausted_T]) |
| 428 | CancerTInt*Tumor.TC1*(1-[Total_TC_Sig])*[%Sig_Inhibit_Cancer] |
| 429 | CancerTInt*Tumor.TC1*[Total_TC_Sig]*[%Sig_Inhibit_Cancer]*[%Deep_Exhausted_T] |
| 430 | [Exp_CD28/80/86/PD1/L1/L2]*Tumor.Teff_EngMDSC*([CD80_receptors-per-Tcell]/[Avagadro's_Num])*(1/Tr_per_T_cell) |
| 431 | [Exp_CD28/80/86/PD1/L1/L2]*Tumor.Teff_EngMDSC*([PD1_receptors-per-Tcell]/[Avagadro's_Num])*(1/Tr_per_T_cell) |
| 432 | [Exp_CD28/80/86/PD1/L1/L2]*Tumor.Teff_EngMDSC*([PDL1_receptors-per-Tcell]/[Avagadro's_Num])*(1/Tr_per_T_cell) |
| 433 | [Exp_CD28/80/86/PD1/L1/L2]*Tumor.Teff_EngTregT*([CD80_receptors-per-Tcell]/[Avagadro's_Num])*(1/Tr_per_T_cell) |
| 434 | [Exp_CD28/80/86/PD1/L1/L2]*Tumor.Teff_EngTregT*([PD1_receptors-per-Tcell]/[Avagadro's_Num])*(1/Tr_per_T_cell) |
| 435 | [Exp_CD28/80/86/PD1/L1/L2]*Tumor.Teff_EngTregT*([PDL1_receptors-per-Tcell]/[Avagadro's_Num])*(1/Tr_per_T_cell) |
| 436 | TregTMDSCEng*Tumor.TregT*Tumor.Effector_TT_TregT/Total_Cell_T |
| 437 | TregTMDSCEng*Tumor.mAPC_T*Tumor.TregT/Total_Cell_T |
| 438 | [Exp_CTLA4]*Tumor.TregT_EngAPC*([CTLA4_receptors-Tr]/[Avagadro's_Num])*(1/mAPC_per_Tr_cell) |
| 439 | [Exp_CD28/80/86/PD1/L1/L2]*Tumor.TregT_EngTeff*([PD1_receptors-per-Tcell]/[Avagadro's_Num])*(1/T_per_Tr_cell) |
| 440 | [Exp_CD28/80/86/PD1/L1/L2]*Tumor.TregT_EngTeff*([PDL1_receptors-per-Tcell]/[Avagadro's_Num])*(1/T_per_Tr_cell) |
| 441 | [Treg:mAPC_IntTime]*Tumor.TregT_mAPCT*(1-[Sig_TrAPCT=Total]) |
| 442 | [Treg:mAPC_IntTime]*Tumor.TregT_mAPCT*[Sig_TrAPCT=Total] |
| 443 | [Treg:T_IntTime]*Tumor.TregT_Teff*(1-[Sig_TrTeff=Total]) |
| 444 | [Treg:T_IntTime]*Tumor.TregT_Teff*[Sig_TrTeff=Total] |
| 445 | [Exp_CTLA4]*Tumor.TregT1*([CTLA4_receptors-Tr]/[Avagadro's_Num]) |

**Table S5 – Model Reaction Rates (End)**

**Table S6 – Model Reaction and Rate Descriptions (Start)**

| Reaction Number | Reaction Description |
| --- | --- |
| 1 | Clearance of Anti-CTLA-4 mAb from central compartment |
| 2 | Diffusion of Anti-CTLA-4 mAb from central to TDLN compartment |
| 3 | Diffusion of Anti-CTLA-4 mAb between central and peripheral compartments |
| 4 | Diffusion of Anti-CTLA-4 mAb between central and tumor compartments |
| 5 | The generation of Effector T cells and their migration into the blood/plasma from the designated number of lymph nodes as a multiple of that from a single lymph node |
| 6 | Distribution of Effector T cells between the blood/plasma (central compartment) and peripheral tissues (peripheral compartment) |
| 7 | Effector T cell transport from central to tumor compartment as free cells |
| 8 | Clearance of Anti-PD-1 mAb from central compartment |
| 9 | Diffusion of Anti-PD-1 mAb from central to TDLN compartment |
| 10 | Diffusion of Anti-PD-1 mAb between central and peripheral compartments |
| 11 | Diffusion of Anti-PD-1 mAb between central and tumor compartments |
| 12 | Clearance of Anti-PD-L1 mAb from central compartment |
| 13 | Diffusion of Anti-PD-L1 mAb from central to TDLN compartment |
| 14 | Diffusion of Anti-PD-L1 mAb between central and peripheral compartments |
| 15 | Diffusion of Anti-PD-L1 mAb between central and tumor compartments |
| 16 | CD80 expression threshold on Primed Naive T cells for interaction with T Regulatory cells in the lymph node |
| 17 | Interaction at the immunological synapse between CD80 on Primed Naive T cells and PD-L1 on T Regulatory cells in the lymph node |
| 18 | PD-1 expression threshold on Primed Naive T cells for interaction with T Regulatory cells in the lymph node |
| 19 | Interaction at the immunological synapse between PD-1 on Primed Naive T cells and PD-L1 on T Regulatory cells in the lymph node |
| 20 | Interaction at the immunological synapse between PD-1 on Primed Naive T cells and Anti-PD-1 mAb in the lymph node |
| 21 | PD-L1 expression threshold on Primed Naive T cells for interaction with T Regulatory cells in the lymph node |
| 22 | Interaction at the immunological synapse between PD-L1 on Primed Naive T cells and Anti-PD-L1 mAb in the lymph node |
| 23 | CD80 expression threshold on mAPCs for interaction with T Regulatory cells in the lymph node |
| 24 | CD86 expression threshold on mAPCs for interaction with T Regulatory cells in the lymph node |
| 25 | CTLA-4 expression threshold on T Regulatory cells for interaction with mAPCs in the lymph node |
| 26 | Interaction at the immunological synapse between CD80 on mAPCs and CTLA-4 on T Regulatory cells in the lymph node |
| 27 | Interaction at the immunological synapse between CD86 on mAPCs and CTLA-4 on T Regulatory cells in the lymph node |
| 28 | Interaction at the immunological synapse between CTLA-4 on T Regulatory cells and Anti-CTLA-4 mAb in the lymph node |
| 29 | PD-1 expression threshold on Regulatory cells for interaction with Primed Naive T cells in the lymph node |
| 30 | Interaction at the immunological synapse between PD-L1 on Primed Naive T cells and PD-1 on T Regulatory cells in the lymph node |
| 31 | Interaction at the immunological synapse between PD-1 on T Regulatory cells and Anti-PD-1 mAb in the lymph node |
| 32 | PD-L1 expression threshold on Regulatory cells for interaction with Primed Naive T cells in the lymph node |
| 33 | Interaction at the immunological synapse between PD-L1 on T Regulatory cells and Anti-PD-L1 mAb in the lymph node |
| 34 | Dissociation of T Regulatory cells from Naïve T cells in the lymph node without inactivation of the latter |
| 35 | Inactivation of Naïve T cells by T Regulatory cells in the lymph node |
| 36 | Dissociation of T Regulatory cells from Primed Naïve T cells in the lymph node without inactivation of the latter |
| 37 | Inactivation of Primed Naïve T cells by T Regulatory cells in the lymph node |
| 38 | CD80 expression by Primed Naive T cells for interaction with T Regulatory cells in the lymph node |
| 39 | PD-1 expression by Primed Naive T cells for interaction with T Regulatory cells in the lymph node |
| 40 | PD-L1 expression by Primed Naive T cells for interaction with T Regulatory cells in the lymph node |
| 41 | PD-1 expression by T Regulatory cells for interaction with Primed Naive T cells in the lymph node |
| 42 | PD-L1 expression by T Regulatory cells for interaction with Primed Naive T cells in the lymph node |
| 43 | Phagocytosis of tumor debris by resident APCs in the lymph node |
| 44 | Decay of tumor debris in the lymph nodes |
| 45 | Lymphatic drainage and diffusion of Anti-CTLA-4 mAb from lymph node to blood |
| 46 | Interaction at the immunological synapse between CTLA-4 on Primed Naïve T cells during priming interactions with mAPCS and Anti-CTLA-4 mAb in the lymph node |
| 47 | Internalization of the CTLA-4 receptor on Primed Naïve T cells following its binding to Anti-CTLA-4 mAb |
| 48 | Migration of Effector T cells from the lymph node into the blood |
| 49 | CD80 expression by total mAPCs in the lymph node |
| 50 | CD86 expression by total mAPCs in the lymph node |
| 51 | PD-1 expression by total mAPCs in the lymph node |
| 52 | PD-L1 expression by total mAPCs in the lymph node |
| 53 | PD-L2 expression by total mAPCs in the lymph node |
| 54 | Natural turnover of mAPCs in the lymph node |
| 55 | Inactivation of mAPCs in the lymph node as a result of CTLA-4 secreted from T Regulatory cells in the lymph nodes binding to CD80 and CD86 |
| 56 | CD80 expression threshold on mAPCs in the lymph node |
| 57 | CD86 expression threshold on mAPCs in the lymph node |
| 58 | CD80 expression by mAPCs that interact with Naive T cells in the lymph node |
| 59 | CD86 expression by mAPCs that interact with Naive T cells in the lymph node |
| 60 | CD80 expression threshold on mAPCs for interaction with Naive T cells in the lymph node |
| 61 | CD86 expression threshold on mAPCs for interaction with Naive T cells in the lymph node |
| 62 | CD80 expression by mAPCs that interact with Primed Naive T cells in the lymph node |
| 63 | CD86 expression by mAPCs that interact with Primed Naive T cells in the lymph node |
| 64 | PD-1 expression by mAPCs that interact with Primed Naive T cells in the lymph node |
| 65 | PD-L1 expression by mAPCs that interact with Primed Naive T cells in the lymph node |
| 66 | PD-L2 expression by mAPCs that interact with Primed Naive T cells in the lymph node |
| 67 | CD80 expression threshold on mAPCs for interaction with Primed Naive T cells in the lymph node |
| 68 | Interaction at the immunological synapse between CD80 on mAPCs and CTLA-4 on Primed Naïve T cells in the lymph node |
| 69 | Interaction at the immunological synapse between CD80 on mAPCs and PD-L1 on Primed Naïve T cells in the lymph node |
| 70 | CD86 expression threshold on mAPCs for interaction with Primed Naive T cells in the lymph node |
| 71 | Interaction at the immunological synapse between CD86 on mAPCs and CTLA-4 on Primed Naïve T cells in the lymph node |
| 72 | Interaction at the immunological synapse between CD86 on mAPCs and CD28 on Primed Naïve T cells in the lymph node |
| 73 | PD-1 expression threshold on mAPCs for interaction with Primed Naive T cells in the lymph node |
| 74 | Interaction at the immunological synapse between PD-1 on mAPCs cells during priming interactions with Primed Naïve T cells and Anti-PD-1 mAb in the lymph node |
| 75 | PD-L1 expression threshold on mAPCs for interaction with Primed Naive T cells in the lymph node |
| 76 | PD-L2 expression threshold on mAPCs for interaction with Primed Naive T cells in the lymph node |
| 77 | Interaction at the immunological synapse between PD-L2 on mAPCs and PD-1 on Primed Naïve T cells in the lymph node |
| 78 | CD80 expression by non-interacting mAPCs in the lymph node |
| 79 | CD86 expression by non-interacting mAPCs in the lymph node |
| 80 | CD80 expression threshold on non-interacting mAPCs |
| 81 | Interaction between CD80 on mAPCs and CTLA-4 secreted by T Regulatory cells in the lymph node |
| 82 | CD86 expression threshold on non-interacting mAPCs in the lymph node |
| 83 | Interaction between CD86 on mAPCs and CTLA-4 secreted by T Regulatory cells in the lymph node |
| 84 | PD-1 expression threshold on total mAPCs in the lymph node |
| 85 | PD-L1 expression threshold on total mAPCs in the lymph node |
| 86 | PD-L2 expression threshold on total mAPCs in the lymph node |
| 87 | Engagement of Naïve T cells in the first priming phase in the lymph node |
| 88 | Naïve T cells cycling out of the lymph node |
| 89 | Naïve T cells being disengaged in the first priming phase without undergoing successful priming in the lymph node |
| 90 | Naïve T cells successfully undergoing the first priming phase in the lymph node |
| 91 | Transendocytosis of the CD80 receptors on mAPCs through CTLA-4 binding and internalization on Primed Naïve T cells in the lymph node |
| 92 | Transendocytosis of the CD86 receptors on mAPCs through CTLA-4 binding and internalization on Primed Naïve T cells in the lymph node |
| 93 | CD28 expression threshold on Naïve T cells in the lymph node |
| 94 | Interaction at the immunological synapse between CD80 on mAPCs cells and CD28 on Naïve T cells in the lymph node |
| 95 | Interaction at the immunological synapse between CD86 on mAPCs cells and CD28 on Naïve T cells in the lymph node |
| 96 | CD28 expression by Naïve T cells for interaction with mAPCs in the lymph node |
| 97 | Lymphatic drainage and diffusion of Anti-PD-1 mAb from lymph node to blood |
| 98 | Lymphatic drainage and diffusion of Anti-PD-L1 mAb from lymph node to blood |
| 99 | Interaction at the immunological synapse between PD-L1 on mAPCs involved in the second priming phase and Anti-PD-L1 mAb in the lymph node |
| 100 | Interaction at the immunological synapse between PD-L1 on Primed Naïve T cells involved in the second priming phase with mAPCs and Anti-PD-L1 mAb in the lymph node |
| 101 | CTLA-4 expression threshold on Primed Naïve T cells for interaction with mAPCs in the lymph node |
| 102 | CD28 expression threshold on Primed Naïve T cells for interaction with mAPCs in the lymph node |
| 103 | Interaction at the immunological synapse between CD80 on mAPCs and CD28 on Primed Naïve T cells in the lymph node |
| 104 | CD80 expression threshold on Primed Naïve T cells for interaction with mAPCs in the lymph node |
| 105 | Interaction at the immunological synapse between PD-L1 on mAPCs and CD80 on Primed Naïve T cells in the lymph node |
| 106 | PD-1 expression threshold on Primed Naïve T cells for interaction with mAPCs in the lymph node |
| 107 | Interaction at the immunological synapse between PD-L1 on mAPCs and PD-1 on Primed Naïve T cells in the lymph node |
| 108 | Interaction at the immunological synapse between PD-1 on Primed Naïve T cells involved in the second priming phase with mAPCs and Anti-PD-1 mAb in the lymph node |
| 109 | PD-L1 expression threshold on Primed Naïve T cells for interaction with mAPCs in the lymph node |
| 110 | Interaction at the immunological synapse between PD-1 on mAPCs and PD-L1 on Primed Naïve T cells in the lymph node |
| 111 | CTLA-4 expression by Primed Naïve T cells for interaction with mAPCs in the lymph node |
| 112 | CD28 expression by Primed Naïve T cells for interaction with mAPCs in the lymph node |
| 113 | CD80 expression by Primed Naïve T cells for interaction with mAPCs in the lymph node |
| 114 | PD-1 expression by Primed Naïve T cells for interaction with mAPCs in the lymph node |
| 115 | PD-L1 expression by Primed Naïve T cells for interaction with mAPCs in the lymph node |
| 116 | Primed Naïve T cells engaging in the second priming phase in the lymph node |
| 117 | Primed Naïve T cells unsuccessfully undergoing the second priming phase to become anergic T cells in the lymph node |
| 118 | Primed Naïve T cells engaged in the second priming phase disengaging form being primed in the lymph node |
| 119 | Primed Naïve T cells engaged in the second priming phase successfully undergoing priming to become Proliferating T cells in the lymph node |
| 120 | Proliferating T cells becoming fully activated Effector T cells in the lymph node |
| 121 | Sets the proliferation threshold for Proliferating T cells in the lymph node |
| 122 | Internalization of the Anti-CTLA-4 antibodies bound to CTLA-4 receptors on T Regulatory cells in the lymph node |
| 123 | Transendocytosis of the CD80 receptors on mAPCs through CTLA-4 binding and internalization on T Regulatory cells in the lymph node |
| 124 | Transendocytosis of the CD86 receptors on mAPCs through CTLA-4 binding and internalization on T Regulatory cells in the lymph node |
| 125 | CTLA-4 expression on the surface of non-interacting T Regulatory cells in the lymph node |
| 126 | Engagement of T Regulatory cells with mAPCs and other T cells in the lymph node |
| 127 | Interaction between T Regulatory cells and mAPCs in lymph node |
| 128 | Interaction between T Regulatory cells and Naïve T cells in lymph node |
| 129 | Interaction between T Regulatory cells and Primed Naïve T cells in lymph node |
| 130 | Dissociation of T Regulatory cells from mAPCs in the lymph node with inactivation of the latter |
| 131 | Dissociation of T Regulatory cells from mAPCs in the lymph node without inactivation of the latter |
| 132 | CD80 expression by mAPCs for interaction with T Regulatory cells in the lymph node |
| 133 | CD86 expression by mAPCs for interaction with T Regulatory cells in the lymph node |
| 134 | CTLA-4 expression by T Regulatory cells for interaction with mAPCs in the lymph node |
| 135 | CTLA-4 secretion by T Regulatory cells in the lymph node |
| 136 | CTLA-4 expression threshold on non-interacting T Regulatory cells in the lymph node |
| 137 | Binding of Anti-CTLA-4 to CTLA-4 expressed on the surface of non-interacting T Regulatory in the lymph node |
| 138 | Binding of Anti-CTLA-4 to CTLA-4 secreted by T Regulatory in the lymph node |
| 139 | Administration of Anti-CTLA-4 mAb into the central compartment |
| 140 | Lymphatic drainage and diffusion of Anti-CTLA-4 mAb from lymph node to blood |
| 141 | Lymphatic drainage and diffusion of Anti-PD-1 mAb from lymph node to blood |
| 142 | Administration of Anti-PD-1 mAb into the central compartment |
| 143 | Lymphatic drainage and diffusion of Anti-PD-L1 mAb from lymph node to blood |
| 144 | Administration of Anti-PD-L1 mAb into the central compartment |
| 145 | Diffusion of Anti-CTLA-4 mAb from blood to lymph node |
| 146 | Lymphatic drainage of Anti-CTLA-4 mAb from tumor to lymph node |
| 147 | Naïve T cells cycling into the lymph node |
| 148 | Diffusion of Anti-PD-1 mAb from blood to lymph node |
| 149 | Lymphatic drainage of Anti-PD-1 mAb from tumor to lymph node |
| 150 | Diffusion of Anti-PD-L1 mAb from blood to lymph node |
| 151 | Lymphatic drainage of Anti-PD-L1 mAb from tumor to lymph node |
| 152 | The proliferation rate of the Proliferating T cells in the lymph node |
| 153 | Appearance of monocytes in the tumor |
| 154 | Proliferation of cancer cells in the tumor |
| 155 | Proliferation of effector T cells in the tumor |
| 156 | The natural turnover of arrested T cells in the peripheral tissues |
| 157 | Transmigration of effector T cells into peripheral tissues |
| 158 | The natural turnover of attached T cells in the peripheral tissues |
| 159 | Adhesion of attached T cell on vascular wall |
| 160 | Attachment and detachment of free effector T cells in the bloodstream in peripheral tissues |
| 161 | Lymphatic drainage of effector T cells from periphery to blood |
| 162 | CD80 expression by the T5 subtype of cancer cells in the tumor (expressing CD80 only) |
| 163 | PD-1 expression by the T2 subtype of cancer cells in the tumor (expressing PD-1 only) |
| 164 | PD-1 expression by the T9 subtype of cancer cells in the tumor (expressing CD80 and PD-1) |
| 165 | CD80 expression by the T9 subtype of cancer cells in the tumor (expressing CD80 and PD-1) |
| 166 | PD-1 expression by the T6 subtype of cancer cells in the tumor (expressing PD-L1 and PD-1) |
| 167 | PD-L1 expression by the T6 subtype of cancer cells in the tumor (expressing PD-L1 and PD-1) |
| 168 | PD-1 expression by the T14 subtype of cancer cells in the tumor (expressing CD80, PD-L1 and PD-1) |
| 169 | PD-L1 expression by the T14 subtype of cancer cells in the tumor (expressing CD80, PD-L1 and PD-1) |
| 170 | CD80 expression by the T14 subtype of cancer cells in the tumor (expressing CD80, PD-L1 and PD-1) |
| 171 | PD-1 expression by the T10 subtype of cancer cells in the tumor (expressing PD-L2, PD-L1 and PD-1) |
| 172 | PD-L1 expression by the T10 subtype of cancer cells in the tumor (expressing PD-L2, PD-L1 and PD-1) |
| 173 | PD-L2 expression by the T10 subtype of cancer cells in the tumor (expressing PD-L2, PD-L1 and PD-1) |
| 174 | PD-1 expression by the T16 subtype of cancer cells in the tumor (expressing CD80, PD-L2, PD-L1 and PD-1) |
| 175 | PD-L1 expression by the T16 subtype of cancer cells in the tumor (expressing CD80, PD-L2, PD-L1 and PD-1) |
| 176 | PD-L2 expression by the T16 subtype of cancer cells in the tumor (expressing CD80, PD-L2, PD-L1 and PD-1) |
| 177 | CD80 expression by the T16 subtype of cancer cells in the tumor (expressing CD80, PD-L2, PD-L1 and PD-1) |
| 178 | PD-1 expression by the T7 subtype of cancer cells in the tumor (expressing PD-L2 and PD-1) |
| 179 | PD-L2 expression by the T7 subtype of cancer cells in the tumor (expressing PD-L2 and PD-1) |
| 180 | PD-1 expression by the T15 subtype of cancer cells in the tumor (expressing CD80, PD-L2 and PD-1) |
| 181 | PD-L2 expression by the T15 subtype of cancer cells in the tumor (expressing CD80, PD-L2 and PD-1) |
| 182 | CD80 expression by the T15 subtype of cancer cells in the tumor (expressing CD80, PD-L2 and PD-1) |
| 183 | PD-L1 expression by the T3 subtype of cancer cells in the tumor (expressing PD-L1 only) |
| 184 | PD-L1 expression by the T11 subtype of cancer cells in the tumor (expressing CD80 and PD-L1) |
| 185 | CD80 expression by the T11 subtype of cancer cells in the tumor (expressing CD80 and PD-L1) |
| 186 | PD-L1 expression by the T8 subtype of cancer cells in the tumor (expressing PD-L2 and PD-L1) |
| 187 | PD-L2 expression by the T8 subtype of cancer cells in the tumor (expressing PD-L2 and PD-L1) |
| 188 | PD-L1 expression by the T13 subtype of cancer cells in the tumor (expressing CD80, PD-L2 and PD-L1) |
| 189 | PD-L2 expression by the T13 subtype of cancer cells in the tumor (expressing CD80, PD-L2 and PD-L1) |
| 190 | CD80 expression by the T13 subtype of cancer cells in the tumor (expressing CD80, PD-L2 and PD-L1) |
| 191 | PD-L2 expression by the T4 subtype of cancer cells in the tumor (expressing PD-L2 only) |
| 192 | PD-L2 expression by the T12 subtype of cancer cells in the tumor (expressing CD80 and PD-L2) |
| 193 | CD80 expression by the T12 subtype of cancer cells in the tumor (expressing CD80 and PD-L2) |
| 194 | PD-1 expression threshold by the T10 subtype of cancer cells in the tumor |
| 195 | Interaction at the immunological synapse between PD-1 expressed by the T10 subtype of cancer cells and Anti-PD-1 in the tumor |
| 196 | PD-L1 expression threshold by the T10 subtype of cancer cells in the tumor |
| 197 | Interaction at the immunological synapse between PD-L1 expressed by the T10 subtype of cancer cells and Anti-PD-L1 in the tumor |
| 198 | PD-L2 expression threshold by the T10 subtype of cancer cells in the tumor |
| 199 | PD-L1 expression threshold by the T11 subtype of cancer cells in the tumor |
| 200 | Interaction at the immunological synapse between PD-L1 expressed by the T11 subtype of cancer cells and Anti-PD-L1 in the tumor |
| 201 | CD80 expression threshold by the T11 subtype of cancer cells in the tumor |
| 202 | PD-L2 expression threshold by the T12 subtype of cancer cells in the tumor |
| 203 | CD80 expression threshold by the T12 subtype of cancer cells in the tumor |
| 204 | PD-L1 expression threshold by the T13 subtype of cancer cells in the tumor |
| 205 | Interaction at the immunological synapse between PD-L1 expressed by the T13 subtype of cancer cells and Anti-PD-L1 in the tumor |
| 206 | PD-L2 expression threshold by the T13 subtype of cancer cells in the tumor |
| 207 | CD80 expression threshold by the T13 subtype of cancer cells in the tumor |
| 208 | PD-1 expression threshold by the T14 subtype of cancer cells in the tumor |
| 209 | Interaction at the immunological synapse between PD-1 expressed by the T14 subtype of cancer cells and PD-L1 on the complementary Effector T cells in the tumor |
| 210 | PD-L1 expression threshold by the T14 subtype of cancer cells in the tumor |
| 211 | Interaction at the immunological synapse between PD-L1 expressed by the T14 subtype of cancer cells and CD80 on the complementary Effector T cells in the tumor |
| 212 | Interaction at the immunological synapse between PD-L1 expressed by the T14 subtype of cancer cells and Anti-PD-L1 mAb in the tumor |
| 213 | CD80 expression threshold by the T14 subtype of cancer cells in the tumor |
| 214 | Interaction at the immunological synapse between CD80 expressed by the T14 subtype of cancer cells and PD-L1 on the complementary Effector T cells in the tumor |
| 215 | PD-1 expression threshold by the T15 subtype of cancer cells in the tumor |
| 216 | Interaction at the immunological synapse between PD-1 expressed by the T15 subtype of cancer cells and Anti-PD-1 in the tumor |
| 217 | PD-L2 expression threshold by the T15 subtype of cancer cells in the tumor |
| 218 | Interaction at the immunological synapse between PD-L2 expressed by the T15 subtype of cancer cells and PD-1 on the complementary Effector T cells in the tumor |
| 219 | CD80 expression threshold by the T15 subtype of cancer cells in the tumor |
| 220 | Interaction at the immunological synapse between CD80 expressed by the T15 subtype of cancer cells and PD-L1 on the complementary Effector T cells in the tumor |
| 221 | PD-1 expression threshold by the T16 subtype of cancer cells in the tumor |
| 222 | Interaction at the immunological synapse between PD-1 expressed by the T16 subtype of cancer cells and PD-L1 on the complementary Effector T cells in the tumor |
| 223 | Interaction at the immunological synapse between PD-1 expressed by the T16 subtype of cancer cells and Anti-PD-1 in the tumor |
| 224 | PD-L1 expression threshold by the T16 subtype of cancer cells in the tumor |
| 225 | Interaction at the immunological synapse between PD-L1 expressed by the T16 subtype of cancer cells and Anti-PD-L1 in the tumor |
| 226 | PD-L2 expression threshold by the T16 subtype of cancer cells in the tumor |
| 227 | Interaction at the immunological synapse between PD-L2 expressed by the T16 subtype of cancer cells and PD-1 on the complementary Effector T cells in the tumor |
| 228 | CD80 expression threshold by the T16 subtype of cancer cells in the tumor |
| 229 | PD-1 expression threshold by the T2 subtype of cancer cells in the tumor |
| 230 | PD-L1 expression threshold by the T3 subtype of cancer cells in the tumor |
| 231 | PD-L2 expression threshold by the T4 subtype of cancer cells in the tumor |
| 232 | CD80 expression threshold by the T5 subtype of cancer cells in the tumor |
| 233 | PD-1 expression threshold by the T6 subtype of cancer cells in the tumor |
| 234 | Interaction at the immunological synapse between PD-L2 expressed by the T6 subtype of cancer cells and PD-1 on the complementary Effector T cells in the tumor |
| 235 | PD-L1 expression threshold by the T6 subtype of cancer cells in the tumor |
| 236 | Interaction at the immunological synapse between PD-L1 expressed by the T6 subtype of cancer cells and PD-1 on the complementary Effector T cells in the tumor |
| 237 | Interaction at the immunological synapse between PD-L1 expressed by the T6 subtype of cancer cells and CD80 on the complementary Effector T cells in the tumor |
| 238 | Interaction at the immunological synapse between PD-L1 expressed by the T6 subtype of cancer cells and Anti-PD-L1 in the tumor |
| 239 | PD-1 expression threshold by the T7 subtype of cancer cells in the tumor |
| 240 | PD-L2 expression threshold by the T7 subtype of cancer cells in the tumor |
| 241 | PD-L1 expression threshold by the T8 subtype of cancer cells in the tumor |
| 242 | Interaction at the immunological synapse between PD-L1 expressed by the T8 subtype of cancer cells and CD80 on Effector T cells in the tumor |
| 243 | Interaction at the immunological synapse between PD-L1 expressed by the T8 subtype of cancer cells and Anti-PD-L1 in the tumor |
| 244 | PD-L2 expression threshold by the T8 subtype of cancer cells in the tumor |
| 245 | PD-1 expression threshold by the T9 subtype of cancer cells in the tumor |
| 246 | Interaction at the immunological synapse between PD-1 expressed by the T9 subtype of cancer cells and PD-L1 on the complementary Effector T cells in the tumor |
| 247 | CD80 expression threshold by the T9 subtype of cancer cells in the tumor |
| 248 | Internalization of the Anti-CTLA-4 antibodies bound to CTLA-4 receptors on T Regulatory cells in the tumor |
| 249 | Transendocytosis of the CD80 receptors on mAPCs through CTLA-4 binding and internalization on T Regulatory cells in the tumor |
| 250 | Transendocytosis of the CD86 receptors on mAPCs through CTLA-4 binding and internalization on T Regulatory cells in the tumor |
| 251 | PD-1 expression by Effector T cells interacting with the T4 subtype of cancer cells in the tumor |
| 252 | PD-1 expression by Effector T cells interacting with the T8 subtype of cancer cells in the tumor |
| 253 | CD80 expression by Effector T cells interacting with the T8 subtype of cancer cells in the tumor |
| 254 | PD-1 expression by Effector T cells interacting with the T3 subtype of cancer cells in the tumor |
| 255 | CD80 expression by Effector T cells interacting with the T3 subtype of cancer cells in the tumor |
| 256 | PD-L1 expression by Effector T cells interacting with the T15 subtype of cancer cells in the tumor |
| 257 | PD-1 expression by Effector T cells interacting with the T15 subtype of cancer cells in the tumor |
| 258 | PD-L1 expression by Effector T cells interacting with the T7 subtype of cancer cells in the tumor |
| 259 | PD-1 expression by Effector T cells interacting with the T7 subtype of cancer cells in the tumor |
| 260 | PD-L1 expression by Effector T cells interacting with the T13 subtype of cancer cells in the tumor |
| 261 | PD-1 expression by Effector T cells interacting with the T13 subtype of cancer cells in the tumor |
| 262 | PD-L1 expression by Effector T cells interacting with the T11 subtype of cancer cells in the tumor |
| 263 | PD-1 expression by Effector T cells interacting with the T11 subtype of cancer cells in the tumor |
| 264 | PD-L1 expression by Effector T cells interacting with the T12 subtype of cancer cells in the tumor |
| 265 | PD-1 expression by Effector T cells interacting with the T12 subtype of cancer cells in the tumor |
| 266 | PD-L1 expression by Effector T cells interacting with the T16 subtype of cancer cells in the tumor |
| 267 | PD-1 expression by Effector T cells interacting with the T16 subtype of cancer cells in the tumor |
| 268 | CD80 expression by Effector T cells interacting with the T16 subtype of cancer cells in the tumor |
| 269 | PD-L1 expression by Effector T cells interacting with the T14 subtype of cancer cells in the tumor |
| 270 | PD-1 expression by Effector T cells interacting with the T14 subtype of cancer cells in the tumor |
| 271 | CD80 expression by Effector T cells interacting with the T14 subtype of cancer cells in the tumor |
| 272 | PD-L1 expression by Effector T cells interacting with the T10 subtype of cancer cells in the tumor |
| 273 | PD-1 expression by Effector T cells interacting with the T10 subtype of cancer cells in the tumor |
| 274 | CD80 expression by Effector T cells interacting with the T10 subtype of cancer cells in the tumor |
| 275 | PD-L1 expression by Effector T cells interacting with the T6 subtype of cancer cells in the tumor |
| 276 | PD-1 expression by Effector T cells interacting with the T6 subtype of cancer cells in the tumor |
| 277 | CD80 expression by Effector T cells interacting with the T6 subtype of cancer cells in the tumor |
| 278 | PD-L1 expression by Effector T cells interacting with the T9 subtype of cancer cells in the tumor |
| 279 | PD-L1 expression by Effector T cells interacting with the T5 subtype of cancer cells in the tumor |
| 280 | PD-L1 expression by Effector T cells interacting with the T2 subtype of cancer cells in the tumor |
| 281 | PD-L1 expression threshold by the Effector T cells interacting with the T10 subtype of cancer cells in the tumor |
| 282 | Interaction at the immunological synapse between PD-1 expressed by the T10 subtype of cancer cells and PD-L1 on the complementary Effector T cells in the tumor |
| 283 | Interaction at the immunological synapse between PD-L1 expressed by the Effector T cells interacting with the T10 subtype of cancer cells and Anti-PD-L1 in the tumor |
| 284 | PD-1 expression threshold by the Effector T cells interacting with the T10 subtype of cancer cells in the tumor |
| 285 | Interaction at the immunological synapse between PD-L1 expressed by the T10 subtype of cancer cells and PD-1 on the complementary Effector T cells in the tumor |
| 286 | Interaction at the immunological synapse between PD-L2 expressed by the T10 subtype of cancer cells and PD-1 on the complementary Effector T cells in the tumor |
| 287 | CD80 expression threshold by the Effector T cells interacting with the T10 subtype of cancer cells in the tumor |
| 288 | Interaction at the immunological synapse between PD-L1 expressed by the T10 subtype of cancer cells and CD80 on the complementary Effector T cells in the tumor |
| 289 | PD-L1 expression threshold by the Effector T cells interacting with the T11 subtype of cancer cells in the tumor |
| 290 | Interaction at the immunological synapse between PD-L1 expressed by the T11 subtype of cancer cells and CD80 on the complementary Effector T cells in the tumor |
| 291 | Interaction at the immunological synapse between PD-L1 expressed by the Effector T cells interacting with the T11 subtype of cancer cells and Anti-PD-L1 in the tumor |
| 292 | PD-1 expression threshold by the Effector T cells interacting with the T11 subtype of cancer cells in the tumor |
| 293 | Interaction at the immunological synapse between PD-L1 expressed by the T11 subtype of cancer cells and PD-1 on the complementary Effector T cells in the tumor |
| 294 | PD-L1 expression threshold by the Effector T cells interacting with the T12 subtype of cancer cells in the tumor |
| 295 | Interaction at the immunological synapse between CD80 expressed by the T12 subtype of cancer cells and PD-L1 on the complementary Effector T cells in the tumor |
| 296 | Interaction at the immunological synapse between PD-L1 expressed by the Effector T cells interacting with the T12 subtype of cancer cells and Anti-PD-L1 in the tumor |
| 297 | PD-1 expression threshold by the Effector T cells interacting with the T12 subtype of cancer cells in the tumor |
| 298 | Interaction at the immunological synapse between PD-L2 expressed by the T12 subtype of cancer cells and PD-1 on the complementary Effector T cells in the tumor |
| 299 | PD-L1 expression threshold by the Effector T cells interacting with the T13 subtype of cancer cells in the tumor |
| 300 | Interaction at the immunological synapse between CD80 expressed by the T13 subtype of cancer cells and PD-L1 on the complementary Effector T cells in the tumor |
| 301 | PD-1 expression threshold by the Effector T cells interacting with the T13 subtype of cancer cells in the tumor |
| 302 | Interaction at the immunological synapse between PD-L1 expressed by the T13 subtype of cancer cells and PD-1 on the complementary Effector T cells in the tumor |
| 303 | Interaction at the immunological synapse between PD-L2 expressed by the T13 subtype of cancer cells and PD-1 on the complementary Effector T cells in the tumor |
| 304 | Interaction at the immunological synapse between PD-1 expressed by the Effector T cells interacting with the T13 subtype of cancer cells and Anti-PD-1 in the tumor |
| 305 | PD-L1 expression threshold by the Effector T cells interacting with the T14 subtype of cancer cells in the tumor |
| 306 | Interaction at the immunological synapse between PD-L1 expressed by the Effector T cells interacting with the T14 subtype of cancer cells and Anti-PD-L1 in the tumor |
| 307 | PD-1 expression threshold by the Effector T cells interacting with the T14 subtype of cancer cells in the tumor |
| 308 | Interaction at the immunological synapse between PD-L1 expressed by the T14 subtype of cancer cells and PD-1 on the complementary Effector T cells in the tumor |
| 309 | CD80 expression threshold by the Effector T cells interacting with the T14 subtype of cancer cells in the tumor |
| 310 | PD-L1 expression threshold by the Effector T cells interacting with the T15 subtype of cancer cells in the tumor |
| 311 | Interaction at the immunological synapse between PD-1 expressed by the T15 subtype of cancer cells and PD-L1 on the complementary Effector T cells in the tumor |
| 312 | Interaction at the immunological synapse between PD-L1 expressed by the Effector T cells interacting with the T15 subtype of cancer cells and Anti-PD-L1 in the tumor |
| 313 | PD-1 expression threshold by the Effector T cells interacting with the T15 subtype of cancer cells in the tumor |
| 314 | Interaction at the immunological synapse between PD-1 expressed by the Effector T cells interacting with the T15 subtype of cancer cells and Anti-PD-1 in the tumor |
| 315 | PD-L1 expression threshold by the Effector T cells interacting with the T16 subtype of cancer cells in the tumor |
| 316 | Interaction at the immunological synapse between CD80 expressed by the T16 subtype of cancer cells and PD-L1 on the complementary Effector T cells in the tumor |
| 317 | Interaction at the immunological synapse between PD-L1 expressed by the Effector T cells interacting with the T16 subtype of cancer cells and Anti-PD-L1 in the tumor |
| 318 | PD-1 expression threshold by the Effector T cells interacting with the T16 subtype of cancer cells in the tumor |
| 319 | Interaction at the immunological synapse between PD-L1 expressed by the T16 subtype of cancer cells and PD-1 on the complementary Effector T cells in the tumor |
| 320 | Interaction at the immunological synapse between PD-1 expressed by the Effector T cells interacting with the T16 subtype of cancer cells and Anti-PD-1 in the tumor |
| 321 | CD80 expression threshold by the Effector T cells interacting with the T16 subtype of cancer cells in the tumor |
| 322 | Interaction at the immunological synapse between PD-L1 expressed by the T16 subtype of cancer cells and CD80 on the complementary Effector T cells in the tumor |
| 323 | PD-L1 expression threshold by the Effector T cells interacting with the T2 subtype of cancer cells in the tumor |
| 324 | Interaction at the immunological synapse between PD-1 expressed by the T2 subtype of cancer cells and PD-L1 on the complementary Effector T cells in the tumor |
| 325 | PD-1 expression threshold by the Effector T cells interacting with the T3 subtype of cancer cells in the tumor |
| 326 | Interaction at the immunological synapse between PD-L1 expressed by the T3 subtype of cancer cells and PD-1 on the complementary Effector T cells in the tumor |
| 327 | Interaction at the immunological synapse between PD-1 expressed by the Effector T cells interacting with the T3 subtype of cancer cells and Anti-PD-1 in the tumor |
| 328 | CD80 expression threshold by the Effector T cells interacting with the T3 subtype of cancer cells in the tumor |
| 329 | Interaction at the immunological synapse between PD-L1 expressed by the T3 subtype of cancer cells and CD80 on the complementary Effector T cells in the tumor |
| 330 | PD-1 expression threshold by the Effector T cells interacting with the T4 subtype of cancer cells in the tumor |
| 331 | Interaction at the immunological synapse between PD-L2 expressed by the T4 subtype of cancer cells and PD-1 on the complementary Effector T cells in the tumor |
| 332 | Interaction at the immunological synapse between PD-1 expressed by the Effector T cells interacting with the T4 subtype of cancer cells and Anti-PD-1 in the tumor |
| 333 | PD-L1 expression threshold by the Effector T cells interacting with the T5 subtype of cancer cells in the tumor |
| 334 | Interaction at the immunological synapse between CD80 expressed by the T5 subtype of cancer cells and PD-L1 on the complementary Effector T cells in the tumor |
| 335 | Interaction at the immunological synapse between PD-L1 expressed by the Effector T cells interacting with the T5 subtype of cancer cells and Anti-PD-L1 in the tumor |
| 336 | PD-L1 expression threshold by the Effector T cells interacting with the T6 subtype of cancer cells in the tumor |
| 337 | Interaction at the immunological synapse between PD-1 expressed by the T6 subtype of cancer cells and PD-L1 on the complementary Effector T cells in the tumor |
| 338 | Interaction at the immunological synapse between PD-L1 expressed by the Effector T cells interacting with the T6 subtype of cancer cells and Anti-PD-L1 in the tumor |
| 339 | PD-1 expression threshold by the Effector T cells interacting with the T6 subtype of cancer cells in the tumor |
| 340 | Interaction at the immunological synapse between PD-1 expressed by the Effector T cells interacting with the T6 subtype of cancer cells and Anti-PD-1 in the tumor |
| 341 | CD80 expression threshold by the Effector T cells interacting with the T6 subtype of cancer cells in the tumor |
| 342 | PD-L1 expression threshold by the Effector T cells interacting with the T7 subtype of cancer cells in the tumor |
| 343 | Interaction at the immunological synapse between PD-1 expressed by the T7 subtype of cancer cells and PD-L1 on the complementary Effector T cells in the tumor |
| 344 | PD-1 expression threshold by the Effector T cells interacting with the T7 subtype of cancer cells in the tumor |
| 345 | Interaction at the immunological synapse between PD-L2 expressed by the T7 subtype of cancer cells and PD-1 on the complementary Effector T cells in the tumor |
| 346 | PD-1 expression threshold by the Effector T cells interacting with the T8 subtype of cancer cells in the tumor |
| 347 | Interaction at the immunological synapse between PD-L1 expressed by the T8 subtype of cancer cells and PD-1 on the complementary Effector T cells in the tumor |
| 348 | Interaction at the immunological synapse between PD-L2 expressed by the T8 subtype of cancer cells and PD-1 on the complementary Effector T cells in the tumor |
| 349 | CD80 expression threshold by the Effector T cells interacting with the T8 subtype of cancer cells in the tumor |
| 350 | PD-L1 expression threshold by the Effector T cells interacting with the T9 subtype of cancer cells in the tumor |
| 351 | Interaction at the immunological synapse between CD80 expressed by the T9 subtype of cancer cells and PD-L1 on the complementary Effector T cells in the tumor |
| 352 | Interaction at the immunological synapse between PD-L1 expressed by the Effector T cells interacting with the T9 subtype of cancer cells and Anti-PD-L1 in the tumor |
| 353 | Turnover of resting macrophages |
| 354 | Transport of tumor debris from the tumor to the lymph node |
| 355 | Decay of tumor debris in the tumor |
| 356 | The distribution of tumor debris away from any of the considered lymph nodes |
| 357 | Accounting for the distribution of tumor debris from the tumor to the lymph nodes other than the lymph node compartment in the model |
| 358 | Phagocytosis of tumor debris by resting APCs in the lymph node |
| 359 | Natural turnover of cancer cells in the tumor |
| 360 | CD80 expression threshold by the mAPCs interacting with the T Regulatory cells in the tumor |
| 361 | CD80 expression threshold by the Effector T cells interacting with the T Regulatory cells in the tumor |
| 362 | Interaction at the immunological synapse between CD80 expressed by the Effector T cells and PD-L1 on the T Regulatory cells in the tumor |
| 363 | CD80 expression threshold by the Effector T cells interacting with the MDSCs in the tumor |
| 364 | CD86 expression threshold by the mAPCs interacting with the T Regulatory cells in the tumor |
| 365 | Lymphatic drainage of Anti-CTLA-4 mAb from tumor to lymph node |
| 366 | Binding of Anti-CTLA-4 to the CTLA-4 receptors expressed on the surface of non-interacting T Regulatory cells in the tumor |
| 367 | CTLA-4 expression threshold by the T Regulatory cells interacting with the mAPCs in the tumor |
| 368 | Interaction at the immunological synapse between CD80 expressed by the mAPCs and CTLA-4 on the T Regulatory cells in the tumor |
| 369 | Interaction at the immunological synapse between CD86 expressed by the mAPCs and CTLA-4 on the T Regulatory cells in the tumor |
| 370 | Interaction at the immunological synapse between CTLA-4 expressed by the T Regulatory cells and Anti-CTLA-4 in the tumor |
| 371 | CTLA-4 expression threshold by non-interacting T Regulatory cells in the tumor |
| 372 | Turnover of arrested effector T cells on vascular wall in tumor |
| 373 | Transmigration of arrested effector T cell into the tumor |
| 374 | Turnover of attached effector T cells on vascular wall in tumor |
| 375 | Adhesion of attached T cells onto the vascular wall in the tumor |
| 376 | Attachment and detachment of free effector T cells in vascular space in tumor |
| 377 | Turnover of Effector T cells in the tumor |
| 378 | Association of Effector T cells with cancer cells in the tumor |
| 379 | Turnover of Effector T cells in deeply exhausted state in the tumor |
| 380 | Transport of mAPCs from the tumor to the lymph node |
| 381 | Accounting for the distribution of mAPCs from the tumor to the lymph nodes other than the lymph node compartment in the model |
| 382 | The distribution of mAPCs away from any of the considered lymph nodes |
| 383 | CD80 expression by mAPCs interacting with the T Regulatory cells in the tumor |
| 384 | CD86 expression by mAPCs interacting with the T Regulatory cells in the tumor |
| 385 | Association of Effector T cells with MDSCs in the tumor |
| 386 | PD-1 expression by MDSCs interacting with the Effector T cells in the tumor |
| 387 | PD-L1 expression by MDSCs interacting with the Effector T cells in the tumor |
| 388 | Dissociation of MDSCs from Effector T cells without inactivation of the latter in the tumor |
| 389 | Dissociation of MDSCs from Effector T cells with inactivation of the latter in the tumor |
| 390 | Lymphatic drainage of Anti-PD-1 mAb from tumor to lymph node |
| 391 | Interaction at the immunological synapse between PD-1 expressed by the T14 subtype of cancer cells and Anti-PD-1 in the tumor |
| 392 | Interaction at the immunological synapse between PD-1 expressed by the T2 subtype of cancer cells and Anti-PD-1 in the tumor |
| 393 | Interaction at the immunological synapse between PD-1 expressed by the T7 subtype of cancer cells and Anti-PD-1 in the tumor |
| 394 | Interaction at the immunological synapse between PD-1 expressed by the T9 subtype of cancer cells and Anti-PD-1 in the tumor |
| 395 | Interaction at the immunological synapse between PD-1 expressed by the Effector T cells interacting with the T10 subtype of cancer cells and Anti-PD-1 in the tumor |
| 396 | Interaction at the immunological synapse between PD-1 expressed by the Effector T cells interacting with the T11 subtype of cancer cells and Anti-PD-1 in the tumor |
| 397 | Interaction at the immunological synapse between PD-1 expressed by the Effector T cells interacting with the T12 subtype of cancer cells and Anti-PD-1 in the tumor |
| 398 | Interaction at the immunological synapse between PD-1 expressed by the Effector T cells interacting with the T14 subtype of cancer cells and Anti-PD-1 in the tumor |
| 399 | Interaction at the immunological synapse between PD-1 expressed by the Effector T cells interacting with the T7 subtype of cancer cells and Anti-PD-1 in the tumor |
| 400 | Interaction at the immunological synapse between PD-1 expressed by the Effector T cells interacting with the T8 subtype of cancer cells and Anti-PD-1 in the tumor |
| 401 | Interaction at the immunological synapse between PD-1 expressed by the Effector T cells interacting with the T Regulatory cells and Anti-PD-1 in the tumor |
| 402 | PD-1 expression threshold by the MDSCs interacting with the Effector T cells in the tumor |
| 403 | Interaction at the immunological synapse between PD-1 expressed by the MDSCs interacting with Effector T cells and Anti-PD-1 in the tumor |
| 404 | Interaction at the immunological synapse between PD-L1 expressed by the Effector T cells and PD-1 on the MDSCs in the tumor |
| 405 | PD-1 expression threshold by the Effector T cells interacting with the T Regulatory cells in the tumor |
| 406 | Interaction at the immunological synapse between PD-1 expressed by the Effector T cells and PD-L1 on the T Regulatory cells in the tumor |
| 407 | PD-1 expression threshold by the Effector T cells interacting with the MDSCs in the tumor |
| 408 | Interaction at the immunological synapse between PD-1 expressed by the Effector T cells interacting with the MDSCs and Anti-PD-1 in the tumor |
| 409 | PD-1 expression threshold by the T Regulatory cells interacting with the Effector T cells in the tumor |
| 410 | Interaction at the immunological synapse between PD-1 expressed by the T Regulatory cells interacting with the Effector T cells and Anti-PD-1 in the tumor |
| 411 | Lymphatic drainage of Anti-PD-L1 mAb from tumor to lymph node |
| 412 | Interaction at the immunological synapse between PD-L1 expressed by the T3 subtype of cancer cells and Anti-PD-L1 in the tumor |
| 413 | Interaction at the immunological synapse between PD-L1 expressed by the Effector T cells interacting with the T13 subtype of cancer cells and Anti-PD-L1 in the tumor |
| 414 | Interaction at the immunological synapse between PD-L1 expressed by the Effector T cells interacting with the T2 subtype of cancer cells and Anti-PD-L1 in the tumor |
| 415 | Interaction at the immunological synapse between PD-L1 expressed by the Effector T cells interacting with the T7 subtype of cancer cells and Anti-PD-L1 in the tumor |
| 416 | Interaction at the immunological synapse between PD-L1 expressed by the Effector T cells interacting with the T Regulatory cells and Anti-PD-L1 in the tumor |
| 417 | Interaction at the immunological synapse between PD-L1 expressed by the Effector T cells interacting with the MDSCs and Anti-PD-L1 in the tumor |
| 418 | PD-L1 expression threshold by the MDSCs interacting with the Effector T cells in the tumor |
| 419 | Interaction at the immunological synapse between CD80 expressed by the Effector T cells and PD-L1 on the MDSCs in the tumor |
| 420 | Interaction at the immunological synapse between PD-1 expressed by the Effector T cells and PD-L1 on the MDSCs in the tumor |
| 421 | Interaction at the immunological synapse between PD-L1 expressed by the MDSCs interacting with the Effector T cells and Anti-PD-L1 in the tumor |
| 422 | PD-L1 expression threshold by the Effector T cells interacting with the T Regulatory cells in the tumor |
| 423 | Interaction at the immunological synapse between PD-L1 expressed by the Effector T cells and PD-1 on the T Regulatory cells in the tumor |
| 424 | PD-L1 expression threshold by the Effector T cells interacting with the MDSCs in the tumor |
| 425 | PD-L1 expression threshold by the T Regulatory cells interacting with the Effector T cells in the tumor |
| 426 | Interaction at the immunological synapse between PD-L1 expressed by the T Regulatory cells interacting with the Effector T cells and Anti-PD-L1 in the tumor |
| 427 | Dissociation of Effector T cells from the cancer cells without killing due to T cell exhaustion |
| 428 | Generation of cancer debris by killing of the cancer cells by the Effector T cells in the tumor |
| 429 | Effector T cell transform into a deeply exhausted state |
| 430 | CD80 expression by Effector T cells interacting with the MDSCs in the tumor |
| 431 | PD-1 expression by Effector T cells interacting with the MDSCs in the tumor |
| 432 | PD-L1 expression by Effector T cells interacting with the MDSCs in the tumor |
| 433 | CD80 expression by Effector T cells interacting with the T Regulatory cells in the tumor |
| 434 | PD-1 expression by Effector T cells interacting with the T Regulatory cells in the tumor |
| 435 | PD-L1 expression by Effector T cells interacting with the T Regulatory cells in the tumor |
| 436 | Association of Effector T cells with T Regulatory cells in the tumor |
| 437 | Association of mAPCs with T Regulatory cells in the tumor |
| 438 | CTLA-4 expression by T Regulatory cells interacting with the mAPCs in the tumor |
| 439 | PD-1 expression by T Regulatory cells interacting with the Effector T cells in the tumor |
| 440 | PD-L1 expression by T Regulatory cells interacting with the Effector T cells in the tumor |
| 441 | Dissociation of T Regulatory cells from mAPCs with inactivation of the latter in the tumor |
| 442 | Dissociation of T Regulatory cells from mAPCs without inactivation of the latter in the tumor |
| 443 | Dissociation of T Regulatory cells from Effector T cells with inactivation of the latter in the tumor |
| 444 | Dissociation of T Regulatory cells from Effector T cells without inactivation of the latter in the tumor |
| 445 | CTLA-4 expression on the surface of non-interacting T Regulatory cells in the tumor |

**Table S6 – Model Reaction and Rate Descriptions (End)**

**Table S7 – Model Parameters (Start)**

| Variable Number | Variable | Value | Units | Source | Description |
| --- | --- | --- | --- | --- | --- |
| 1 | %_NT_LN | 56 | dimensionless | [19, 20] | Percent Naïve T cells of total T cells in each lymph node (taken as all CD3+ cells) |
| 2 | %_Tr_LN | 6 | dimensionless | [19, 21-23] | Percent Regulatory T cells of total T cells in each lymph node |
| 3 | %{CD80}{PD1}C | Calculated | dimensionless | Calculated | Fraction of cancer cells expressing CD80 and PD-1 checkpoint receptors only |
| 4 | %{CD80}C | Calculated | dimensionless | Calculated | Fraction of cancer cells expressing the CD80 checkpoint receptor only |
| 5 | %{Other}C | Calculated | dimensionless | Calculated | Fraction of cancer cells expressing only unknown factors, other than CD80, PD-1, PD-L1 and PD-L2 |
| 6 | %{PD1}{80}{L1}{L2}C | Calculated | dimensionless | Calculated | Fraction of cancer cells expressing CD80, PD-1, PD-L1 and PD-L2 checkpoint receptors only |
| 7 | %{PD1}{80}{L1}C | Calculated | dimensionless | Calculated | Fraction of cancer cells expressing CD80, PD-1, and PD-L1 checkpoint receptors only |
| 8 | %{PD1}{80}{PDL2}C | Calculated | dimensionless | Calculated | Fraction of cancer cells expressing CD80, PD-1, and PD-L2 checkpoint receptors only |
| 9 | %{PD1}{L1}{L2}C | Calculated | dimensionless | Calculated | Fraction of cancer cells expressing PD-1, PD-L1 and PD-L2 checkpoint receptors only |
| 10 | %{PD1}{L1}C | Calculated | dimensionless | Calculated | Fraction of cancer cells expressing PD-1, and PD-L1 checkpoint receptors only |
| 11 | %{PD1}{L2}C | Calculated | dimensionless | Calculated | Fraction of cancer cells expressing PD-1, and PD-L2 checkpoint receptors only |
| 12 | %{PD1}C | Calculated | dimensionless | Calculated | Fraction of cancer cells expressing the PD-1 checkpoint receptor only |
| 13 | %{PDL1}{80}{L2}C | Calculated | dimensionless | Calculated | Fraction of cancer cells expressing CD80, PD-L1, and PD-L1 checkpoint receptors only |
| 14 | %{PDL1}{80}C | Calculated | dimensionless | Calculated | Fraction of cancer cells expressing CD80 and PD-L1 checkpoint receptors only |
| 15 | %{PDL1}{PDL2}C | Calculated | dimensionless | Calculated | Fraction of cancer cells expressing PD-L1, and PD-L2 checkpoint receptors only |
| 16 | %{PDL1}C | Calculated | dimensionless | Calculated | Fraction of cancer cells expressing the PD-L1 checkpoint receptor only |
| 17 | %{PDL2}{80}C | Calculated | dimensionless | Calculated | Fraction of cancer cells expressing CD80 and PD-L2 checkpoint receptors only |
| 18 | %{PDL2}C | Calculated | dimensionless | Calculated | Fraction of cancer cells expressing the PD-L2 checkpoint receptor only |
| 19 | %CD80_Exp_Cancer | 0 | dimensionless | Vary in different cancer types | Percent of cancer cells of total cancer cells that are CD80 positive that interact with the Effector T cells in the tumor compartment |
| 20 | %CD80_receptor_level_PNT | 10 | dimensionless | Estimated | Percent of maximum CD80 receptor expression on Primed Naive T cells in the lymph nodes |
| 21 | %Deep_Exhausted_T | 0.1 | dimensionless | Estimated | Percentage of effector T cells that will transform into a deeply exhausted state |
| 22 | %PD1_Exp_Cancer | 0 | dimensionless | Vary in different cancer types | Percent of cancer cells of total cancer cells that are PD-1 positive that interact with the Effector T cells in the tumor compartment |
| 23 | %PD1_receptor_level_PNT | 25 | dimensionless | [24]  Estimated | Percent of maximum PD-1 receptor expression on Primed Naive T cells in the lymph nodes |
| 24 | %PDL1_Exp_Cancer | 33/59 for ER+/TNBC | dimensionless | [25]  Estimated | Percent of cancer cells of total cancer cells that are PD-L1 positive that interact with the Effector T cells in the tumor compartment |
| 25 | %PDL1_receptor_level_PNT | 25 | dimensionless | Estimated | Percent of maximum PD-L1 receptor expression on Primed Naive T cells in the lymph nodes |
| 26 | %PDL2_Exp_Cancer | 37 | dimensionless | [26]  Estimated | Percent of cancer cells of total cancer cells that are PD-L2 positive that interact with the Effector T cells in the tumor compartment |
| 27 | %Sig_Inhibit_Cancer | 0.85 | dimensionless | Estimated | The percent effect that total immune checkpoints on the cancer cells have on inactivating the Effector T cells in the tumor |
| 28 | %T_MDSCs_per_Cancer | 0.05 | dimensionless | [27]  Estimated | Defines the percentage of MDSCs in the tumor compartment as a percent of total tumor cells |
| 29 | %T_Tregs_per_Cancer | 0.05 | dimensionless | [27]  Estimated | Defines the percentage of Regulatory T cells in the tumor compartment as a percent of total tumor cells |
| 30 | Antigen_Intensity | Assigned from 0.10-1.0 | dimensionless | Estimated | The strength of the tumor antigens that are involved in priming of the T cells in the lymph nodes |
| 31 | AR_P | 2.70E-03 | 1/minute | [17] | Arrest rate of attached effector T cells on vascular wall in peripheral tissues |
| 32 | AR_T | 1 | 1/minute | [17] | Arrest rate of attached effector T cells on vascular wall in tumor |
| 33 | Avogadro's_Num | 6.022E+23 | molecules  /mole | Exact value | Avogadro’s number – converts receptor numbers to moles |
| 34 | B_P | 500000000 | mole/mL | [17] | ad hoc adhesion site density in periphery |
| 35 | B_T | 5E+11 | mole/liter | [17] | ad hoc adhesion site density in tumor |
| 36 | BodyWeight (kg) | 75 | kg | [28] | Body weight of an average human being |
| 37 | Cancer_Cell_Diam_um | 17 | µm | [29]  Estimated | Diameter of each cancer cell when considering each cell’s volume to be represented by a sphere |
| 38 | Cancer_Cell_Vol_mm3 | 9.0478E-7 | mm^3^ | Calculated | Volume of each cancer cell when the diameter is that of a sphere |
| 39 | Cancer_Diam_mm | Calculated | mm | Calculated | Diameter of the entire tumor – considering all cells and a void fraction |
| 40 | Cancer_max | 3.7E+12 | mole | [30]  Estimate | Maximal number of cancer cells in the tumor |
| 41 | Cancer_mm_Start_Therapy | Assigned | mm | Assigned | Diameter of the tumor when to start the therapeutic regimen |
| 42 | Cancer_per_T_Cell_Int | 1 | cell/cell | Estimated | Number of cancer cells that interact with each Effector T cell in the model |
| 43 | Cancer_per_T_Cell_max | 4 | cell/cell | [31, 32]  Estimated | Maximum number of cancer cells that can interact with each Effector T cell in the model; assuming that each Effector T cell uses 25% of its surface area to interact with each cancer cell (can be up to 50%) [32]. |
| 44 | Cancer_Vol_cm3 | Calculated | cm^3^ | Calculated | Volume of the entire tumor – considering all cells and a void fraction |
| 45 | CancerTEng | 2.079 | 1/hour | [33] Estimated | Rate constant defining the half-life of engagement between cancer cells and Effector T cells in the tumor (at a migration rate of 5-10 µm/min, T cells encounter and engage with a cancer cell approximately every 15 minutes to 1 hour). |
| 46 | CancerTInt | 0.03465 | 1/minute | [33] | Rate constant defining the half-life of dissociation between Effector T cells and cancer cells (thus, determining total time of interaction) |
| 47 | CD28_POS-Sig_NT | Calculated | dimensionless | Calculated | Fraction of total CD28 receptors on Naïve T cells that are involved in interacting with the CD80 and CD86 receptors on mAPCs during priming in the lymph nodes |
| 48 | CD28_POS-to-Total | Calculated | dimensionless | Calculated | Fraction of total CD28 receptors on Primed Naïve T cells that are involved in interacting with the CD80 and CD86 receptors on mAPCs during priming in the lymph nodes |
| 49 | CD28_receptors-per-Tcell | 15000 | molecules/cell | [34] | Number of CD28 receptors expressed on each T cell during priming |
| 50 | CD80_PNT_NEG-to-Max | Calculated | dimensionless | Calculated | Fraction of total CD80 receptors on Primed Naïve T cells that are involved in interacting with the PD-L1 receptors on mAPCs during priming in the lymph nodes |
| 51 | CD80_receptors_per_C_Cl | 30000 | molecules/cell | Estimated | Number of CD80 receptors expressed on each cancer cell (assumed same as for T cells). |
| 52 | CD80_receptors-per-mAPC | 130000 | molecules/cell | [35] | Maximum number of CD80 receptors expressed on each mAPC |
| 53 | CD80_receptors-per-Tcell | 30000 | molecules/cell | [36, 37]  Estimated | Number of CD80 receptors expressed on each T cell (by acquisition of CD80 from mAPCs over two rounds of priming) [37]. |
| 54 | CD80Sig_Tr-PNT | Calculated | dimensionless | Calculated | Fraction of total CD80 receptors on Primed Naïve T cells that are involved in interacting with the PD-L1 receptors on Regulatory T cells during priming in the lymph nodes |
| 55 | CD86_receptors-per-mAPC | 208000 | molecules/cell | [35] | Maximum number of CD86 receptors expressed on each mAPC |
| 56 | Copies-per-T_Cell_Clone | 100 | dimensionless | [38-40] | Number of copies of each Naïve T cell clone in the lymph nodes (taken as the average naïve T cells clone size). |
| 57 | CTLA4_change_schedule | Based on regimen | day | [28]  Based on regimen | Time in simulation to switch schedule in regimen for Anti-CTLA-4 therapy |
| 58 | CTLA4_counter_off | Based on regimen | day | [28]  Based on regimen | Time in simulation to stop Anti-CTLA-4 dose |
| 59 | CTLA4_counter_on | Based on regimen | day | [28]  Based on regimen | Time in simulation to start Anti-CTLA-4 dose |
| 60 | CTLA4_DoseSet | 1 | dimensionless | [28]  Based on regimen | Dose of Anti-CTLA-4 mAb |
| 61 | CTLA4_NEG-to-Total | Calculated | dimensionless | Calculated | Fraction of total CTLA-4 receptors on Primed Naïve T cells that are involved in interacting with the CD80 and CD86 receptors on mAPCs during priming in the lymph nodes |
| 62 | CTLA4_receptors-Int-PNT | Calculated | molecules/cell | [41]  Calculated | Maximum number of CTLA-4 receptors that can be recruited to the immunological synapse by Primed Naive T cells when interacting with mAPCs |
| 63 | CTLA4_receptors-nInt-PNT | Calculated | molecules/cell | [42]  Calculated | Number of CTLA-4 receptors that are expressed on the surface of non-interacting Primed Naive T cells |
| 64 | CTLA4_receptors-Tr | Calculated | molecules/cell | Calculated | Maximum number of CTLA-4 receptors on T-regulatory cells |
| 65 | CTLA4mAb | Based on regimen | mg/kg | [43]  Based on regimen | Dose of Anti-CTLA-4 being delivered |
| 66 | CTLA4mAb_Dose | Based on regimen | mg/kg | [43]  Based on regimen | Dose of Anti-CTLA-4 to deliver based on the current schedule |
| 67 | CTLA4mAb_New_Dose | Based on regimen | mg/kg | [43]  Based on regimen | Dose of Anti-CTLA-4 to be delivered when schedule is changed |
| 68 | CTLA4S_Molec_per_Sec_TregLN | 10 | 1/second | [21]  Estimated | Rate of CTLA-4 constitutively secreted by T Regulatory cells in the lymph nodes (based on IL-2 secretion rate) |
| 69 | CTLA4Sig_Secrete | Calculated | dimensionless | Calculated | Fraction of CD80 and CD86 receptors on non-interacting mAPCs that are occupied by CTLA-4 secreted from T Regulatory cells in the lymph nodes |
| 70 | CTLA4Sig_Secrete_max | 6E-13 | mole | Estimated | Maximum level of CTLA-4 secretion by T Regulatory cells in the lymph nodes |
| 71 | CTLA4Sig_TrLN | Calculated | dimensionless | Calculated | Fraction of CTLA-4 receptors expressed on T Regulatory cells that are occupied by Anti-CTLA-4 mAb in the lymph nodes |
| 72 | CTLA4Sig_TrLN-mAPC | Calculated | dimensionless | Calculated | Fraction of CTLA-4 receptors expressed on T Regulatory cells that are interacting with CD80 and CD86 on the mAPCs in the lymph nodes |
| 73 | CTLA4-to-CD28_Ratio_Int | Calculated | dimensionless | [41]  Calculated | Ratio of CTLA-4 to CD28 expression on interacting Primed Naive T cells |
| 74 | CTLA4-to-CD28_Ratio_nInt | 0.035 | dimensionless | [42] | Ratio of CTLA-4 to CD28 expression on non-interacting Primed Naive T cells |
| 75 | CTLA4-to-CD28_Ratio_Tr | 0.5 | dimensionless | [44] | CTLA-4 expression levels on T Regulatory cells relative to CD28 expression on Primed Naive T cells |
| 76 | day | 1 | day | Unit assignment | Assigns value of 1 day to the term day in the model |
| 77 | Debris_Decay | 2 | 1/day | [45, 46] | Half-life of tumor debris and non-phagocytosed tumor antigens |
| 78 | Debris_Transport | 3 | 1/day | [45] | Transport rate of tumor debris by the lymphatics to the lymph nodes (based on "permeation of tumor debris and the blood") |
| 79 | Dose_sched_CTLA4 | Based on regimen | day | [43]  Based on regimen | Time between sequential doses of Anti-CTLA-4 in a regimen |
| 80 | Dose_sched_PD1 | Based on regimen | day | Based on regimen | Time between sequential doses of Anti-PD-1 in a regimen |
| 81 | Dose_sched_PDL1 | Based on regimen | day | [43]  Based on regimen | Time between sequential doses of Anti-PD-L1 in a regimen |
| 82 | Durvalumab_MW | 146300000 | mg/mole | [47] | Molecular weight of Anti-PD-L1 antibody, Durvalumab |
| 83 | EffT_InOutLN | 0.0693 | 1/hour | [48] | Rate constant defining the half-life of Naïve T cell migration into and out of the lymph nodes |
| 84 | EffT_Migrate | 0.9 | 1/day | [45, 49] | Rate constant defining the half-life of Effector T cell migration from the lymph nodes to the blood |
| 85 | EffT_Turnover | 0.02 | 1/day | [45, 46, 50, 51] | Rate constant defining the half-life of Effector T cells |
| 86 | Endo_CTLA4 | 0.3465 | 1/minute | [52, 53] | Rate constant defining the half-life of (trans)endocytosis of CTLA-4 on T cells |
| 87 | EndTherapy | 1 | dimensionless | Assigned | Parameter used to define when to stop therapy after tumor becomes smaller than a certain size |
| 88 | Exp_All_mAPCLN | 69.3 | 1/second | Estimated | Rate constant defining the half-life of expression of receptors on mAPCs that are part of the mAPC count |
| 89 | Exp_CD28/80/86/PD1/L1/L2 | 2.772 | 1/minute | [41, 54] | Rate constant defining the half-life of expression of CD28, CD80, CD86, PD-1, PD-L1 and PD-L2 to the immunological synapse by all interacting T cells, mAPCs and cancer cells |
| 90 | Exp_CTLA4 | 0.0462 | 1/minute | [55] | Rate constant defining the half-life of expression of CTLA-4 to the immunological synapse by all interacting T cells |
| 91 | Frac_CD80_Exp_Cancer | Calculated | dimensionless | Calculated | Fraction form of %CD80_Exp_Cancer |
| 92 | Frac_PD1_Exp_Cancer | Calculated | dimensionless | Calculated | Fraction form of %PD1_Exp_Cancer |
| 93 | Frac_PDL1_Exp_Cancer | Calculated | dimensionless | Calculated | Fraction form of %PDL1_Exp_Cancer |
| 94 | Frac_PDL2_Exp_Cancer | Calculated | dimensionless | Calculated | Fraction form of %PDL2_Exp_Cancer |
| 95 | Tremelimumab_MW | 1.463805E8 | mg/mole | Exact number | Molecular weight of Anti-CTLA-4 antibody, Tremelimumab |
| 96 | IS_Scaling | 2 | dimensionless | [56, 57] | Sets the immunological synapse diameter to 30nm, from 15nm. |
| 97 | J_P | 2.90E-03 | 1/minute | [17] | Transmigration rate of arrested effector T cells into peripheral tissues |
| 98 | J_T | 2.90E-03 | 1/minute | [17] | Transmigration rate of arrested effector T cells into the tumor |
| 99 | k_DoseAdmin_AntiCTLA4 | 1 | 1/hour | [5]  Assigned | Zero-order rate constant for the delivery of Anti-CTLA-4 into the central compartment for a designated dose |
| 100 | k_DoseAdmin_AntiPDL1 | 1 | 1/hour | [6]  Assigned | Zero-order rate constant for the delivery of Anti-PD-L1 into the central compartment for a designated dose |
| 101 | k_DoseAdmin_AntiPD1 | 1 | 1/hour | [28]  Assigned | Zero-order rate constant for the delivery of Anti-PD-1 into the central compartment for a designated dose |
| 102 | kf_APC_turnover | 0.462 | 1/day | [58] | Half-life of mAPC turnover in the lymph nodes |
| 103 | kf_CanDecay | 0.001 | 1/day | [45, 46] | Rate constant defining the half-life decay of cancer cells by natural death |
| 104 | kf_Monocytes_intoT | 0.0231 | 1/minute | [59] | Rate constant defining the half-life of the appearance of monocytes in the tumor following the appearance of tumor antigens |
| 105 | kf_no_prolif | 99.72 | 1/day | Estimated | Rate constant defining the half-life for the fraction of Primed Naive T cells that will not successfully undergo the second phase of priming, and will therefore be considered anergic |
| 106 | kf_P | 3.10E-06 | milliliter/min/mole | [17] | Attachment rate of free effector T cells in vascular space in peripheral tissues |
| 107 | kf_Phase2P | 0.1155 | 1/hour | [60-62]  Estimated | Rate constant defining the approximate half-life of the entire second phase of priming in the lymph nodes, considering that the Primed Naïve T cells are\in the deep T-cell areas in the lymph node. This, along with the second priming phase, accounts for T cells starting to proliferate 1.5-2 days following the encounter of antigens on mAPCs [62]. |
| 108 | kf_Prolif_end | 15000 | 1/day | [32, 61, 63]  Estimated | Rate constant defining the half-life for conversion of proliferating Naive T cells into Effector T cells in the lymph nodes. The T cells were optimized to account for 3 divisions per day over 5 days. |
| 109 | kf_RestingMacrophage | 0.01 | 1/day | [45, 49] | Rate constant defining the half-life for turnover of resting macrophages |
| 110 | kf_T | 6.90E-09 | milliliter/min/mole | [17] | Attachment rate of free effector T cells in vascular space in tumor |
| 111 | kf_TregLNS_Inact | 0.07557 | 1/hour | Estimated | Rate constant defining the half-life of inactivation of mAPCs in the lymph nodes resulting from secreted CTLA-4 from T Regulatory cells interacting with CD80 and CD86 receptors on mAPCs |
| 112 | kg | 1 | kilogram | Unit assignment | Assigns value of 1 kilogram to the term kg in the model |
| 113 | koff_CD28_CD80 | 1.6 | 1/second | [64] | Dissociation rate constant for the interaction of CD28 with CD80 |
| 114 | koff_CD28_CD86 | 28 | 1/second | [64] | Dissociation rate constant for the interaction of CD28 with CD86 |
| 115 | koff_CTLA4_CD80 | 0.43 | 1/second | [64] | Dissociation rate constant for the interaction of CTLA-4 with CD80 |
| 116 | koff_CTLA4_CD86 | 5.1 | 1/second | [64] | Dissociation rate constant for the interaction of CTLA-4 with CD86 |
| 117 | koff_CTLA4mAb_CTLA4 | 0.0018 | 1/second | [65] | Dissociation rate constant for the interaction of CTLA-4 with Tremelimumab |
| 118 | koff_PD1_PDL1 | 1.44 | 1/second | [66] | Dissociation rate constant for the interaction of PD-1 with PD-L1 |
| 119 | koff_PD1_PDL2 | 0.55 | 1/second | [66] | Dissociation rate constant for the interaction of PD-1 with PD-L2 |
| 120 | koff_PD1-PD1mAb | 6.75e-6 | 1/second | [67] | Dissociation rate constant for the interaction of PD-1 with Pembrolizumab  (given a Kd of 27.0 pM) |
| 121 | koff_PDL1_CD80 | 5.94 | 1/second | [66] | Dissociation rate constant for the interaction of PD-L1 with CD80 |
| 122 | koff_PDL1-PDL1mAb | 0.000285 | 1/second | [68] | Dissociation rate constant for the interaction of PD-L1 with Durvalumab |
| 123 | kon_CD28_CD80 | 660000 | 1/(molarity*second) | [64] | Association rate constant for the interaction of CD28 with CD80 |
| 124 | kon_CD28_CD86 | 1400000 | 1/(molarity*second) | [64] | Association rate constant for the interaction of CD28 with CD86 |
| 125 | kon_CTLA4_CD80 | 2150000 | 1/(molarity*second) | [64] | Association rate constant for the interaction of CTLA-4 with CD80 |
| 126 | kon_CTLA4_CD86 | 1960000 | 1/(molarity*second) | [64] | Association rate constant for the interaction of CTLA-4 with CD86 |
| 127 | kon_CTLA4mAb_CTLA4 | 308000 | 1/(molarity*second) | [65] | Association rate constant for the interaction of CTLA-4 with Tremelimumab |
| 128 | kon_PD1_PDL1 | 184000 | 1/(molarity*second) | [66] | Association rate constant for the interaction of PD-1 with PD-L1 |
| 129 | kon_PD1_PDL2 | 250000 | 1/(molarity*second) | [66] | Association rate constant for the interaction of PD-1 with PD-L2 |
| 130 | kon_PD1-PD1mAb | 250000 | 1/(molarity*second) | [67] | Association rate constant for the interaction of PD-1 with Pembrolizumab  (given a Kd of 27.0 pM) |
| 131 | kon_PDL1_CD80 | 316000 | 1/(molarity*second) | [66] | Association rate constant for the interaction of PD-L1 with CD80 |
| 132 | kon_PDL1-PDL1mAb | 408000 | 1/(molarity*second) | [68] | Association rate constant for the interaction of PD-L1 with Durvalumab |
| 133 | kr_P | 200 | 1/minute | [17] | Detachment rate of attached effector T cells in vascular space in peripheral tissues |
| 134 | kr_T | 200 | 1/minute | [17] | Detachment rate of attached effector T cells in vascular space in tumor |
| 135 | L | 1 | liter | Unit assignment | Assigns value of 1 liter to the term L in the model |
| 136 | mAPC_activation_level | 1 | dimensionless | [69]  Defined | The fraction of CD80 and CD86 expression on the mAPCs from 0-1. |
| 137 | mAPC_Debis_T_Inact | 0.2 | dimensionless | Estimated | The fraction of tumor debris that is considered to not be transported to the lymph nodes, and is removed from the system |
| 138 | mAPC_Migrate | 0.2 | 1/day | [45, 49, 70] | Maximum migration rate of mAPCs |
| 139 | mAPC_per_T_cell | 10 | cell/cell | [32, 71-73]  Estimated | Maximum number of mAPCs that can interact with each Naive and Primed Naive T cell. Note: all T cells were estimated to be the same size, and a surface area analysis was used for this estimation. |
| 140 | mAPC_per_Tr_cell | 10 | cell/cell | [32, 71-73]  Estimated | Maximum number of mAPCs that can interact with each Effector T cell. Note: all T cells were estimated to be the same size, and a surface area analysis was used for this estimation. |
| 141 | mAPC50_per_T_cell | 3 | cell/cell | [48]  Estimated | Number of mAPCs that should interact with each Naive and Primed Naive T cell to achieve a 50% priming rate |
| 142 | Max_#Cells_per_mm^3 | 1.105242E+6 | cells/mm^3^ | Calculated | Maximum number of cells per mm^3^ in the tumor based on volumetric analysis (volume of tumor cells/total tumor volume) cannot exceed 1 at any given time). Current value given for 12µm cells considered as spheres, keeping tumor void fraction relevant to melanoma tumors |
| 143 | MDSCs_per_Treg | 3 | cell/cell | [27]  Estimated | The ratio of MDSCs per T Regulatory cells in the tumor |
| 144 | mg | 1 | milligram | Unit assignment | Assigns value of 1 miligram to the term mg in the model |
| 145 | min | 1 | minute | Unit assignment | Assigns value of 1 minute to the term min in the model |
| 146 | mole | 1 | mole | Unit assignment | Assigns value of 1 mole to the term mole in the model |
| 147 | New_sched_CTLA4 | Based on regimen | day | [43]  Based on regimen | New time between sequential doses of Anti-CTLA-4 in a regimen |
| 148 | New_sched_PD1 | Based on regimen | day | Based on regimen | New time between sequential doses of Anti-PD-1 in a regimen |
| 149 | New_sched_PDL1 | Based on regimen | day | [43]  Based on regimen | New time between sequential doses of Anti-PD-L1 in a regimen |
| 150 | Pembrolizumab_MW | 143600000 | mg/mole | Exact number | Molecular weight of Anti-PD-1 antibody, Pembrolizumab |
| 151 | Num_TDLN_Considered | 17 | dimensionless | [74-77]  Estimated | The number of total different lymph nodes (or lobules receiving a variety of antigens) in a region near the tumor (can be >9cm) that can produce an immune response against the tumor. |
| 152 | PD1_change_schedule | Based on regimen | day | Based on regimen | Time in simulation to switch schedule in regimen for Anti-PD-1 therapy |
| 153 | PD1_counter_off | Based on regimen | day | Based on regimen | Time in simulation to stop Anti-PD-1 dose |
| 154 | PD1_counter_on | Based on regimen | day | Based on regimen | Time in simulation to start Anti-PD-1 dose |
| 155 | PD1_DoseSet | Based on regimen | dimensionless | Based on regimen | Dose of Anti-PD-1 mAb |
| 156 | PD1_PNT_NEG-to-Max | Calculated | dimensionless | Calculated | Fraction of total PD-1 receptors on Primed Naïve T cells that are involved in interacting with the PD-L1 and PD-L2 receptors on mAPCs during priming in the lymph nodes |
| 157 | PD1_receptors_per_C_Cl | 9288 | molecules/cell | Estimated | Number of PD-1 receptors expressed on each cancer cell |
| 158 | PD1_receptors-per-mAPC | 9288 | molecules/cell | Estimated | Maximum number of PD-1 receptors expressed on each mAPC (maximum taken as max from mAPC levels) |
| 159 | PD1_receptors-per-Tcell | 3096 | molecules/cell | [66] | Maximum number of PD-1 receptors expressed on each T cell |
| 160 | PD1mAb | Based on regimen | milligram/kilogram | [43]  Based on regimen | Dose of Anti-PD-1 being delivered |
| 161 | PD1mAb_Dose | Based on regimen | milligram/kilogram | Based on regimen | Dose of Anti-PD-1 to deliver based on the current schedule |
| 162 | PD1mAb_New_Dose | Based on regimen | milligram/kilogram | [43]  Based on regimen | Dose of Anti-PD-1 to be delivered when schedule is changed |
| 163 | PD1Sig_Tr-PNT | Calculated | dimensionless | Calculated | Fraction of total PD-1 receptors on Primed Naïve T cells that are involved in interacting with the PD-L1 receptors on Regulatory T cells during priming in the lymph nodes |
| 164 | PDL1_change_schedule | Based on regimen | day | [43]  Based on regimen | Time in simulation to switch schedule in regimen for Anti-PD-L1 therapy |
| 165 | PDL1_counter_off | Based on regimen | day | [43]  Based on regimen | Time in simulation to stop Anti-PD-L1 dose |
| 166 | PDL1_counter_on | Based on regimen | day | [43]  Based on regimen | Time in simulation to start Anti-PD-L1 dose |
| 167 | PDL1_DoseSet | Based on regimen | dimensionless | [43]  Based on regimen | Dose of Anti-PD-L1 mAb |
| 168 | PDL1_PNT_NEG-to-Max | Calculated | dimensionless | Calculated | Fraction of total PD-L1 receptors on Primed Naïve T cells that are involved in interacting with the PD-1 and CD80 receptors on mAPCs during priming in the lymph nodes |
| 169 | PDL1_receptors_per_C_Cl | 80372 | molecules/cell | [66] | Number of PD-L1 receptors expressed on each cancer cell (maximum taken as max from mAPC levels) |
| 170 | PDL1_receptors-per-mAPC | 80372 | molecules/cell | [66] | Maximum number of PD-L1 receptors expressed on each mAPC |
| 171 | PDL1_receptors-per-Tcell | 9282 | molecules/cell | [66] | Maximum number of PD-L1 receptors expressed on each T cell |
| 172 | PDL1mAb | Based on regimen | milligram/kilogram | [43]  Based on regimen | Dose of Anti-PD-L1 being delivered |
| 173 | PDL1mAb_Dose | Based on regimen | milligram/kilogram | [43]  Based on regimen | Dose of Anti-PD-L1 to deliver based on the current schedule |
| 174 | PDL1mAb_New_Dose | Based on regimen | milligram/kilogram | [78]  Based on regimen | Dose of Anti-PD-L1 to be delivered when schedule is changed |
| 175 | PDL1Sig_Tr-PNT | Calculated | dimensionless | Calculated | Fraction of total PD-L1 receptors on Primed Naïve T cells that are involved in interacting with the PD-1 receptors on Regulatory T cells during priming in the lymph nodes |
| 176 | PDL2_receptors_per_C_Cl | 5243 | molecules/cell | [66] | Number of PD-L2 receptors expressed on each cancer cell (maximum taken as max from mAPC levels) |
| 177 | PDL2_receptors-per-mAPC | 5243 | molecules/cell | [66] | Maximum number of PD-L2 receptors expressed on each mAPC |
| 178 | phi | 3.96E-01 | 1/day | [17] | Uninhibited proliferation rate of effector T cells in the tumor (In this study, T cell proliferation is considered). |
| 179 | Phago_Debris | 1E-7 | 1/(mole*day) | [45, 49] | Tumor antigen uptake rate by APCs |
| 180 | Precursor_Frequen_NT | Calculated | dimensionless | [73, 79]  Calculated | Calculates the ratio of available Naive T cells for priming phase 1 to total T cells in the lymph nodes |
| 181 | Precursor_Frequen_PNT | Calculated | dimensionless | Calculated | Calculates the ratio of available Primed Naive T cells for priming phase 2 to total T cells in the lymph nodes |
| 182 | PrimeNT_rate | 0.05775 | 1/hour | [60-62] | Rate constant defining the approximate half-life of the entire first phase of priming in the lymph nodes, including the priming of all of the superficial Naïve T cells and those in the deep T-cell areas in the lymph node. This, along with the second priming phase, accounts for T cells starting to proliferate 1.5-2 days following the encounter of antigens on mAPCs [62]. |
| 183 | PrimeNT1_rate | 2.772 | 1/hour | [60, 61, 80] | Rate constant defining the approximate half-life of each interaction between Naive T cells and mAPCs during the first phase of priming in the lymph nodes, including the formation time of mature synapses between the Naïve T cells and the mAPCs. |
| 184 | PrimeTLN2_rate | 1.386 | 1/hour | [60, 61] | Rate constant defining the approximate half-life of each interaction between Primed Naive T cells and mAPCs during the second phase of priming in the lymph nodes, which was dominated by stable T-cell-mAPC interactions. |
| 185 | Prob_NT-mAPC_Interact | Calculated | dimensionless | [73] Calculated | Calculates the probability in a deterministic manner that at least one successful priming interaction will occur between Naive T cells and mAPCs during the first phase of priming |
| 186 | Prob_PNT-mAPC_Interact | Calculated | dimensionless | [73]  Calculated | Calculates the probability in a deterministic manner that at least one successful priming interaction will occur between Primed Naive T cells and mAPCs during the second phase of priming |
| 187 | Prolif_Fract | 718.30381081261 | 1/hour | [32, 61, 63]  Estimated | Proliferation threshold for fully primed and activated T cells in the lymph nodes that are undergoing proliferation. The T cells were optimized to account for 3 divisions per day over 5 days. |
| 188 | Prolif_Thresh | 93.32288149744991 | 1/hour | [32, 61, 63]  Estimated | Proliferation level for fully primed and activated T cells in the lymph nodes that are undergoing proliferation. The T cells were optimized to account for 3 divisions per day over 5 days. |
| 189 | Q_T | 2.22579 | milliliter/minute | [81]  Calculated | Median flow rate of blood by which Effector T cells circulate from blood to the tumor |
| 190 | Q_P | 5.93 | liter/minute | [17]  Calculated | Flow rate of blood in an average human being by which Effector T cells circulate from blood to periphery |
| 191 | Rate_Tumor_Growth | 241/103  for ER+/TNBC | 1/day | [82]  Estimated | Proliferation rate of cancer cells that defines the volumetric doubling time of the tumor.  241-day doubling: 0.00287612937  103-day doubling: 0.00672958427 |
| 192 | Sig_MDSCTeff=Other | 0.2 | dimensionless | Estimated | Fraction of inhibitory effect that other factors expressed by MDSCs have on the Effector T cells in the tumor |
| 193 | Sig_MDSCTeff=CD80 | Calculated | dimensionless | Calculated | Fraction of CD80 receptors expressed on Effector T cells that are interacting with PD-L1 on the MDSCs in the lymph nodes |
| 194 | Sig_MDSCTeff=PD1 | Calculated | dimensionless | Calculated | Fraction of PD-1 receptors expressed on Effector T cells that are interacting with PD-L1 on the MDSCs in the lymph nodes |
| 195 | Sig_MDSCTeff=PDL1 | Calculated | dimensionless | Calculated | Fraction of PD-L1 receptors expressed on Effector T cells that are interacting with PD-1 on the MDSCs in the lymph nodes |
| 196 | Sig_MDSCTeff=Total | Calculated | dimensionless | Calculated | Total inhibitory fraction that MDSCs have on the Effector T cells in the tumor following their interaction |
| 197 | Sig_NT=CD28 | Calculated | dimensionless | Calculated | Positive CD28 co-receptor fraction signaling on Naive T cells by CD80 and CD86 on mAPC in the lymph nodes following their interaction |
| 198 | Sig_PNT=CTLA4/PD1/L1/CD80 | Calculated | dimensionless | Calculated | Total inhibitory fraction that mAPCs have on the Effector T cells during priming following their interaction |
| 199 | Sig_T=Other | 0.2 | dimensionless | Estimated | Fraction of inhibitory effect that other factors expressed by all cancer cells have on the Effector T cells in the tumor |
| 200 | Sig_T1=Total | Calculated | dimensionless | Calculated | Total fraction of inhibitory effect that cancer cells expressing only other factors have on the Effector T cells in the tumor |
| 201 | Sig_T10=Total | Calculated | dimensionless | Calculated | Total fraction of inhibitory effect that cancer cells expressing PD-1, PD-L1, PD-L2 and other factors have on the Effector T cells in the tumor |
| 202 | Sig_T10a=PDL1 | Calculated | dimensionless | Calculated | Fraction of PD-L1 receptors on Effector T cells that are occupied by PD-1 expressed on the T10 subset of cancer cells in the tumor |
| 203 | Sig_T10b=PD1 | Calculated | dimensionless | Calculated | Fraction of PD-1 receptors on Effector T cells that are occupied by PD-L1 and PD-L2 expressed on the T10 subset of cancer cells in the tumor |
| 204 | Sig_T10c=CD80 | Calculated | dimensionless | Calculated | Fraction of CD80 receptors on Effector T cells that are occupied by PD-L1 expressed on the T10 subset of cancer cells in the tumor |
| 205 | Sig_T11=Total | Calculated | dimensionless | Calculated | Total fraction of inhibitory effect that cancer cells expressing PD-L1, CD80 and other factors have on the Effector T cells in the tumor |
| 206 | Sig_T11a=PDL1 | Calculated | dimensionless | Calculated | Fraction of PD-L1 receptors on Effector T cells that are occupied by CD80 expressed on the T11 subset of cancer cells in the tumor |
| 207 | Sig_T11b=PD1 | Calculated | dimensionless | Calculated | Fraction of PD-1 receptors on Effector T cells that are occupied by PD-L1 expressed on the T11 subset of cancer cells in the tumor |
| 208 | Sig_T12=Total | Calculated | dimensionless | Calculated | Total fraction of inhibitory effect that cancer cells expressing PD-L2, CD80 and other factors have on the Effector T cells in the tumor |
| 209 | Sig_T12a=PDL1 | Calculated | dimensionless | Calculated | Fraction of PD-L1 receptors on Effector T cells that are occupied by CD80 expressed on the T12 subset of cancer cells in the tumor |
| 210 | Sig_T12b=PD1 | Calculated | dimensionless | Calculated | Fraction of PD-1 receptors on Effector T cells that are occupied by PD-L2 expressed on the T12 subset of cancer cells in the tumor |
| 211 | Sig_T13=Total | Calculated | dimensionless | Calculated | Total fraction of inhibitory effect that cancer cells expressing PD-L1, PD-L2, CD80 and other factors have on the Effector T cells in the tumor |
| 212 | Sig_T13a=PDL1 | Calculated | dimensionless | Calculated | Fraction of PD-L1 receptors on Effector T cells that are occupied by CD80 expressed on the T13 subset of cancer cells in the tumor |
| 213 | Sig_T13b=PD1 | Calculated | dimensionless | Calculated | Fraction of PD-1 receptors on Effector T cells that are occupied by PD-L1 and PD-L2 expressed on the T13 subset of cancer cells in the tumor |
| 214 | Sig_T14=Total | Calculated | dimensionless | Calculated | Total fraction of inhibitory effect that cancer cells expressing PD-1, PD-L1, CD80 and other factors have on the Effector T cells in the tumor |
| 215 | Sig_T14a=PDL1 | Calculated | dimensionless | Calculated | Fraction of PD-L1 receptors on Effector T cells that are occupied by CD80 and PD-1 expressed on the T14 subset of cancer cells in the tumor |
| 216 | Sig_T14b=PD1 | Calculated | dimensionless | Calculated | Fraction of PD-1 receptors on Effector T cells that are occupied by PD-L1 expressed on the T14 subset of cancer cells in the tumor |
| 217 | Sig_T14c=CD80 | Calculated | dimensionless | Calculated | Fraction of CD80 receptors on Effector T cells that are occupied by PD-L1 expressed on the T14 subset of cancer cells in the tumor |
| 218 | Sig_T15=Total | Calculated | dimensionless | Calculated | Total fraction of inhibitory effect that cancer cells expressing PD-1, PD-L2, CD80 and other factors have on the Effector T cells in the tumor |
| 219 | Sig_T15a=PDL1 | Calculated | dimensionless | Calculated | Fraction of PD-L1 receptors on Effector T cells that are occupied by CD80 and PD-1 expressed on the T15 subset of cancer cells in the tumor |
| 220 | Sig_T15b=PD1 | Calculated | dimensionless | Calculated | Fraction of PD-1 receptors on Effector T cells that are occupied by PD-L2 expressed on the T15 subset of cancer cells in the tumor |
| 221 | Sig_T16=Total | Calculated | dimensionless | Calculated | Total fraction of inhibitory effect that cancer cells expressing PD-1, PD-L1, PD-L2, CD80 and other factors have on the Effector T cells in the tumor |
| 222 | Sig_T16a=PDL1 | Calculated | dimensionless | Calculated | Fraction of PD-L1 receptors on Effector T cells that are occupied by CD80 and PD-1 expressed on the T16 subset of cancer cells in the tumor |
| 223 | Sig_T16b=PD1 | Calculated | dimensionless | Calculated | Fraction of PD-1 receptors on Effector T cells that are occupied by PD-L1 and PD-L2 expressed on the T16 subset of cancer cells in the tumor |
| 224 | Sig_T16c=CD80 | Calculated | dimensionless | Calculated | Fraction of CD80 receptors on Effector T cells that are occupied by PD-L1 expressed on the T16 subset of cancer cells in the tumor |
| 225 | Sig_T2=PDL1 | Calculated | dimensionless | Calculated | Fraction of PD-L1 receptors on Effector T cells that are occupied by PD-1 expressed on the T2 subset of cancer cells in the tumor |
| 226 | Sig_T2=Total | Calculated | dimensionless | Calculated | Total fraction of inhibitory effect that cancer cells expressing PD-1 and other factors have on the Effector T cells in the tumor |
| 227 | Sig_T3=Total | Calculated | dimensionless | Calculated | Total fraction of inhibitory effect that cancer cells expressing PD-L1 and other factors have on the Effector T cells in the tumor |
| 228 | Sig_T3a=PD1 | Calculated | dimensionless | Calculated | Fraction of PD-1 receptors on Effector T cells that are occupied by PD-L1 expressed on the T3 subset of cancer cells in the tumor |
| 229 | Sig_T3b=CD80 | Calculated | dimensionless | Calculated | Fraction of CD80 receptors on Effector T cells that are occupied by PD-L1 expressed on the T3 subset of cancer cells in the tumor |
| 230 | Sig_T4=PD1 | Calculated | dimensionless | Calculated | Fraction of PD-1 receptors on Effector T cells that are occupied by PD-L2 expressed on the T4 subset of cancer cells in the tumor |
| 231 | Sig_T4=Total | Calculated | dimensionless | Calculated | Total fraction of inhibitory effect that cancer cells expressing PD-L2 and other factors have on the Effector T cells in the tumor |
| 232 | Sig_T5=PDL1 | Calculated | dimensionless | Calculated | Fraction of PD-L1 receptors on Effector T cells that are occupied by CD80 expressed on the T5 subset of cancer cells in the tumor |
| 233 | Sig_T5=Total | Calculated | dimensionless | Calculated | Total fraction of inhibitory effect that cancer cells expressing CD80 and other factors have on the Effector T cells in the tumor |
| 234 | Sig_T6=Total | Calculated | dimensionless | Calculated | Total fraction of inhibitory effect that cancer cells expressing PD-1, PD-L1 and other factors have on the Effector T cells in the tumor |
| 235 | Sig_T6a=PDL1 | Calculated | dimensionless | Calculated | Fraction of PD-L1 receptors on Effector T cells that are occupied by PD-1 expressed on the T6 subset of cancer cells in the tumor |
| 236 | Sig_T6b=PD1 | Calculated | dimensionless | Calculated | Fraction of PD-1 receptors on Effector T cells that are occupied by PD-L1 expressed on the T6 subset of cancer cells in the tumor |
| 237 | Sig_T6c=CD80 | Calculated | dimensionless | Calculated | Fraction of CD80 receptors on Effector T cells that are occupied by PD-L1 expressed on the T6 subset of cancer cells in the tumor |
| 238 | Sig_T7=Total | Calculated | dimensionless | Calculated | Total fraction of inhibitory effect that cancer cells expressing PD-1, PD-L2 and other factors have on the Effector T cells in the tumor |
| 239 | Sig_T7a=PDL1 | Calculated | dimensionless | Calculated | Fraction of PD-L1 receptors on Effector T cells that are occupied by PD-1 expressed on the T7 subset of cancer cells in the tumor |
| 240 | Sig_T7b=PD1 | Calculated | dimensionless | Calculated | Fraction of PD-1 receptors on Effector T cells that are occupied by PD-L2 expressed on the T7 subset of cancer cells in the tumor |
| 241 | Sig_T8=Total | Calculated | dimensionless | Calculated | Total fraction of inhibitory effect that cancer cells expressing PD-L1, PD-L2 and other factors have on the Effector T cells in the tumor |
| 242 | Sig_T8a=PD1 | Calculated | dimensionless | Calculated | Fraction of PD-1 receptors on Effector T cells that are occupied by PD-L1 and PD-L2 expressed on the T8 subset of cancer cells in the tumor |
| 243 | Sig_T8b=CD80 | Calculated | dimensionless | Calculated | Fraction of CD80 receptors on Effector T cells that are occupied by PD-L1 expressed on the T8 subset of cancer cells in the tumor |
| 244 | Sig_T9=PDL1 | Calculated | dimensionless | Calculated | Fraction of PD-L1 receptors on Effector T cells that are occupied by PD-1 and CD80 expressed on the T9 subset of cancer cells in the tumor |
| 245 | Sig_T9=Total | Calculated | dimensionless | Calculated | Total fraction of inhibitory effect that cancer cells expressing PD-1, CD80 and other factors have on the Effector T cells in the tumor |
| 246 | Sig_TrACTLA4S=Total | Calculated | dimensionless | Calculated | Fraction of CTLA4 receptors expressed on T Regulatory cells that occupied by Anti-CTLA-4 in the tumor |
| 247 | Sig_TrAPC=Other | 0 | dimensionless | Estimated | Fraction of inhibitory effect that other factors expressed by T Regulatory cells have on the mAPCs in the tumor |
| 248 | Sig_TrAPCT=CTLA4 | Calculated | dimensionless | Calculated | Fraction of CD80 and CD86 receptors on mAPCs that are occupied by CTLA-4 expressed on T Regulatory cells in the tumor |
| 249 | Sig_TrAPCT=Total | Calculated | dimensionless | Calculated | Total inhibitory fraction that T Regulatory cells have on the mAPCs in the tumor following their interaction |
| 250 | Sig_TrPNT=PD1/L1/CD80 | Calculated | dimensionless | Calculated | Total inhibitory fraction that T Regulatory cells have on the Primed Naive T cells in the lymph nodes following their interaction |
| 251 | Sig_TrTeff=Other | 0.2 | dimensionless | Estimated | Fraction of inhibitory effect that other factors expressed by T Regulatory cells have on the Effector T cells in the tumor |
| 252 | Sig_TrTeff=CD80 | Calculated | dimensionless | Calculated | Fraction of CD80 receptors expressed on Effector T cells that are interacting with PD-L1 on the T Regulatory cells in the lymph nodes |
| 253 | Sig_TrTeff=PD1 | Calculated | dimensionless | Calculated | Fraction of PD-1 receptors expressed on Effector T cells that are interacting with PD-L1 on the T Regulatory cells in the lymph nodes |
| 254 | Sig_TrTeff=PDL1 | Calculated | dimensionless | Calculated | Fraction of PD-L1 receptors expressed on Effector T cells that are interacting with PD-1 on the T Regulatory cells in the lymph nodes |
| 255 | Sig_TrTeff=Total | Calculated | dimensionless | Calculated | Total inhibitory fraction that T Regulatory cells have on the Effector T cells in the tumor following their interaction |
| 256 | Sigmax_MDSCTeff=CD80 | 0.048477683 | dimensionless | Calculated | Maximum fraction of CD80 receptors expressed on Effector T cells that are interacting with PD-L1 on the MDSCs in the lymph nodes |
| 257 | Sigmax_MDSCTeff=PD1 | 0.105575948 | dimensionless | Calculated | Maximum fraction of PD-1 receptors expressed on Effector T cells that are interacting with PD-L1 on the MDSCs in the lymph nodes |
| 258 | Sigmax_MDSCTeff=PDL1 | 0.040757628 | dimensionless | Calculated | Maximum fraction of PD-L1 receptors expressed on Effector T cells that are interacting with PD-1 on the MDSCs in the lymph nodes |
| 259 | Sigmax_NT=CD28 | 0.839539945 | dimensionless | Calculated | Maximum fraction of CD28 co-receptor interaction on Naive T cells by CD80 and CD86 on mAPC in the lymph nodes |
| 260 | Sigmax_PNT=CD80 | 0.023415171 | dimensionless | Calculated | Maximum fraction of total CD80 receptors on Primed Naïve T cells that can interact with the PD-L1 receptors on mAPCs during priming in the lymph nodes |
| 261 | Sigmax_PNT=CTLA4 | 0.969874083 | dimensionless | Calculated | Maximum fraction of total CTLA-4 receptors on Primed Naïve T cells that can interact with the CD80 and CD86 receptors on mAPCs during priming in the lymph nodes |
| 262 | Sigmax_PNT=PD1 | 0.046478399 | dimensionless | Calculated | Maximum fraction of total PD-1 receptors on Primed Naïve T cells that can interact with the PD-L1 and PD-L2 receptors on mAPCs during priming in the lymph nodes |
| 263 | Sigmax_PNT=PDL1 | 0.044654093 | dimensionless | Calculated | Maximum fraction of total PD-L1 receptors on Primed Naïve T cells that can interact with the PD-1 and CD80 receptors on mAPCs during priming in the lymph nodes |
| 264 | Sigmax_T10a=PDL1 | 0.04855961 | dimensionless | Calculated | Maximum fraction of PD-L1 receptors on Effector T cells that are occupied by PD-1 expressed on the T10 subset of cancer cells in the tumor |
| 265 | Sigmax_T10b=PD1 | 0.343986457 | dimensionless | Calculated | Maximum fraction of PD-1 receptors on Effector T cells that are occupied by PD-L1 and PD-L2 expressed on the T10 subset of cancer cells in the tumor |
| 266 | Sigmax_T10c=CD80 | 0.15220109 | dimensionless | Calculated | Maximum fraction of CD80 receptors on Effector T cells that are occupied by PD-L1 expressed on the T10 subset of cancer cells in the tumor |
| 267 | Sigmax_T11a=PDL1 | 0.068857858 | dimensionless | Calculated | Maximum fraction of PD-L1 receptors on Effector T cells that are occupied by CD80 expressed on the T11 subset of cancer cells in the tumor |
| 268 | Sigmax_T11b=PD1 | 0.327405152 | dimensionless | Calculated | Maximum fraction of PD-1 receptors on Effector T cells that are occupied by PD-L1 expressed on the T11 subset of cancer cells in the tumor |
| 269 | Sigmax_T12a=PDL1 | 0.068857858 | dimensionless | Calculated | Maximum fraction of PD-L1 receptors on Effector T cells that are occupied by CD80 expressed on the T12 subset of cancer cells in the tumor |
| 270 | Sigmax_T12b=PD1 | 0.091621416 | dimensionless | Calculated | Maximum fraction of PD-1 receptors on Effector T cells that are occupied by PD-L2 expressed on the T12 subset of cancer cells in the tumor |
| 271 | Sigmax_T13a=PDL1 | 0.068857858 | dimensionless | Calculated | Maximum fraction of PD-L1 receptors on Effector T cells that are occupied by CD80 expressed on the T13 subset of cancer cells in the tumor |
| 272 | Sigmax_T13b=PD1 | 0.372279914 | dimensionless | Calculated | Maximum fraction of PD-1 receptors on Effector T cells that are occupied by PD-L1 and PD-L2 expressed on the T13 subset of cancer cells in the tumor |
| 273 | Sigmax_T14a=PDL1 | 0.111571041 | dimensionless | Calculated | Maximum fraction of PD-L1 receptors on Effector T cells that are occupied by CD80 and PD-1 expressed on the T14 subset of cancer cells in the tumor |
| 274 | Sigmax_T14b=PD1 | 0.295136441 | dimensionless | Calculated | Maximum fraction of PD-1 receptors on Effector T cells that are occupied by PD-L1 expressed on the T14 subset of cancer cells in the tumor |
| 275 | Sigmax_T14c=CD80 | 0.152201088 | dimensionless | Calculated | Maximum fraction of CD80 receptors on Effector T cells that are occupied by PD-L1 expressed on the T14 subset of cancer cells in the tumor |
| 276 | Sigmax_T15a=PDL1 | 0.111571041 | dimensionless | Calculated | Maximum fraction of PD-L1 receptors on Effector T cells that are occupied by CD80 and PD-1 expressed on the T15 subset of cancer cells in the tumor |
| 277 | Sigmax_T15b=PD1 | 0.091621416 | dimensionless | Calculated | Maximum fraction of PD-1 receptors on Effector T cells that are occupied by PD-L2 expressed on the T15 subset of cancer cells in the tumor |
| 278 | Sigmax_T16a=PDL1 | 0.111571041 | dimensionless | Calculated | Maximum fraction of PD-L1 receptors on Effector T cells that are occupied by CD80 and PD-1 expressed on the T16 subset of cancer cells in the tumor |
| 279 | Sigmax_T16b=PD1 | 0.343986457 | dimensionless | Calculated | Maximum fraction of PD-1 receptors on Effector T cells that are occupied by PD-L1 and PD-L2 expressed on the T16 subset of cancer cells in the tumor |
| 280 | Sigmax_T16c=CD80 | 0.15220109 | dimensionless | Calculated | Maximum fraction of CD80 receptors on Effector T cells that are occupied by PD-L1 expressed on the T16 subset of cancer cells in the tumor |
| 281 | Sigmax_T2=PDL1 | 0.04855961 | dimensionless | Calculated | Maximum fraction of PD-L1 receptors on Effector T cells that are occupied by PD-1 expressed on the T2 subset of cancer cells in the tumor |
| 282 | Sigmax_T3a=PD1 | 0.295136441 | dimensionless | Calculated | Maximum fraction of PD-1 receptors on Effector T cells that are occupied by PD-L1 expressed on the T3 subset of cancer cells in the tumor |
| 283 | Sigmax_T3b=CD80 | 0.152201076 | dimensionless | Calculated | Maximum fraction of CD80 receptors on Effector T cells that are occupied by PD-L1 expressed on the T3 subset of cancer cells in the tumor |
| 284 | Sigmax_T4=PD1 | 0.091621416 | dimensionless | Calculated | Maximum fraction of PD-1 receptors on Effector T cells that are occupied by PD-L2 expressed on the T4 subset of cancer cells in the tumor |
| 285 | Sigmax_T5=PDL1 | 0.068857858 | dimensionless | Calculated | Maximum fraction of PD-L1 receptors on Effector T cells that are occupied by CD80 expressed on the T5 subset of cancer cells in the tumor |
| 286 | Sigmax_T6a=PDL1 | 0.04855961 | dimensionless | Calculated | Maximum fraction of PD-L1 receptors on Effector T cells that are occupied by PD-1 expressed on the T6 subset of cancer cells in the tumor |
| 287 | Sigmax_T6b=PD1 | 0.295136441 | dimensionless | Calculated | Maximum fraction of PD-1 receptors on Effector T cells that are occupied by PD-L1 expressed on the T6 subset of cancer cells in the tumor |
| 288 | Sigmax_T6c=CD80 | 0.152201087 | dimensionless | Calculated | Maximum fraction of CD80 receptors on Effector T cells that are occupied by PD-L1 expressed on the T6 subset of cancer cells in the tumor |
| 289 | Sigmax_T7a=PDL1 | 0.04855961 | dimensionless | Calculated | Maximum fraction of PD-L1 receptors on Effector T cells that are occupied by PD-1 expressed on the T7 subset of cancer cells in the tumor |
| 290 | Sigmax_T7b=PD1 | 0.091621416 | dimensionless | Calculated | Maximum fraction of PD-1 receptors on Effector T cells that are occupied by PD-L2 expressed on the T7 subset of cancer cells in the tumor |
| 291 | Sigmax_T8a=PD1 | 0.343986457 | dimensionless | Calculated | Maximum fraction of PD-1 receptors on Effector T cells that are occupied by PD-L1 and PD-L2 expressed on the T8 subset of cancer cells in the tumor |
| 292 | Sigmax_T8b=CD80 | 0.152201087 | dimensionless | Calculated | Maximum fraction of CD80 receptors on Effector T cells that are occupied by PD-L1 expressed on the T8 subset of cancer cells in the tumor |
| 293 | Sigmax_T9=PDL1 | 0.111571041 | dimensionless | Calculated | Maximum fraction of PD-L1 receptors on Effector T cells that are occupied by PD-1 and CD80 expressed on the T9 subset of cancer cells in the tumor |
| 294 | Sigmax_TrAPCT=CTLA4 | 0.152864738 | dimensionless | Calculated | Maximum fraction of CD80 and CD86 receptors on mAPCs that are occupied by CTLA-4 expressed on T Regulatory cells in the tumor |
| 295 | Sigmax_TrPNT=CD80 | 0.020305139 | dimensionless | Calculated | Maximum fraction of total CD80 receptors on Primed Naïve T cells that are involved in interacting with the PD-L1 receptors on Regulatory T cells during priming in the lymph nodes |
| 296 | Sigmax_TrPNT=Other | 1 | dimensionless | Calculated | Maximum level of inhibitory effects against Primed Naive T cells by other factors expressed by T Regulatory cells |
| 297 | Sigmax_TrPNT=PD1 | 0.034241129 | dimensionless | Calculated | Maximum fraction of total PD-1 receptors on Primed Naïve T cells that are involved in interacting with the PD-L1 receptors on Regulatory T cells during priming in the lymph nodes |
| 298 | Sigmax_TrPNT=PDL1 | 0.010435276 | dimensionless | Calculated | Maximum fraction of total PD-L1 receptors on Primed Naïve T cells that are involved in interacting with the PD-1 receptors on Regulatory T cells during priming in the lymph nodes |
| 299 | Sigmax_TrTeff=CD80 | 0.048477733 | dimensionless | Calculated | Maximum fraction of CD80 receptors expressed on Effector T cells that are interacting with PD-L1 on the T Regulatory cells in the tumor |
| 300 | Sigmax_TrTeff=PD1 | 0.105576051 | dimensionless | Calculated | Maximum fraction of PD-1 receptors expressed on Effector T cells that are interacting with PD-L1 on the T Regulatory cells in the tumor |
| 301 | Sigmax_TrTeff=PDL1 | 0.040757673 | dimensionless | Calculated | Maximum fraction of PD-L1 receptors expressed on Effector T cells that are interacting with PD-1 on the T Regulatory cells in the tumor |
| 302 | Sigweight_MDSCTeff=CD80 | 0.25 | dimensionless | Calculated | Weight of Effector T cell inhibition by signaling through CD80 receptors expressed on Effector T cells that are interacting with PD-L1 on the MDSCs in the tumor |
| 303 | Sigweight_MDSCTeff=PD1 | 0.541667 | dimensionless | Calculated | Weight of Effector T cell inhibition by signaling through PD-1 receptors expressed on Effector T cells that are interacting with PD-L1 on the MDSCs in the tumor |
| 304 | Sigweight_MDSCTeff=PDL1 | 0.208333 | dimensionless | Calculated | Weight of Effector T cell inhibition by signaling through PD-L1 receptors expressed on Effector T cells that are interacting with PD-1 on the MDSCs in the tumor |
| 305 | SigWeight_PNT=CD80 | 0.01875 | dimensionless | Calculated | Weight of Primed Naive T cell inhibition by signaling through CD80 receptors on Primed Naïve T cells that can interact with the PD-L1 receptors on mAPCs during priming in the lymph nodes |
| 306 | SigWeight_PNT=CTLA4 | 0.80625 | dimensionless | Calculated | Weight of Primed Naive T cell inhibition by signaling through CTLA-4 receptors on Primed Naïve T cells that can interact with the CD80 and CD86 receptors on mAPCs during priming in the lymph nodes |
| 307 | SigWeight_PNT=PD1 | 0.0375 | dimensionless | Calculated | Weight of Primed Naive T cell inhibition by signaling through PD-1 receptors on Primed Naïve T cells that can interact with the PD-L1 and PD-L2 receptors on mAPCs during priming in the lymph nodes |
| 308 | SigWeight_PNT=PDL1 | 0.0375 | dimensionless | Calculated | Weight of Primed Naive T cell inhibition by signaling through PD-L1 receptors on Primed Naïve T cells that can interact with the PD-1 and CD80 receptors on mAPCs during priming in the lymph nodes |
| 309 | Sigweight_T10a=PDL1 | 0.09 | dimensionless | Calculated | Weight of Effector T cell inhibition by signaling through PD-L1 receptors on Effector T cells that are occupied by PD-1 expressed on the T10 subset of cancer cells in the tumor |
| 310 | Sigweight_T10b=PD1 | 0.63 | dimensionless | Calculated | Weight of Effector T cell inhibition by signaling through PD-1 receptors on Effector T cells that are occupied by PD-L1 and PD-L2 expressed on the T10 subset of cancer cells in the tumor |
| 311 | Sigweight_T10c=CD80 | 0.28 | dimensionless | Calculated | Weight of Effector T cell inhibition by signaling through CD80 receptors on Effector T cells that are occupied by PD-L1 expressed on the T10 subset of cancer cells in the tumor |
| 312 | Sigweight_T11a=PDL1 | 0.17 | dimensionless | Calculated | Weight of Effector T cell inhibition by signaling through PD-L1 receptors on Effector T cells that are occupied by CD80 expressed on the T11 subset of cancer cells in the tumor |
| 313 | Sigweight_T11b=PD1 | 0.83 | dimensionless | Calculated | Weight of Effector T cell inhibition by signaling through PD-1 receptors on Effector T cells that are occupied by PD-L1 expressed on the T11 subset of cancer cells in the tumor |
| 314 | Sigweight_T12a=PDL1 | 0.44 | dimensionless | Calculated | Weight of Effector T cell inhibition by signaling through PD-L1 receptors on Effector T cells that are occupied by CD80 expressed on the T12 subset of cancer cells in the tumor |
| 315 | Sigweight_T12b=PD1 | 0.56 | dimensionless | Calculated | Weight of Effector T cell inhibition by signaling through PD-1 receptors on Effector T cells that are occupied by PD-L2 expressed on the T12 subset of cancer cells in the tumor |
| 316 | Sigweight_T13a=PDL1 | 0.16 | dimensionless | Calculated | Weight of Effector T cell inhibition by signaling through PD-L1 receptors on Effector T cells that are occupied by CD80 expressed on the T13 subset of cancer cells in the tumor |
| 317 | Sigweight_T13b=PD1 | 0.84 | dimensionless | Calculated | Weight of Effector T cell inhibition by signaling through PD-1 receptors on Effector T cells that are occupied by PD-L1 and PD-L2 expressed on the T13 subset of cancer cells in the tumor |
| 318 | Sigweight_T14a=PDL1 | 0.2 | dimensionless | Calculated | Weight of Effector T cell inhibition by signaling through PD-L1 receptors on Effector T cells that are occupied by CD80 and PD-1 expressed on the T14 subset of cancer cells in the tumor |
| 319 | Sigweight_T14b=PD1 | 0.53 | dimensionless | Calculated | Weight of Effector T cell inhibition by signaling through PD-1 receptors on Effector T cells that are occupied by PD-L1 expressed on the T14 subset of cancer cells in the tumor |
| 320 | Sigweight_T14c=CD80 | 0.27 | dimensionless | Calculated | Weight of Effector T cell inhibition by signaling through CD80 receptors on Effector T cells that are occupied by PD-L1 expressed on the T14 subset of cancer cells in the tumor |
| 321 | Sigweight_T15a=PDL1 | 0.55 | dimensionless | Calculated | Weight of Effector T cell inhibition by signaling through PD-L1 receptors on Effector T cells that are occupied by CD80 and PD-1 expressed on the T15 subset of cancer cells in the tumor |
| 322 | Sigweight_T15b=PD1 | 0.45 | dimensionless | Calculated | Weight of Effector T cell inhibition by signaling through PD-1 receptors on Effector T cells that are occupied by PD-L2 expressed on the T15 subset of cancer cells in the tumor |
| 323 | Sigweight_T16a=PDL1 | 0.19 | dimensionless | Calculated | Weight of Effector T cell inhibition by signaling through PD-L1 receptors on Effector T cells that are occupied by CD80 and PD-1 expressed on the T16 subset of cancer cells in the tumor |
| 324 | Sigweight_T16b=PD1 | 0.56 | dimensionless | Calculated | Weight of Effector T cell inhibition by signaling through PD-1 receptors on Effector T cells that are occupied by PD-L1 and PD-L2 expressed on the T16 subset of cancer cells in the tumor |
| 325 | Sigweight_T16c=CD80 | 0.25 | dimensionless | Calculated | Weight of Effector T cell inhibition by signaling through CD80 receptors on Effector T cells that are occupied by PD-L1 expressed on the T16 subset of cancer cells in the tumor |
| 326 | Sigweight_T2=PDL1 | 1 | dimensionless | Calculated | Weight of Effector T cell inhibition by signaling through PD-L1 receptors on Effector T cells that are occupied by PD-1 expressed on the T2 subset of cancer cells in the tumor |
| 327 | Sigweight_T3a=PD1 | 0.666666667 | dimensionless | Calculated | Weight of Effector T cell inhibition by signaling through PD-1 receptors on Effector T cells that are occupied by PD-L1 expressed on the T3 subset of cancer cells in the tumor |
| 328 | Sigweight_T3b=CD80 | 0.333333333 | dimensionless | Calculated | Weight of Effector T cell inhibition by signaling through CD80 receptors on Effector T cells that are occupied by PD-L1 expressed on the T3 subset of cancer cells in the tumor |
| 329 | Sigweight_T4=PD1 | 1 | dimensionless | Calculated | Weight of Effector T cell inhibition by signaling through PD-1 receptors on Effector T cells that are occupied by PD-L2 expressed on the T4 subset of cancer cells in the tumor |
| 330 | Sigweight_T5=PDL1 | 1 | dimensionless | Calculated | Weight of Effector T cell inhibition by signaling through PD-L1 receptors on Effector T cells that are occupied by CD80 expressed on the T5 subset of cancer cells in the tumor |
| 331 | Sigweight_T6a=PDL1 | 0.1 | dimensionless | Calculated | Weight of Effector T cell inhibition by signaling through PD-L1 receptors on Effector T cells that are occupied by PD-1 expressed on the T6 subset of cancer cells in the tumor |
| 332 | Sigweight_T6b=PD1 | 0.6 | dimensionless | Calculated | Weight of Effector T cell inhibition by signaling through PD-1 receptors on Effector T cells that are occupied by PD-L1 expressed on the T6 subset of cancer cells in the tumor |
| 333 | Sigweight_T6c=CD80 | 0.3 | dimensionless | Calculated | Weight of Effector T cell inhibition by signaling through CD80 receptors on Effector T cells that are occupied by PD-L1 expressed on the T6 subset of cancer cells in the tumor |
| 334 | Sigweight_T7a=PDL1 | 0.34 | dimensionless | Calculated | Weight of Effector T cell inhibition by signaling through PD-L1 receptors on Effector T cells that are occupied by PD-1 expressed on the T7 subset of cancer cells in the tumor |
| 335 | Sigweight_T7b=PD1 | 0.66 | dimensionless | Calculated | Weight of Effector T cell inhibition by signaling through PD-1 receptors on Effector T cells that are occupied by PD-L2 expressed on the T7 subset of cancer cells in the tumor |
| 336 | Sigweight_T8a=PD1 | 0.69 | dimensionless | Calculated | Weight of Effector T cell inhibition by signaling through PD-1 receptors on Effector T cells that are occupied by PD-L1 and PD-L2 expressed on the T8 subset of cancer cells in the tumor |
| 337 | Sigweight_T8b=CD80 | 0.31 | dimensionless | Calculated | Weight of Effector T cell inhibition by signaling through CD80 receptors on Effector T cells that are occupied by PD-L1 expressed on the T8 subset of cancer cells in the tumor |
| 338 | Sigweight_T9=PDL1 | 1 | dimensionless | Calculated | Weight of Effector T cell inhibition by signaling through PD-L1 receptors on Effector T cells that are occupied by PD-1 and CD80 expressed on the T9 subset of cancer cells in the tumor |
| 339 | SigWeight_TrPNT=CD80 | 0.266666667 | dimensionless | Calculated | Weight of Primed Naive T cell inhibition by signaling through CD80 receptors on Primed Naïve T cells that are involved in interacting with the PD-L1 receptors on Regulatory T cells during priming in the lymph nodes |
| 340 | SigWeight_TrPNT=Other | 0.2 | dimensionless | Calculated | Weight of of inhibitory effects against Primed Naive T cells by other factors expressed by T Regulatory cells |
| 341 | SigWeight_TrPNT=PD1 | 0.266666667 | dimensionless | Calculated | Weight of Primed Naive T cell inhibition by signaling through PD-1 receptors on Primed Naïve T cells that are involved in interacting with the PD-L1 receptors on Regulatory T cells during priming in the lymph nodes |
| 342 | SigWeight_TrPNT=PDL1 | 0.266666667 | dimensionless | Calculated | Weight of Primed Naive T cell inhibition by signaling through PD-L1 receptors on Primed Naïve T cells that are involved in interacting with the PD-1 receptors on Regulatory T cells during priming in the lymph nodes |
| 343 | Sigweight_TrTeff=CD80 | 0.25 | dimensionless | Calculated | Weight of Effector T cell inhibition by signaling through CD80 receptors expressed on Effector T cells that are interacting with PD-L1 on the T Regulatory cells in the tumor |
| 344 | Sigweight_TrTeff=PD1 | 0.541667 | dimensionless | Calculated | Weight of Effector T cell inhibition by signaling through PD-1 receptors expressed on Effector T cells that are interacting with PD-L1 on the T Regulatory cells in the tumor |
| 345 | Sigweight_TrTeff=PDL1 | 0.208333 | dimensionless | Calculated | Weight of Effector T cell inhibition by signaling through PD-L1 receptors expressed on Effector T cells that are interacting with PD-1 on the T Regulatory cells in the tumor |
| 346 | Starting_Cancer_Diam_mm | Assigned | mm | Assigned | The diameter of the tumor at which to start its growth in the simulation |
| 347 | Success_NT-mAPC | 500 | dimensionless | [61, 63, 73, 79, 83, 84] | The number of Naive T:mAPC interactions that would occur prior to a successful priming interaction |
| 348 | Success_PNT-mAPC | 50 | dimensionless | [61, 63, 73, 79, 83, 85]  Estimated | The number of Primed Naive T:mAPC interactions that would occur prior to a successful priming interaction. The difference between the number of successful interactions prior to priming between the first and second stage is estimated by enhanced immunity through chemokines that attract the CD8+ T cells where the APCs and T cells have gathered in a more localized area during priming [85]. |
| 349 | T_Cell_Clonality | 10-300 | cell | [38, 40, 86] | Number of Naïve T cells populations specific for different tumor antigens; in other words; the number of anti-tumor T cell clones in each lymph node. |
| 350 | T_Cell_Diffusion | 10 | µm^2^/min | [63, 73, 79, 87] | The diffusion coefficient of Naive and Primed Naive T cells in the lymph nodes during priming |
| 351 | T_cells_per_mAPC | 75 | cell/cell | [32, 48, 72, 73, 88, 89]  Estimated | The maximum number of Naïve and Primed Naïve T cells that can interact with an mAPC at once (surface area of APCs is ~900µm^2^ as a sphere – from ~2500 µm^3^ [88] per APC – and APC:T contact area is ~50µm^2^[48], but have been shown to range from 1um^2^ to >70um^2^ and even as much as 50% of a cell’s surface area [32]). Note: all T cells were estimated to be the same size, and a surface area analysis was used for this estimation. Furthermore, the available surface area on the mAPCs was multiplied by a factor of 3-4-fold [32] to consider dendritic projections, which would increase the surface area relative to the volume. |
| 352 | T_Density | Calculated | cell/µm^3^ | [73]  Calculated | The density of total T cells in the lymph nodes |
| 353 | T_per_Cancer_Cell_Int | 1 | cell/cell | Estimated | The number of Effector T cells that interact with each cancer cell at once |
| 354 | T_per_Cancer_Cell_max | 10 | cell/cell | [72, 73]  Estimated | The maximum number of Effector T cells that can interact with a cancer at once. Note: all T cells were estimated to be the same size, and a surface area analysis was used for this estimation. |
| 355 | T_per_Tr_cell | 10 | cell/cell | [72, 73, 90]  Estimated | The maximum number of T (Naive, Primed Naive and Effector) cells that can interact with a T regulatory cell at once. Note: all T cells were estimated to be the same size, and a surface area analysis was used for this estimation. |
| 356 | TDLN_Radius | 6203.504909 | µm | [73]  Calculated | The radius of the lymph node region where T cell priming and interactions occur |
| 357 | TheEnd | 1 | dimensionless | Assigned | Factor that is 0 or 1, which stops the immune response when the tumor has died, former, or allows the immune response to be active, latter |
| 358 | Time_Start_Therapy | Calculated | day | Calculated | Recorded time when therapy began |
| 359 | Time_Tumor_Death | Calculated | day | Calculated | Recorded time when tumor was sufficiently killed by the immune response |
| 360 | T-mAPC_Cell_Size | 12 | µm | [73] | The diameter of a T cell undergoing priming |
| 361 | Total_Cell_T | Calculated | cell | Calculated | Total number of cells in the tumor used to calculate the chance of engagement |
| 362 | T-mAPC_Inter_tmax_min | 1440 | min | [73]  Estimated | The maximum time (24 hours) over which interaction during each priming stage are considered. |
| 363 | Total_T_per_TDLN | 5000000 | cell | [21] | The total number of T cells in each lymph node |
| 364 | Total_TC_Sig | Calculated | dimensionless | Calculated | The total immune checkpoint inhibiting effect on the Effector T cells by the tumor |
| 365 | Tr_cells_per_mAPC | 75 | cell/cell | [32, 72, 73, 88, 89]  Estimated | The maximum number of T Regulatory cells that can interact with a mAPC cell at once. Note: all T cells were estimated to be the same size, and an approximate surface area analysis was used for this estimation. Furthermore, the available surface area on the mAPCs was multiplied by a factor of 3-4-fold [32] to consider dendritic projections, which would increase the surface area relative to the volume. |
| 366 | Tr_per_T_cell | 10 | cell/cell | [72, 73, 90]  Estimated | The maximum number of T Regulatory cells that can interact with a T (Naive, Primed Naive and Effector) cell at once. Note: all T cells were estimated to be the same size, and a surface area analysis was used for this estimation. |
| 367 | Treg:mAPC_IntTime | 0.0924 | 1/minute | [90] | Rate constant defining the half-life of interaction between T Regulatory cells or MDSCs and mAPCs |
| 368 | Treg:T_IntTime | 0.1386 | 1/minute | [90] | Rate constant defining the half-life of interaction between T Regulatory cells or MDSCs and T (Naive, Primed Naive and Effector) cells |
| 369 | TregLN_Engage | 0.05775 | 1/(mole*hour) | Estimated | Rate constant defining the half-life of engagement between T Regulatory cells and mAPCs or T (Naive, Primed Naive and Effector) cells |
| 370 | Tregs_per_MDSC | 1 | dimensionless | [27]  Estimated | The ratio of T Regulatory cells per MDSC in the tumor |
| 371 | TregTMDSCEng | 0.2079 | 1/hour | Estimated | Rate constant defining the half-life of engagement between mAPCs or Effector T cells, and T Regulatory cells or MDSCs in the tumor |
| 372 | TrLN-mAPC_Cell_Size | 12 | µm | [73, 90]  Estimated | The diameter of a T Regulatory cell interacting with mAPCs |
| 373 | TrLN-PNT_Cell_Size | 12 | µm | [73, 90]  Estimated | The diameter of a T Regulatory cell interacting with Primed Naive T cells |
| 374 | Tumor_Void_Fraction | 0.522024 | dimensionless | [91] | The fraction of tumor volume that is not occupied by cancer cells |
| 375 | V_T:C_10 | Calculated | liter | Calculated | Volume of receptor interactions between Effector T cells and cancer cells in the T10 subgroup at the immunological synapse |
| 376 | V_T:C_11 | Calculated | liter | Calculated | Volume of receptor interactions between Effector T cells and cancer cells in the T11 subgroup at the immunological synapse |
| 377 | V_T:C_12 | Calculated | liter | Calculated | Volume of receptor interactions between Effector T cells and cancer cells in the T12 subgroup at the immunological synapse |
| 378 | V_T:C_13 | Calculated | liter | Calculated | Volume of receptor interactions between Effector T cells and cancer cells in the T13 subgroup at the immunological synapse |
| 379 | V_T:C_14 | Calculated | liter | Calculated | Volume of receptor interactions between Effector T cells and cancer cells in the T14 subgroup at the immunological synapse |
| 380 | V_T:C_15 | Calculated | liter | Calculated | Volume of receptor interactions between Effector T cells and cancer cells in the T15 subgroup at the immunological synapse |
| 381 | V_T:C_16 | Calculated | liter | Calculated | Volume of receptor interactions between Effector T cells and cancer cells in the T16 subgroup at the immunological synapse |
| 382 | V_T:C_2 | Calculated | liter | Calculated | Volume of receptor interactions between Effector T cells and cancer cells in the T2 subgroup at the immunological synapse |
| 383 | V_T:C_3 | Calculated | liter | Calculated | Volume of receptor interactions between Effector T cells and cancer cells in the T3 subgroup at the immunological synapse |
| 384 | V_T:C_4 | Calculated | liter | Calculated | Volume of receptor interactions between Effector T cells and cancer cells in the T4 subgroup at the immunological synapse |
| 385 | V_T:C_5 | Calculated | liter | Calculated | Volume of receptor interactions between Effector T cells and cancer cells in the T5 subgroup at the immunological synapse |
| 386 | V_T:C_6 | Calculated | liter | Calculated | Volume of receptor interactions between Effector T cells and cancer cells in the T6 subgroup at the immunological synapse |
| 387 | V_T:C_7 | Calculated | liter | Calculated | Volume of receptor interactions between Effector T cells and cancer cells in the T7 subgroup at the immunological synapse |
| 388 | V_T:C_8 | Calculated | liter | Calculated | Volume of receptor interactions between Effector T cells and cancer cells in the T8 subgroup at the immunological synapse |
| 389 | V_T:C_9 | Calculated | liter | Calculated | Volume of receptor interactions between Effector T cells and cancer cells in the T9 subgroup at the immunological synapse |
| 390 | Vv_P | 2.12216206 | liter | [17]  Calculated | Volume of blood through which Effector T cells circulate throughout the body |
| 391 | Vi_P | 7.85 | liter | [17]  Calculated | Volume of interstitial space available to effector T cells in periphery |
| 392 | Vol_Cell-Rec_MDSC-TeffT | Calculated | liter | Calculated | Volume of receptor interactions between Effector T cells and MDSCs at the immunological synapse in the tumor |
| 393 | Vol_Cell-Rec_Tr-APCT | Calculated | liter | Calculated | Volume of receptor interactions between mAPCs and T Regulatory cells at the immunological synapse in the tumor |
| 394 | Vol_Cell-Rec_Tr-mAPC | Calculated | liter | Calculated | Volume of receptor interactions between mAPCs and T Regulatory cells at the immunological synapse in the lymph nodes |
| 395 | Vol_Cell-Rec_Tr-PNT | Calculated | liter | Calculated | Volume of receptor interactions between Primed Naive T cells and T Regulatory cells at the immunological synapse in the lymph nodes |
| 396 | Vol_Cell-Rec_Tr-TeffT | Calculated | liter | Calculated | Volume of receptor interactions between Effector T cells and T Regulatory cells at the immunological synapse in the tumor |
| 397 | Vol_per_TDLN | 1 | ml | [92]  Estimated | Volume of a single human lymph node (represented as a sphere) where the T cells reside and undergo priming for probability calculations |
| 398 | Volume_NT-Receptor_Int | Calculated | liter | Calculated | Volume of receptor interactions between Naive T cells and mAPCs at the immunological synapse in the lymph nodes during priming |
| 399 | Volume_PNT-Receptor_Int | Calculated | liter | Calculated | Volume of receptor interactions between Primed Naive T cells and mAPCs at the immunological synapse in the lymph nodes during priming |
| 400 | Vtdln | 0.001 | liter | [92]  Estimated | Volume of a single human lymph node (represented as a sphere) where the T cells reside and undergo priming |
| 401 | Vv_T | 0.2 | milliliter | [17]  Calculated | Volume of vascular space available to effector T cells in the tumor |

**Table S7 – Model Parameters (End)**

**Table S8 – Model Algebraic Equations (Start)**

| Parameter and Species Assignment Equation | Assignment Type |
| --- | --- |
| [Blood-Lymph].CTLA4_mabB_ugml = ([Blood-Lymph].CTLA4_mabB*Tremelimumab_MW)/(K_B_CTLA4) | repeated |
| [Blood-Lymph].PDL1_mabB_ugml = ([Blood-Lymph].PDL1_mabB*Durvalumab_MW)/(K_B_PDL1) | repeated |
| [Prob_NT-mAPC_Interact] = (1-exp((-4*pi*[T-mAPC_Cell_Size]*T_Cell_Diffusion*mAPC_Total_Calc_Pr*Precursor_Frequen_NT*T_Density*[T-mAPC_Inter_tmax_min]*Antigen_Intensity)/(T_Cell_Clonality*[Success_NT-mAPC]))) | repeated |
| Lymph_Node.mAPC_Total_Calc_Pr = (mAPC-mAPC_Int_P1-mAPC_Int_P2)*ge(mAPC-mAPC_Int_P1-mAPC_Int_P2,0) | repeated |
| T_Density = [Total_T_CD8-CD4]/((4/3)*pi*TDLN_Radius^3) | repeated |
| TDLN_Radius = 10000*([Vol_per_TDLN]*(3/(4*pi)))^(1/3) | repeated |
| Precursor_Frequen_NT = Naive_T/[Total_T_CD8-CD4] | repeated |
| Precursor_Frequen_PNT = Primed_Naive_T/[Total_T_CD8-CD4] | repeated |
| [Success_NT-mAPC] = 500 | repeated |
| [Prob_PNT-mAPC_Interact] = (1-exp((-4*pi*[T-mAPC_Cell_Size]*T_Cell_Diffusion*mAPC_Total_Calc_Pr*Precursor_Frequen_PNT*T_Density*[T-mAPC_Inter_tmax_min]*Antigen_Intensity)/(T_Cell_Clonality*[Success_PNT-mAPC]))) | repeated |
| [Success_PNT-mAPC] = 50 | repeated |
| [CTLA4-to-CD28_Ratio_nInt] = 0.035 | repeated |
| [CTLA4_receptors-nInt-PNT] = [CD28_receptors-per-Tcell]*[CTLA4-to-CD28_Ratio_nInt] | repeated |
| [CTLA4-to-CD28_Ratio_Int] = 0.035*(1+Antigen_Intensity*2) | repeated |
| [CTLA4_receptors-Int-PNT] = [CD28_receptors-per-Tcell]*[CTLA4-to-CD28_Ratio_Int] | repeated |
| Lymph_Node.Naive_T0 = T_Cell_Clonality * [Copies-per-T_Cell_Clone] | repeated |
| Lymph_Node.Naive_T = Lymph_Node.Naive_T0 | initial |
| Lymph_Node.PNT1_Int = Lymph_Node.Primed_Naive_T1+0.001*mole | repeated |
| Lymph_Node.mAPC_Int_P1 = 0.001*mole+gt(Naive_T1,0)*gt(Primed_Naive_T1+Naive_T1,0)*( Naive_T1*[mAPC_per_T_cell] * gt(((Naive_T1 * mAPC)/(Primed_Naive_T1 + Naive_T1+1E-100*mole)) / (Naive_T1 *[mAPC_per_T_cell]+ 1E-100*mole),1) + (Naive_T1/(Primed_Naive_T1 + Naive_T1+1E-100*mole)) *mAPC* le(((Naive_T1 * mAPC)/(Primed_Naive_T1 + Naive_T1+1E-100*mole)) / (Naive_T1 *[mAPC_per_T_cell]+ 1E-100*mole),1)) | repeated |
| Lymph_Node.mAPC_Int_P2 = 0.001*mole+gt(Primed_Naive_T1,0)*gt(Primed_Naive_T1+Naive_T1,0)*(Primed_Naive_T1*[mAPC_per_T_cell] * gt(((Primed_Naive_T1 * mAPC)/(Primed_Naive_T1 + Naive_T1+1E-100*mole)) / (Primed_Naive_T1 *[mAPC_per_T_cell]+ 1E-100*mole),1) + (Primed_Naive_T1/(Primed_Naive_T1 + Naive_T1+1E-100*mole)) *mAPC* le(((Primed_Naive_T1 * mAPC)/(Primed_Naive_T1 + Naive_T1+1E-100*mole)) / (Primed_Naive_T1 *[mAPC_per_T_cell]+ 1E-100*mole),1)) | repeated |
| [CD28_POS-to-Total] = (POS_Sig_PNT_CD80+POS_Sig_PNT_CD86)/(POS_Sig_PNT_CD80+POS_Sig_PNT_CD86+PNT_Int_CD28+1E-100*mole) | repeated |
| Lymph_Node.mAPC_nInt = (mAPC - mAPC_Int_P1 - mAPC_Int_P2) * ge(mAPC - mAPC_Int_P1 - mAPC_Int_P2,0) | repeated |
| [CTLA4_NEG-to-Total] = (NEG_Sig_PNT_CD80+NEG_Sig_PNT_CD86)/(NEG_Sig_PNT_CD80+NEG_Sig_PNT_CD86+PNT_CTLA4+CTLA4_mAb_CTLA4+1E-100*mole) | repeated |
| [Volume_PNT-Receptor_Int] = ((4*pi*(([T-mAPC_Cell_Size]/2)^2)*((mAPC_Int_P2)/mAPC_per_T_cell)*15E-3)*(L/mole))*(1E-15)*(IS_Scaling) | repeated |
| [CD80_PNT_NEG-to-Max] = [PNT_CD80-PDL1]/([PNT_Int_CD80] + [PNT_CD80-PDL1]+1E-100*mole) | repeated |
| [PD1_PNT_NEG-to-Max] = ([PNT_PD1-PDL2]+[PNT_PD1-PDL1])/([PNT_Int_PD1] + [PNT_PD1-PDL2]+[PNT_PD1-PDL1] + [PD1mAb_PNT_PD1]+1E-100*mole) | repeated |
| [PDL1_PNT_NEG-to-Max] = ([PNT_PDL1-CD80]+[PNT_PDL1-PD1])/([PNT_Int_PDL1] +[PNT_PDL1-CD80]+[PNT_PDL1-PD1] + [PDL1mAb-PNT_PDL1]+1E-100*mole) | repeated |
| [Blood-Lymph].PD1_mabB_ugml = ([Blood-Lymph].PD1_mabB*Pembrolizumab_MW)/(K_B_PD1) | repeated |
| Lymph_Node.[Total_T_CD8-CD4] = Total_T_per_TDLN | repeated |
| [Vol_Cell-Rec_Tr-PNT] = ((4*pi*(([TrLN-PNT_Cell_Size]/2)^2)*([TregLN-PNT1]/T_per_Tr_cell)*15E-3))*(1E-15)*(IS_Scaling)*(L/mole) | repeated |
| [Vol_Cell-Rec_Tr-mAPC] = ((4*pi*(([TrLN-mAPC_Cell_Size]/2)^2)*(TregLN_mAPC1/mAPC_per_Tr_cell)*15E-3))*(1E-15)*(IS_Scaling)*(L/mole) | repeated |
| [CTLA4_receptors-Tr] = [CD28_receptors-per-Tcell]*[CTLA4-to-CD28_Ratio_Tr] | repeated |
| [CTLA4Sig_TrLN-mAPC] = ([TrALN_CT_CD80] + [TrALN_CT_CD86])/([TrALN_CT_CD80] + [TrALN_CT_CD86] + [Tr-mAPC_CTLA4] + [TrALN_CT_aCT]+1E-100*mole) | repeated |
| [CD80Sig_Tr-PNT] = [TrPNT_PDL1-CD80]/([TrPNT_PDL1-CD80] + [PNT-Tr_CD80] +1E-100*mole) | repeated |
| [PD1Sig_Tr-PNT] = ([TrPNT_PDL1-PD1])/([TrPNT_PDL1-PD1] + [PNT-Tr_PD1] + [PNTTr_PD1_aPD1] +1E-100*mole) | repeated |
| [PDL1Sig_Tr-PNT] = ([TrPNT_PD1-L1])/([TrPNT_PD1-L1] +[PNT-Tr_PDL1]+[PNTTr_PDL1_aPDL1] + 1E-100*mole) | repeated |
| Lymph_Node.TregLN_mAPC1 = Lymph_Node.TregLN_mAPC + 0.001*mole | repeated |
| Lymph_Node.[TregLN-PNT1] = Lymph_Node.[TregLN-PNT]+0.001*mole | repeated |
| Tumor.Effector_TT_C_Eng = (Tumor.Effector_TT-TC2-Exhausted_TT)*ge(Tumor.Effector_TT-TC2-Exhausted_TT,0) | repeated |
| TC_per_max = (T_per_Cancer_Cell_Int/T_per_Cancer_Cell_max) | repeated |
| CTLA4Sig_TrLN = [TrLN_CT_aCT]/([TrLN_CT_aCT] + [TrLN_CTLA4] +1E-100*mole) | repeated |
| Tumor.Cancer1 = (Cancer-TC1)*ge(Cancer-TC1,0) | repeated |
| [V_T:C_2] = ((4*pi*(([T-mAPC_Cell_Size]/2)^2)*([T{PDL1}-{PD1}C])*(Cancer_per_T_Cell_Int/Cancer_per_T_Cell_max)*15E-3)*(L/mole))*(1E-15)*(IS_Scaling) | repeated |
| [V_T:C_3] = ((4*pi*(([T-mAPC_Cell_Size]/2)^2)*([T{PD1}{CD80}-{PDL1}C])*(Cancer_per_T_Cell_Int/Cancer_per_T_Cell_max)*15E-3)*(L/mole))*(1E-15)*(IS_Scaling) | repeated |
| [V_T:C_4] = ((4*pi*(([T-mAPC_Cell_Size]/2)^2)*([T{PD1}-{PDL2}C])*(Cancer_per_T_Cell_Int/Cancer_per_T_Cell_max)*15E-3)*(L/mole))*(1E-15)*(IS_Scaling) | repeated |
| [V_T:C_5] = ((4*pi*(([T-mAPC_Cell_Size]/2)^2)*([T{PDL1}-{CD80}C])*(Cancer_per_T_Cell_Int/Cancer_per_T_Cell_max)*15E-3)*(L/mole))*(1E-15)*(IS_Scaling) | repeated |
| [V_T:C_6] = ((4*pi*(([T-mAPC_Cell_Size]/2)^2)*([T{PD1}{L1}{80}-{PD1}{L1}C])*(Cancer_per_T_Cell_Int/Cancer_per_T_Cell_max)*15E-3)*(L/mole))*(1E-15)*(IS_Scaling) | repeated |
| [V_T:C_7] = ((4*pi*(([T-mAPC_Cell_Size]/2)^2)*([T{PD1}{L1}-{PD1}{L2}C])*(Cancer_per_T_Cell_Int/Cancer_per_T_Cell_max)*15E-3)*(L/mole))*(1E-15)*(IS_Scaling) | repeated |
| [V_T:C_8] = ((4*pi*(([T-mAPC_Cell_Size]/2)^2)*([T{PD1}{80}-{PDL1}{PDL2}C])*(Cancer_per_T_Cell_Int/Cancer_per_T_Cell_max)*15E-3)*(L/mole))*(1E-15)*(IS_Scaling) | repeated |
| [V_T:C_9] = ((4*pi*(([T-mAPC_Cell_Size]/2)^2)*([T{PDL1}-{CD80}{PD1}C])*(Cancer_per_T_Cell_Int/Cancer_per_T_Cell_max)*15E-3)*(L/mole))*(1E-15)*(IS_Scaling) | repeated |
| [V_T:C_10] = ((4*pi*(([T-mAPC_Cell_Size]/2)^2)*([T{PD1}{L1}{80}-{PD1}{L1}{L2}C])*(Cancer_per_T_Cell_Int/Cancer_per_T_Cell_max)*15E-3)*(L/mole))*(1E-15)*(IS_Scaling) | repeated |
| [V_T:C_11] = ((4*pi*(([T-mAPC_Cell_Size]/2)^2)*([T{PD1}{L1}-{PDL1}{80}C])*(Cancer_per_T_Cell_Int/Cancer_per_T_Cell_max)*15E-3)*(L/mole))*(1E-15)*(IS_Scaling) | repeated |
| [V_T:C_12] = ((4*pi*(([T-mAPC_Cell_Size]/2)^2)*([T{PD1}{L1}-{PDL2}{80}C])*(Cancer_per_T_Cell_Int/Cancer_per_T_Cell_max)*15E-3)*(L/mole))*(1E-15)*(IS_Scaling) | repeated |
| [V_T:C_13] = ((4*pi*(([T-mAPC_Cell_Size]/2)^2)*([T{PD1}{L1}-{PDL1}{80}{L2}C])*(Cancer_per_T_Cell_Int/Cancer_per_T_Cell_max)*15E-3)*(L/mole))*(1E-15)*(IS_Scaling) | repeated |
| [V_T:C_14] = ((4*pi*(([T-mAPC_Cell_Size]/2)^2)*([T{PD1}{L1}{80}-{PD1}{80}{L1}C])*(Cancer_per_T_Cell_Int/Cancer_per_T_Cell_max)*15E-3)*(L/mole))*(1E-15)*(IS_Scaling) | repeated |
| [V_T:C_15] = ((4*pi*(([T-mAPC_Cell_Size]/2)^2)*([T{PD1}{L1}-{PD1}{80}{PDL2}C])*(Cancer_per_T_Cell_Int/Cancer_per_T_Cell_max)*15E-3)*(L/mole))*(1E-15)*(IS_Scaling) | repeated |
| [V_T:C_16] = ((4*pi*(([T-mAPC_Cell_Size]/2)^2)*([T{PD1}{L1}{80}-{PD1}{80}{L1}{L2}C])*(Cancer_per_T_Cell_Int/Cancer_per_T_Cell_max)*15E-3)*(L/mole))*(1E-15)*(IS_Scaling) | repeated |
| Tumor.[T{PDL1}-{PD1}C] = 0.001*mole+TC2*[%{PD1}C] | repeated |
| Tumor.[T{PD1}{CD80}-{PDL1}C] = 0.001*mole+TC2*[%{PDL1}C] | repeated |
| Tumor.[T{PD1}-{PDL2}C] = 0.001*mole+TC2*[%{PDL2}C] | repeated |
| Tumor.[T{PDL1}-{CD80}C] = 0.001*mole+TC2*[%{CD80}C] | repeated |
| Tumor.[T{PD1}{L1}{80}-{PD1}{L1}C] = 0.001*mole+TC2*[%{PD1}{L1}C] | repeated |
| Tumor.[T{PD1}{L1}-{PD1}{L2}C] = 0.001*mole+TC2*[%{PD1}{L2}C] | repeated |
| Tumor.[T{PD1}{80}-{PDL1}{PDL2}C] = 0.001*mole+TC2*[%{PDL1}{PDL2}C] | repeated |
| Tumor.[T{PDL1}-{CD80}{PD1}C] = 0.001*mole+TC2*[%{CD80}{PD1}C] | repeated |
| Tumor.[T{PD1}{L1}{80}-{PD1}{L1}{L2}C] = 0.001*mole+TC2*[%{PD1}{L1}{L2}C] | repeated |
| Tumor.[T{PD1}{L1}-{PDL1}{80}C] = 0.001*mole+TC2*[%{PDL1}{80}C] | repeated |
| Tumor.[T{PD1}{L1}-{PDL2}{80}C] = 0.001*mole+TC2*[%{PDL2}{80}C] | repeated |
| Tumor.[T{PD1}{L1}-{PDL1}{80}{L2}C] = 0.001*mole+TC2*[%{PDL1}{80}{L2}C] | repeated |
| Tumor.[T{PD1}{L1}{80}-{PD1}{80}{L1}C] = 0.001*mole+TC2*[%{PD1}{80}{L1}C] | repeated |
| Tumor.[T{PD1}{L1}-{PD1}{80}{PDL2}C] = 0.001*mole+TC2*[%{PD1}{80}{PDL2}C] | repeated |
| Tumor.[T{PD1}{L1}{80}-{PD1}{80}{L1}{L2}C] = 0.001*mole+TC2*[%{PD1}{80}{L1}{L2}C] | repeated |
| Tumor.[C{PD1}] = 0.001*mole+TC1*[%{PD1}C] | repeated |
| Tumor.[C{PDL1}] = 0.001*mole+TC1*[%{PDL1}C] | repeated |
| Tumor.[C{PDL2}] = 0.001*mole+TC1*[%{PDL2}C] | repeated |
| Tumor.[C{CD80}] = 0.001*mole+TC1*[%{CD80}C] | repeated |
| Tumor.[C{PD1}{PDL1}] = 0.001*mole+TC1*[%{PD1}{L1}C] | repeated |
| Tumor.[C{PD1}{PDL2}] = 0.001*mole+TC1*[%{PD1}{L2}C] | repeated |
| Tumor.[C{PDL1}{PDL2}] = 0.001*mole+TC1*[%{PDL1}{PDL2}C] | repeated |
| Tumor.[C{PD1}{CD80}] = 0.001*mole+TC1*[%{CD80}{PD1}C] | repeated |
| Tumor.[C{PD1}{PDL1}{PDL2}] = 0.001*mole+TC1*[%{PD1}{L1}{L2}C] | repeated |
| Tumor.[C{PDL1}{CD80}] = 0.001*mole+TC1*[%{PDL1}{80}C] | repeated |
| Tumor.[C{PDL2}{CD80}] = 0.001*mole+TC1*[%{PDL2}{80}C] | repeated |
| Tumor.[C{PDL1}{PDL2}{CD80}] = 0.001*mole+TC1*[%{PDL1}{80}{L2}C] | repeated |
| Tumor.[C{PD1}{PDL1}{CD80}] = 0.001*mole+TC1*[%{PD1}{80}{L1}C] | repeated |
| Tumor.[C{PD1}{PDL2}{CD80}] = 0.001*mole+TC1*[%{PD1}{80}{PDL2}C] | repeated |
| Tumor.[C{PD1}{PDL1}{PDL2}{CD80}] = 0.001*mole+TC1*[%{PD1}{80}{L1}{L2}C] | repeated |
| [%{Other}C] = (1-Frac_PDL1_Exp_Cancer)*(1-Frac_CD80_Exp_Cancer)*(1-Frac_PDL2_Exp_Cancer)*(1-Frac_PD1_Exp_Cancer) | repeated |
| [%{PD1}C] = Frac_PD1_Exp_Cancer*(1-Frac_PDL1_Exp_Cancer)*(1-Frac_PDL2_Exp_Cancer)*(1-Frac_CD80_Exp_Cancer) | repeated |
| [%{PDL1}C] = Frac_PDL1_Exp_Cancer*(1-Frac_PD1_Exp_Cancer)*(1-Frac_PDL2_Exp_Cancer)*(1-Frac_CD80_Exp_Cancer) | repeated |
| [%{PDL2}C] = Frac_PDL2_Exp_Cancer*(1-Frac_PDL1_Exp_Cancer)*(1-Frac_PD1_Exp_Cancer)*(1-Frac_CD80_Exp_Cancer) | repeated |
| [%{CD80}C] = Frac_CD80_Exp_Cancer*(1-Frac_PDL1_Exp_Cancer)*(1-Frac_PDL2_Exp_Cancer)*(1-Frac_PD1_Exp_Cancer) | repeated |
| [%{PD1}{L1}C] = Frac_PD1_Exp_Cancer*Frac_PDL1_Exp_Cancer*(1-Frac_PDL2_Exp_Cancer)*(1-Frac_CD80_Exp_Cancer) | repeated |
| [%{PD1}{L2}C] = Frac_PD1_Exp_Cancer*Frac_PDL2_Exp_Cancer*(1-Frac_PDL1_Exp_Cancer)*(1-Frac_CD80_Exp_Cancer) | repeated |
| [%{PDL1}{PDL2}C] = Frac_PDL1_Exp_Cancer*Frac_PDL2_Exp_Cancer*(1-Frac_PD1_Exp_Cancer)*(1-Frac_CD80_Exp_Cancer) | repeated |
| [%{CD80}{PD1}C] = Frac_PD1_Exp_Cancer*Frac_CD80_Exp_Cancer*(1-Frac_PDL2_Exp_Cancer)*(1-Frac_PDL1_Exp_Cancer) | repeated |
| [%{PD1}{L1}{L2}C] = Frac_PD1_Exp_Cancer*Frac_PDL1_Exp_Cancer*Frac_PDL2_Exp_Cancer*(1-Frac_CD80_Exp_Cancer) | repeated |
| [%{PDL1}{80}C] = Frac_CD80_Exp_Cancer*Frac_PDL1_Exp_Cancer*(1-Frac_PDL2_Exp_Cancer)*(1-Frac_PD1_Exp_Cancer) | repeated |
| [%{PDL2}{80}C] = Frac_CD80_Exp_Cancer*Frac_PDL2_Exp_Cancer*(1-Frac_PDL1_Exp_Cancer)*(1-Frac_PD1_Exp_Cancer) | repeated |
| [%{PDL1}{80}{L2}C] = Frac_PDL1_Exp_Cancer*Frac_CD80_Exp_Cancer*Frac_PDL2_Exp_Cancer*(1-Frac_PD1_Exp_Cancer) | repeated |
| [%{PD1}{80}{L1}C] = Frac_PD1_Exp_Cancer*Frac_CD80_Exp_Cancer*Frac_PDL1_Exp_Cancer*(1-Frac_PDL2_Exp_Cancer) | repeated |
| [%{PD1}{80}{PDL2}C] = Frac_PD1_Exp_Cancer*Frac_CD80_Exp_Cancer*Frac_PDL2_Exp_Cancer*(1-Frac_PDL1_Exp_Cancer) | repeated |
| [%{PD1}{80}{L1}{L2}C] = Frac_PDL1_Exp_Cancer*Frac_CD80_Exp_Cancer*Frac_PDL2_Exp_Cancer*Frac_PD1_Exp_Cancer | repeated |
| [Sig_T2=PDL1] = ([T2=PDL1:PD1=C2])/([T2=PDL1:PD1=C2] +[T2=PDL1]+[T2=PDL1:aPDL1] +1E-100*mole) | repeated |
| [Sig_T3a=PD1] = ([T3a=PD1:PDL1=C3])/([T3a=PD1:PDL1=C3] +[T3a=PD1]+[T3a=PD1:aPD1] +1E-100*mole) | repeated |
| [Sig_T3b=CD80] = ([T3b=CD80:PDL1=C3])/([T3b=CD80:PDL1=C3] +[T3b=CD80] +1E-100*mole) | repeated |
| [Sig_T4=PD1] = ([T4=PD1:PDL2=C4])/([T4=PD1:PDL2=C4] +[T4=PD1]+[T4=PD1:aPD1] +1E-100*mole) | repeated |
| [Sig_T5=PDL1] = ([T5=PDL1:CD80=C5])/([T5=PDL1:CD80=C5] +[T5=PDL1]+[T5=PDL1:aPDL1] +1E-100*mole) | repeated |
| [Sig_T6a=PDL1] = ([T6a=PDL1:PD1=C6a])/([T6a=PDL1:PD1=C6a] +[T6a=PDL1]+[T6a=PDL1:aPDL1] +1E-100*mole) | repeated |
| [Sig_T6b=PD1] = ([T6b=PD1:PDL1=C6b])/([T6b=PD1:PDL1=C6b] +[T6b=PD1]+[T6b=PD1:aPD1] +1E-100*mole) | repeated |
| [Sig_T6c=CD80] = ([T6c=CD80:PDL1=C6b])/([T6c=CD80:PDL1=C6b] +[T6c=CD80] +1E-100*mole) | repeated |
| [Sig_T7a=PDL1] = ([T7a=PDL1:PD1=C7a])/([T7a=PDL1:PD1=C7a] +[T7a=PDL1]+[T7a=PDL1:aPDL1] +1E-100*mole) | repeated |
| [Sig_T7b=PD1] = ([T7b=PD1:PDL2=C7b])/([T7b=PD1:PDL2=C7b] +[T7b=PD1]+[T7b=PD1:aPD1] +1E-100*mole) | repeated |
| [Sig_T8a=PD1] = ([T8a=PD1:PDL1=C8a] + [T8a=PD1:PDL2=C8b])/([T8a=PD1:PDL1=C8a] +[T8a=PD1:PDL2=C8b] + [T8a=PD1]+[T8a=PD1:aPD1] +1E-100*mole) | repeated |
| [Sig_T8b=CD80] = ([T8b=CD80:PDL1=C8a])/([T8b=CD80:PDL1=C8a] +[T8b=CD80] +1E-100*mole) | repeated |
| [Sig_T9=PDL1] = ([T9=PDL1-PD1=C9a] + [T9=PDL1-CD80=C9b])/([T9=PDL1-PD1=C9a] + [T9=PDL1-CD80=C9b] +[T9=PDL1]+[T9=PDL1:aPDL1] +1E-100*mole) | repeated |
| [Sig_T10a=PDL1] = ([T10a=PDL1:PD1=C10a])/([T10a=PDL1:PD1=C10a] +[T10a=PDL1]+[T10a=PDL1:aPDL1] +1E-100*mole) | repeated |
| [Sig_T10b=PD1] = ([T10b=PD1:PDL1=C10b] + [T10b=PD1:PDL2=C10c])/([T10b=PD1:PDL1=C10b] + [T10b=PD1:PDL2=C10c] +[T10b=PD1]+[T10b=PD1:aPD1] +1E-100*mole) | repeated |
| [Sig_T10c=CD80] = ([T10c=CD80:PDL1=C10b])/([T10c=CD80:PDL1=C10b] +[T10c=CD80] +1E-100*mole) | repeated |
| [Sig_T11a=PDL1] = ([T11a=PDL1:CD80=C11b])/([T11a=PDL1:CD80=C11b] +[T11a=PDL1]+[T11a=PDL1:aPDL1] +1E-100*mole) | repeated |
| [Sig_T11b=PD1] = ([T11b=PD1:PDL1=C11a])/([T11b=PD1:PDL1=C11a] +[T11b=PD1]+[T11b=PD1:aPD1] +1E-100*mole) | repeated |
| [Sig_T12a=PDL1] = ([T12a=PDL1:CD80=C12b])/([T12a=PDL1:CD80=C12b] +[T12a=PDL1]+[T12a=PDL1:aPDL1] +1E-100*mole) | repeated |
| [Sig_T12b=PD1] = ([T12b=PD1:PDL2=C12a])/([T12b=PD1:PDL2=C12a] +[T12b=PD1]+[T12b=PD1:aPD1] +1E-100*mole) | repeated |
| [Sig_T13a=PDL1] = ([T13a=PDL1:CD80=C13c])/([T13a=PDL1:CD80=C13c] +[T13a=PDL1]+[T13a=PDL1:aPDL1] +1E-100*mole) | repeated |
| [Sig_T13b=PD1] = ([T13b=PD1:PDL2=C13b] + [T13b=PD1:PDL1=C13a])/([T13b=PD1:PDL2=C13b] + [T13b=PD1:PDL1=C13a] +[T13b=PD1]+[T13b=PD1:aPD1] +1E-100*mole) | repeated |
| [Sig_T14a=PDL1] = ([T14a=PDL1:PD1=C14a] + [T14a=PDL1:CD80=C14c])/([T14a=PDL1:PD1=C14a] + [T14a=PDL1:CD80=C14c] +[T14a=PDL1]+[T14a=PDL1:aPDL1] +1E-100*mole) | repeated |
| [Sig_T14b=PD1] = ([T14b=PD1:PDL1=C14b])/([T14b=PD1:PDL1=C14b] +[T14b=PD1]+[T14b=PD1:aPD1] +1E-100*mole) | repeated |
| [Sig_T14c=CD80] = ([T14c=CD80:PDL1=C14b])/([T14c=CD80:PDL1=C14b] +[T14c=CD80] +1E-100*mole) | repeated |
| [Sig_T15a=PDL1] = ([T15a=PDL1:CD80=C15c] + [T15a=PDL1:PD1=C15a])/([T15a=PDL1:CD80=C15c]+[T15a=PDL1:PD1=C15a] +[T15a=PDL1]+[T15a=PDL1:aPDL1] +1E-100*mole) | repeated |
| [Sig_T15b=PD1] = ([T15b=PD1:PDL2=C15b])/([T15b=PD1:PDL2=C15b] +[T15b=PD1]+[T15b=PD1:aPD1] +1E-100*mole) | repeated |
| [Sig_T16a=PDL1] = ([T16a=PDL1:PD1=C16a] + [T16a=PDL1:CD80=C16d])/([T16a=PDL1:PD1=C16a]+[T16a=PDL1:CD80=C16d] +[T16a=PDL1]+[T16a=PDL1:aPDL1] +1E-100*mole) | repeated |
| [Sig_T16b=PD1] = ([T16b=PD1:PDL2=C16c]+[T16b=PD1:PDL1=C16b])/([T16b=PD1:PDL2=C16c]+[T16b=PD1:PDL1=C16b] +[T16b=PD1]+[T16b=PD1:aPD1] +1E-100*mole) | repeated |
| [Sig_T16c=CD80] = ([T16c=CD80:PDL1=C16b])/([T16c=CD80:PDL1=C16b] +[T16c=CD80]+1E-100*mole) | repeated |
| Total_TC_Sig = [Sig_T1=Total]*[%{Other}C] + [Sig_T2=Total]*[%{PD1}C] + [Sig_T3=Total]*[%{PDL1}C] + [Sig_T4=Total]*[%{PDL2}C] + [Sig_T5=Total]*[%{CD80}C] + [Sig_T6=Total]*[%{PD1}{L1}C] + [Sig_T7=Total]*[%{PD1}{L2}C] + [Sig_T8=Total]*[%{PDL1}{PDL2}C] + [Sig_T9=Total]*[%{CD80}{PD1}C] + [Sig_T10=Total]*[%{PD1}{L1}{L2}C] + [Sig_T11=Total]*[%{PDL1}{80}C] + [Sig_T12=Total]*[%{PDL2}{80}C] + [Sig_T13=Total]*[%{PDL1}{80}{L2}C] + [Sig_T14=Total]*[%{PD1}{80}{L1}C] + [Sig_T15=Total]*[%{PD1}{80}{PDL2}C] + [Sig_T16=Total]*[%{PD1}{80}{L1}{L2}C] | repeated |
| [Sig_T2=Total] = ([Sigweight_T2=PDL1]*([Sig_T2=PDL1]/[Sigmax_T2=PDL1]))*(1-[Sig_T=Other])+[Sig_T=Other] | repeated |
| [Sig_T3=Total] = ([Sigweight_T3a=PD1]*([Sig_T3a=PD1]/[Sigmax_T3a=PD1]) + [Sigweight_T3b=CD80]*([Sig_T3b=CD80]/[Sigmax_T3b=CD80]))*(1-[Sig_T=Other])+[Sig_T=Other] | repeated |
| [Sig_T4=Total] = ([Sigweight_T4=PD1]*([Sig_T4=PD1]/[Sigmax_T4=PD1]))*(1-[Sig_T=Other])+[Sig_T=Other] | repeated |
| [Sig_T5=Total] = ([Sigweight_T5=PDL1]*([Sig_T5=PDL1]/[Sigmax_T5=PDL1]))*(1-[Sig_T=Other])+[Sig_T=Other] | repeated |
| [Sig_T6=Total] = ([Sigweight_T6a=PDL1]*([Sig_T6a=PDL1]/[Sigmax_T6a=PDL1]) + [Sigweight_T6b=PD1]*([Sig_T6b=PD1]/[Sigmax_T6b=PD1]) + [Sigweight_T6c=CD80]*([Sig_T6c=CD80]/[Sigmax_T6c=CD80]))*(1-[Sig_T=Other])+[Sig_T=Other] | repeated |
| [Sig_T7=Total] = ([Sigweight_T7a=PDL1]*([Sig_T7a=PDL1]/[Sigmax_T7a=PDL1]) + [Sigweight_T7b=PD1]*([Sig_T7b=PD1]/[Sigmax_T7b=PD1]))*(1-[Sig_T=Other])+[Sig_T=Other] | repeated |
| [Sig_T8=Total] = ([Sigweight_T8a=PD1]*([Sig_T8a=PD1]/[Sigmax_T8a=PD1]) + [Sigweight_T8b=CD80]*([Sig_T8b=CD80]/[Sigmax_T8b=CD80]))*(1-[Sig_T=Other])+[Sig_T=Other] | repeated |
| [Sig_T9=Total] = ([Sigweight_T9=PDL1]*([Sig_T9=PDL1]/[Sigmax_T9=PDL1]))*(1-[Sig_T=Other])+[Sig_T=Other] | repeated |
| [Sig_T10=Total] = ([Sigweight_T10a=PDL1]*([Sig_T10a=PDL1]/[Sigmax_T10a=PDL1]) + [Sigweight_T10b=PD1]*([Sig_T10b=PD1]/[Sigmax_T10b=PD1]) + [Sigweight_T10c=CD80]*([Sig_T10c=CD80]/[Sigmax_T10c=CD80]))*(1-[Sig_T=Other])+[Sig_T=Other] | repeated |
| [Sig_T11=Total] = ([Sigweight_T11a=PDL1]*([Sig_T11a=PDL1]/[Sigmax_T11a=PDL1]) + [Sigweight_T11b=PD1]*([Sig_T11b=PD1]/[Sigmax_T11b=PD1]))*(1-[Sig_T=Other])+[Sig_T=Other] | repeated |
| [Sig_T12=Total] = ([Sigweight_T12a=PDL1]*([Sig_T12a=PDL1]/[Sigmax_T12a=PDL1]) + [Sigweight_T12b=PD1]*([Sig_T12b=PD1]/[Sigmax_T12b=PD1]))*(1-[Sig_T=Other])+[Sig_T=Other] | repeated |
| [Sig_T13=Total] = ([Sigweight_T13a=PDL1]*([Sig_T13a=PDL1]/[Sigmax_T13a=PDL1]) + [Sigweight_T13b=PD1]*([Sig_T13b=PD1]/[Sigmax_T13b=PD1]))*(1-[Sig_T=Other])+[Sig_T=Other] | repeated |
| [Sig_T14=Total] = ([Sigweight_T14a=PDL1]*([Sig_T14a=PDL1]/[Sigmax_T14a=PDL1]) + [Sigweight_T14b=PD1]*([Sig_T14b=PD1]/[Sigmax_T14b=PD1]) + [Sigweight_T14c=CD80]*([Sig_T14c=CD80]/[Sigmax_T14c=CD80]))*(1-[Sig_T=Other])+[Sig_T=Other] | repeated |
| [Sig_T15=Total] = ([Sigweight_T15a=PDL1]*([Sig_T15a=PDL1]/[Sigmax_T15a=PDL1]) + [Sigweight_T15b=PD1]*([Sig_T15b=PD1]/[Sigmax_T15b=PD1]))*(1-[Sig_T=Other])+[Sig_T=Other] | repeated |
| [Sig_T16=Total] = ([Sigweight_T16a=PDL1]*([Sig_T16a=PDL1]/[Sigmax_T16a=PDL1]) + [Sigweight_T16b=PD1]*([Sig_T16b=PD1]/[Sigmax_T16b=PD1]) + [Sigweight_T16c=CD80]*([Sig_T16c=CD80]/[Sigmax_T16c=CD80]))*(1-[Sig_T=Other])+[Sig_T=Other] | repeated |
| [Sig_T1=Total] = 0.95 | repeated |
| Frac_CD80_Exp_Cancer = [%CD80_Exp_Cancer]/100 | repeated |
| Frac_PD1_Exp_Cancer = [%PD1_Exp_Cancer]/100 | repeated |
| Frac_PDL1_Exp_Cancer = [%PDL1_Exp_Cancer]/100 | repeated |
| Frac_PDL2_Exp_Cancer = [%PDL2_Exp_Cancer]/100 | repeated |
| Tumor.TC2 = TC1*T_per_Cancer_Cell_Int | repeated |
| Tumor.MDSCsT_EngTeff = MDSCsT_Teff+0.001*mole | repeated |
| Tumor.TregT_EngAPC = TregT_mAPCT+0.001*mole | repeated |
| Tumor.mAPCT_EngTregT = TregT_mAPCT+0.001*mole | repeated |
| Tumor.Teff_EngTregT = TregT_Teff+0.001*mole | repeated |
| Tumor.TregT_EngTeff = TregT_Teff+0.001*mole | repeated |
| Tumor.Teff_EngMDSC = MDSCsT_Teff+0.001*mole | repeated |
| [Vol_Cell-Rec_Tr-APCT] = ((4*pi*(([TrLN-mAPC_Cell_Size]/2)^2)*(TregT_EngAPC/mAPC_per_Tr_cell)*15E-3))*(1E-15)*(IS_Scaling)*(L/mole) | repeated |
| [Vol_Cell-Rec_MDSC-TeffT] = ((4*pi*(([TrLN-PNT_Cell_Size]/2)^2)*(MDSCsT_EngTeff/T_per_Tr_cell)*15E-3))*(1E-15)*(IS_Scaling)*(L/mole) | repeated |
| [Vol_Cell-Rec_Tr-TeffT] = ((4*pi*(([TrLN-PNT_Cell_Size]/2)^2)*(TregT_EngTeff/T_per_Tr_cell)*15E-3))*(1E-15)*(IS_Scaling)*(L/mole) | repeated |
| [Sig_TrTeff=Total] = ([Sigweight_TrTeff=CD80]*([Sig_TrTeff=CD80]/[Sigmax_TrTeff=CD80])+[Sigweight_TrTeff=PD1]*([Sig_TrTeff=PD1]/[Sigmax_TrTeff=PD1])+[Sigweight_TrTeff=PDL1]*([Sig_TrTeff=PDL1]/[Sigmax_TrTeff=PDL1]))*(1-[Sig_TrTeff=Other])+[Sig_TrTeff=Other] | repeated |
| [Sig_TrTeff=CD80] = ([PDL1:CD80_TrTeff])/([PDL1:CD80_TrTeff] + [CD80_TeffT] + 1E-100*mole) | repeated |
| [Sig_TrTeff=PD1] = ([PDL1:PD1_TrTeff])/([PDL1:PD1_TrTeff] + [PD1_TeffT] + [PD1:aPD1_Teff] + 1E-100*mole) | repeated |
| [Sig_TrTeff=PDL1] = ([PD1:PDL1_TrTeff])/([PD1:PDL1_TrTeff] + [PDL1_TeffT] + [PDL1:aPDL1_Teff] + 1E-100*mole) | repeated |
| [Sig_MDSCTeff=CD80] = ([PDL1:CD80_MDSCT])/([PDL1:CD80_MDSCT] +[CD80_TeffT1] + 1E-100*mole) | repeated |
| [Sig_MDSCTeff=PD1] = ([PDL1:PD1_MDSCT])/([PDL1:PD1_MDSCT] + [PD1_TeffT1] + [PD1:aPD1_Teff1] + 1E-100*mole) | repeated |
| [Sig_MDSCTeff=PDL1] = ([PD1:PDL1_MDSCT])/([PD1:PDL1_MDSCT] + [PDL1_TeffT1] + [PDL1:aPDL1_Teff1] + 1E-100*mole) | repeated |
| [Sig_TrAPCT=Total] = ([Sig_TrAPCT=CTLA4]/[Sigmax_TrAPCT=CTLA4])*(1-[Sig_TrAPC=Other])+[Sig_TrAPC=Other] | repeated |
| [Sig_TrAPCT=CTLA4] = ([CTLA4:CD80_TrAT] + [CTLA4:CD86_TrAT])/ ([CTLA4:CD80_TrAT] + [CTLA4:CD86_TrAT] + [CD80_mAPCT] + [CD86_mAPCT] + [CTLA4_CTLA4-Trt] + 1E-100*mole) | repeated |
| [Sig_MDSCTeff=Total] = ([Sigweight_MDSCTeff=CD80]*([Sig_MDSCTeff=CD80]/[Sigmax_MDSCTeff=CD80])+[Sigweight_MDSCTeff=PD1]*([Sig_MDSCTeff=PD1]/[Sigmax_MDSCTeff=PD1])+[Sigweight_MDSCTeff=PDL1]*([Sig_MDSCTeff=PDL1]/[Sigmax_MDSCTeff=PDL1]))*(1-[Sig_MDSCTeff=Other])+[Sig_MDSCTeff=Other] | repeated |
| Tumor.TregT1 = TregT + 0.001*mole | repeated |
| [Sig_TrACTLA4S=Total] = ([CTLA4_aCTLA4-TrTS])/( [CTLA4_aCTLA4-TrTS] +[CTLA4_TregTS] + 1E-100*mole) | repeated |
| [Sig_PNT=CTLA4/PD1/L1/CD80] = ([SigWeight_PNT=CTLA4]*([CTLA4_NEG-to-Total]/[Sigmax_PNT=CTLA4])+[SigWeight_PNT=PDL1]*([PDL1_PNT_NEG-to-Max]/[Sigmax_PNT=PDL1])+[SigWeight_PNT=PD1]*([PD1_PNT_NEG-to-Max]/[Sigmax_PNT=PD1])+[SigWeight_PNT=CD80]*([CD80_PNT_NEG-to-Max]/[Sigmax_PNT=CD80])) | repeated |
| [Sig_TrPNT=PD1/L1/CD80] = ([SigWeight_TrPNT=CD80]*([CD80Sig_Tr-PNT]/[Sigmax_TrPNT=CD80]) + [SigWeight_TrPNT=PD1]*([PD1Sig_Tr-PNT]/[Sigmax_TrPNT=PD1]) + [SigWeight_TrPNT=PDL1]*([PDL1Sig_Tr-PNT]/[Sigmax_TrPNT=PDL1]))*(1-[SigWeight_TrPNT=Other]) + [SigWeight_TrPNT=Other]*[Sigmax_TrPNT=Other] | repeated |
| Lymph_Node.TregLN = ((Naive_T0)*([%_Tr_LN]/[%_NT_LN]) - TregLN_Int - TregLN_mAPC - [TregLN-NT] - [TregLN-PNT])*gt(mAPC,0)*(1-[CTLA4Sig_TrLN]) | repeated |
| Cancer_Diam_mm = ((6*[Cancer])/(pi*(1/((4/3)*pi*(([Cancer_Cell_Diam_um]/2000)^3)))*(1-[Tumor_Void_Fraction])*mole))^(1/3) | repeated |
| Cancer_Vol_cm3 = (4/3)*pi*(([Cancer_Diam_mm]/2)^2)*(([Cancer_Diam_mm])/2000) | repeated |
| Tumor.Cancer = (4/3)*pi*(([Starting_Cancer_Diam_mm]/2)^2)*(([Starting_Cancer_Diam_mm])/2)* (1/((4/3)*pi*(([Cancer_Cell_Diam_um]/2000)^3)))*(1-[Tumor_Void_Fraction])*mole | initial |
| Lymph_Node.NT1_Int1 = Naive_T+0.001*mole | repeated |
| [Volume_NT-Receptor_Int] = ((4*pi*(([T-mAPC_Cell_Size]/2)^2)*((mAPC_Int_P1)/mAPC_per_T_cell)*15E-3)*(L/mole))*(1E-15)*(IS_Scaling) | repeated |
| [CD28_POS-Sig_NT] = (POS_Sig_NT_CD80+POS_Sig_NT_CD86)/(POS_Sig_NT_CD80+POS_Sig_NT_CD86+NT_Int_CD28+1E-100*mole) | repeated |
| [Sig_NT=CD28] = ([CD28_POS-Sig_NT]/[Sigmax_NT=CD28]) | repeated |
| Lymph_Node.TregLN_Secrete = (Naive_T0)*([%_Tr_LN]/[%_NT_LN]) | repeated |
| CTLA4Sig_Secrete = (CTLA4S_CD80 + CTLA4S_CD86)/(mAPC_CD80 + mAPC_CD86 + 1E-100*mole)*gt(mAPC_CD80 + mAPC_CD86,0) | repeated |
| Tumor.Effector_TT_TregT = Tumor.Effector_TT-TregT_Teff | repeated |
| Tumor.Effector_TT_MDSCs = Tumor.Effector_TT-MDSCsT_Teff | repeated |
| Tumor.mAPC_T_TregT = mAPC_T-TregT_mAPCT | repeated |
| Tumor.TregT = (((Cancer*[%T_Tregs_per_Cancer]/100)) - TregT_Teff - TregT_mAPCT)*(1-[Sig_TrACTLA4S=Total]) | repeated |
| Tumor.MDSC_T = (Cancer*[%T_MDSCs_per_Cancer]/100)-MDSCsT_Teff | repeated |
| [Blood-Lymph].Effector_T_TOTAL = Effector_TP + Effector_TT_Count + Effector_TB | repeated |
| Tumor.Effector_TT_Count = Tumor.Effector_TT+Exhausted_TT | repeated |
| [CD86_receptors-per-mAPC] = 208000*mAPC_activation_level | repeated |
| [CD80_receptors-per-mAPC] = 130000*mAPC_activation_level | repeated |
| Tumor.Effector_TT_per_Treg = (Effector_TT_Count/(Cancer*[%T_Tregs_per_Cancer]/100))*ge(Cancer,10*mole) | repeated |
| Tumor.Effector_TT_per_mm3 = (Effector_TT_Count/(1000*Cancer_Vol_cm3))*ge(Cancer,10*mole) | repeated |
| Cancer_Cell_Vol_mm3 = (4/3)*pi*(([Cancer_Cell_Diam_um]/2000)^3) | initial |
| [Max_#Cells_per_mm^3] = 1/[Cancer_Cell_Vol_mm3] | initial |
| Tumor.[Ratio_T/cm3_per_max] = (Effector_TT_per_mm3/1000)/[Max_#Cells_per_mm^3] | repeated |
| [Blood-Lymph].Effector_T_TB = Effector_T * [Num_TDLN_Considered] | repeated |
| Q_T = (21*1.0599*Tumor/100)/min | initial |
| Total_Cell_T = Effector_TT+Cancer+TregT+MDSC_T+mAPC_T+APC_T | repeated |
| [%T_Tregs_per_Cancer] = [%T_MDSCs_per_Cancer]*[Tregs_per_MDSC] | repeated |

**Table S8 – Model Algebraic Equations (End)**

**Table S9 – Model Discontinuous Equation Sets (Start)**

| Trigger | Event Functions | Description |
| --- | --- | --- |
| Cancer<=1.446759259259259e+05*mole | EndTherapy = 0;  Time_Tumor_Death = time | When the tumor is less than 1mm in diameter (no longer detectable; given in cancer cell count), then therapy will no longer be administered and tumor death time will be recorded. |
| Cancer<=1*mole | Phi = 0/day | When the tumor is less than 1mm in diameter, effector T cells stop proliferating |
| CTLA4_counter_off<=time | CTLA4_counter_on = time+Dose_sched_CTLA4 - 0.0416666*day;CTLA4mAb = 0*(mg/kg) | Sets time to stop giving Anti-CTLA-4 mAb dose |
| CTLA4_counter_on<=time | CTLA4_counter_off = time + 0.0416666*day;CTLA4mAb = CTLA4mAb_Dose*EndTherapy | Sets time to start giving Anti-CTLA-4 mAb dose |
| PD1_counter_off<=time | PD1_counter_on = time+Dose_sched_PD1 - 0.0416666*day;PD1mAb = 0*(mg/kg) | Sets time to stop giving Anti-PD-1 mAb dose |
| PD1_counter_on<=time | PD1_counter_off = time+0.0416666*day;PD1mAb = PD1mAb_Dose*EndTherapy | Sets time to start giving Anti-PD-1 mAb dose |
| PDL1_counter_off<=time | PDL1_counter_on = time + Dose_sched_PDL1 - 0.0416666*day;PDL1mAb = 0*(mg/kg) | Sets time to stop giving Anti-PD-L1 mAb dose |
| PDL1_counter_on<=time | PDL1_counter_off = time + 0.0416666*day;PDL1mAb = PDL1mAb_Dose*EndTherapy | Sets time to start giving Anti-PD-L1 mAb dose |
| time >= 60*day | CTLA4mAb_Dose = CTLA4_DoseSet*(mg/kg);CTLA4_counter_on = time+0.0001*day;CTLA4_counter_off = CTLA4_counter_on + 0.0416666*day;CTLA4_change_schedule = time + 95*day;Cancer_mm_Start_Therapy = Cancer_mm_Start_Therapy*100;Time_Start_Therapy = time;Cancer_mm_Start_Therapy = Cancer_Diam_mm | Once the system reaches steady state, anti-CTLA-4 antibody is administrated |
| time >= 60*day | PDL1_counter_on = time+0.0001*day;PDL1_counter_off = PDL1_counter_on + 0.0416666*day;PDL1mAb_Dose = PDL1_DoseSet*(mg/kg);Cancer_mm_Start_Therapy = Cancer_mm_Start_Therapy*100;Time_Start_Therapy = time;PDL1_change_schedule = time + 119.9999*day;Cancer_mm_Start_Therapy = Cancer_Diam_mm | Once the system reaches steady state, anti-PD-L1 antibody is administrated |
| time >= 60*day | CTLA4mAb_Dose = CTLA4_DoseSet* (mg/kg);CTLA4_counter_on = time+0.0001*day;CTLA4_counter_off = CTLA4_counter_on + 0.0416666*day;CTLA4_change_schedule = time + 95*day;PDL1_counter_on = time+0.0001*day;PDL1_counter_off = PDL1_counter_on + 0.0416666*day;PDL1mAb_Dose = PDL1_DoseSet* (mg/kg);PDL1_change_schedule = time + 119.9999*day;Cancer_mm_Start_Therapy = Cancer_mm_Start_Therapy*100;Time_Start_Therapy = time; Cancer_mm_Start_Therapy = Cancer_Diam_mm | Once the system reaches steady state, combination therapy is administrated |
| time>=CTLA4_change_schedule | CTLA4mAb = 0*(mg/kg);CTLA4mAb_Dose = CTLA4mAb_New_Dose;New_sched_CTLA4 = 365*day + time;Dose_sched_CTLA4 = New_sched_CTLA4 | Change dosing regimen at the time scheduled |
| time>=PD1_change_schedule | PD1mAb = 0*(mg/kg);PD1mAb_Dose = PD1mAb_New_Dose;Dose_sched_PD1 = New_sched_PD1;PD1_change_schedule = 1500*day | Change dosing regimen at the time scheduled |
| time>=PDL1_change_schedule | PDL1mAb = 0*(mg/kg);PDL1mAb_Dose = PDL1mAb_New_Dose;Dose_sched_PDL1 = New_sched_PDL1;PDL1_change_schedule = 735*day | Change dosing regimen at the time scheduled |

**Table S9 – Model Discontinuous Equation Sets (End)**

**Reference**

1. Venturoli D, Rippe B. Ficoll and dextran vs. globular proteins as probes for testing glomerular permselectivity: effects of molecular size, shape, charge, and deformability. Am J Physiol Renal Physiol. 2005;288(4):F605-13.

2. Garlick DG, Renkin EM. Transport of large molecules from plasma to interstitial fluid and lymph in dogs. Am J Physiol. 1970;219(6):1595-605.

3. Yuan F, Dellian M, Fukumura D, Leunig M, Berk DA, Torchilin VP, et al. Vascular permeability in a human tumor xenograft: molecular size dependence and cutoff size. Cancer Res. 1995;55(17):3752-6.

4. Thurber GM, Dane Wittrup K. A mechanistic compartmental model for total antibody uptake in tumors. J Theor Biol. 2012;314:57-68.

5. Wang E, Kang D, Bae KS, Marshall MA, Pavlov D, Parivar K. Population pharmacokinetic and pharmacodynamic analysis of tremelimumab in patients with metastatic melanoma. J Clin Pharmacol. 2014;54(10):1108-16.

6. Baverel PG, Dubois VFS, Jin CY, Zheng Y, Song X, Jin X, et al. Population Pharmacokinetics of Durvalumab in Cancer Patients and Association With Longitudinal Biomarkers of Disease Status. Clin Pharmacol Ther. 2018;103(4):631-42.

7. Piperno-Neumann S, Diallo A, Etienne-Grimaldi MC, Bidard FC, Rodrigues M, Plancher C, et al. Phase II Trial of Bevacizumab in Combination With Temozolomide as First-Line Treatment in Patients With Metastatic Uveal Melanoma. Oncologist. 2016;21(3):281-2.

8. Wang Y, Hobbs BP, Ng CS. CT Perfusion Characteristics Identify Metastatic Sites in Liver. Biomed Res Int. 2015;2015:120749.

9. Osimani M, Bellini D, Di Cristofano C, Palleschi G, Petrozza V, Carbone A, et al. Perfusion MDCT of prostate cancer: correlation of perfusion CT parameters and immunohistochemical markers of angiogenesis. AJR Am J Roentgenol. 2012;199(5):1042-8.

10. Ng CS, Charnsangavej C, Wei W, Yao JC. Perfusion CT findings in patients with metastatic carcinoid tumors undergoing bevacizumab and interferon therapy. AJR Am J Roentgenol. 2011;196(3):569-76.

11. Wang J, Wu N, Cham MD, Song Y. Tumor response in patients with advanced non-small cell lung cancer: perfusion CT evaluation of chemotherapy and radiation therapy. AJR Am J Roentgenol. 2009;193(4):1090-6.

12. Ng CS, Wei W, Ghosh P, Anderson E, Herron DH, Chandler AG. Observer Variability in CT Perfusion Parameters in Primary and Metastatic Tumors in the Lung. Technol Cancer Res Treat. 2018;17:1533034618769767.

13. Di Nallo AM, Vidiri A, Marzi S, Mirri A, Fabi A, Carapella CM, et al. Quantitative analysis of CT-perfusion parameters in the evaluation of brain gliomas and metastases. J Exp Clin Cancer Res. 2009;28:38.

14. Finley SD, Popel AS. Predicting the effects of anti-angiogenic agents targeting specific VEGF isoforms. AAPS J. 2012;14(3):500-9.

15. Baldazzi V, Paci P, Bernaschi M, Castiglione F. Modeling lymphocyte homing and encounters in lymph nodes. BMC Bioinformatics. 2009;10:387.

16. Stefanini MO, Wu FT, Mac Gabhann F, Popel AS. A compartment model of VEGF distribution in blood, healthy and diseased tissues. BMC Syst Biol. 2008;2:77.

17. Zhu H, Melder RJ, Baxter LT, Jain RK. Physiologically based kinetic model of effector cell biodistribution in mammals: implications for adoptive immunotherapy. Cancer Res. 1996;56(16):3771-81.

18. Meijer EFJ, Blatter C, Chen IX, Bouta E, Jones D, Pereira ER, et al. Lymph node effective vascular permeability and chemotherapy uptake. Microcirculation. 2017;24(6).

19. Battaglia A, Ferrandina G, Buzzonetti A, Malinconico P, Legge F, Salutari V, et al. Lymphocyte populations in human lymph nodes. Alterations in CD4+ CD25+ T regulatory cell phenotype and T-cell receptor Vβ repertoire. Immunology. 2003;110(3):304-12.

20. Backteman K, Andersson C, Dahlin L-G, Ernerudh J, Jonasson L. Lymphocyte subpopulations in lymph nodes and peripheral blood: a comparison between patients with stable angina and acute coronary syndrome. PLoS One. 2012;7(3):e32691.

21. Höfer T, Krichevsky O, Altan-Bonnet G. Competition for IL-2 between Regulatory and Effector T Cells to Chisel Immune Responses. Front Immunol. 2012;3:1-9.

22. Kohrt HE, Nouri N, Nowels K, Johnson D, Holmes S, Lee PP. Profile of immune cells in axillary lymph nodes predicts disease-free survival in breast cancer. PLoS Med. 2005;2(9):e284.

23. Faghih Z, Erfani N, Haghshenas MR, Safaei A, Talei AR, Ghaderi A. Immune profiles of CD4+ lymphocyte subsets in breast cancer tumor draining lymph nodes. Immunol Lett. 2014;158(1-2):57-65.

24. Chemnitz JM, Parry RV, Nichols KE, June CH, Riley JL. SHP-1 and SHP-2 associate with immunoreceptor tyrosine-based switch motif of programmed death 1 upon primary human T cell stimulation, but only receptor ligation prevents T cell activation. J Immunol. 2004;173(2):945-54.

25. Gatalica Z, Snyder C, Maney T, Ghazalpour A, Holterman DA, Xiao N, et al. Programmed cell death 1 (PD-1) and its ligand (PD-L1) in common cancers and their correlation with molecular cancer type. Cancer Epidemiol Biomarkers Prev. 2014;23(12):2965-70.

26. Yearley JH, Gibson C, Yu N, Moon C, Murphy E, Juco J, et al. PD-L2 Expression in Human Tumors: Relevance to Anti-PD-1 Therapy in Cancer. Clin Cancer Res. 2017;23(12):3158-67.

27. Wang J, Yang J. Identification of CD4(+)CD25(+)CD127(-) regulatory T cells and CD14(+)HLA(-)DR(-)/low myeloid-derived suppressor cells and their roles in the prognosis of breast cancer. Biomed Rep. 2016;5(2):208-12.

28. Ahamadi M, Freshwater T, Prohn M, Li CH, de Alwis DP, de Greef R, et al. Model-Based Characterization of the Pharmacokinetics of Pembrolizumab: A Humanized Anti-PD-1 Monoclonal Antibody in Advanced Solid Tumors. CPT Pharmacometrics Syst Pharmacol. 2017;6(1):49-57.

29. Abramczyk H, Surmacki J, Kopec M, Olejnik AK, Lubecka-Pietruszewska K, Fabianowska-Majewska K. The role of lipid droplets and adipocytes in cancer. Raman imaging of cell cultures: MCF10A, MCF7, and MDA-MB-231 compared to adipocytes in cancerous human breast tissue. Analyst. 2015;140(7):2224-35.

30. Lai X, Friedman A. Combination therapy of cancer with cancer vaccine and immune checkpoint inhibitors: A mathematical model. PLoS One. 2017;12(5):e0178479.

31. Ritter AT, Asano Y, Stinchcombe JC, Dieckmann NMG, Chen B-C, Gawden-Bone C, et al. Actin Depletion Initiates Events Leading to Granule Secretion at the Immunological Synapse. Immunity. 2015;42(5):864-76.

32. Miller MJ, Hejazi AS, Wei SH, Cahalan MD, Parker I. T cell repertoire scanning is promoted by dynamic dendritic cell behavior and random T cell motility in the lymph node. Proc Natl Acad Sci U S A. 2004;101(4):998–1003.

33. Mrass P, Takano H, Ng LG, Daxini S, Lasaro MO, Iparraguirre A, et al. Random migration precedes stable target cell interactions of tumor-infiltrating T cells. J Exp Med. 2006;203(12):2749-61.

34. Ewa Bryl, Abbe N. Vallejo, Eric L. Matteson, Jacek M. Witkowski, Cornelia M. Weyand, Goronzy JJ. Modulation of CD28 expression with anti-tumor necrosis factor alpha therapy in rheumatoid arthritis. Arthritis Rheum. 2005;52(10):2996-3003.

35. Unternaehrer JJ, Chow A, Pypaert M, Inaba K, Mellman I. The tetraspanin CD9 mediates lateral association of MHC class II molecules on the dendritic cell surface. Proc Natl Acad Sci U S A. 2007;104(1):234-9.

36. Haile ST, Bosch JJ, Agu NI, Zeender AM, Somasundaram P, Srivastava MK, et al. Tumor Cell Programmed Death Ligand 1-Mediated T Cell Suppression Is Overcome by Coexpression of CD80. J Immunol. 2011;186(12):6822-9.

37. Tatari-Calderone Z, Semnani RT, B T, Nutman, Schlom J, Sabzevari H. Acquisition of CD80 by human T cells at early stages of activation: functional involvement of CD80 acquisition in T cell to T cell interaction. J Immunol. 2002;169(11):6162-9.

38. Qia Q, Liu Y, Cheng Y, Glanville J, Zhang D, Lee J-Y, et al. Diversity and clonal selection in the human T-cell repertoire. Proc Natl Acad Sci U S A. 2014;111(36):13139–44.

39. Jenkins MK, Moon JJ. The Role of Naive T Cell Precursor Frequency and Recruitment in Dictating Immune Response Magnitude. J Immunol. 2012;188(9):4135-40.

40. Zarnitsyna VI, Evavold BD, Schoettle LN, Blattman JN, Antia R. Estimating the diversity, completeness, and cross-reactivity of the T cell repertoire. Front Immunol. 2013;4.

41. Egen JG, Allison JP. Cytotoxic T Lymphocyte Antigen-4 Accumulation in the Immunological Synapse Is Regulated by TCR Signal Strength. Immunity. 2002;16(1):23-35.

42. Linsley PS, Greene JL, Tan P, Bradshaw J, Ledbetter JA, Anasetti C, et al. Coexpression and functional cooperation of CTLA-4 and CD28 on activated T lymphocytes. J Exp Med. 1992;176(6):1595-604.

43. Santa-Maria CA, Kato T, Park JH, Kiyotani K, Rademaker A, Shah AN, et al. A pilot study of durvalumab and tremelimumab and immunogenomic dynamics in metastatic breast cancer. Oncotarget. 2018;9(27):18985-96.

44. Read S GR, Izcue A, Robinson N, Mandelbrot D, Francisco L, Sharpe AH, Powrie F. Blockade of CTLA-4 on CD4+CD25+ regulatory T cells abrogates their function in vivo. J Immunol. 2006;177(7):4376-83.

45. Palsson S, Hickling TP, Bradshaw-Pierce EL, Zager M, Jooss K, O’Brien PJ, et al. The development of a fully-integrated immune response model (FIRM) simulator of the immune response through integration of multiple subset models. BMC Syst Biol. 2013;7.

46. De Boer RJ HP, Dullens HF, De Weger RA, Den Otter W. Macrophage T lymphocyte interactions in the anti-tumor immune response: a mathematical model. J Immunol. 1985;134(4):2748-58.

47. Wikipedia contributors. Durvalumab: Wikipedia, The Free Encyclopedia; 2016 [updated 8 December 2016 18:37 UTC. Available from: <https://en.wikipedia.org/wiki/Durvalumab>.

48. Henrickson SE, Mempel TR, Mazo IB, Liu B, Artyomov MN, Zheng H, et al. T cell sensing of antigen dose governs interactive behavior with dendritic cells and sets a threshold for T cell activation. Nat Immunol. 2008;9(3):282-91.

49. Marino S, Kirschner DE. The human immune response to Mycobacterium tuberculosis in lung and lymph node. J Theor Biol. 2004;227(4):463-86.

50. Macallan DC, Wallace D, Zhang Y, Lara Cd, Worth AT, Ghattas H, et al. Rapid Turnover of Effector–Memory CD4+ T Cells in Healthy Humans. J Exp Med. 2004;200(2):255-60.

51. De Boer RJ, Mohri H, Ho DD, Perelson AS. Turnover rates of B cells, T cells, and NK cells in simian immunodeficiency virus-infected and uninfected rhesus macaques. J Immunol. 2003;170(5):2479-87.

52. Qureshi OS, Kaur S, Hou TZ, Jeffery LE, Poulter NS, Briggs Z, et al. Constitutive Clathrin-mediated Endocytosis of CTLA-4 Persists during T Cell Activation. J Biol Chem. 2012;287(12):9429-40.

53. Kaur S, Qureshi OS, Sansom DM. Comparison of the Intracellular Trafficking Itinerary of CTLA-4 Orthologues. PLoS One. 2013;8(4):e60903.

54. Pentcheva-Hoang T, Chen L, Pardoll DM, Allison JP. Programmed death-1 concentration at the immunological synapse is determined by ligand affinity and availability. Proc Natl Acad Sci U S A. 2007;104(45):17765-70.

55. Linsley PS, Bradshaw J, Greene J, Peach R, Bennett KL, Mittler RS. Intracellular Trafficking of CTLA-4 and Focal Localization Towards Sites of TCR Engagement. Immunity. 1996;4(6):535-43.

56. Wild MK, Cambiaggi A, Brown MH, Davies EA, Ohno H, Saito T, et al. Dependence of T Cell Antigen Recognition on the Dimensions of an Accessory Receptor–Ligand Complex. J Exp Med. 1999;190(1):31-41.

57. Burroughs NJ, Wulfing C. Differential Segregation in a Cell-Cell Contact Interface: The Dynamics of the Immunological Synapse. Biophys J. 2002;83(4):1784-96.

58. Ruedl C KP, Bachmann M, Hess M, Karjalainen K. Anatomical origin of dendritic cells determines their life span in peripheral lymph nodes. 165. 2000;9(4910-6).

59. Domínguez PM AC. Differentiation and function of mouse monocyte-derived dendritic cells in steady state and inflammation. Immunol Rev. 2010;234(1):90-104.

60. Henrickson SE, Mempel TR, Mazo IB, Liu B, Artyomov MN, Zheng H, et al. In vivo imaging of T cell priming. Sci Signal. 2008;1(12):pt2.

61. Mempel TR, Henrickson SE, Andrian UHv. T-cell priming by dendritic cells in lymph nodes occurs in three distinct phases. Nature. 2004;427(6970):154-9.

62. Stoll S, Delon J, Brotz TM, Germain RN. Dynamic imaging of T cell-dendritic cell interactions in lymph nodes. Science. 2002;296(5574):1873-6.

63. Miller MJ, Safrina O, Parker I, Cahalan MD. Imaging the Single Cell Dynamics of CD4+ T Cell Activation by Dendritic Cells in Lymph Nodes. J Exp Med. 2004;200(7):847-56.

64. Collins AV, Brodie DW, Gilbert RJC, Iaboni A, Manso-Sancho R, Walse B, et al. The Interaction Properties of Costimulatory Molecules Revisited. Immunity. 2002;17(2):201-10.

65. He M, Chai Y, Qi J, Zhang CWH, Tong Z, Shi Y, et al. Remarkably similar CTLA-4 binding properties of therapeutic ipilimumab and tremelimumab antibodies. Oncotarget. 2017;8(40):67129-39.

66. Cheng X, Veverka V, Radhakrishnan A, Waters LC, Muskett FW, Morgan SH, et al. Structure and Interactions of the Human Programmed Cell Death 1 Receptor. J Biol Chem. 2013;288(17):11771-85.

67. Tan S, Zhang CW, Gao GF. Seeing is believing: anti-PD-1/PD-L1 monoclonal antibodies in action for checkpoint blockade tumor immunotherapy. Signal Transduct Target Ther. 2016;1:16029.

68. Tan S, Liu K, Chai Y, Zhang CW, Gao S, Gao GF, et al. Distinct PD-L1 binding characteristics of therapeutic monoclonal antibody durvalumab. Protein Cell. 2018;9(1):135-9.

69. Rabinovich GA, Gabrilovich D, Sotomayor EM. Immunosuppressive strategies that are mediated by tumor cells. Annu Rev Immunol. 2007;25:267-96.

70. Norbury CC, Malide D, Gibbs JS, Bennink JR, Yewdell JW. Visualizing priming of virus-specific CD8+ T cells by infected dendritic cells in vivo. Nat Immunol. 2002;3(3):265-71.

71. Haghighi HR, Read LR, Haeryfar SMM, Behboudi S, Sharif S. Identification of a Dual-Specific T Cell Epitope of the Hemagglutinin Antigen of an H5 Avian Influenza Virus in Chickens. PLoS ONE. 2009;4(11):e7772.

72. Sims TN, Soos TJ, Xenias HS, Dubin-Thaler B, Hofman JM, Waite JC, et al. Opposing effects of PKCtheta and WASp on symmetry breaking and relocation of the immunological synapse. Cell. 2007;129(4):773-85.

73. Celli S, Day M, Muller AJ, Molina-Paris C, Lythe G, Bousso P. How many dendritic cells are required to initiate a T-cell response? Blood. 2012;120(19):3945-8.

74. Blancas I, Garcia-Puche JL, Bermejo B, Hanrahan EO, Monteagudo C, Martinez-Agullo A, et al. Low number of examined lymph nodes in node-negative breast cancer patients is an adverse prognostic factor. Ann Oncol. 2006;17(11):1644-9.

75. Willard-Mack CL. Normal Structure, Function, and Histology of Lymph Nodes. Toxicol Pathol. 2006;34(5):409-24.

76. Native American Cancer Researc (NACR). Introduction to Lymphedema Branch: NACR; 2016 [Available from: <http://natamcancer.org/lymphedema-intro.html>.

77. Wikipedia contributors. List of lymph nodes of the human body: Wikipedia, The Free Encyclopedia; 2016 [updated 11 December 2016 00:14 UTC. Available from: <https://en.wikipedia.org/wiki/List_of_lymph_nodes_of_the_human_body>.

78. Cogswell JP, Goldberg SM, Gupta AK, Jure-Kunkel M, Wang XT, Wigginton JM. Cancer immunotherapy by disrupting pd-1/pd-l1 signaling. Google Patents; 2013.

79. Bousso P, Robey E. Dynamics of CD8+ T cell priming by dendritic cells in intact lymph nodes. Nat Immunol. 2003;4(6):579-85.

80. Lee K-H, Holdorf AD, Dustin ML, Chan AC, Allen PM, Shaw AS. T cell receptor signaling precedes immunological synapse formation. Science. 2002;295(5559):1539-42.

81. Humbert O, Riedinger JM, Vrigneaud JM, Kanoun S, Dygai-Cochet I, Berriolo-Riedinger A, et al. 18F-FDG PET-Derived Tumor Blood Flow Changes After 1 Cycle of Neoadjuvant Chemotherapy Predicts Outcome in Triple-Negative Breast Cancer. J Nucl Med. 2016;57(11):1707-12.

82. Ryu EB, Chang JM, Seo M, Kim SA, Lim JH, Moon WK. Tumour volume doubling time of molecular breast cancer subtypes assessed by serial breast ultrasound. Eur Radiol. 2014;24(9):2227-35.

83. Bousso P. T-cell activation by dendritic cells in the lymph node: lessons from the movies. Nat Rev Immunol. 2008;8(9):675-84.

84. Obst R. The timing of T cell priming and cycling. Front Immunol. 2015;6.

85. Castellino F, Huang AY, Altan-Bonnet Gg, Stoll S, Scheinecker C, Germain RN. Chemokines enhance immunity by guiding naive CD81 T cells to sites of CD41 T cell–dendritic cell interaction. Nature. 2006;440(7086):890-5.

86. Tumeh PC, Harview CL, Yearley JH, Shintaku IP, Taylor EJM, Robert L, et al. PD-1 blockade induces responses by inhibiting adaptive immune resistance. Nature. 2014;515(7528):568-71.

87. Miller MJ, Wei SH, Parker I, Cahalan MD. Two-Photon Imaging of Lymphocyte Motility and Antigen Response in Intact Lymph Node. Science. 2002;296(5574):1869-73.

88. Sallusto F, Cella M, Danieli C, Lanzavecchi A. Dendritic cells use macropinocytosis and the mannose receptor to concentrate macromolecules in the major histocompatibility complex class II compartment: downregulation by cytokines and bacterial products. J Exp Med. 1995;182(2):389-400.

89. Baey Ad, Lanzavecchia A. The role of aquaporins in dendritic cell macropinocytosis. J Exp Med. 2000;191(4):743-8.

90. Matheu MP, Othy S, Greenberg ML, Dong TX, Schuijs M, Deswarte K, et al. Imaging regulatory T cell dynamics and suppression of T cell priming mediated by CTLA4. Nat Commun. 2015;6:6219.

91. Barnes SL, Sorace AG, Loveless ME, Whisenant JG, Yankeelov TE. Correlation of tumor characteristics derived from DCE-MRI and DW-MRI with histology in murine models of breast cancer. NMR Biomed. 2015;28(10):1345-56.

92. Baldazzi V, Paci P, Bernaschi M, Castiglione F. Modeling lymphocyte homing and encounters in lymph nodes. BMC Bioinformatics. 2009;10:1-11.
